# *Carpe diene*! Europium-catalyzed [3,3] and [5,5] rearrangements of aryl-pentadienyl ethers†

**DOI:** 10.1039/d3ra05641d

**Published:** 2023-11-01

**Authors:** Maximilian Kaiser, Michael Steinacher, Florian Lukas, Peter Gaertner

**Affiliations:** a Institute of Applied Synthetic Chemistry, Technische Universität Wien Getreidemarkt 9/163 1060 Wien Austria Maximilian.kaiser@tuwien.ac.at

## Abstract

A general protocol for the europium-catalyzed rearrangement of aryl-pentadienyl-ethers is described. The mode of rearrangement and product formation in this reaction was solely determined by the aryl substituent *para* to the phenol. If the *para*-position is occupied by a substituent, the substrate undergoes a [3,3] rearrangement to the *ortho*-position to form a prochiral branched diene. In turn, a free *para*-position in the starting material allows the reaction to proceed *via* a [5,5] rearrangement and leads to a linear conjugated diene product. The severely underdeveloped and synthetically valuable [5,5] rearrangement was investigated in terms of scope and mechanism.

## Abstract 2

A catalytic and highly regioselective method for the rearrangement of aryl-pentadienyl ethers into the corresponding *ortho*- or *para*-alkylated products.

## Graphical abstract

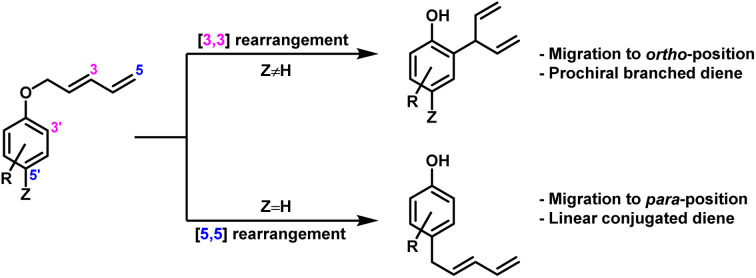

## Introduction

**Scheme 1:**
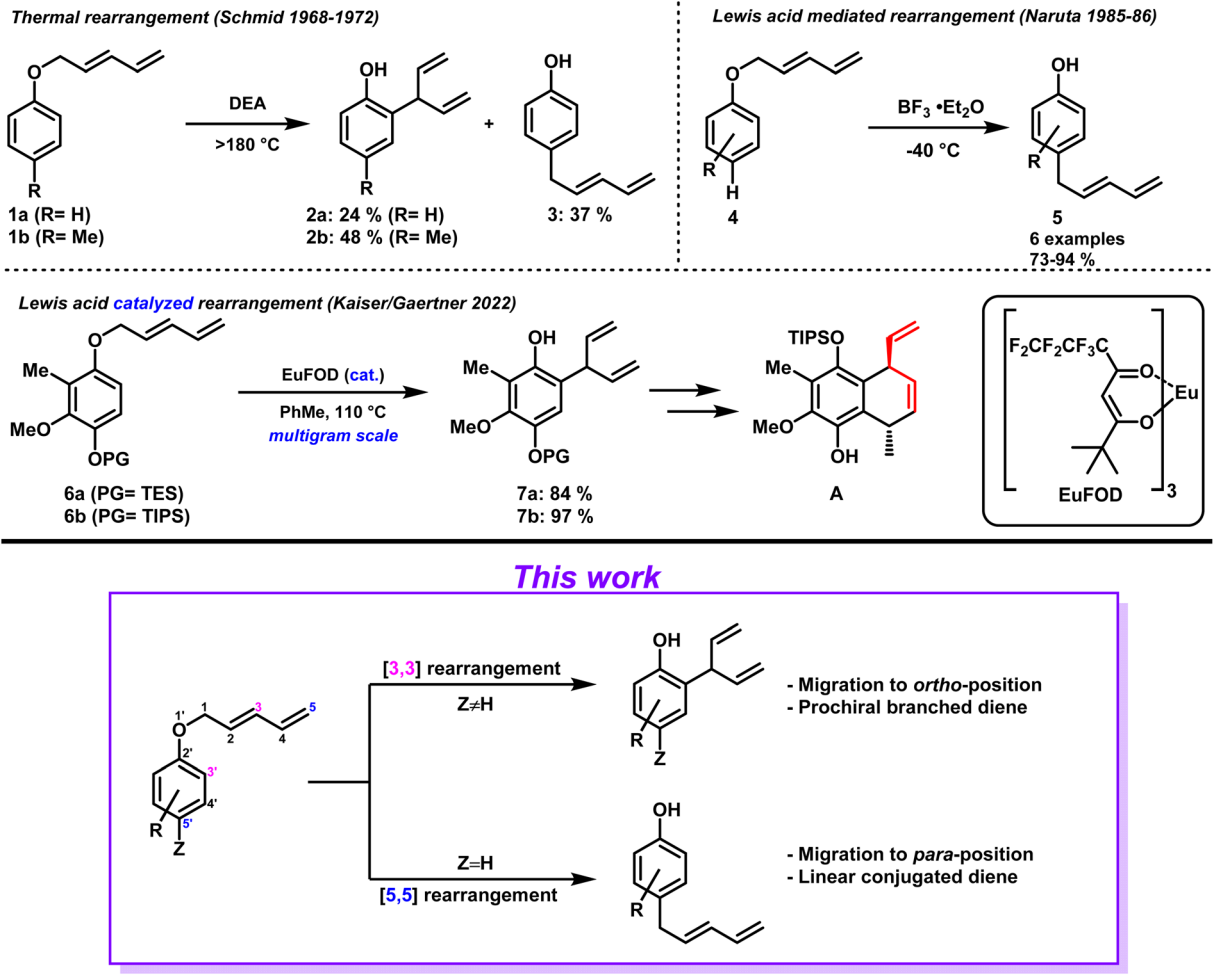
Aryl-pentadienyl ether rearrangements.

Since its initial disclosure in 1912, the Claisen rearrangement and its variants belong to the standard repertoire of organic chemistry.^1^ Among them is the aromatic Claisen rearrangement which has found wide-spread use in the synthesis of complex natural products as well as in the pharmaceutical industry.^2^ Its predictability with respect to regio- and-diastereoselectivity paired with excellent chirality transfer properties makes it a powerful tool for synthetic chemists.^3–5^ In contrast to the well-known aryl–allyl ethers, the corresponding aryl-dienyl ethers however – potentially equally useful – have been greatly overlooked by the scientific community. Although the first accounts of such a transformation were reported by Schmid *et al.* in the late 1960's to early 70's, the topic has only received little attention since. The authors described the thermal rearrangement of two different aryl-pentadienyl ethers 1a and 1b in diethylaniline (DEA).^6–8^ In the case of unsubstituted phenol, products 2a and 3 were obtained as approximately 1 : 1.5 mixture favoring *para*-rearranged product 3 (Scheme 1, top left). In the case of substrate 1b, *ortho*-substituted phenol 2b was the prevalent product formed. Obvious drawbacks of this method are the harsh reaction conditions, the lack of selectivity with respect to product distribution as well as mediocre yields. In 1985/86 Naruta *et al.* presented their findings of an improved protocol to facilitate the aryl-pentadienyl ether rearrangement.^9,10^ By employing stoichiometric amounts of BF_3_ etherate the reactions could be conducted at low temperature. As before, *para*-unsubstituted phenols 4 formed *para*-migrated products 5*via* a [5,5] rearrangement. The substrate scope of the conducted study remained limited, and the functional group tolerance continued to be a major drawback due to the applied strong acidic conditions (Scheme 1, top right). In general, transformations proceeding *via* [5,5] rearrangements have attracted little attention, although theoretically allowed according to the Woodward–Hoffmann rules.^11^ Besides the examples discussed above, literature accounts are limited to 2-pentadienyloxypyridine *N*-oxides rearrangements,^12^ DFT-calculations,^13^ gold-catalyzed cycloisomerizations,^14^ aryl-sulfoxide rearrangements,^15^ and an aryl-pentadienyl ether rearrangement^16^ that has not been recognized as [5,5] transformation. Apart from the initial report by Schmid on the preparation of branched diene aryl systems (2) by [3,3] rearrangements, methods to access such prochiral diene moieties remained difficult and literature scarce.^17–20^

In 2022, we published our synthetic efforts towards the marine terpenoid Elisabethin A, including a method confronting this issue head on. After extensive investigation we were delighted to identify conditions (cat. EuFOD,^21^ PhMe, 110 °C) that allowed conversion of aryl-pentadienyl ethers 6a and 6b into the corresponding *ortho* migrated prochiral branched dienes 7a and 7b, respectively.^22^ This [3,3] sigmatropic rearrangement could be conducted on multigram scale while obtaining high yields (Scheme 1, middle). Besides, the branched diene moiety proved to be of high synthetic value as a subsequent ring closing metathesis allowed the formation of bicyclic compound A as single diastereomer (former diene moiety in red). This protocol proved to be facial and robust and allowed easy access to branched diene scaffolds which were susceptible to desymmetrization. Prompted by these encouraging results, we decided to determine the scope of this transformation as well as expand the investigation into the corresponding [5,5] rearrangement (Scheme 1, bottom).

## Results and discussion

**Scheme 2:**
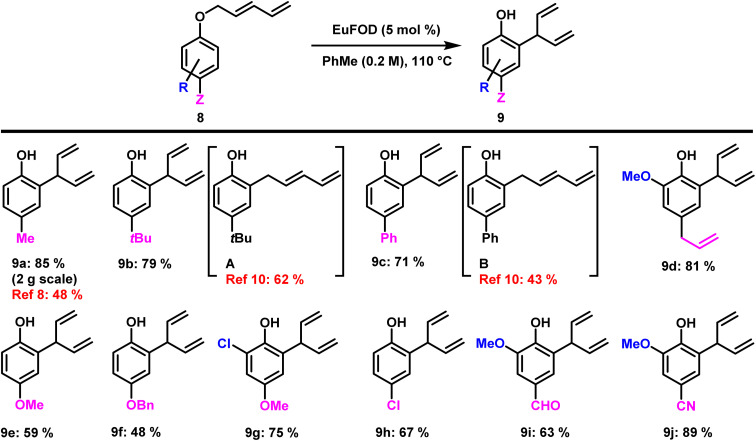
Scope of [3,3] rearrangement into *ortho* position forming branched dienes.

Besides our own findings, the applied Eu^3+^ reagent EuFOD has already proven its value in numerous synthetic applications.^23–29^ The rearrangement conditions identified in our previous work (Scheme 1, middle) are mild and selective with excellent functional group tolerance. The reaction products were obtained in high yields and with predictable regioselectivity. The different aryl-pentadienyl ethers required as substrates could be easily accessed by a modified literature procedure (see ESI†). Hence, numerous suitable aryl-dienyl ethers 8 could be subjected to Lewis-acid catalysis under standard conditions (5 mol% EuFOD, PhMe, 110 °C – see ESI† for optimization) and the results are presented in Scheme 2. Our initial choice of substrates focused on alkyl- and phenyl substitution in *para* position. As in the first example, compound 9a was obtained in 85% isolated yield, a significant improvement over the contemporary state-of-the art method. Incorporation of *t*Bu and Ph (9b and 9c) also provided the desired rearrangement products in satisfying yields. In contrast to these findings, Naruta reported the formation of *ortho* rearranged compounds A and B bearing a linear diene-chain obtained by an intermolecular pathway. Eugenol-derived compound 9d was prepared in good yields without alteration of the *para*-allyl moiety. Similarly, methoxy and benzyloxy substituted phenols (9e and 9f) proved to be well tolerated. Functional group tolerance towards aromatic halides was exemplified by chlorinated compounds (9g and 9h). Furthermore, aldehyde and nitrile (9i and 9j) groups were well tolerated, highlighting the mild nature of this transformation. Limitations of this rearrangement are presented by substrates bearing strong electron withdrawing groups in *para*-position (see ESI† for details).

**Scheme 3:**
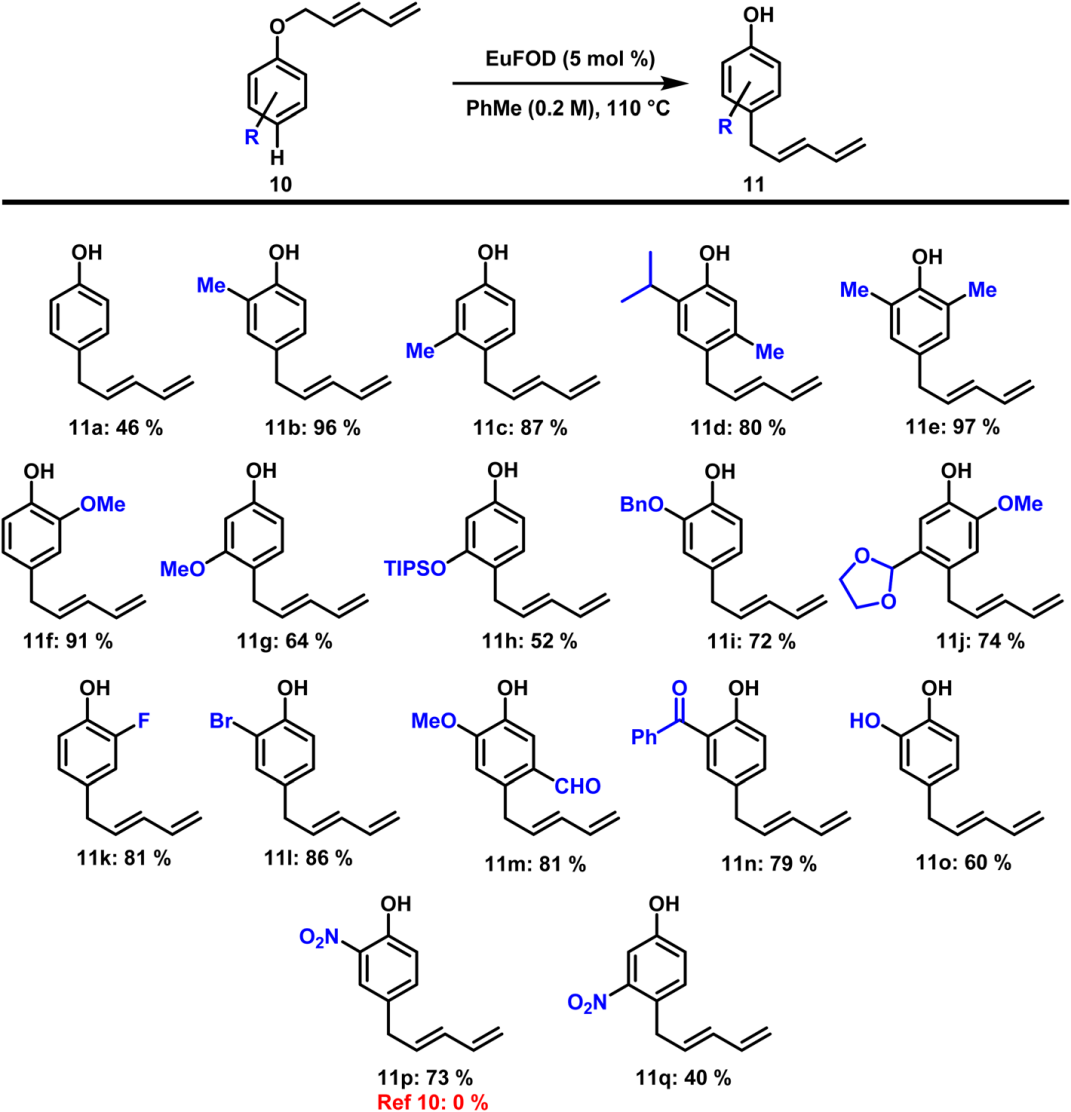
Scope of [5,5] rearrangement into *para* position forming linear conjugated dienes.

With these results at hand, we then turned our attention to substrates 10 possessing an unsubstituted *para*-position (Z = H). Comparably to the results depicted in Scheme 2, these ethers smoothly underwent the desired rearrangement, this time rearranging to the *para*-position. Our studies commenced with the transformation of phenol-derived (R = H) dienyl ether and compound 11a was obtained in moderate yield of 46% (Scheme 3). Incorporation of alkyl substituents in *ortho*- and *meta*-position (R = C_*x*_H_*y*_) significantly increased the isolated yield of the rearranged products (11b, 11c, 11d, and 11e). Widely used phenol protection groups such as methyl, silyl, and benzyl ethers (11f, 11g, 11h and 11i) were well tolerated, as were acetals (11j). Besides chlorine, substrates containing fluorine and bromine (11k and 11l) also gave the desired products in good yields under the applied conditions. Carbonyl functionalities such as aldehydes and ketones (11m and 11n) remained unchanged during the reaction and the rearranged materials were obtained in good yield. The presence of a free phenolic hydroxyl group did not interfere with reactivity and the resulting catechol (11o) was formed in 60% yield. Especially noteworthy is the formation of two compounds bearing strong electron withdrawing nitro groups in *ortho*- and *meta*-position. While inaccessible *via* current literature procedures, our application of EuFOD-catalysis allowed the preparation of 11p for the first time in 73% yield. The corresponding *meta*-substituted nitro compound 11q was obtained in 40% yield, again demonstrating the broad functional group tolerance of this methodology.

### Mechanistic studies

**Scheme 4:**
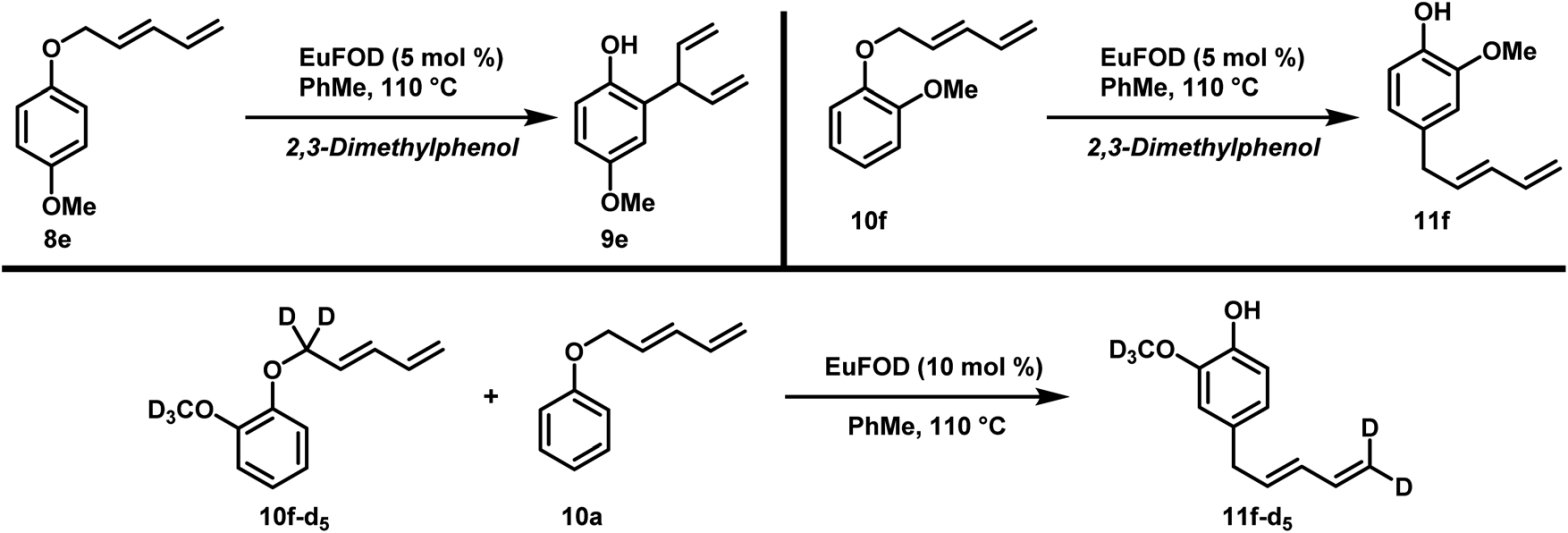
Cross-over experiments.

To gain insight into this interesting transformation we decided to carry out some mechanistic studies. First of all, we wanted to investigate whether the products obtained in Schemes 2 and 3 were derived from an inter- or an intramolecular pathway through a cross-over experiment. First, compound 8e together with 2,3-dimethylphenol was subjected to standard conditions (Scheme 4, top left).^10^ To our delight, no cross-over product was detected in the crude mixture and 9e was obtained, whereas 2,3-dimethylphenol remained unchanged. In the rearrangement of ether 10f together with scavenger phenol, a similar outcome was observed (Scheme 4, top right). Only the desired product 11f was detectable in the crude reaction mixture of the cross-over experiment and no diene-chain migration onto 2,3-dimethylphenol was detected. Additionally, an equimolar mixture of deuterium labeled compound 10f-d_5_ and ether 10a was subjected to standard rearrangement conditions (Scheme 4, bottom). To our delight, compound 11f-d_5_ was the only product isolated in this reaction, and no transfer of the deuterated diene chain was observed.

**Scheme 5:**
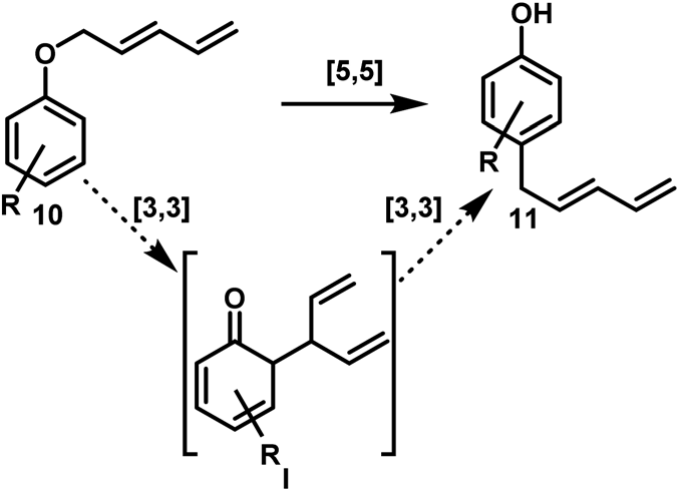
Possible reaction pathways from ether 10 to phenol 11.

Next, we investigated the reaction pathway of the rearrangement leading to *para*-alkylation. From type-10 aryl-pentadienyl ethers, two possible mechanisms are conceivable (Scheme 5). The first consists of a concerted [5,5] rearrangement directly leading to type-11 products. The second pathway would commence with a [3,3] rearrangement forming intermediate I, which in turn would undergo a second [3,3] rearrangement giving rise to type-11 products.

**Scheme 6:**
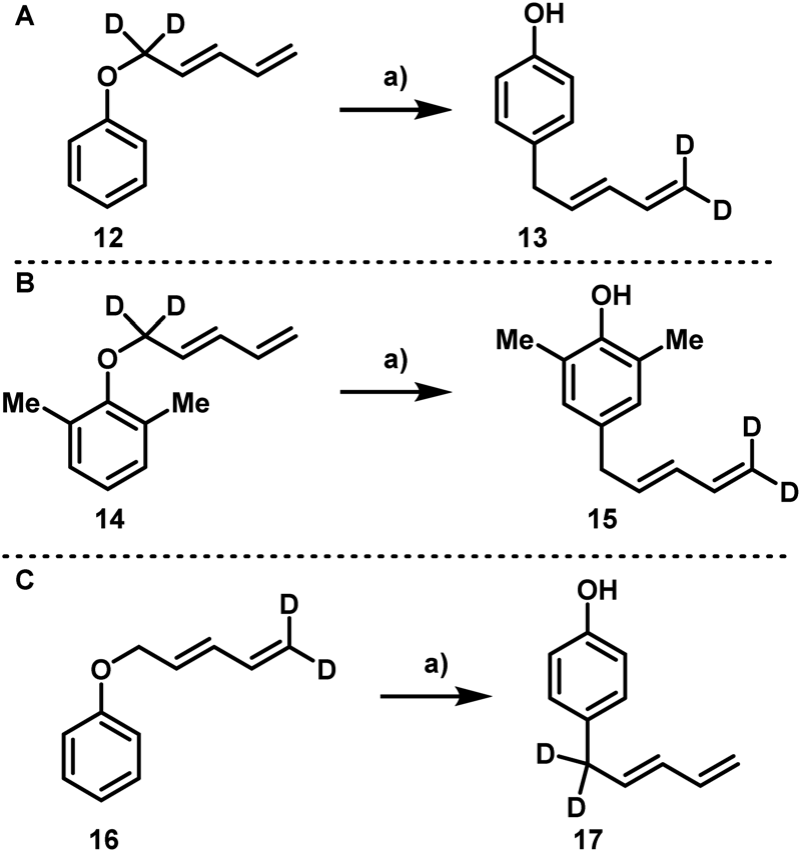
Deuterium labeling experiments. Reaction conditions: (a) EuFOD (5 mol%), PhMe (0.2 M), 110 °C.

In order to determine the underlying mechanism, a series of deuterium labeling experiments were conducted. First, compound 12 was subjected to standard conditions and compound 13 was obtained as the only product (Scheme 6A). Similarly, the conversion of ether 14 into compound 15 also proceeded smoothly (Scheme 6B). In both cases, the rearranged material possessing a terminal CD_2_ moiety was the only product obtained. To rule out the influence of a kinetic effect during a conceivable double [3,3] rearrangement, compound 16 bearing a terminal CD_2_ group was subjected to rearrangement and compound 17 was the sole product isolated (Scheme 6C).

These results very strongly suggest the underlying mechanism consisting of a [5,5] rearrangement rather than two consecutive [3,3] rearrangements. To further investigate the origin of the *ortho*-branched and the *para*-linear dienes an interconversion experiment was conducted.

**Scheme 7:**
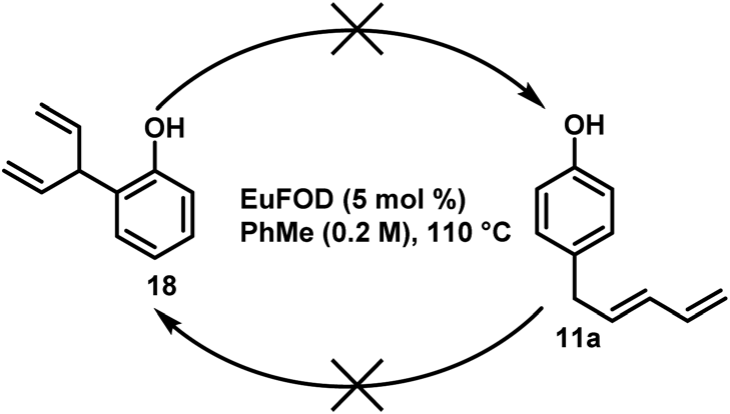
Interconversion experiments.

Here, dienes 18 and 11a were subjected to standard conditions and even after prolonged heating (>10 h) no interconversion of the two products was detected and both substrates were recovered unchanged (Scheme 7). This result further strengthens the evidence that the *ortho*- and the *para*-alkylated rearrangement products arise from two separate reaction pathways.

## Conclusions

The established method represents a significant improvement of the current literature conditions featuring either high temperatures or stoichiometric application of strong Lewis-acid. We have identified mild and selective reaction conditions applying catalytic amounts of EuFOD to facilitate the rearrangement of aryl-pentadienyl ethers. Depending on the aryl-substitution pattern, two possible products are formed. *Para*-substituted substrates undergo a [3,3] rearrangement to form branched diene moieties in *ortho*-position. The functional and protecting group tolerance in this reaction was shown to be broad, although strong electron withdrawing groups currently present a limitation. Substrates possessing an unsubstituted *para*-position proceed *via* a [5,5] rearrangement giving rise to linear, conjugated dienes in *para*-position. The functional group tolerance in this transformation proved to be excellent, including ketones, free phenols, nitro groups and aromatic halides. Additionally, to the presented scope and limitations the conducted mechanistic studies gave a clear picture of the underlying reaction pathway. The formation of the *para*-rearranged products followed a [5,5] reaction mechanism which was unambiguously proven by deuterium-labeling experiments.

## Conflicts of interest

There are no conflicts to declare.

## Supplementary Material

RA-013-D3RA05641D-s001
