# Supplementary material for: Carpe diene! Europium-catalyzed [3,3] and [5,5] rearrangements of aryl-pentadienyl ethers
Source: RSC Adv. 2023 Nov 1;13(46):32077–82. doi: 10.1039/d3ra05641d (PMC10618941; doi:10.1039/d3ra05641d)
Supplement: RA-013-D3RA05641D-s001 [file RA-013-D3RA05641D-s001.pdf]

Electronic Supplementary Material (ESI) for RSC Advances.
This journal is © The Royal Society of Chemistry 2023

Carpe Diene! Europium-catalyzed [3,3] and [5,5] rearrangements of aryl-pentadienyl ethers

Maximilian Kaiser*, Michael Steinacher, Florian Lukas, Peter Gaertner

Table of contents:

General Information______1

Optimization of reaction conditions______1

Experimental Section______4

NMR spectra______27

References ______101

General Information

The reactions were performed as described in the general procedures. All reactions were stirred magnetically. Dry acetone and K_2CO_3 were purchased from Sigma Aldrich. Phenols were purchased from commercial suppliers and used as received. Non-commercial phenols were prepared as stated in the references given in the compound description. General procedure A is a modification of a known literature procedure. Dry toluene was retrieved from an Innovative Technologies PureSolv system. ^{1}H and $_{13}C$ NMR spectra were recorded on a Bruker AC 400 at 400 and 101 MHz; AC 600 at 600 and 151 MHz using the solvent peak as reference. ^{13}C NMR spectra were run in proton-decoupled mode. Multiplicities of ^{1}H signals were referred to as s (singlet), d (doublet), t (triplet), q (quartet) and more complex patterns or m (multiplet). TLC-analysis was done with precoated aluminum-backed plates (Silica gel 60 F254, Merck). Compounds were visualized by submerging in: an acidic phosphomolybdic acid / Cerium sulphate solution, $KMnO_4$, Vanillin or Anisaldehyde and dried with a heat gun. Column chromatography was carried out with silica gel Merck 60. Eluent systems refer to volumetric ratios, e.g., 4:1 =80 %: 20 %. HR-MS measurements were carried out in acetonitrile, methanol, water or a mixture on an Agilent 1100/1200 HPLC with binary pumps, a degassed and a column thermostat and an Agilent 6230 AJS ESI-TOF mass spectrometer. Melting points were determined with a Leica Galen III Kofler hot-stage apparatus.

Optimization of reaction conditions

General procedure for solvent & temperature screening:

An oven-dried screw vial was charged with starting material (1 equiv) dissolved in dry solvent (c= 0.2 M). The solution was degassed by ten vacuo/Ar cycles. Then, EuFOD (5 mol %) was added and the solution was degassed again ten times and the reaction was heated in a metal heating block for the given time. In case TLC confirmed full or partial conversion, the crude material was filtered through a short plug of silica to remove the catalyst. Then, conversion was determined by crude ^{1}H-NMR. The material was purified by column chromatography (petroleum ether/ ethyl acetate 12:1 or 20:1) to obtain the desired product in the given yield.

Table 1. Solvent and temperature optimization for the [5,5] rearrangement

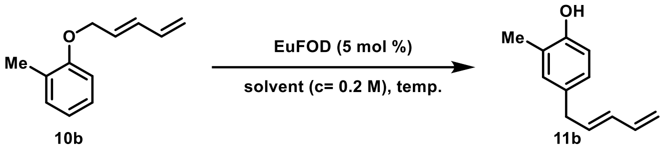

Entry	Solvent	Temperature (°C)	Time	Results
1	DCM	Reflux	22 h	No conversion
2	ACN	Reflux	22 h	No conversion
3	THF	Reflux	22 h	No conversion
4	Ethyl acetate	Reflux	22 h	No conversion
5	$CHCl_3$	Reflux	22 h	50 % conversion[b]
6	DCE	70 [a]	22 h	55 % conversion[b]
7	Heptane	70 [a]	22 h	40 % conversion[b]
8	Hexane	70[a]	24 h	77 % isolated yield
9	Heptane	100[a]	24 h	77 % isolated yield
10	PhMe	70 [a]	22 h	56 % isolated yield (93 % conversion[b])
11	PhMe	100[a]	24 h	80 % isolated yield
12	PhMe	110	2 h	96 % yield

[a] Heating block temperature; [b] Determined by ^{1}H-NMR of the crude material.

Table 2. Solvent and temperature optimization for the [3,3] rearrangement

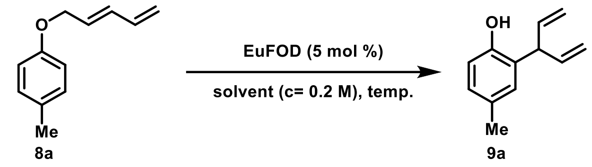

Entry	Solvent	Temperature (°C)	Time	Results
1	PhMe	110[a]	4 h	85 % isolated yield
2	Hexane	Reflux	23 h	No conversion
3	Heptane	100[a]	23 h	68 % isolated yield
4	PhMe	100[a]	23 h	53 % isolated yield
5	PhMe	90[a]	11 h	No conversion

[a] Heating block temperature; [b] Determined by ^{1}H-NMR of the crude material.

Table 3 Lewis-Acid screening

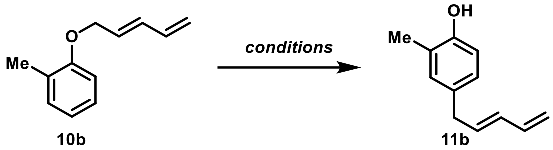

Entry	Solvent (c= 0.2 M)	Lewis-Acid (mol %)	Temperature (°C)	Time	Results
1	PhMe	$AlMe_3$ (10 mol%)	110	2 h	60 % yield
2	PhMe	$AlCl_3$ (10 mol%)	110	2 min	Decomposition
3	PhMe	HoFOD (10 mol %)	110	2 h	94 % yield
4	PhMe	$Eu(OTf)_3$ (10 mol %)	110	20 h	No conversion
5	PhMe	$Zn(OTf)_2$ (10 mol %)	110	22	No conversion
6	PhMe	$InBr_3$ (10 mol %)	110	10 min	Decomposition
7	PhMe	$Sc(OTf)_3$ (10 mol %)	110	1 h	28 % crude yield
8	CH_2Cl_2	$AlMe_3$ (10 mol%)	-50 °C to rt	5 h	No conversion
9	PhMe	BF_3 etherate (10 mol %)	23	1 h	22 % crude yield
10	PhMe	$B(C_6F_5)_3$ (10 mol %)	23	1 h	54 % crude yield
11	PhMe	Et_2AlCl (10 mol %)	23	1 h	33 % crude yield

Experimental section

General procedure A – Etherification by modified literature procedure[1]

An oven-dried screw cap vial was charged with phenol (1 equiv), K_2CO_3 (1.6 equiv), and literature known (*E*)-5-bromopenta-1,3-diene [2](1.2 equiv) in dry acetone (c= 0.2 M) and the reaction mixture was heated to reflux. After TLC confirmed full conversion, it was quenched by addition of H_2O. The aqueous layer was extracted with DCM three times. The combined organic layer was dried over $MgSO_4$ and concentrated in vacuo. The crude material was received either sufficiently pure or was subjected to column chromatography.
Note: Products may contain up to 5 % of Z-isomer as inseparable side-products.

Type 8 Aryl-dienyl ethers

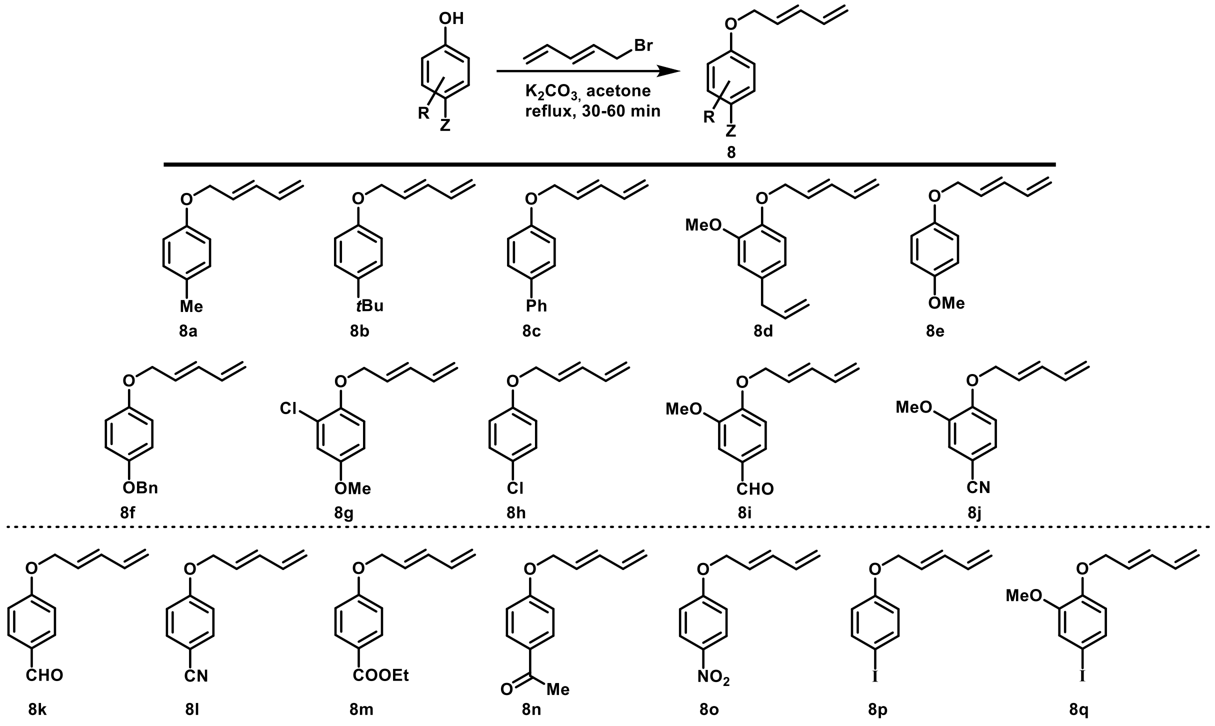

Scheme S1

(*E*)-1-methyl-4-(penta-2,4-dien-1-yloxy)benzene (8a)

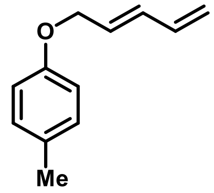

The title compound was synthesized from *p*-cresol (147 mg, 1.36 mmol) following **general procedure A**. The crude material was purified by column chromatography (petroleum ether/ ethyl acetate 10:1) to provide the desired product **8a** as yellow oil in 65 % yield (155 mg, 0.90 mmol); ^{1}H NMR (400 MHz, $CDCl_3$) d 7.14 – 7.05 (m, 2H), 6.88 – 6.79 (m, 2H), 6.47 – 6.33 (m, 2H), 6.00 – 5.85 (m, 1H), 5.33 – 5.20 (m, 1H), 5.20 – 5.11 (m, 1H), 4.56 (d, *J* = 5.2 Hz, 2H), 2.30 (s, 3H); ^{13}C-NMR (101 MHz, $CDCl_3$): δ = 156.6, 136.3, 133.7, 130.2, 130.0, 128.8, 118.2, 114.7, 68.3, 20.6; HRMS (ESI): exact mass calculated for $C_{12}H_{15}O^+$ [$(M + H)^+$], 175.1117; found 175.1130.

(*E*)-1-(tert-butyl)-4-(penta-2,4-dien-1-yloxy)benzene (8b)

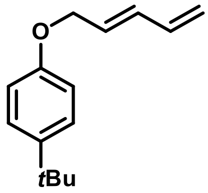

The title compound was synthesized from 4-(tert-butyl) phenol (203 mg, 1.35 mmol) following **general procedure A**. The crude material was purified by column chromatography (petroleum ether/ ethyl acetate 20:1) to provide the desired product **8b** as yellow needles in 66 % yield (193 mg,0.89 mmol); ^{1}H-NMR (400 MHz, $CDCl_3$): δ = 7.32 – 7.28 (m, 2H), 6.90 – 6.81 (m, 2H), 6.45 – 6.31 (m, 2H), 5.99 – 5.85 (m, 1H), 5.36 – 5.20 (m, 1H), 5.17 – 5.08 (m, 1H), 4.56 (d, *J* = 5.1 Hz, 2H), 1.30 (s, 9H);^{13}C-NMR (101 MHz, $CDCl_3$): δ = 156.5, 143.7, 136.3, 133.7, 128.8, 126.4, 118.2, 114.3, 68.2, 34.2, 31.7; HRMS (ESI): exact mass calculated for $C_{15}H_{21}O^+$ [(M + H)$^+$], 217.1587; found 217.1586; m.p. 33-36 °C.

(*E*)-4-(Penta-2,4-dien-1-yloxy)-1,1'-biphenyl (8c)

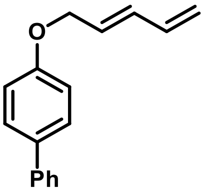

The title compound was synthesized from [1,1'-biphenyl]-4-ol (205 mg, 1.20 mmol) following **general procedure A**. The crude material was purified by column chromatography (petroleum ether/ ethyl acetate 10:1) to provide the desired product **8c** as white amorphous solid in 96 % yield (239 mg, 1.01 mmol); ^{1}H-NMR (400 MHz, $CDCl_3$): δ = 7.61 – 7.50 (m, 4H), 7.48 – 7.38 (m, 2H), 7.37 – 7.28 (m, 1H), 7.05 – 6.97 (m, 2H), 6.49 – 6.33 (m, 2H), 6.03 – 5.88 (m, 1H), 5.40 – 5.23 (m, 1H), 5.21 – 5.13 (m, 1H), 4.63 (d, *J* = 5.2 Hz, 2H); ^{13}C-NMR (101 MHz, $CDCl_3$): δ = 158.3, 140.9, 136.2, 134.1, 133.9, 128.9, 128.4, 128.3, 126.9, 126.8, 118.4, 115.1, 68.3; HRMS (ESI): exact mass calculated for $C_{17}H_{17}O^+$ [(M + H)$^+$], 237.1274; found 237.1291; m.p. 73-75 °C.

(*E*)-4-allyl-2-methoxy-1-(penta-2,4-dien-1-yloxy)benzene (8d)

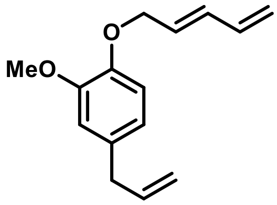

The title compound was synthesized from 4-allyl-2-methoxyphenol (150 mg, 0.91 mmol) following **general procedure A**. The crude material was purified by column chromatography (petroleum ether/ ethyl acetate 20:1) to provide the desired product **8d** as colorless oil in 60 % yield (126 mg, 0.55 mmol); ^{1}H-NMR (400 MHz, $CDCl_3$): δ = 6.82 (d, *J* = 8.0 Hz, 1H), 6.75 – 6.67 (m, 2H), 6.44 – 6.29 (m, 2H), 6.04 – 5.87 (m, 2H), 5.34 – 5.18 (m, 1H), 5.15 – 5.03 (m, 3H), 4.62 (d, *J* = 5.4 Hz, 2H), 3.87 (s, 3H), 3.34 (d, *J* = 6.7 Hz, 2H); ^{13}C-NMR (101 MHz, $CDCl_3$): δ = 157.3, 154.2, 145.5, 144.0, 141.7, 141.0, 136.6, 128.2, 125.9, 123.5, 121.4, 120.1, 77.2, 63.7, 47.7; HRMS (ESI): exact mass calculated for $C_{15}H_{19}O_2^+$ [(M + H)$^+$], 231.1380; found 231.1391.

(*E*)-1-methoxy-4-(penta-2,4-dien-1-yloxy)benzene (8e)

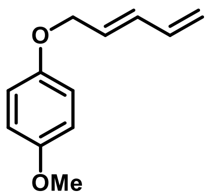

The title compound was synthesized from 4-methoxyphenol (193 mg, 1.55 mmol) following **general procedure A**. The crude material was purified by column chromatography (petroleum ether/ ethyl acetate 20:1) to provide the desired product **8e** as yellow oil in 58 % yield (172 mg,0.90 mmol); ^{1}H-NMR (600 MHz, $CDCl_3$): δ = 6.89 – 6.75 (m, 4H), 6.42 – 6.34 (m, 2H), 5.93 – 5.87 (m, 1H), 5.29 – 5.22 (m, 1H), 5.17 – 5.11 (m, 1H), 4.53 (d, *J* = 5.2 Hz, 2H), 3.77 (s, 3H); ^{13}C-NMR (151 MHz, $CDCl_3$): δ = 154.1, 152.9, 136.3, 133.7, 128.8, 118.2, 116.2, 115.9, 114.9, 114.8, 68.9, 55.8; HRMS (ESI): exact mass calculated for $C_{12}H_{15}O_2^+$ [(M + H)$^+$], 191.1067; found 191.1067.

(*E*)-1-(benzyloxy)-4-(penta-2,4-dien-1-yloxy)benzene (8f)

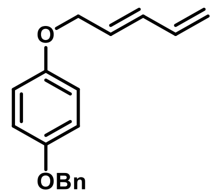

The title compound was synthesized from 4-(benzyloxy)phenol[32] (200 mg, 1.00 mmol) following **general procedure A**. The crude material was purified by column chromatography (petroleum ether/ ethyl acetate 20:1) to provide the desired product **8f** as white solids in 83 % yield (220 mg, 0.83 mmol); ^{1}H-NMR (400 MHz, $CDCl_3$): δ = 7.50 – 7.30 (m, 5H), 6.98 – 6.83 (m, 4H), 6.48 – 6.32 (m, 2H), 5.99 – 5.85 (m, 1H), 5.32 – 5.22 (m, 1H), 5.19 – 5.12 (m, 1H), 5.03 (s, 2H), 4.54 (d, *J* = 5.2 Hz, 2H); ^{13}C-NMR (101 MHz, $CDCl_3$): δ = 153.3, 153.1, 137.4, 136.3, 133.7, 128.8, 128.7, 128.0, 127.6, 118.2, 115.9, 115.8, 70.8, 68.9; HRMS (ESI): exact mass calculated for $C_{18}H_{19}O_2^+$ [(M + H)$^+$], 267.1380; found 267.1389; m.p. 52-53 °C.

(*E*)-2-chloro-4-methoxy-1-(penta-2,4-dien-1-yloxy)benzene (8g)

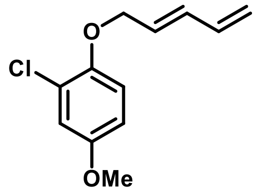

The title compound was synthesized from 2-Chloro-4-methoxyphenol (100 mg, 0.63 mmol) following **general procedure A**. The crude material was sufficiently pure, and no purification was necessary. The desired product **8g** was obtained as yellow oil in 99 % yield (141 mg, 0.63 mmol); ^{1}H-NMR (400 MHz, $CDCl_3$): δ = 6.95 (d, *J* = 3.0 Hz, 1H), 6.88 (d, *J* = 9.0 Hz, 1H), 6.74 (dd, *J* = 9.0, 3.0 Hz, 1H), 6.42 – 6.34 (m, 2H), 5.98 – 5.83 (m, 1H), 5.29 – 5.21 (m, 1H), 5.17 – 5.12 (m, 1H), 4.59 (d, *J* = 5.0 Hz, 2H), 3.76 (s, 3H); ^{13}C-NMR (101 MHz, $CDCl_3$): δ = 154.3, 148.5, 136.2, 133.9, 128.3, 124.1, 118.4, 116.1, 115.9, 113.0, 70.4, 55.9; HRMS (ESI): exact mass calculated for $C_{12}H_{14}ClO_2^+$ [(M + H)$^+$], 225.0677; found 225.0677.

(*E*)-1-chloro-4-(penta-2,4-dien-1-yloxy)benzene (8h)

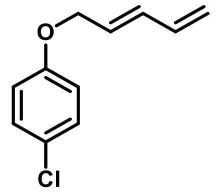

The title compound was synthesized from 4-chlorophenol (400 mg, 3.11 mmol) following **general procedure A**. The crude material was purified by column chromatography (petroleum ether/ ethyl acetate 20:1) to provide the desired product **8h** as yellow oil in 93 % yield (566 mg, 2.91 mmol); ^{1}H-NMR (400 MHz, $CDCl_3$): δ = 7.28 – 7.19 (m, 2H), 6.89 – 6.80 (m, 2H), 6.45 – 6.31 (m, 2H), 5.96 – 5.77 (m, 1H), 5.30 – 5.23 (m, 1H), 5.19 – 5.14 (m, 1H), 4.55 (d, *J* = 5.2 Hz, 2H); ^{13}C-NMR (101 MHz, $CDCl_3$): δ = 157.3, 136.1, 134.1, 129.4, 128.0, 118.6, 116.1, 113.0, 68.5; HRMS (ESI): exact mass calculated for $C_{11}H_{12}ClO^+$ [(M + H)$^+$], 195.0571; found 195.0573.

(*E*)-3-methoxy-4-(penta-2,4-dien-1-yloxy)benzaldehyde (8i)

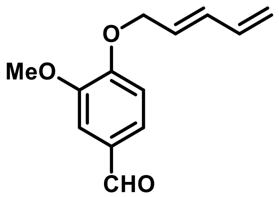

The title compound was synthesized from 4-hydroxy-3-methoxybenzaldehyde (100 mg, 0.66 mmol) following **general procedure A**. The crude material was purified by column chromatography (petroleum ether/ ethyl acetate 8:1) to provide the desired product **8i** as colorless oil in 92 % yield (133 mg,0.61 mmol) which solidified in the freezer; ^{1}H-NMR (400 MHz, $CDCl_3$): δ = 9.84 (s, 1H), 7.44 – 7.40 (m, 2H), 6.97 (d, *J* = 8.5 Hz, 1H), 6.44 – 6.29 (m, 2H), 6.00 – 5.86 (m, 1H), 5.26 (d, *J* = 16.0 Hz, 1H), 5.16 (d, *J* = 8.4 Hz, 1H), 4.73 (d, *J* = 5.4 Hz, 2H), 3.93 (s, 3H); ^{13}C-NMR (101 MHz, $CDCl_3$): δ = 191.0, 153.6, 150.0, 135.9, 135.0, 130.3, 127.2, 126.8, 119.0, 112.0, 109.4, 69.3, 56.2; HRMS (ESI): exact mass calculated for $C_{13}H_{15}O_3^+$ [(M + H)$^+$], 219.1016; found 219.1017; m.p. 78-81 °C.

(*E*)-3-Methoxy-4-(penta-2,4-dien-1-yloxy)benzonitrile (8j)

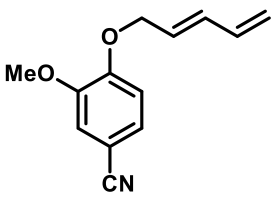

The title compound was synthesized from 4-hydroxy-3-methoxybenzonitrile (200 mg, 1.34 mmol) following **general procedure A**. The crude material was purified by column chromatography (petroleum ether/ ethyl acetate 5:1) to provide the desired product **8j**as yellow solids in 96 % yield (277 mg, 1.29 mmol); ^{1}H-NMR (400 MHz, $CDCl_3$): δ = 7.23 (dd, *J* = 8.3, 1.9 Hz, 1H), 7.07 (d, *J* = 1.9 Hz, 1H), 6.88 (d, *J* = 8.4 Hz, 1H), 6.41 – 6.30 (m, 2H), 5.96 – 5.82 (m, 1H), 5.33 – 5.21 (m, 1H), 5.19 – 5.11 (m, 1H), 4.70 – 4.64 (m, 2H), 3.88 (s, 3H); ^{13}C-NMR (101 MHz, $CDCl_3$): δ = 151.9, 149.5, 135.8, 135.0, 126.9, 126.3, 119.3, 119.1, 114.3, 112.9, 104.1, 69.2, 56.2; HRMS (ESI): exact mass calculated for $C_{13}H_{14}NO_2^+$ [$(M + H)^+$], 216.1019; found 216.1022; m.p. 85-86 °C.

(*E*)-4-(Penta-2,4-dien-1-yloxy)benzaldehyde (8k)

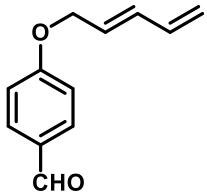

The title compound was synthesized from 4-hydroxybenzaldehyde (250 mg, 2.05 mmol) following **general procedure A**. The crude material was purified by column chromatography (petroleum ether/ ethyl acetate 5:1) to provide the desired product **8k** as yellow oil in 97 % yield (373 mg, 1.98 mmol); ^{1}H-NMR (400 MHz, $CDCl_3$): δ = 10.09 (s, 1H), 8.00 – 7.92 (m, 2H), 7.09 – 7.01 (m, 2H), 6.45 – 6.33 (m, 2H), 5.97 – 5.83 (m, 1H), 5.36 – 5.22 (m, 1H), 5.21 – 5.12 (m, 1H), 4.67 (d, *J* = 5.3 Hz, 2H); ^{13}C-NMR (101 MHz, $CDCl_3$): δ= 191.6, 163.8, 135.9, 134.6, 132.3, 129.9, 127.2, 119.0, 115.1, 68.5; HRMS (ESI): exact mass calculated for $C_{11}H_{11}O^+$ [$(M - CHO)^+$], 159.0804; found 159.0814.

(*E*)-4-(penta-2,4-dien-1-yloxy)benzonitrile (8l)

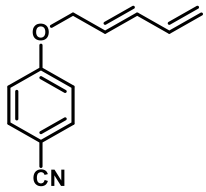

The title compound was synthesized from 4-hydroxybenzonitrile (150 mg, 1.26 mmol) following **general procedure A**. The crude material was purified by column chromatography (petroleum ether/ ethyl acetate 20:1) to provide the desired product **8l** as white solids in 84 % yield (196 mg, 1.06 mmol); ^{1}H-NMR (400 MHz, $CDCl_3$): δ = 7.62 – 7.53 (m, 2H), 7.00 – 6.91 (m, 2H), 6.45 – 6.30 (m, 2H), 5.94 – 5.79 (m, 1H), 5.40 – 5.22 (m, 1H), 5.22 – 5.12 (m, 1H), 4.62 (d, *J* = 5.1 Hz, 2H); ^{13}C-NMR (101 MHz, $CDCl_3$): δ= 161.9, 135.8, 134.7, 134.1, 126.9, 119.3, 119.1, 115.6, 104.2, 68.5; HRMS (ESI): exact mass calculated for $C_{12}H_{11}NO^-$ [$(M)^-$], 185.0846; found 185.0843; m.p. 50-52 °C.

Ethyl (*E*)-4-(penta-2,4-dien-1-yloxy)benzoate (8m)

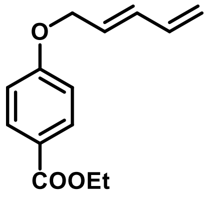

The title compound was synthesized from ethyl 4-hydroxybenzoate (204 mg, 1.23 mmol) following **general procedure A**. The crude material was purified by column chromatography (petroleum ether/ ethyl acetate 15:1) to provide the desired product **8m** as yellow solids in 95 % yield (272 mg, 1.17 mmol); ^{1}H-NMR (400 MHz, $CDCl_3$): δ = 8.03 – 7.95 (m, 2H), 6.96 – 6.88 (m, 2H), 6.45 – 6.29 (m, 2H), 5.96 – 5.82 (m, 1H), 5.34 – 5.20 (m, 1H), 5.20 – 5.10 (m, 1H), 4.62 (d, *J* = 5.1 Hz, 2H), 4.34 (q, *J* = 7.1 Hz, 2H), 1.37 (t, *J* = 7.1 Hz, 3H); ^{13}C-NMR (101 MHz, $CDCl_3$): δ= 166.5, 162.4, 136.0, 134.3, 131.7, 127.6, 123.2, 118.8, 114.4, 68.3, 60.8, 14.5; HRMS (ESI): exact mass calculated for $C_{14}H_{16}NaO_3^+$ [$(M + Na)^+$], 255.0992; found 255.1012; m.p. 47-48 °C.

(*E*)-1-(4-(penta-2,4-dien-1-yloxy)phenyl)ethan-1-one (8n)

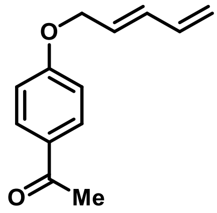

The title compound was synthesized from 1-(4-hydroxyphenyl)ethan-1-one (150 mg, 1.10 mmol) following **general procedure A**. The crude material was purified by column chromatography (petroleum ether/ ethyl acetate 20:1) to provide the desired product **8n** as white solids in 99 % yield (221 mg, 0.83 mmol); ^{1}H-NMR (400 MHz, $CDCl_3$): δ = 7.95 – 7.87 (m, 2H), 6.96 – 6.88 (m, 2H), 6.43 – 6.30 (m, 2H), 5.94 – 5.80 (m, 1H), 5.30 – 5.21 (m, 1H), 5.17 – 5.13 (m, 1H), 4.61 (d, *J* = 5.1 Hz, 2H), 2.53 (s, 3H); ^{13}C-NMR (101 MHz, $CDCl_3$): δ= 196.7, 162.5, 135.9, 134.3, 130.6, 130.5, 127.4, 118.8, 114.4, 68.3, 26.4; HRMS (ESI): exact mass calculated for $C_{13}H_{15}O_2^+$ [(M + H)$^+$], 203.1067; found 203.1074; m.p. 39-40 °C.

(*E*)-1-nitro-4-(penta-2,4-dien-1-yloxy)benzene (8o)

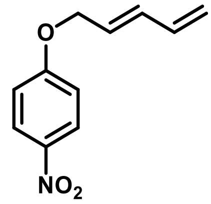

The title compound was synthesized from 4-nitrophenol (103 mg, 0.74 mmol) following **general procedure A**. The crude material was sufficiently pure, and no purification was necessary. The desired product **8o** was obtained as yellow solids in 93 % yield (142 mg, 0.69 mmol); ^{1}H-NMR (400 MHz, $CDCl_3$): δ = 8.24 – 8.16 (m, 2H), 7.01 – 6.92 (m, 2H), 6.46 – 6.31 (m, 2H), 5.97 – 5.81 (m, 1H), 5.41 – 5.24 (m, 1H), 5.24 – 5.14 (m, 1H), 4.67 (d, *J* = 5.3 Hz, 2H); ^{13}C-NMR (101 MHz, $CDCl_3$): δ= 163.7, 141.7, 135.8, 134.9, 126.7, 126.1, 119.3, 114.8, 68.9; HRMS (ESI): exact mass calculated for $C_{11}H_{10}NO_3^-$ [(M - H)$^-$], 204.0666; found 204.0664; m.p. 80-81 °C.

(*E*)-1-Iodo-4-(penta-2,4-dien-1-yloxy)benzene (8p)

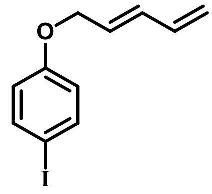

The title compound was synthesized from 4-iodophenol (355 mg, 1.61 mmol) following **general procedure A**. The crude material was purified by column chromatography (petroleum ether/ ethyl acetate 15:1) to provide the desired product **8p** as pale-yellow oil in 95 % yield (439 mg, 1.53 mmol); ^{1}H-NMR (400 MHz, $CDCl_3$): δ = 7.60 – 7.50 (m, 2H), 6.78 – 6.64 (m, 2H), 6.46 – 6.29 (m, 2H), 5.95 – 5.80 (m, 1H), 5.31 – 5.23 (m, 1H), 5.18 – 5.13 (m, 1H), 4.57 – 4.51 (m, 2H).^{13}C-NMR (151 MHz, $CDCl_3$): δ= 158.5, 138.3, 136.0, 134.1, 127.9, 118.7, 117.2, 83.1, 68.3; HRMS (ESI): exact mass calculated for $C_{11}H_{11}O^+$ [(M -I)$^+$], 159.0804; found 159.0816.

(E)-4-iodo-2-methoxy-1-(penta-2,4-dien-1-yloxy)benzene (8q)

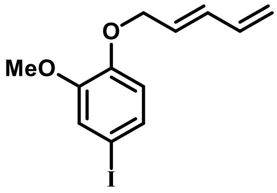

The title compound was synthesized from 4-iodo-2-methoxyphenol (216 mg, 0.86 mmol) following **general procedure A**. The crude material was purified by column chromatography (petroleum ether/ ethyl acetate 20:1) to provide the desired product **8q** as orange oil in 42 % yield (114 mg, 0.36 mmol); ^{1}H-NMR (400 MHz, $CDCl_3$): δ = 7.19 (dd, *J* = 8.4, 2.0 Hz, 1H), 7.13 (d, *J* = 2.0 Hz, 1H), 6.62 (d, *J* = 8.4 Hz, 1H), 6.41 – 6.26 (m, 2H), 5.97 – 5.83 (m, 1H), 5.35 – 5.19 (m, 1H), 5.19 – 5.08 (m, 1H), 4.60 (d, *J* = 5.4 Hz, 2H), 3.85 (s, 3H); ^{13}C-NMR (101 MHz, $CDCl_3$): δ= 150.4, 148.2, 136.1, 134.4, 129.8, 128.0, 120.9, 118.6, 115.4, 83.0, 69.3, 56.2.

Type 10 Aryl-dienyl ethers

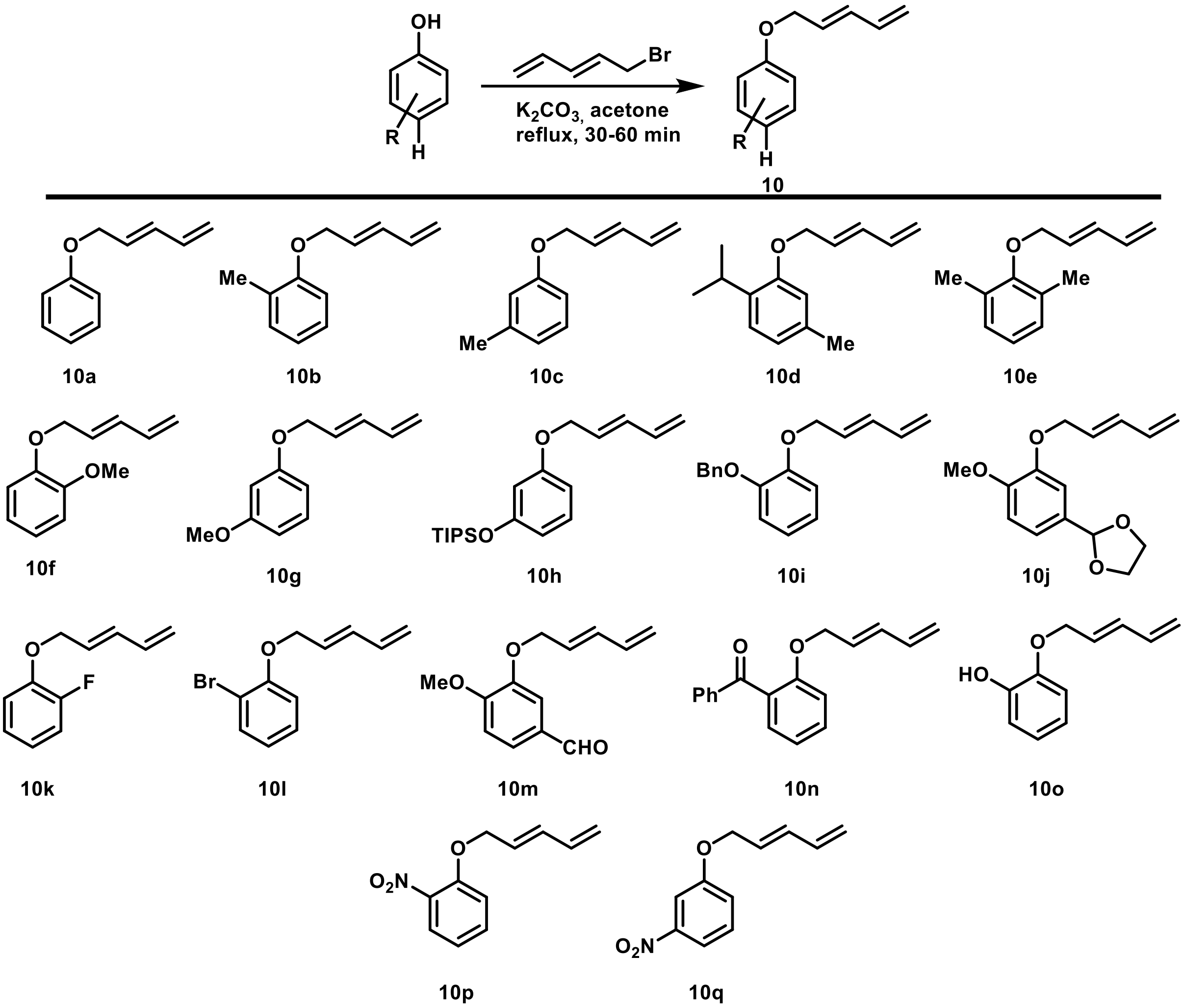

Scheme S2

(*E*)-(penta-2,4-dien-1-yloxy)benzene (10a)

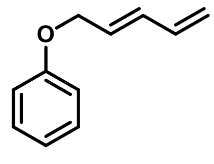

The title compound was synthesized from phenol (100 mg, 1.06 mmol) following **general procedure A**. The crude material was purified by column chromatography (petroleum ether/ ethyl acetate 10:1) to provide the desired product **10a** as colorless oil in 83 % yield (142 mg, 0.87 mmol); ^{1}H-NMR (400 MHz, $CDCl_3$): δ = 7.33 – 7.25 (m, 2H), 6.99 – 6.90 (m, 3H), 6.44 – 6.34 (m, 2H), 5.97 – 5.89 (m, 1H), 5.29 – 5.24 (m, 1H), 5.17 – 5.14 (m, 1H), 4.59 (d, *J* = 5.0 Hz, 2H); ^{13}C-NMR (101 MHz, $CDCl_3$): δ = 158.7, 136.2, 133.8, 129.6, 128.6, 121.0, 118.3, 114.9, 68.1; HRMS (ESI): exact mass calculated for $C_{11}H_{13}O^+$ [(M + H)$^+$], 161.0961; found 161.0961.

(*E*)-1-methyl-2-(penta-2,4-dien-1-yloxy)benzene (10b)

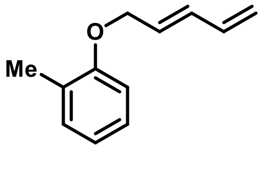

The title compound was synthesized from *o*-cresol (1.49 g, 13.78 mmol) following **general procedure A**. The crude material was purified by column chromatography (petroleum ether/ ethyl acetate 20:1) to provide the desired product **10b** as colorless oil in 68 % yield (1.62 g, 9.30 mmol); ^{1}H-NMR (400 MHz, $CDCl_3$): δ = 7.19 – 7.11 (m, 2H), 6.91 – 6.74 (m, 2H), 6.48 – 6.32 (m,

2H), 6.02 – 5.87 (m, 1H), 5.31 – 5.21 (m, 1H), 5.18 – 5.11 (m, 1H), 4.59 (d, *J* = 5.0 Hz, 2H), 2.27 (s, 3H); ^{13}C-NMR (101 MHz, $CDCl_3$): δ = 156.9, 136.4, 133.1, 130.9, 129.1, 127.2, 126.9, 120.6, 118.0, 111.5, 68.2, 16.4; HRMS (ESI): exact mass calculated for $C_{12}H_{15}O^+$ [(M + H)$^+$], 175.1117; found 175.1118.

(*E*)-1-methyl-3-(penta-2,4-dien-1-yloxy)benzene (10c)

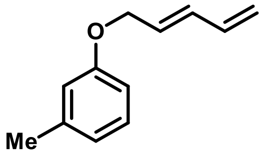

The title compound was synthesized from 3-methylphenol (208 mg, 1.92 mmol) following **general procedure A**. The crude material was purified by column chromatography (petroleum ether/ ethyl acetate 40:1) to provide the desired product **10c** as colorless oil in 62 % yield (208 mg, 1.19 mmol); ^{1}H-NMR (600 MHz, $CDCl_3$): δ = 7.17 (t, *J* = 7.8 Hz, 1H), 6.78 (d, *J* = 7.5 Hz, 1H), 6.76 (s, 1H), 6.73 (dd, *J* = 8.2, 2.7 Hz, 1H), 6.44 – 6.35 (m, 2H), 5.97 – 5.90 (m, 1H), 5.35 – 5.22 (m, 1H), 5.18 – 5.13 (m, 1H), 4.57 (d, *J* = 5.2 Hz, 2H), 2.34 (s, 3H).^{13}C-NMR (151 MHz, $CDCl_3$): δ = 158.7, 139.6, 136.3, 133.7, 129.3, 128.7, 121.8, 118.2, 115.7, 111.7, 68.1, 21.7; HRMS (ESI): exact mass calculated for $C_{12}H_{15}O^+$ [(M + H)$^+$], 175.1117; found 175.1118.

(*E*)-1-isopropyl-4-methyl-2-(penta-2,4-dien-1-yloxy)benzene (10d)

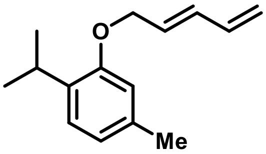

The title compound was synthesized from thymol (150 mg, 0.99 mmol) following **general procedure A**. The crude material was purified by column chromatography (petroleum ether/ ethyl acetate 20:1) to provide the desired product **10d** as pale-yellow oil in 65 % yield (224 mg, 0.65 mmol); ^{1}H-NMR (400 MHz, $CDCl_3$): δ = 7.14 (d, *J* = 7.7 Hz, 1H), 6.79 (dd, *J* = 7.7, 1.6 Hz, 1H), 6.69 (d, *J* = 1.7 Hz, 1H), 6.52 – 6.34 (m, 2H), 6.04 – 5.90 (m, 1H), 5.35 – 5.24 (m, 1H), 5.22 – 5.12 (m, 1H), 4.63 – 4.57 (m, 2H), 3.36 (hept, *J* = 6.9 Hz, 1H), 2.35 (s, 3H), 1.25 (d, *J* = 6.9 Hz, 6H); ^{13}C-NMR (101 MHz, $CDCl_3$): δ = 155.8, 136.4, 136.4, 134.5, 132.9, 129.3, 126.1, 121.5, 121.5, 117.9, 112.9, 68.3, 26.7, 22.9, 21.5; HRMS (ESI): exact mass calculated for $C_{15}H_{21}O^+$ [(M + H)$^+$], 217.1587; found 217.1597.

(*E*)-1,3-Dimethyl-2-(penta-2,4-dien-1-yloxy)benzene (10e)

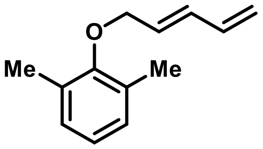

The title compound was synthesized from 2,6-dimethylphenol (400 mg, 3.27 mmol) following **general procedure A**. The crude material was purified by column chromatography (petroleum ether/ ethyl acetate 4:1) to provide the desired product **10e** as colorless oil in 49 % yield (304 mg, 1.61 mmol); ^{1}H-NMR (400 MHz, $CDCl_3$): δ = 7.06 – 7.00 (m, 2H), 6.94 (ddt, *J* = 8.1, 6.6, 1.3 Hz, 1H), 6.49 – 6.36 (m, 2H), 5.99 (dddd, *J* = 12.3, 8.3, 5.1, 1.4 Hz, 1H), 5.36 – 5.21 (m, 1H), 5.21 – 5.11 (m, 1H), 4.37 (d, *J* = 5.4 Hz, 2H), 2.30 (s, 6H); ^{13}C-NMR (101 MHz, $CDCl_3$): δ = 156.0, 136.4, 133.3, 131.1, 129.5, 128.9, 124.0, 124.0, 118.1, 72.4, 16.5; HRMS (ESI): exact mass calculated for $C_{13}H_{17}O^+$ [(M + H)$^+$], 189.1274; found 189.1289.

(*E*)-1-methoxy-2-(penta-2,4-dien-1-yloxy)benzene (10f)

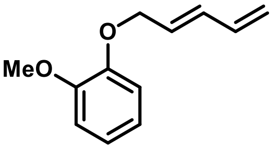

The title compound was synthesized from 2-methoxyphenol (210 mg, 1.69 mmol) following **general procedure A**. The crude material was purified by column chromatography (petroleum ether/ ethyl acetate 10:1) to provide the desired product **10f** as yellow oil in 94 % yield (301 mg, 1.58 mmol); ^{1}H-NMR (400 MHz, $CDCl_3$): δ = 6.96 – 6.88 (m, 4H), 6.44 – 6.27 (m, 2H), 6.03 – 5.88 (m, 1H), 5.26 – 5.21 (m, 1H), 5.15 – 5.10 (m, 1H), 4.65 (d, *J* = 5.4 Hz, 2H), 3.88 (s, 3H); ^{13}C-NMR (101 MHz, $CDCl_3$): δ = 149.6, 148.1, 136.3, 134.1, 128.7, 121.4, 120.9, 118.2, 113.7, 111.8, 69.3, 56.0; HRMS (ESI): exact mass calculated for $C_{12}H_{15}O_2^+$ [(M + H)$^+$], 191.1067; found 191.1075.

(*E*)-1-methoxy-3-(penta-2,4-dien-1-yloxy)benzene (10g)

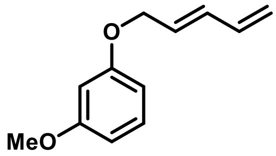

The title compound was synthesized from 3-methoxyphenol (150 mg, 1.21 mmol) following **general procedure A**. The crude material was purified by column chromatography (petroleum ether/ ethyl acetate 20:1) to provide the desired product **10g** as colorless oil in 71 % yield (163 mg, 0.86 mmol); ^{1}H-NMR (400 MHz, $CDCl_3$): δ = 7.19 (t, *J* = 8.2 Hz, 1H), 6.56 – 6.49 (m, 3H), 6.45 – 6.33 (m, 2H), 5.99 – 5.84 (m, 1H), 5.37 – 5.22 (m, 1H), 5.19 – 5.10 (m, 1H), 4.57 (d, *J* = 5.2 Hz, 2H), 3.80 (s, 3H); ^{13}C-NMR (101 MHz, $CDCl_3$): δ = 161.0, 160.0, 136.2, 133.9, 130.0, 128.4, 118.3, 107.0, 106.6, 101.4, 68.2, 55.4; HRMS (ESI): exact mass calculated for $C_{12}H_{15}O_2^+$ [(M + H)$^+$], 191.1067; found 191.1075.

(*E*)-triisopropyl(3-(penta-2,4-dien-1-yloxy)phenoxy)silane (10h)

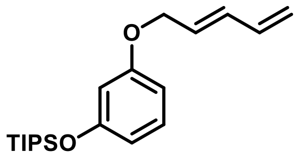

The title compound was synthesized from 3-((triisopropylsilyl)oxy)phenol[32] (200 mg, 0.75 mmol) following **general procedure A**. The crude material was purified by column chromatography (petroleum ether/ ethyl acetate 8:1) to provide the desired product **10h** as colorless oil in 96 % yield (240 mg,0.75 mmol); ^{1}H-NMR (400 MHz, $CDCl_3$): δ = 7.14 – 7.07 (m, 1H), 6.57 – 6.30 (m, 5H), 5.97 – 5.83 (m, 1H), 5.31 – 5.12 (m, 2H), 4.59 – 4.50 (m, 2H), 1.31 – 1.20 (m, 3H), 1.10 (d, *J* = 7.4 Hz, 18H); ^{13}C-NMR (101 MHz, $CDCl_3$): δ = 159.8, 157.3, 136.2, 133.8, 129.8, 128.6, 118.3, 112.8, 107.6, 107.1, 68.2, 18.1, 12.8; HRMS (ESI): exact mass calculated for $C_{20}H_{33}O_2Si^+$ [(M + H)$^+$], 333.2244; found 333.2257.

(*E*)-1-(benzyloxy)-2-(penta-2,4-dien-1-yloxy)benzene (10i)

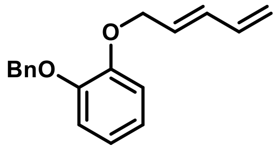

The title compound was synthesized from 2-(benzyloxy)phenol[34] (200 mg, 1.00 mmol) following **general procedure A**. The crude material was purified by column chromatography (petroleum ether/ ethyl acetate 20:1) to provide the desired product **10i** as colorless oil in 56 % yield (150 mg, 0.56 mmol); ^{1}H-NMR (400 MHz, $CDCl_3$): δ = 7.50 – 7.46 (m, 2H), 7.42 – 7.30 (m, 3H), 7.02 – 6.84 (m, 4H), 6.47 – 6.31 (m, 2H), 6.01 – 5.93 (m, 1H), 5.28 – 5.21 (m, 1H), 5.21 – 5.13 (m, 3H), 4.68 (d, *J* = 5.1 Hz, 2H); ^{13}C-NMR (101 MHz, $CDCl_3$): δ = 149.0, 149.0, 137.5, 136.3, 133.6, 128.9, 128.6, 127.8, 127.4, 127.3, 121.8, 121.7, 121.5, 118.1, 115.1, 114.8, 71.3, 69.5.; HRMS (ESI): exact mass calculated for $C_{18}H_{19}O_2^+$ [(M + H)$^+$], 267.1380; found 267.1392.

(*E*)-2-(4-methoxy-3-(penta-2,4-dien-1-yloxy)phenyl)-1,3-dioxolane (10j)

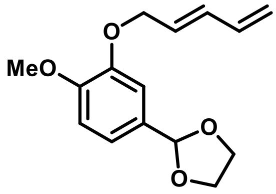

The title compound was synthesized from 5-(1,3-dioxolan-2-yl)-2-methoxyphenol[35] (200 mg, 1.02 mmol) following **general procedure A**. The crude material was purified by column chromatography (petroleum ether/ ethyl acetate 20:1) to provide the desired product **10j** as colorless oil in 68 % yield (183 mg, 0.70 mmol) which solidified in the freezer; ^{1}H-NMR (400 MHz, $CDCl_3$): δ = 7.05 – 7.01 (m, 2H), 6.87 (d, *J* = 8.0 Hz, 1H), 6.44 – 6.29 (m, 2H), 6.03 – 5.89 (m, 1H), 5.74 (s, 1H), 5.29 – 5.18 (m, 1H), 5.18 – 5.07 (m, 1H), 4.65 (d, *J* = 5.3 Hz, 2H), 4.14 – 4.09 (m, 2H), 4.04 – 3.99 (m, 2H), 3.87 (s, 3H); ^{13}C-NMR (101 MHz, $CDCl_3$): δ = 150.3, 148.1, 136.3, 134.3, 130.3, 128.4, 119.7, 118.3, 111.3, 111.3, 103.8, 69.3, 65.3, 65.3, 56.1; HRMS (ESI): exact mass calculated for $C_{15}H_{19}O_4^+$ [(M + H)$^+$], 263.1278; found 263.1289; m.p. 37-39 °C.

(*E*)-1-fluoro-2-(penta-2,4-dien-1-yloxy)benzene (10k)

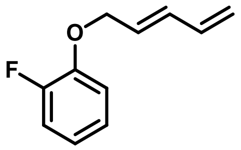

The title compound was synthesized from 2-fluorophenol (120 mg, 1.07 mmol) following **general procedure A**. The crude material was purified by column chromatography (petroleum ether/ ethyl acetate 20:1) to provide the desired product **10k** as colorless oil in 64 % yield (122 mg,0.68 mmol); ^{1}H-NMR (400 MHz, $CDCl_3$): δ = 7.13 – 6.87 (m, 4H), 6.44 – 6.32 (m, 2H), 6.01 – 5.85 (m, 1H), 5.32 – 5.21 (m, 1H), 5.21 – 5.10 (m, 1H), 4.65 (d, *J* = 5.4 Hz, 2H); ^{13}C-NMR (101 MHz, $CDCl_3$): δ = 154.2, 151.8, 146.8, 146.7, 136.1, 134.3, 128.0, 124.4, 124.4, 121.5, 121.5, 118.6, 116.5, 116.3, 115.6, 115.6, 69.7; ^{19}F-NMR (376 MHz, $CDCl_3$): δ = -134.3; HRMS (ESI): exact mass calculated for $C_{11}H_{12}FO^+$ [(M + H)$^+$], 179.0867; found 179.0868.

(*E*)-1-bromo-2-(penta-2,4-dien-1-yloxy)benzene (10l)

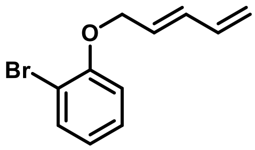

The title compound was synthesized from 2-bromophenol (150 mg, 0.87 mmol) following **general procedure A**. The crude material was purified by column chromatography (petroleum ether/ ethyl acetate 20:1) to provide the desired product **10l** as colorless oil in 90 % yield (186 mg, 0.78 mmol); ^{1}H-NMR (400 MHz, $CDCl_3$): δ = 7.55 (dd, *J* = 7.8, 1.6 Hz, 1H), 7.25 (ddd, *J* = 8.2, 7.4, 1.6 Hz, 1H), 6.90 (dd, *J* = 8.3, 1.4 Hz, 1H), 6.84 (td, *J* = 7.6, 1.4 Hz, 1H), 6.50 – 6.32 (m, 2H), 6.00 – 5.86 (m, 1H), 5.33 – 5.23 (m, 1H), 5.19 – 5.14 (m, 1H), 4.65 (dd, *J* = 5.5, 1.3 Hz, 2H); ^{13}C-NMR (101 MHz, $CDCl_3$): δ = 155.1, 136.2, 133.9, 133.6, 128.5, 127.9, 122.2, 118.5, 113.8, 112.5, 69.3; HRMS (ESI): exact mass calculated for $C_{11}H_{12}BrO^+$ [(M + H)$^+$], 239.0066; found 239.0066.

(*E*)-4-methoxy-3-(penta-2,4-dien-1-yloxy)benzaldehydebenzene (10m)

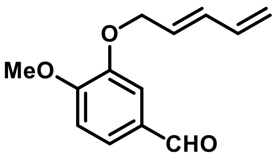

The title compound was synthesized from 3-hydroxy-4-methoxybenzaldehyde (160 mg, 1.01 mmol) following **general procedure A**. The crude material was purified by column chromatography (petroleum ether/ ethyl acetate 8:1) to provide the desired product **10m** as yellow solids oil in 97 % yield (220 mg,0.98 mmol); ^{1}H-NMR (400 MHz, $CDCl_3$): δ = 9.84 (s, 1H), 7.47 (dd, *J* = 8.2, 1.9 Hz, 1H), 7.41 (d, *J* = 1.9 Hz, 1H), 6.99 (d, *J* = 8.2 Hz, 1H), 6.45 – 6.28 (m, 2H), 6.03 – 5.88 (m, 1H), 5.33 – 5.21 (m, 1H), 5.21 – 5.09 (m, 1H), 4.69 (d, *J* = 5.5 Hz, 2H), 3.96 (s, 3H); ^{13}C-NMR (101 MHz, $CDCl_3$): δ = 190.9, 154.9, 148.6, 136.0, 134.7, 130.1, 127.5, 126.9, 118.7, 110.8, 110.7, 69.1, 56.2; HRMS (ESI): exact mass calculated for $C_{12}H_{13}O_2^+$ [(M -CHO)$^+$], 189.0910; found 189.0905; m.p. 55-57 °C.

(*E*)-(2-(penta-2,4-dien-1-yloxy)phenyl)(phenyl)methanone (10n)

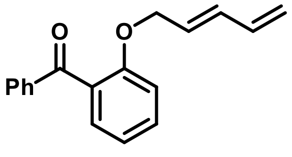

The title compound was synthesized from (2-hydroxyphenyl)(phenyl)methanone (230 mg, 1.16 mmol) following **general procedure A**. The crude material was purified by column chromatography (petroleum ether/ ethyl acetate 20:1) to provide the desired product **10n** as colorless oil in 65 % yield (197 mg, 0.75 mmol); ^{1}H-NMR (400 MHz, $CDCl_3$): δ = 7.86 – 7.76 (m, 2H), 7.59 – 7.50 (m, 1H), 7.50 – 7.37 (m, 4H), 7.07 (td, *J* = 7.5, 1.0 Hz, 1H), 7.00 – 6.95 (m, 1H), 6.21 (dt, *J* = 16.9, 10.3 Hz, 1H), 5.95 – 5.82 (m, 1H), 5.57 (dtd, *J* = 15.4, 5.2, 0.7 Hz, 1H), 5.09 – 5.01 (m, 2H), 4.50 (dd, *J* = 5.1, 1.6 Hz, 2H); ^{13}C-NMR (101 MHz, $CDCl_3$): δ = 196.7, 156.5, 138.3, 136.1, 132.9, 132.7, 132.2, 130.1, 129.8, 129.4, 128.3, 128.3, 127.6, 121.1, 118.0, 112.9, 68.3; HRMS (ESI): exact mass calculated for $C_{18}H_{17}O_2^+$ [(M + H)$^+$], 265.1223; found 265.1233.

(*E*)-2-(penta-2,4-dien-1-yloxy)phenol (10o)

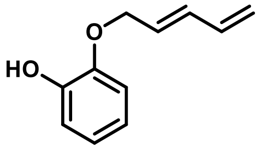

The title compound was synthesized from 2-hydroxyphenyl acetate (200 mg, 1.31 mmol) following **general procedure A**. The crude material was purified by column chromatography (petroleum ether/ ethyl acetate 20:1) to provide the desired product **10o** as colorless oil in 46 % yield (106 mg, 0.60 mmol); ^{1}H-NMR (400 MHz, $CDCl_3$): δ = 7.00 – 6.77 (m, 4H), 6.47 – 6.27 (m, 2H), 5.99 – 5.85 (m, 1H), 5.69 (s, 1H), 5.37 – 5.14 (m, 2H), 4.64 (d, *J* = 5.4 Hz, 1H); ^{13}C-NMR (101 MHz, $CDCl_3$): δ = 146.1, 145.7, 136.0, 134.5, 127.9, 121.9, 120.2, 118.9, 114.9, 112.3, 69.3; HRMS (ESI): exact mass calculated for $C_{11}H_{13}O_2^+$ [(M + H)$^+$], 177.0910; found 177.0913.

(*E*)-1-nitro-2-(penta-2,4-dien-1-yloxy)benzene (10p)

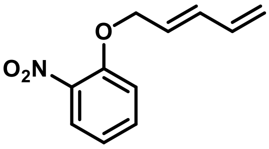

The title compound was synthesized from 2-nitrophenol (92 mg, 0.66 mmol) following **general procedure A**. The crude material was sufficiently pure, and no purification was necessary. The desired product **10p** was obtained as yellow oil in virtually quantitative yield (135 mg, 0.66 mmol); ^{1}H-NMR (400 MHz, $CDCl_3$): δ = 7.87 – 7.78 (m, 1H), 7.55 – 7.45 (m, 1H), 7.11 – 7.05 (m, 1H), 7.05 – 6.98 (m, 1H), 6.48 – 6.28 (m, 2H), 5.93 – 5.83 (m, 1H), 5.32 – 5.24 (m, 1H), 5.19 – 5.15 (m, 1H), 4.72 (d, *J* = 5.6 Hz, 2H); ^{13}C-NMR (101 MHz, $CDCl_3$): δ = 152.0, 135.9, 134.4, 134.1, 126.8, 125.8, 120.6, 119.0, 115.1, 115.1, 69.6; HRMS (ESI): exact mass calculated for $C_{11}H_{10}NO_3^-$ [(M - H)$^-$], 204.0666; found 204.0668.

(*E*)-1-nitro-3-(penta-2,4-dien-1-yloxy)benzene (10q)

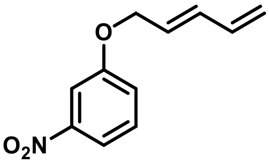

The title compound was synthesized from 3-nitrophenol (100 mg, 0.72 mmol) following **general procedure A**. The crude material was sufficiently pure, and no purification was necessary. The desired product **10q** was obtained as yellow oil in 92 % yield (135 mg, 0.66 mmol); ^{1}H-NMR (400 MHz, $CDCl_3$): δ = 7.83 (ddd, *J* = 8.1, 2.1, 1.0 Hz, 1H), 7.74 (t, *J* = 2.3 Hz, 1H), 7.43 (t, *J* = 8.2 Hz, 1H), 7.24 (ddd, *J* = 8.3, 2.6, 1.0 Hz, 1H), 6.46 – 6.33 (m, 2H), 5.97 – 5.82 (m, 1H), 5.32 – 5.25 (m, 1H), 5.21 – 5.17 (m, 1H), 4.66 (d, *J* = 5.2 Hz, 2H); ^{13}C-NMR (101 MHz, $CDCl_3$): δ = 159.2, 149.4, 135.9, 134.7, 130.1, 127.0, 122.0, 119.2, 116.1, 109.2, 68.8; HRMS (ESI): exact mass calculated for $C_{11}H_{10}NO_3^-$ [(M - H)$^-$], 204.0666; found 204.0669.

General procedure B - Rearrangement

An oven-dried screw vial was charged with starting material (1 equiv) dissolved in dry toluene (c= 0.2 M). The solution was degassed by ten vacuo/Ar cycles. Then, EuFOD (5 mol %) was added and the solution was degassed again ten times. The reaction vial was heated to 110 °C in a metal heating block until TLC confirmed full consumption of the starting material. The solvent was removed in vacuo and the crude material was purified by column chromatography.

Type 9 ortho rearranged branched dienes

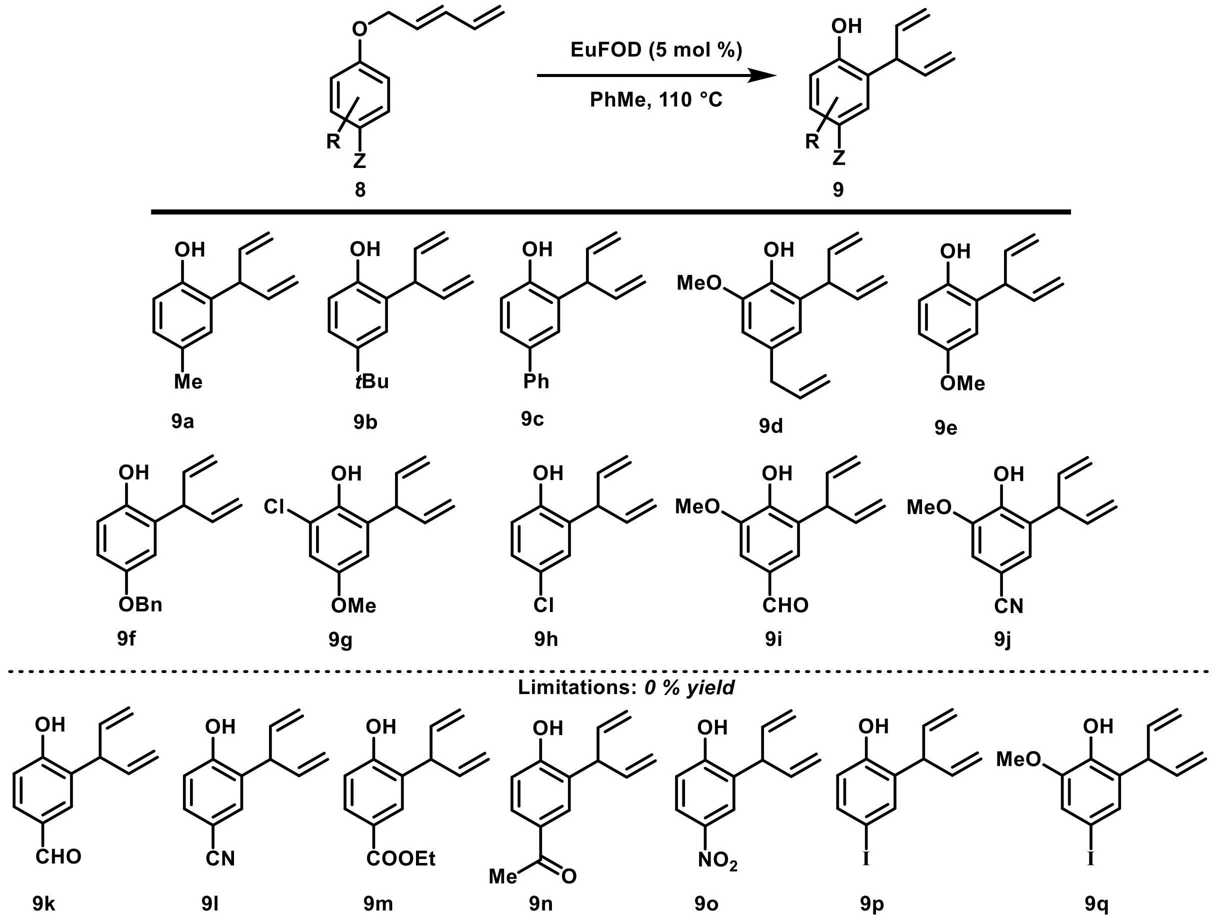

Scheme S3

Note: Structures **9k** to **9q** bearing a strong EWG *para* to the ether and phenol moiety respectively could not be prepared. Upon subjection of the corresponding ethers **8k** to **8q** to the standard rearrangement conditions, no reaction took place, and the starting materials were recovered unchanged. Switching from toluene to *o*-xylene and raising the temperature up to 140 °C did not lead to the desired product formation but decomposition of the starting material.

4-Methyl-2-(penta-1,4-dien-3-yl)phenol (9a)

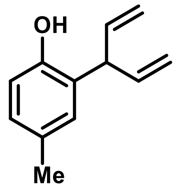

The title compound was synthesized from ether **8a** (98 mg, 0.56 mmol) following **general procedure B**. After column chromatography (petroleum ether/ ethyl acetate 15:1) the desired product **9a** was obtained as pale-yellow oil in 85 % yield (83 mg, 0.48 mmol); ^{1}H-NMR (400 MHz, $CDCl_3$): δ = 7.01 – 6.92 (m, 2H), 6.79 (dd, *J* = 8.5, 2.4 Hz, 1H), 6.13 (ddd, *J* = 14.5, 10.2, 6.2 Hz, 2H), 5.27 (*app*. dt, *J* = 10.3, 1.6 Hz, 2H), 5.22 – 5.11 (m, 3H), 4.34 – 4.25 (m, 1H), 2.29 (s, 3H); ^{13}C-NMR (101 MHz, $CDCl_3$): δ = 151.8, 138.7, 130.4, 130.0, 128.7, 127.1, 116.6, 116.5, 48.0, 20.7; HRMS (ESI): exact mass calculated for $C_{12}H_{15}O^{+}$ [$(M + H)^{+}$], 175.1117; found 175.1122.

4-(tert-butyl)-2-(penta-1,4-dien-3-yl)phenol (9b)

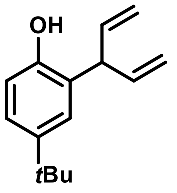

The title compound was synthesized from ether **8b** (113 mg, 0.52 mmol) following **general procedure B**. After column chromatography (petroleum ether/ ethyl acetate 15:1) the desired product **9b** was obtained as white solid in 79 % yield (89 mg, 0.41 mmol). ^{1}H-NMR (400 MHz, $CDCl_3$): δ = 7.19 (dd, *J* = 8.3, 2.5 Hz, 1H), 7.15 (d, *J* = 2.4 Hz, 1H), 6.86 (d, *J* = 8.5 Hz, 1H), 6.16 (ddd, *J* = 16.8, 10.2, 6.2 Hz, 2H), 5.36 (broad s, 1H), 5.27 (d, *J* = 10.2 Hz, 1H), 5.21 (d, *J* = 17.3 Hz, 1H), 4.37 – 4.27 (m, 1H), 1.30 (d, *J* = 0.9 Hz, 9H).^{13}C-NMR (101 MHz, $CDCl_3$): δ = 151.9, 143.9, 138.8, 126.6, 126.4, 125.1, 116.6, 116.3, 48.6, 31.7. HRMS (ESI): exact mass calculated for $C_{15}H_{21}O^+$ [(M + H)$^+$], 217.1587; found 217.1583; m.p. 36-37 °C.

3-(Penta-1,4-dien-3-yl)-[1,1'-biphenyl]-4-ol (9c)

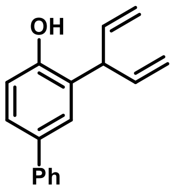

The title compound was synthesized from ether **8c** (100 mg, 0.42 mmol) following **general procedure B**. After column chromatography (petroleum ether/ ethyl acetate 20:1) the desired product **9c** was obtained as pale orange oil in 71 % yield (71 mg, 0.30 mmol); ^{1}H-NMR (400 MHz, $CDCl_3$): δ = 7.59 – 7.51 (m, 2H), 7.47 – 7.35 (m, 4H), 7.35 – 7.27 (m, 1H), 6.92 (d, *J* = 8.2 Hz, 1H), 6.16 (ddd, *J* = 17.3, 10.3, 6.2 Hz, 2H), 5.30 (*app.* dt, *J* = 10.3, 1.5 Hz, 2H), 5.26 – 5.19 (m, 3H), 4.41 – 4.31 (m, 1H); ^{13}C-NMR (101 MHz, $CDCl_3$): δ = 153.6, 141.0, 138.4, 134.4, 128.8, 128.3, 127.6, 127.0, 126.9, 126.8, 117.0, 116.9, 48.1; HRMS (ESI): exact mass calculated for $C_{17}H_{17}O^+$ [(M + H)$^+$], 237.1274; found 237.1287.

4-Allyl-2-methoxy-6-(penta-1,4-dien-3-yl)phenol (9d)

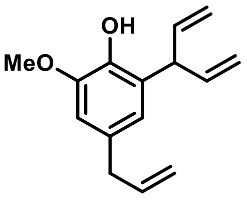

The title compound was synthesized from ether **8d** (100 mg, 0.43 mmol) following **general procedure B**. After column chromatography (petroleum ether/ ethyl acetate 20:1) the desired product **9d** was obtained as yellow oil in 81 % yield (81 mg, 0.35 mmol); ^{1}H-NMR (400 MHz, $CDCl_3$): δ = 6.76 (s, 1H), 6.65 (s, 1H), 6.17 – 5.93 (m, 3H), 5.19 – 5.05 (m, 7H), 4.56 – 4.47 (m, 1H), 4.02 (s, 3H), 3.37 (d, *J* = 6.7 Hz, 2H); ^{13}C-NMR (101 MHz, $CDCl_3$): δ = 147.1, 142.0, 139.5, 138.0, 131.5, 128.0, 121.0, 115.7, 115.4, 109.4, 56.6, 46.4, 40.2; HRMS (ESI): exact mass calculated for $C_{15}H_{18}NaO_2^+$ [(M + Na)$^+$], 253.1199; found 253.1209.

4-Methoxy-2-(penta-1,4-dien-3-yl)phenol (9e)

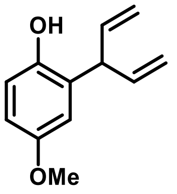

The title compound was synthesized from ether **8e** (105 mg, 0.55 mmol) following **general procedure B**. After column chromatography (petroleum ether/ ethyl acetate 10:1) the desired product **9e** was obtained as colorless oil in 59 % yield (62 mg, 0.33 mmol); ^{1}H-NMR (400 MHz, $CDCl_3$): δ 6.81 – 6.74 (m, 1H), 6.74 – 6.67 (m, 2H), 6.08 (ddd, *J* = 17.2, 10.3, 6.2 Hz, 2H), 5.25 (*app.* dt, *J* = 10.3, 1.5 Hz, 2H), 5.16 (*app.* dt, *J* = 17.2, 1.6 Hz, 2H), 4.89 – 4.78 (m, 1H), 4.30 – 4.23 (m, 1H), 3.76 (s, 3H).); ^{13}C-NMR (101 MHz, $CDCl_3$): δ = 154.0, 147.8, 138.4, 117.7, 117.3, 116.6, 115.1, 112.9, 55.8, 47.8; HRMS (ESI): exact mass calculated for $C_{12}H_{15}O_2^+$ [(M + H)$^+$], 191.1067; found 191.1063.

4-(Benzyloxy)-2-(penta-1,4-dien-3-yl)phenol (9f)

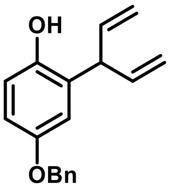

The title compound was synthesized from ether **8f** (100 mg, 0.38 mmol) following **general procedure B**. After column chromatography (petroleum ether/ ethyl acetate 20:1) the desired product **9f** was obtained as colorless oil in 48 % yield (48 mg, 0.18 mmol); ^{1}H-NMR (400 MHz, $CDCl_3$): δ = 7.47 – 7.28 (m, 5H), 6.82 – 6.77 (m, 3H), 6.08 (ddd, *J* = 17.3, 10.3, 6.3 Hz, 2H), 5.26 (*app.* dt, *J* = 10.2, 1.5 Hz, 2H), 5.17 (*app.* dt, *J* = 17.3, 1.6 Hz, 2H), 5.00 (s, 2H), 4.80 (s, 1H), 4.30 – 4.24 (m, 1H); ^{13}C-NMR (101 MHz, $CDCl_3$): δ = 153.2, 148.0, 138.4, 137.4, 128.7, 128.7, 128.0, 127.7, 117.3, 116.7, 116.3, 114.0, 70.8, 47.8; HRMS (ESI): exact mass calculated for $C_{18}H_{19}O_2^+$ [$(M + H)^+$], 267.1380; found 267.1388.

2-Chloro-4-methoxy-6-(penta-1,4-dien-3-yl)phenol (9g)

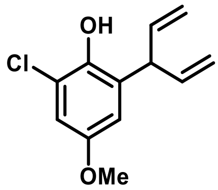

The title compound was synthesized from ether **8g** (77 mg, 0.32 mmol) following **general procedure B**. After column chromatography (petroleum ether/ ethyl acetate 20:1) the desired product **9g** was obtained as pale-green oil in 75 % yield (58 mg, 0.26 mmol); ^{1}H-NMR (400 MHz, $CDCl_3$): δ = 6.78 (d, *J* = 3.0 Hz, 1H), 6.65 (d, *J* = 3.0 Hz, 1H), 6.02 (ddd, *J* = 17.2, 10.3, 6.4 Hz, 2H), 5.29 (s, 1H), 5.18 (*app.* dt, *J* = 10.3, 1.5 Hz, 2H), 5.11 (*app.* dt, *J* = 17.2, 1.6 Hz, 2H), 4.48 – 4.41 (m, 1H), 3.74 (s, 3H); ^{13}C-NMR (101 MHz, $CDCl_3$): δ = 153.3, 143.2, 138.6, 130.8, 120.3, 116.2, 114.5, 112.1, 56.0, 46.8; HRMS (ESI): exact mass calculated for $C_{12}H_{14}ClO_2^+$ [$(M + H)^+$], 225.0677; found 225.0667.

4-Chloro-2-(penta-1,4-dien-3-yl)phenol (9h)

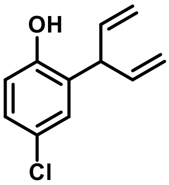

The title compound was synthesized from ether **8h** (600 mg, 3.08 mmol) following **general procedure B**. After column chromatography (petroleum ether/ ethyl acetate 20:1) the desired product **9h** was obtained as yellow oil in 67 % yield (400 mg, 2.05 mmol); ^{1}H-NMR (400 MHz, $CDCl_3$): δ = 7.15 – 7.07 (m, 2H), 6.81 – 6.73 (m, 1H), 6.06 (ddd, *J* = 17.2, 10.3, 6.2 Hz, 2H), 5.28 (*app.* dt, *J* = 10.3, 1.4 Hz, 2H), 5.17 (*app.* dt, *J* = 17.3, 1.5 Hz, 2H), 5.10 (s, 1H), 4.29 – 4.20 (m, 1H); ^{13}C-NMR (101 MHz, $CDCl_3$): δ = 152.6, 137.8, 129.3, 129.2, 128.1, 125.9, 117.9, 117.3, 47.6; HRMS (ESI): exact mass calculated for $C_{11}H_{10}ClO^-$ [$(M - H)^-$], 193.0426; found 193.0422.

4-Hydroxy-3-methoxy-5-(penta-1,4-dien-3-yl)benzaldehyde (9i)

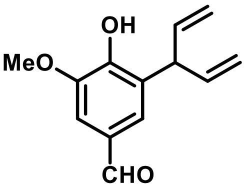

The title compound was synthesized from ether **8i** (99 mg, 0.45 mmol) following **general procedure B**. After column chromatography (petroleum ether/ ethyl acetate 10:1) the desired product **9i** was obtained as pale-yellow solids in 63 % yield (62 mg, 0.28 mmol); ^{1}H-NMR (400 MHz, $CDCl_3$): δ = 9.81 (s, 1H), 7.32 (s, 2H), 6.39 (d, *J* = 1.0 Hz, 1H), 6.05 (ddd, *J* = 17.0, 10.3, 6.4 Hz, 2H), 5.18 (*app.* dt, *J* = 10.3, 1.5 Hz, 2H), 5.11 (*app.* dt, *J* = 17.2, 1.6 Hz, 2H), 4.56 – 4.50 (m, 1H), 3.95 (s, 3H).; ^{13}C-NMR (101 MHz, $CDCl_3$): δ = 191.2, 149.2, 147.2, 138.5, 129.2, 128.4, 127.0, 116.2, 107.1, 56.4, 45.6; HRMS (ESI): exact mass calculated for $C_{13}H_{15}O_3^+$ [$(M + H)^+$], 219.1016; found 219.0994; m.p. 75-76 °C.

4-Hydroxy-3-methoxy-5-(penta-1,4-dien-3-yl)benzonitrile(9j)

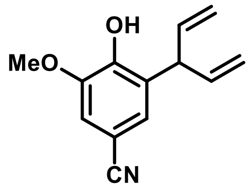

The title compound was synthesized from ether **8j** (104 mg, 0.48 mmol) following **general procedure B**. After column chromatography (petroleum ether/ ethyl acetate 15:1) the desired product **9j** was obtained as white solids in 89 % yield (93 mg, 0.43 mmol); ^{1}H-NMR (400 MHz, CDCl$_3$): δ = 7.13 (d, *J* = 1.9 Hz, 1H), 6.99 (d, *J* = 1.9 Hz, 1H), 6.24 (broad s, *J* = 1.2 Hz, 1H), 5.98 (ddd, *J* = 16.9, 10.3, 6.4 Hz, 2H), 5.18 (*app.* dt, *J* = 10.3, 1.4 Hz, 2H), 5.09 (*app.* dt, *J* = 17.2, 1.5 Hz, 2H), 4.50 – 4.45 (m, 1H), 3.92 (s, 3H); ^{13}C-NMR (101 MHz, CDCl$_3$): δ = 147.4, 146.6, 138.0, 129.5, 126.6, 119.5, 116.4, 112.0, 102.9, 56.5, 45.3; HRMS (ESI): exact mass calculated for $C_{13}H_{14}NO_2^+$ [(M + H)$^+$], 216.1019; found 216.1023; m.p. 83-84 °C.

Type 11 para rearranged linear conjugated dienes

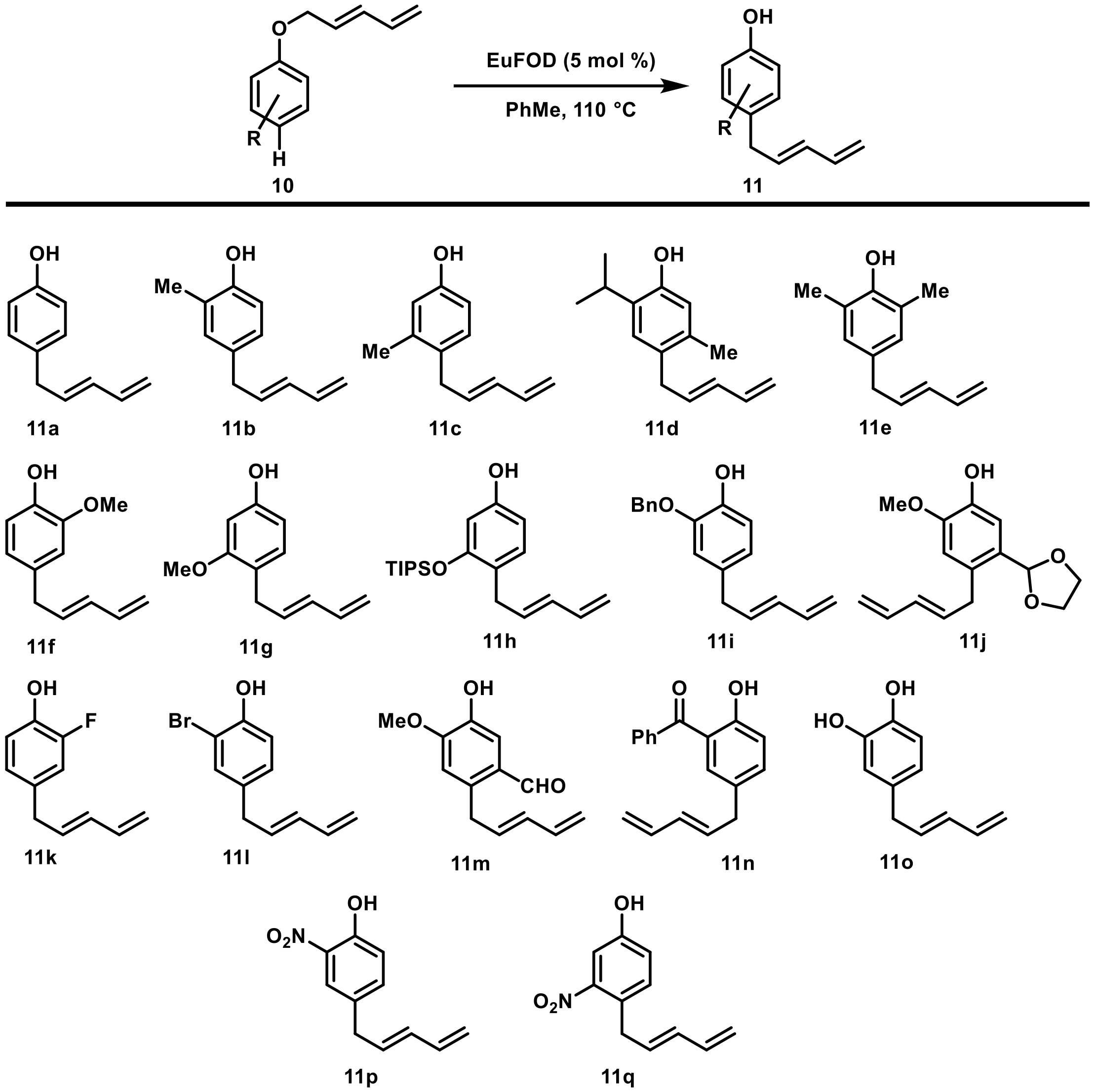

Scheme S4

(*E*)-4-(penta-2,4-dien-1-yl)phenol (11a)

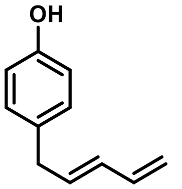

The title compound was synthesized from ether **10a** (77 mg, 0.48 mmol) following **general procedure B**. After column chromatography (petroleum ether/ ethyl acetate 10:1) the desired product **11a** was obtained as colorless oil in 46 % yield (35 mg, 0.22 mmol); ^{1}H-NMR (400 MHz, $CDCl_3$): δ = 7.06 (d, *J* = 8.4 Hz, 2H), 6.78 (d, *J* = 8.6 Hz, 2H), 6.41 – 6.28 (m, 1H), 6.17 – 6.04 (m, 1H), 5.89 – 5.77 (m, 1H), 5.14 (dd, *J* = 16.6, 1.4 Hz, 1H), 5.01 (dd, *J* = 10.1, 1.8 Hz, 1H), 3.36 (d, *J* = 6.3 Hz, 2H) ;^{13}C-NMR (101 MHz, $CDCl_3$): δ = 154.0, 137.1, 134.0, 132.4, 131.9, 129.9, 115.8, 115.4, 38.1; HRMS (ESI): exact mass calculated for $C_{11}H_{13}O^+$ [(M + H)$^+$], 161.0961; found 161.0959.

(*E*)-2-methyl-4-(penta-2,4-dien-1-yl)phenol (11b)

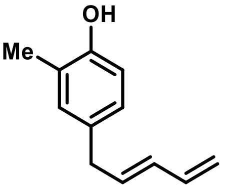

The title compound was synthesized from ether **10b** (85 mg, 0.49 mmol) following **general procedure B**. After column chromatography (petroleum ether/ ethyl acetate 12:1) the desired product **11b** was obtained as colorless oil in 96 % yield (82 mg, 0.47 mmol); ^{1}H-NMR (400 MHz, $CDCl_3$): δ = 6.94 (d, *J* = 2.2 Hz, 1H), 6.90 (dd, *J* = 8.0, 2.3 Hz, 1H), 6.72 (d, *J* = 8.0 Hz, 1H), 6.45 – 6.27 (m, 1H), 6.17 – 6.04 (m, 1H), 5.88 – 5.77 (m, 1H), 5.14 (d, *J* = 17.8 Hz, 1H), 5.01 (d, *J* = 10.2 Hz, 1H), 4.73 (s, 1H), 3.33 (d, *J* = 6.6 Hz, 2H), 2.24 (s, 3H); ^{13}C-NMR (101 MHz, $CDCl_3$): δ = 152.3, 137.2, 134.2, 132.3, 131.8, 131.3, 127.2, 123.8, 115.7, 115.0, 38.2, 15.9; HRMS (ESI): exact mass calculated for $C_{12}H_{15}O^+$ [(M + H)$^+$], 175.1117; found 175.1111.

(*E*)-3-methyl-4-(penta-2,4-dien-1-yl)phenol (11c)

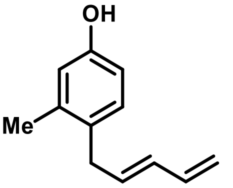

The title compound was synthesized from ether **10c** (115 mg, 0.66 mmol) following **general procedure B**. After column chromatography (petroleum ether/ ethyl acetate 8:1) the desired product **11c** was obtained as colorless oil in 87 % yield (100 mg, 0.57 mmol); ^{1}H-NMR (400 MHz, $CDCl_3$): δ = 7.01 (d, *J* = 8.0 Hz, 1H), 6.76 – 6.62 (m, 2H), 6.41 – 6.27 (m, 1H), 6.06 – 5.95 (m, 1H), 5.88 – 5.75 (m, 1H), 5.17 – 5.04 (m, 2H), 4.99 (d, *J* = 10.1 Hz, 1H), 3.35 (d, *J* = 7.8 Hz, 2H), 2.25 (s, 3H); ^{13}C-NMR (101 MHz, $CDCl_3$): δ = 154.1, 138.1, 137.1, 133.4, 131.8, 130.6, 130.5, 117.2, 115.5, 112.9, 35.7, 19.6; HRMS (ESI): exact mass calculated for $C_{12}H_{15}O^+$ [(M + H)$^+$], 175.1117; found 175.1115.

(*E*)-2-isopropyl-5-methyl-4-(penta-2,4-dien-1-yl)phenol (11d)

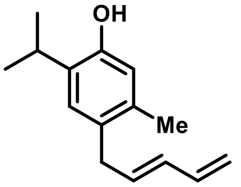

The title compound was synthesized from ether **10d** (100 mg, 0.46 mmol) following **general procedure B**. After column chromatography (petroleum ether/ ethyl acetate 20:1) the desired product **11d** was obtained as colorless oil in 80 % yield (80 mg, 0.37 mmol); ^{1}H-NMR (400 MHz, $CDCl_3$): δ = 6.95 (s, 1H), 6.60 (s, 1H), 6.42 – 6.28 (m, 1H), 6.09 – 5.97 (m, 1H), 5.87 – 5.76 (m, 1H), 5.11 (dd, *J* = 17.0, 1.8 Hz, 1H), 4.99 (dd, *J* = 10.2, 1.8 Hz, 1H), 4.75 (s, 1H), 3.35 (d, *J* = 6.6 Hz, 2H), 3.19 (hept, *J* = 6.9 Hz, 1H), 2.21 (s, 3H), 1.27 (d, *J* = 6.9 Hz, 6H); ^{13}C-NMR (101 MHz, $CDCl_3$): δ = 151.2, 137.2, 134.9, 133.7, 131.9, 131.6, 130.5, 127.5, 117.3, 115.4, 36.2, 27.1, 22.8, 19.0; HRMS (ESI): exact mass calculated for $C_{15}H_{21}O^+$ [(M + H)$^+$], 217.1587; found 217.1591.

(*E*)-2,6-Dimethyl-4-(penta-2,4-dien-1-yl)phenol (11e)

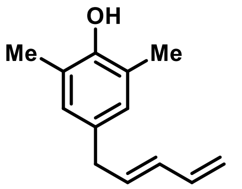

The title compound was synthesized from ether **10e** (100 mg, 0.53 mmol) following **general procedure B**. After column chromatography (petroleum ether/ ethyl acetate 10:1) the desired product **11e** was obtained as yellow solids in 97 % yield (97 mg, 0.52 mmol); ^{1}H-NMR (400 MHz, CDCl$_3$): δ = 6.82 (s, 2H), 6.36 (dt, *J* = 17.0, 10.3 Hz, 1H), 6.17 – 6.05 (m, 1H), 5.89 – 5.78 (m, 1H), 5.15 (dd, *J* = 17.0, 1.9 Hz, 1H), 5.02 (dd, *J* = 10.1, 1.9 Hz, 1H), 4.73 (s, 1H), 3.31 (dd, *J* = 7.1, 1.6 Hz, 2H), 2.27 (s, 6H); ^{13}C-NMR (101 MHz, CDCl$_3$): δ = HRMS (ESI): exact mass calculated for $C_{13}H_{17}O^+$ [(M + H)$^+$], 189.1274; found 189.1288; m.p. 54-56 °C.

(*E*)-2-methoxy-4-(penta-2,4-dien-1-yl)phenol (11f)

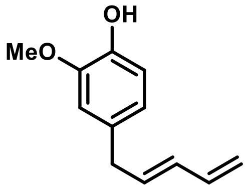

The title compound was synthesized from ether **10f** (120 mg, 0.63 mmol) following **general procedure B**. After column chromatography (petroleum ether/ ethyl acetate 8:1) the desired product **11f** was obtained as colorless oil in 91 % yield (109 mg, 0.57 mmol); ^{1}H-NMR (400 MHz, CDCl$_3$): δ = 6.88 (d, *J* = 7.9 Hz, 1H), 6.73 – 6.67 (m, 2H), 6.43 – 6.29 (m, 1H), 6.11 (dd, *J* = 15.1, 10.4 Hz, 1H), 5.90 – 5.80 (m, 1H), 5.60 (broad s, 1H), 5.15 (dd, *J* = 16.8, 1.7 Hz, 1H), 5.02 (dd, *J* = 10.2, 1.7 Hz, 1H), 3.90 (s, 3H), 3.37 (d, *J* = 6.9 Hz, 2H); ^{13}C-NMR (101 MHz, CDCl$_3$): δ = 146.7, 144.2, 137.1, 134.0, 132.1, 131.9, 121.4, 115.8, 114.4, 111.3, 56.1, 38.7; HRMS (ESI): exact mass calculated for $C_{12}H_{15}O_2^+$ [(M + H)$^+$], 191.1067; found 191.1058.

(*E*)-3-methoxy-4-(penta-2,4-dien-1-yl)phenol (11g)

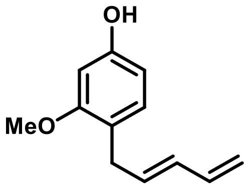

The title compound was synthesized from ether **10g** (100 mg, 0.53 mmol) following **general procedure B**. After column chromatography (petroleum ether/ ethyl acetate 20:1) the desired product **11g** was obtained as orange oil in % 64 yield (64 mg, 0.33 mmol); ^{1}H-NMR (400 MHz, CDCl$_3$): δ = 6.95 (d, *J* = 8.0 Hz, 1H), 6.41 (d, *J* = 2.4 Hz, 1H), 6.39 – 6.28 (m, 2H), 6.12 – 6.00 (m, 1H), 5.84 (dt, *J* = 15.1, 6.8 Hz, 1H), 5.09 (dd, *J* = 17.0, 1.8 Hz, 1H), 4.97 (dd, *J* = 10.1, 1.8 Hz, 1H), 4.81 (s, 1H), 3.79 (s, 4H), 3.33 (d, *J* = 6.4 Hz, 2H); ^{13}C-NMR (101 MHz, CDCl$_3$): δ = 158.4, 155.2, 137.4, 133.7, 131.6, 130.4, 121.0, 115.2, 106.8, 99.0, 55.5, 32.4; HRMS (ESI): exact mass calculated for $C_{12}H_{14}NaO_2^+$ [(M + Na)$^+$], 213.0886; found 213.0913.

(*E*)-4-(penta-2,4-dien-1-yl)-3-((triisopropylsilyl)oxy)phenol (11h)

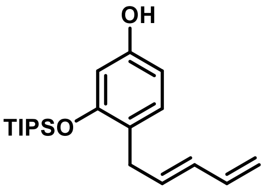

The title compound was synthesized from ether **10h** (130 mg, 0.39 mmol) following **general procedure B**. After column chromatography (petroleum ether/ ethyl acetate 20:1) the desired product **11h** was obtained as brown oil in 52 % yield (68 mg, 0.20 mmol); ^{1}H-NMR (400 MHz, CDCl$_3$): δ = 6.94 (d, *J* = 8.6 Hz, 1H), 6.41 – 6.27 (m, 3H), 6.11 – 5.99 (m, 1H), 5.89 – 5.77 (m, 1H), 5.07 (dd, *J* = 17.0, 1.9 Hz, 1H), 4.96 (dd, *J* = 10.2, 1.8 Hz, 1H), 4.59 (broad s, 1H), 3.35 (d, *J* = 6.7 Hz, 2H), 1.36 – 1.25 (m, 3H), 1.11 (d, *J* = 7.4 Hz, 18H); ^{13}C-NMR (101 MHz, CDCl$_3$): δ = 154.8, 137.3, 133.8, 131.7, 130.6, 122.9, 115.1, 107.7, 105.8, 32.8, 18.2, 13.2; HRMS (ESI): exact mass calculated for $C_{20}H_{33}O_2Si^+$ [(M + H)$^+$], 333.2244; found 333.2245.

(*E*)-2-(benzyloxy)-4-(penta-2,4-dien-1-yl)phenol (11i)

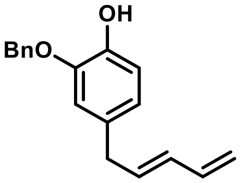

The title compound was synthesized from ether **10i** (100 mg, 0.38 mmol) following **general procedure B**. After column chromatography (petroleum ether/ ethyl acetate 20:1) the desired product **11i** was obtained as yellow oil in 72 % yield (72 mg, 0.27mmol); ^{1}H-NMR (400 MHz, $CDCl_3$): δ = 7.50 – 7.33 (m, 5H), 6.88 (d, *J* = 8.1 Hz, 1H), 6.78 (d, *J* = 1.9 Hz, 1H), 6.72 (dd, *J* = 8.1, 1.9 Hz, 1H), 6.35 (dt, *J* = 17.0, 10.2 Hz, 1H), 6.10 (dd, *J* = 15.1, 10.3 Hz, 1H), 5.83 (dt, *J* = 14.6, 6.9 Hz, 1H), 5.55 (s, 1H), 5.15 (dd, *J* = 17.1, 1.9 Hz, 1H), 5.09 (s, 2H), 5.05 – 5.00 (m, 1H), 3.35 (dd, *J* = 7.0, 1.4 Hz, 2H); ^{13}C-NMR (101 MHz, $CDCl_3$): δ = 145.8, 144.4, 137.1, 136.5, 133.9, 132.0, 132.0, 128.8, 128.5, 128.1, 121.8, 115.8, 114.7, 112.8, 71.3, 38.7; HRMS (ESI): exact mass calculated for $C_{18}H_{19}O_2^+$ [(M + H)$^+$], 267.1380; found 267.1391.

(*E*)-5-(1,3-dioxolan-2-yl)-2-methoxy-4-(penta-2,4-dien-1-yl)phenol (11j)

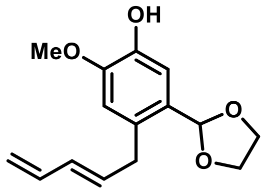

The title compound was synthesized from ether **10j** (100 mg, 0.38 mmol) following **general procedure B**. After column chromatography (petroleum ether/ ethyl acetate 20:1) the desired product **11j** was obtained as colorless oil in 74 % yield (74 mg, 0.28mmol); ^{1}H-NMR (400 MHz, $CDCl_3$): δ = 7.14 (s, 1H), 6.64 (s, 1H), 6.33 (dt, *J* = 17.0, 10.2 Hz, 1H), 6.11 – 5.98 (m, 1H), 5.89 (s, 1H), 5.89 – 5.79 (m, 1H), 5.51 (s, 1H), 5.11 (dd, *J* = 17.2, 1.9 Hz, 1H), 4.99 (dd, *J* = 10.1, 1.8 Hz, 1H), 4.15 – 4.07 (m, 2H), 4.05 – 3.97 (m, 2H), 3.87 (s, 3H), 3.49 (d, *J* = 4.9 Hz, 2H); ^{13}C-NMR (101 MHz, $CDCl_3$): δ = 147.1, 144.0, 137.1, 133.8, 131.9, 130.5, 128.4, 115.7, 112.8, 112.4, 101.5, 65.3, 56.1, 35.0; HRMS (ESI): exact mass calculated for $C_{15}H_{19}O_4^+$ [(M + H)$^+$], 263.1278; found 263.1290.

(*E*)-2-fluoro-4-(penta-2,4-dien-1-yl)phenol (11k)

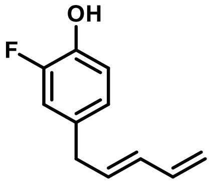

The title compound was synthesized from ether **10k** (100 mg, 0.56 mmol) following **general procedure B**. After column chromatography (petroleum ether/ ethyl acetate 8:1) the desired product **11k** was obtained as pale-yellow oil in 81 % yield (81 mg, 0.45 mmol); ^{1}H-NMR (400 MHz, $CDCl_3$): δ = 6.97 – 6.87 (m, 2H), 6.84 (dd, *J* = 8.4, 2.1 Hz, 1H), 6.34 (dt, *J* = 17.0, 10.3 Hz, 1H), 6.09 (dd, *J* = 15.2, 10.4 Hz, 1H), 5.80 (dt, *J* = 14.6, 6.9 Hz, 1H), 5.15 (dd, *J* = 17.1, 1.7 Hz, 1H), 5.11 (s, 1H), 5.03 (dd, *J* = 9.6, 1.4 Hz, 1H), 3.34 (d, *J* = 6.9 Hz, 2H); ^{13}C-NMR (101 MHz, $CDCl_3$): δ = 152.2, 149.8, 141.9, 141.7, 136.9, 133.2, 133.2, 133.1, 132.4, 124.9, 124.9, 117.2, 117.2, 116.2, 115.8, 115.6, 38.1, 38.0; ^{19}F-NMR (376 MHz, $CDCl_3$): δ = -141.1; HRMS (ESI): exact mass calculated for $C_{11}H_{12}FO^+$ [(M + H)$^+$], 179.0867; found 179.0861.

(*E*)-2-bromo-4-(penta-2,4-dien-1-yl)phenol (11l)

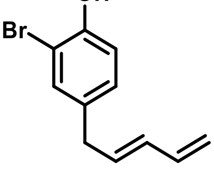

The title compound was synthesized from ether **10l** (100 mg, 0.42 mmol) following **general procedure B**. After column chromatography (petroleum ether/ ethyl acetate 20:1) the desired product **11l** was obtained as colorless oil in 86 % yield (86 mg, 0.36 mmol); ^{1}H-NMR (400 MHz, $CDCl_3$): δ = 7.28 (d, *J* = 2.1 Hz, 1H), 7.03 (dd, *J* = 8.3, 2.1 Hz, 1H), 6.95 (d, *J* = 8.3 Hz, 1H), 6.34 (dt, *J* = 17.0, 10.3 Hz, 1H), 6.15 – 6.03 (m, 1H), 5.79 (dt, *J* = 14.6, 6.9 Hz, 1H), 5.42 (s, 1H), 5.16 (dd, *J* = 17.0, 1.9 Hz, 1H),

5.04 (dd, J = 10.2, 1.9 Hz, 1H), 3.34 (dd, J = 7.0, 1.4 Hz, 2H); ^{13}C-NMR (101 MHz, $CDCl_3$): δ = 150.7, 136.9, 133.9, 133.0, 132.5, 131.9, 129.5, 116.2, 116.1, 110.2, 37.8; HRMS (ESI): exact mass calculated for $C_{11}H_{10}BrO^-$ [(M - H)$^-$], 236.9921; found 236.9916.

(*E*)-5-hydroxy-4-methoxy-2-(penta-2,4-dien-1-yl)benzaldehyde (11m)

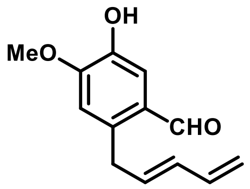

The title compound was synthesized from ether **10m** (103 mg, 0.47 mmol) following **general procedure B**. After column chromatography (ether) the desired product **11m** was obtained as orange solids in 81 % yield (83 mg, 0.38 mmol); ^{1}H-NMR (400 MHz, $CDCl_3$): δ = 10.12 (s, 1H), 7.45 (s, 1H), 6.71 (s, 1H), 6.38 – 6.24 (m, 1H), 6.07 – 5.96 (m, 1H), 5.93 – 5.82 (m, 1H), 5.77 (s, 2H), 5.10 (d, J = 17.0 Hz, 1H), 5.00 (d, J = 10.1 Hz, 1H), 3.97 (s, 2H), 3.80 (d, J = 6.3 Hz, 2H); ^{13}C-NMR (101 MHz, $CDCl_3$): δ = 190.9, 151.4, 144.5, 136.8, 136.6, 133.2, 132.4, 127.5, 116.7, 116.4, 112.7, 56.3, 35.0; HRMS (ESI): exact mass calculated for $C_{13}H_{15}O_3^+$ [(M + H)$^+$], 219.1016; found 216.1038; m.p. 60-62 °C.

(*E*)-(2-hydroxy-5-(penta-2,4-dien-1-yl)phenyl)(phenyl)methanone (11n)

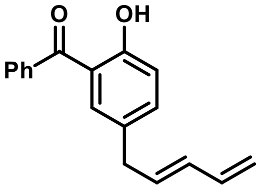

The title compound was synthesized from ether **10n** (100 mg, 0.38 mmol) following **general procedure B**. After column chromatography (petroleum ether/ ethyl acetate 20:1) the desired product **11n** was obtained as pale-yellow oil in 79 % yield (79 mg, 0.30 mmol); ^{1}H-NMR (400 MHz, $CDCl_3$): δ = 11.93 (s, 1H), 7.72 – 7.64 (m, 2H), 7.64 – 7.56 (m, 1H), 7.55 – 7.46 (m, 2H), 7.40 – 7.33 (m, 2H), 7.03 (d, J = 8.5 Hz, 1H), 6.31 (dt, J = 17.0, 10.2 Hz, 1H), 6.12 – 5.99 (m, 1H), 5.77 (dt, J = 14.5, 6.8 Hz, 1H), 5.12 (dd, J = 17.0, 1.7 Hz, 1H), 5.01 (dd, J = 10.2, 1.7 Hz, 1H), 3.32 (d, J = 7.2 Hz, 2H); ^{13}C-NMR (101 MHz, $CDCl_3$): δ = 201.6, 161.9, 138.1, 137.0, 136.8, 133.2, 133.1, 132.4, 132.1, 130.3, 129.4, 128.5, 119.0, 118.6, 116.2, 37.9; HRMS (ESI): exact mass calculated for $C_{18}H_{17}O_2^+$ [(M + H)$^+$], 265.1223; found 265.1206.

(*E*)-4-(penta-2,4-dien-1-yl)benzene-1,2-diol (11o)

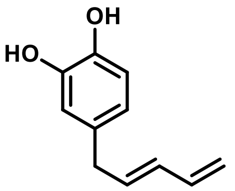

The title compound was synthesized from ether **10o** (106 mg, 0.49 mmol) following **general procedure B**. After column chromatography (petroleum ether/ ethyl acetate 10:1 to 5:1) the desired product **11o** was obtained as colorless oil in 60 % yield (64 mg, 0.36 mmol); ^{1}H-NMR (400 MHz, $CDCl_3$): δ = 6.79 (d, J = 8.0 Hz, 1H), 6.70 (d, J = 2.0 Hz, 1H), 6.62 (dd, J = 8.1, 2.0 Hz, 1H), 6.41 – 6.25 (m, 1H), 6.14 – 6.02 (m, 1H), 5.86 – 5.76 (m, 1H), 5.18 – 5.11 (m, 1H), 5.04 – 4.99 (m, 1H), 3.31 (d, J = 6.9 Hz, 2H); ^{13}C-NMR (101 MHz, $CDCl_3$): δ = 143.6, 141.8, 137.1, 133.8, 133.4, 132.0, 121.2, 115.9, 115.8, 115.6, 38.3; HRMS (ESI): exact mass calculated for $C_{11}H_{13}O_2^+$ [(M + H)$^+$], 177.0910; found 177.0912.

(*E*)-2-nitro-4-(penta-2,4-dien-1-yl)phenol (11p)

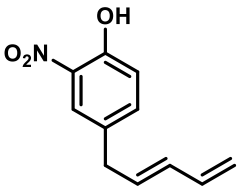

The title compound was synthesized from ether **10p** (100 mg, 0.49 mmol) following **general procedure B**. After column chromatography (petroleum ether/ ethyl acetate 4:1) the desired product **11p** was obtained as orange oil in 73 % yield (73 mg, 0.36 mmol); ^{1}H-NMR (400 MHz, $CDCl_3$): δ = 10.49 (s, 1H), 7.90 (d, J = 2.2 Hz, 1H), 7.41 (dd, J = 8.6, 2.2 Hz, 1H), 7.10 (d, J = 8.6 Hz, 1H), 6.41 – 6.27 (m, 1H), 6.17 – 6.05 (m, 1H), 5.84 – 5.72 (m, 1H), 5.17 (dd, J = 16.8, 0.9 Hz, 1H), 5.06 (dd, J = 10.2, 0.9 Hz,

1H), 3.41 (d, *J* = 6.6 Hz, 2H); ^{13}C-NMR (101 MHz, $CDCl_3$): δ = 153.8, 138.4, 136.6, 133.5, 133.3, 132.6, 131.7, 124.3, 120.1, 116.9, 37.7; HRMS (ESI): exact mass calculated for $C_{11}H_{10}NO_3^-$ [(M - H)$^-$], 204.0666; found 204.0688.

(*E*)-3-nitro-4-(penta-2,4-dien-1-yl)phenol (11q)

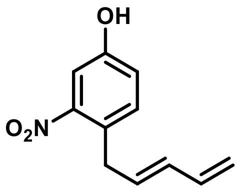

The title compound was synthesized from ether **10q** (100 mg, 0.49 mmol) following **general procedure B**. After column chromatography (petroleum ether/ ethyl acetate 8:1) the desired product **11q** was obtained as yellow solids in 40 % yield (40 mg, 0.19 mmol); ^{1}H-NMR (400 MHz, $CDCl_3$): δ = 7.42 (d, *J* = 2.7 Hz, 1H), 7.23 (d, *J* = 8.4 Hz, 1H), 7.03 (dd, *J* = 8.4, 2.7 Hz, 1H), 6.38 – 6.24 (m, 1H), 6.15 – 6.03 (m, 1H), 5.86 – 5.76 (m, 1H), 5.14 (d, *J* = 18.6 Hz, 1H), 5.03 (d, *J* = 10.3 Hz, 1H), 4.97 (s, 1H), 3.64 (d, *J* = 6.7 Hz, 2H); ^{13}C-NMR (101 MHz, $CDCl_3$): δ = 154.6, 149.3, 136.7, 133.2, 133.0, 131.3, 127.4, 120.9, 116.6, 111.7, 35.3; HRMS (ESI): exact mass calculated for $C_{11}H_{11}NO_3^-$ [M$^-$] , 205.0744; found 205.0714; m.p. 31-32 °C.

(*E*)-penta-2,4-dien-1,1-d$_2$-1-ol (S1)

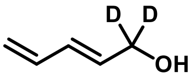

A 50 mL Schlenk was charged with (*E*)-penta-2,4-dienoic acid (513 mg, 5.23 mmol, 1 equiv) in 20 mL dry Et_2O and $LiAlD_4$ was added. The grey suspension was heated to reflux for 1 hour. After TLC (petroleum ether/ ethyl acetate 1:1) confirmed full consumption of starting material the reaction mixture was transferred into an ice-cooled 250 mL Erlenmeyer flask. Then, saturated aqueous Rochelle's salt solution was added dropwise, and the resulting quenched reaction mixture was stirred overnight. The next day, the aqueous layer was extracted with Et_2O, and the combined organic layer was dried over $MgSO_4$. The solvent was partially removed at 40 °C and 800 mBar and the desired product **S1** was obtained as colorless liquid in 55 % yield (549 mg, 45 % in Et_2O, 2.87 mmol); ^{1}H NMR (600 MHz, $CDCl_3$) δ = 6.35 – 6.25 (m, 1H), 6.20 (ddd, *J* = 15.3, 10.4, 0.8 Hz, 1H), 5.78 (dd, *J* = 15.0, 0.9 Hz, 1H), 5.16 (ddd, *J* = 16.9, 1.6, 0.8 Hz, 1H), 5.04 (ddd, *J* = 10.0, 1.6, 0.7 Hz, 1H); ^{13}C NMR (151 MHz, $CDCl_3$) δ = 136.4, 132.7, 131.7, 117.4; HRMS (ESI): exact mass calculated for $C_5H_5D_2^+$ [(M -OH)$^+$], 69.0668; found 69.0689.

(*E*)-1-(methoxy-d$_3$)-2-((penta-2,4-dien-1-yl-1,1-d$_2$)oxy)benzene (10f-d$_5$)

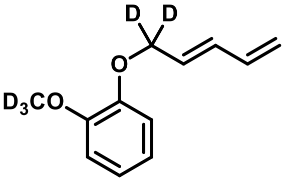

A 25 mL Schlenk flask was charged with 2-(methoxy-d$_3$)phenol (100 mg, 0.79 mmol, 1 equiv), PPh_3 (289 mg, 1.18 mmol, 1.4 equiv) and **S1** (226 mg, 45 % in Et_2O, 1.18 mmol, 1.5 equiv) in 2 mL dry THF. After five minutes, diisopropyl azodicarboxylate (0.22mL, 1.10 mmol, 1.4 equiv) in 0.8 mL dry THF was added. The dark yellow solution was stirred for 30 minutes, upon which TLC (petroleum ether/ ethyl acetate 5:1) indicated full consumption of starting material. The solvent was removed in vacuo and the crude material was subjected to column chromatography (petroleum ether/ ethyl acetate 30:1). The desired product was obtained as colorless oil in unimproved 29 % yield (44 mg, 0.23 mmol). ^{1}H NMR (400 MHz, $CDCl_3$) δ = 6.96 – 6.86 (m, 4H), 6.44 – 6.29 (m, 2H), 5.99 – 5.90 (m, 1H), 5.27 – 5.20 (m, 1H), 5.15 – 5.09 (m, 1H); ^{13}C NMR (151 MHz, $CDCl_3$) δ = 149.6, 148.1, 136.3, 134.2, 128.5, 121.4, 120.8, 118.3, 113.6, 111.8; HRMS (ESI): exact mass calculated for $C_{12}H_{10}D_5O_2^+$ [(M + H)$^+$], 196.1380; found 196.1383.

(*E*)-2-(methoxy-d$_3$)-4-(penta-2,4-dien-1-yl-5,5-d$_2$)phenol (11f-d$_5$)

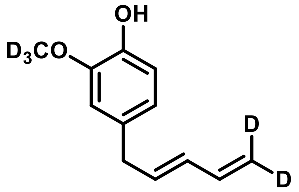

An oven-dried screw vial was charged with an equimolar mixture of compound **10a** (26 mg, 0.16 mmol, 1 equiv) and **10f-d$_5$** (32 mg, .016 mmol, 1 equiv) in 1 mL dry toluene. The solution was degassed by ten vacuo/Ar cycles. Then, EuFOD (17 mg, 5 mol % each) was added and the solution was degassed again ten times. The reaction vial was heated to 110 °C in a metal heating block. After 2 hours 30 minutes, TLC (petroleum ether/ ethyl acetate 10:1) confirmed full consumption of the compound **10f-d$_5$**. The solvent was removed in vacuo and the crude material was purified by column chromatography (petroleum ether/ ethyl acetate 20:1). Compound **11f-d$_5$** was obtained as yellow oil in 53 % yield (17 mg, 0.087mmol), accompanied by 69 % of starting material **10a** (18 mg, 0.11 mmol); ^{1}H NMR (600 MHz, $CDCl_3$) δ 6.85 (d, *J* = 7.9 Hz, 1H), 6.69 (d, *J* = 9.3 Hz, 2H), 6.34 (dd, *J* = 10.4, 3.2 Hz, 1H), 6.14 – 6.07 (m, 1H), 5.87 – 5.79 (m, 1H), 5.51 (s, 1H), 3.36 (d, *J* = 6.9 Hz, 2H); ^{13}C NMR (151 MHz, $CDCl_3$) δ 146.6, 144.1, 136.9, 134.0, 132.1, 131.9, 121.3, 114.4, 111.2, 38.7.

(*E*)-((penta-2,4-dien-1-yl-1,1-d$_2$)oxy)benzene (12)

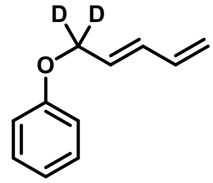

A 25 mL Schlenk flask was charged with compound **S1** (1.49 g, 24 % in Et_2O, 4.14 mmol, 1.3 equiv), PPh_3 (1.17 g, 4.46 mmol, 1.4 equiv) and phenol (300 mg, 3.19 mmol, 1 equiv) in 4 mL dry THF. The colorless solution was cooled by ice bath and after five minutes DIAD (0.88 mL, 4.46 mmol, 1.4 equiv) in 0.6 mL dry THF was added dropwise. After complete addition, the reaction mixture turned turbid. TLC (petroleum ether/ ethyl acetate 5:1) confirmed full consumption of starting material. All volatiles were evaporated, and the residue was taken up in EE. It was washed with 6 % H_2O_2 solution twice, followed by H_2O and Brine. The organic layer was dried over $MgSO_4$ and concentrated in vacuo. The crude red oil was purified by column chromatography (50 g silica, petroleum ether ethyl acetate 30:1). The desired product **12** was obtained as colorless oil in unimproved 38 % yield (199mg, 1.23 mmol). ^{1}H-NMR (400 MHz, $CDCl_3$): δ = 7.33 – 7.27 (m, 2H), 6.99 – 6.91 (m, 3H), 6.45 – 6.34 (m, 2H), 5.96 – 5.88 (m, 1H), 5.32 – 5.22 (m, 1H), 5.21 – 5.11 (m, 1H); ^{13}C-NMR (101 MHz, $CDCl_3$): δ = 158.7, 136.3, 134.0, 129.6, 128.4, 121.0, 118.3, 114.8; HRMS (APCI): exact mass calculated for $C_{11}H_{11}D_2O^+$ [(M +H)$^+$], 163.1086; found 163.1091.

(*E*)-4-(penta-2,4-dien-1-yl-5,5-d$_2$)phenol (13)

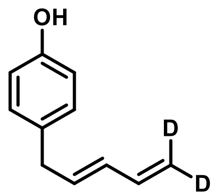

The title compound was synthesized from ether **12** (100 mg, 0.62 mmol) following **general procedure B**. After column chromatography (petroleum ether/ ethyl acetate 30:1) the desired product **13** was obtained as pale-yellow oil in 33 % yield (33 mg, 0.20 mmol). ^{1}H-NMR (600 MHz, $CDCl_3$): δ = 7.06 (d, *J* = 8.0 Hz, 2H), 6.78 (d, J = 7.9 Hz, 2H), 6.33 (dd, *J* = 10.4, 3.2 Hz, 1H), 6.10 (dd, *J* = 15.1, 10.4 Hz, 1H), 5.83 (dt, *J* = 14.6, 6.9 Hz, 1H), 4.93 (s, 1H), 3.36 (d, *J* = 7.0 Hz, 2H); ^{13}C-NMR (151 MHz, $CDCl_3$): δ = 153.9, 136.9, 134.0, 132.4, 131.9, 129.9, 115.4, 38.1; HRMS (ESI): exact mass calculated for $C_{11}H_{11}D_2O^+$ [(M + H)$^+$], 163.1086; found 163.1087.

(*E*)-1,3-Dimethyl-2-((penta-2,4-dien-1-yl-1,1-d$_2$)oxy)benzene (14)

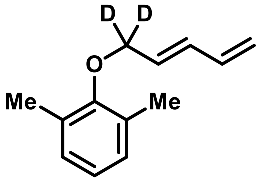

A 25 mL Schlenk flask was charged with 2,6-dimethylphenol (120 mg, 0.98 mmol, 1 equiv) in 5 ml dry toluene and **S1** (244 mg, 45 % in Et_2O, 1.28 mmol, 1.3 equiv) in 2 mL dry toluene was added. The mixture was cooled by ice bath and PBu_3 (0.31 mL, 93 %, 1.18 mmol, 1.15 equiv) was added. After 5 minutes, diisopropyl azodicarboxylate in 2 mL dry toluene was added dropwise. The dark yellow solution was stirred overnight, upon which TLC (petroleum ether/ ethyl acetate 5:1) indicated full consumption of starting material. Ethyl acetate was added to the reaction mixture and the organic layer was washed with 6 % H_2O_2 solution twice, followed by H_2O. The organic layer was dried over $MgSO_4$ and concentrated in vacuo. The crude material filtered over silica gel with Et_2O, and desired product **14** was obtained as colorless oil in unimproved 24 % yield (45 mg, 0.24 mmol). ^1H NMR (400 MHz, $CDCl_3$) δ = 7.04 – 7.01 (m, 2H), 6.93 (dd, *J* = 8.2, 6.6 Hz, 1H), 6.47 – 6.36 (m, 2H), 6.03 – 5.92 (m, 1H), 5.35 – 5.21 (m, 1H), 5.21 – 5.10 (m, 1H), 2.30 (s, 6H), ^{13}C-NMR (101 MHz, $CDCl_3$): δ = 156.0, 136.4, 133.5, 131.2, 129.3, 128.9, 128.9, 124.0, 118.2, 71.7, 16.5; HRMS (APCI): exact mass calculated for $C_{13}H_{15}D_2O^+$ [(M + H)$^+$], 191.1399; found 191.1400.

(*E*)-2,6-Dimethyl-4-(penta-2,4-dien-1-yl-5,5-d$_2$)phenol (15)

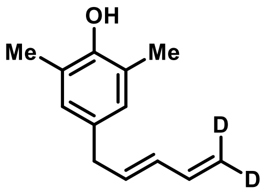

The title compound was synthesized from ether **14** (45 mg, 0.24 mmol) following **general procedure B**. After column chromatography (petroleum ether/ ethyl acetate 5:1) the desired product **15** was obtained as orange solids in 93 % yield (42 mg, 0.22 mmol); ^1H-NMR (600 MHz, $CDCl_3$): δ = 6.84 (s, 2H), 6.34 (d, *J* = 9.6 Hz, 1H), 6.12 (ddt, *J* = 15.1, 10.5, 1.5 Hz, 1H), 5.84 (dt, *J* = 14.6, 7.0 Hz, 1H), 5.05 (s, 1H), 3.32 (dd, *J* = 7.0, 1.5 Hz, 2H), 2.30 (s, 6H); ^{13}C-NMR (151 MHz, $CDCl_3$): δ = 150.9, 137.0, 134.3, 131.8, 131.6, 128.9, 123.3, 115.0 (p, *J* = 24.1 Hz), 38.3, 16.1; HRMS (ESI): exact mass calculated for $C_{13}H_{15}D_2O^+$ [(M + H)$^+$], 191.1399; found 191.1400; m.p. 49-50 °C.

Tert-butyl (*E*)-4-phenoxybut-2-enoate (S2)

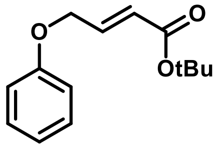

A 250 ml Schlenk flask was charged with (allyloxy)benzene (590 mg, 4.40 mmol, 1 equiv) and *tert*-butyl acrylate in 45 mL dry degassed (freeze thaw 2 cycles) DCM. The mix was stirred for five minutes, before Grubbs 2nd generation catalyst (189 mg, 0.22 mmol, 5 mol %) was added. The mixture was heated to reflux and after four hours TLC (petroleum ether/ ethyl acetate 5:1) confirmed full conversion. All volatiles were evaporated, and the crude brown oil was purified by column chromatography (40 g silica, petroleum ether ethyl acetate 20:1). The desired product **S2** was obtained in 71 % yield (734 mg, 3.13 mmol). ^1H NMR (400 MHz, $CDCl_3$) δ = 7.33 – 7.27 (m, 2H), 7.02 – 6.95 (m, 2H), 6.94 – 6.89 (m, 2H), 6.12 (dt, *J* = 15.7, 2.1 Hz, 1H), 4.68 (dd, *J* = 4.2, 2.0 Hz, 2H), 1.50 (s, 9H); ^{13}C NMR (101 MHz, $CDCl_3$) δ = 165.5, 158.3, 141.3, 129.7, 124.0, 121.4, 114.8, 80.8, 66.6, 28.2; HRMS (ESI): exact mass calculated for $C_{14}H_{19}O_3^+$ [(M + H)$^+$], 235.1329; found 235.1327.

(*E*)-4-phenoxybut-2-enal (S3)

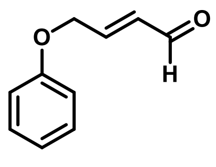

A 100 ml Schlenk flask was charged with compound **S2** (700 mg, 2.99 mmol, 1 equiv) in 30 mL dry DCM and cooled to -90 °C. After five minutes DIBAL (1.0 M in hexanes, 3.59 mL, 3.59 mmol, 1.20 equiv) was added dropwise over ten minutes. TLC (petroleum ether/ ethyl acetate 10:1) after one hour confirmed full conversion of the starting material. The reaction was quenched by addition of saturated methanolic NH_4Cl solution followed by saturated aqueous Seignette's salt solution. The mixture was removed from cooling and stirred at rt for 30 minutes. The aqueous layer was extracted with DCM three times and the combined organic layer was dried over $MgSO_4$. It was concentrated in vacuo and crude material was purified by flash chromatography (5 g silica, petroleum ether/ ethyl acetate 5:1). The desired product **S3** was obtained as yellow oil in 87 % yield (421 mg, 2.60 mmol). ^{1}H NMR (600 MHz, $CDCl_3$) δ = 9.62 (d, *J* = 7.8 Hz, 1H), 7.33 – 7.29 (m, 2H), 7.02 – 6.99 (m, 1H), 6.96 (dt, *J* = 15.9, 4.0 Hz, 1H), 6.93 – 6.91 (m, 2H), 6.48 (ddt, *J* = 15.8, 7.8, 2.0 Hz, 1H), 4.82 (dd, *J* = 4.0, 2.0 Hz, 2H); ^{13}C NMR (151 MHz, $CDCl_3$) δ = 193.1, 157.9, 151.1, 132.4, 129.8, 121.7, 114.7, 66.4; HRMS (ESI): exact mass calculated for $C_{10}H_{11}O_2^+$ [(M + H)$^+$], 163.0754; found 163.0754.

(*E*)-((penta-2,4-dien-1-yl-5,5-d$_2$)oxy)benzene (16)

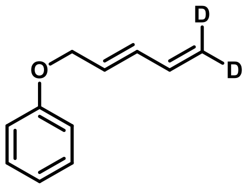

A 100 mL Schlenk flask was charged with CD_3PPh_3I (1.45 g, 3.56 mmol, 1.5 equiv) in 40 mL dry THF and the white suspension was cooled to -7 °C. After 5 minutes, *n*-BuLi (2.50 M in hexanes, 1.23 mL, 3.09 mmol, 1.30 equiv) was added dropwise causing a color change to yellow. The reaction mixture was kept at -7 °C for one hour before compound **S3** (385 mg, 2.37 mmol, 1 equiv) dissolved in 15 mL dry THF was added. The color changed from yellow over orange to brown after complete addition. TLC (petroleum ether/ ethyl acetate 3:1) after 30 minutes, confirmed full consumption of starting material. The reaction was quenched by addition of saturated aqueous NH_4Cl solution and H_2O. The aqueous layer was extracted with Et_2O three times, and the combined organic layer was washed with H_2O and Brine. It was dried over $MgSO_4$ and concentrated in vacuo. To remove excess of phosphine the crude yellow material was taken up in ethyl acetate and washed with 6 % H_2O_2 solution followed by H_2O and Brine. It was dried over $MgSO_4$ and concentrated in vacuo. The residue was purified by flash chromatography (petroleum ether/ ethyl acetate 15:1) and the desired product was obtained as colorless oil in unimproved 57 % yield (219 mg, 1.35 mmol). ^{1}H NMR (600 MHz, $CDCl_3$) δ = 7.33 – 7.28 (m, 2H), 6.99 – 6.95 (m, 1H), 6.95 – 6.92 (m, 2H), 6.43 – 6.36 (m, 2H), 5.98 – 5.88 (m, 1H), 4.59 (d, *J* = 6.4 Hz, 2H); ^{13}C NMR (151 MHz, $CDCl_3$) δ = 158.7, 136.0, 133.8, 129.6, 128.5, 121.0, 114.8, 68.1; HRMS (APCI): exact mass calculated for $C_{11}H_{11}D_2O^+$ [(M + H)$^+$], 163.1086; found 163.1091.

(*E*)-4-(penta-2,4-dien-1-yl-1,1-d$_2$)phenol (17)

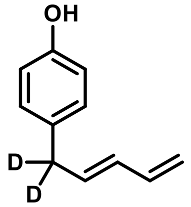

The title compound was synthesized from ether **16** (103 mg, 0.63 mmol) following **general procedure B**. After column chromatography (petroleum ether/ ethyl acetate 20:1) the desired product **17** was obtained as yellow oil in 75 % yield (78 mg, 0.48 mmol); ^{1}H-NMR (600 MHz, $CDCl_3$): δ = 7.09 – 7.04 (m, 2H), 6.82 – 6.75 (m, 2H), 6.35 (dt, *J* = 17.0, 10.3 Hz, 1H), 6.10 (dd, *J* = 15.2, 10.4 Hz, 1H), 5.83 (d, *J* = 15.1 Hz, 1H), 5.21 (s, 1H), 5.15 (dd, *J* = 17.0, 1.8 Hz, 1H), 5.03 (dd, *J* = 10.2, 1.8 Hz, 1H); ^{13}C NMR (151 MHz, $CDCl_3$) δ = 153.9, 137.1, 133.9, 132.3, 131.9, 129.9, 115.8, 115.4; HRMS (APCI): exact mass calculated for $C_{11}H_9D_2O^-$ [(M - H)$^-$], 161.0941; found 161.0937.

2-(penta-1,4-dien-3-yl)phenol (18)

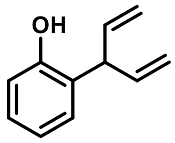

A Schlenk tube was charged with ether **10a** (380 mg, 2.37 mmol, 1 equiv) in 2.4 mL diethyl aniline. The yellow solution was heated to 186° C under Ar. After reached the desired temperature, the valve was closed, and the solution was heated for 4 hours. After TLC (petroleum ether/ ethyl acetate 10:1) indicated full consumption of starting material the reaction mixture was taken up in pentane and washed with 2 M H_2SO_4 4 times followed by H_2O and Brine. It was dried over $MgSO_4$ and concentrated in vacuo. The crude orange oil was purified by column chromatography (pentane to pentane/ Et_2O 99:1 --> 50:1 --> 10:1) and desired product **18** was obtained as colorless oil in 12 % yield (45 mg, 0.28 mmol), accompanied by 19 % of compound **11a** (71 mg, 0.44 mmol); ^1H NMR (400 MHz, $CDCl_3$) δ = 7.21 – 7.10 (m, 2H), 6.92 (td, *J* = 7.5, 1.3 Hz, 1H), 6.84 (dd, *J* = 8.0, 1.2 Hz, 1H), 6.12 (ddd, *J* = 17.3, 10.3, 6.2 Hz, 2H), 5.26 (*app.* dt, *J* = 10.3, 1.5 Hz, 2H), 5.17 (*app.* dt, *J* = 17.3, 1.6 Hz, 2H), 5.12 (broad s, 1H), 4.32 – 4.26 (m, 1H); ^{13}C-NMR (101 MHz, $CDCl_3$): δ = 154.0, 138.6, 129.5, 128.3, 127.4, 121.2, 116.6, 116.6, 47.9; HRMS (ESI): exact mass calculated for $C_{11}H_{13}O^+$ [$(M + H)^+$], 161.0961; found 161.0962.

Representative large-scale preparation of 9a

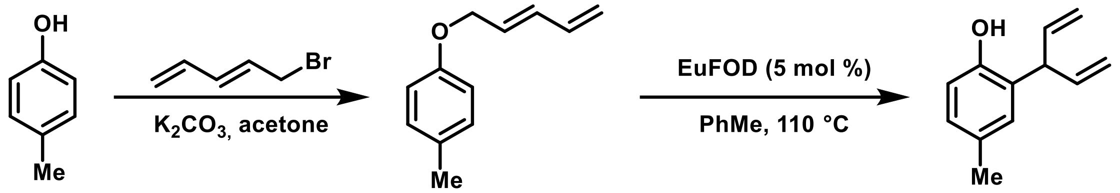

8a 9a

A 250 mL round bottom flask was charged with *p*-cresol (2.0 g, 18.49 mmol), K_2CO_3 (4.09 g, 29.59 mmol, 1.6 equiv) and literature known (*E*)-5-bromopenta-1,3-diene [2](2.99 g, 20.34 mmol, 1.1 equiv) in 93 mL dry acetone. A reflux condenser was attached, and the mixture was heated to reflux. After 50 minutes, TLC (petroleum ether/ ethyl acetate 5:1) confirmed full conversion of starting material. The reaction was quenched by addition of H_2O and extracted with DCM three times. The combined organic layer was dried over $MgSO_4$ and concentrated under reduced pressure. The crude material was purified by column chromatography (115 g silica, petroleum ether/ ethyl acetate 30:1) and the desired product was obtained as pale-yellow oil in 68 % yield (2.18 g, 12.51 mmol). Spectroscopic data were identical to those reported for compound **8a** above. A 250 mL Schlenk flask was charged with the just prepared ether **8a** (2.18 g, 12.51 mmol) and dissolved in 60 mL dry toluene. The mixture was degassed by ten vacuo/Ar cycles. Then, EuFOD (650 mg, 0.625 mmol, 5 mol %) was added and the solution was degassed again ten times. The flask was heated to 110 °C in a preheated oil bath. After 4 hours, TLC (petroleum ether/ ethyl acetate 10:1) confirmed full consumption of starting material. The flask was removed from heating and allowed to cool to room temperature. Subsequently, 50 mL H_2O was added, and the biphasic mixture was stirred overnight. The next day, layers were separated, and the organic layer was washed with H_2O eight times followed by Brine. It was dried over $MgSO_4$ and concentrated in vacuo. The crude material was purified by column chromatography (110 g silica, petroleum ether/ ethyl acetate 20:1) and the desired product was obtained as yellow oil in 85 % yield (1.85 g, 10.62 mmol). Spectroscopic data were identical to those reported for compound **9a** above.

NMR spectra of all prepared compounds

(*E*)-1-methyl-4-(penta-2,4-dien-1-yloxy)benzene (8a)

^1H NMR (400 MHz, $CDCl_3$)

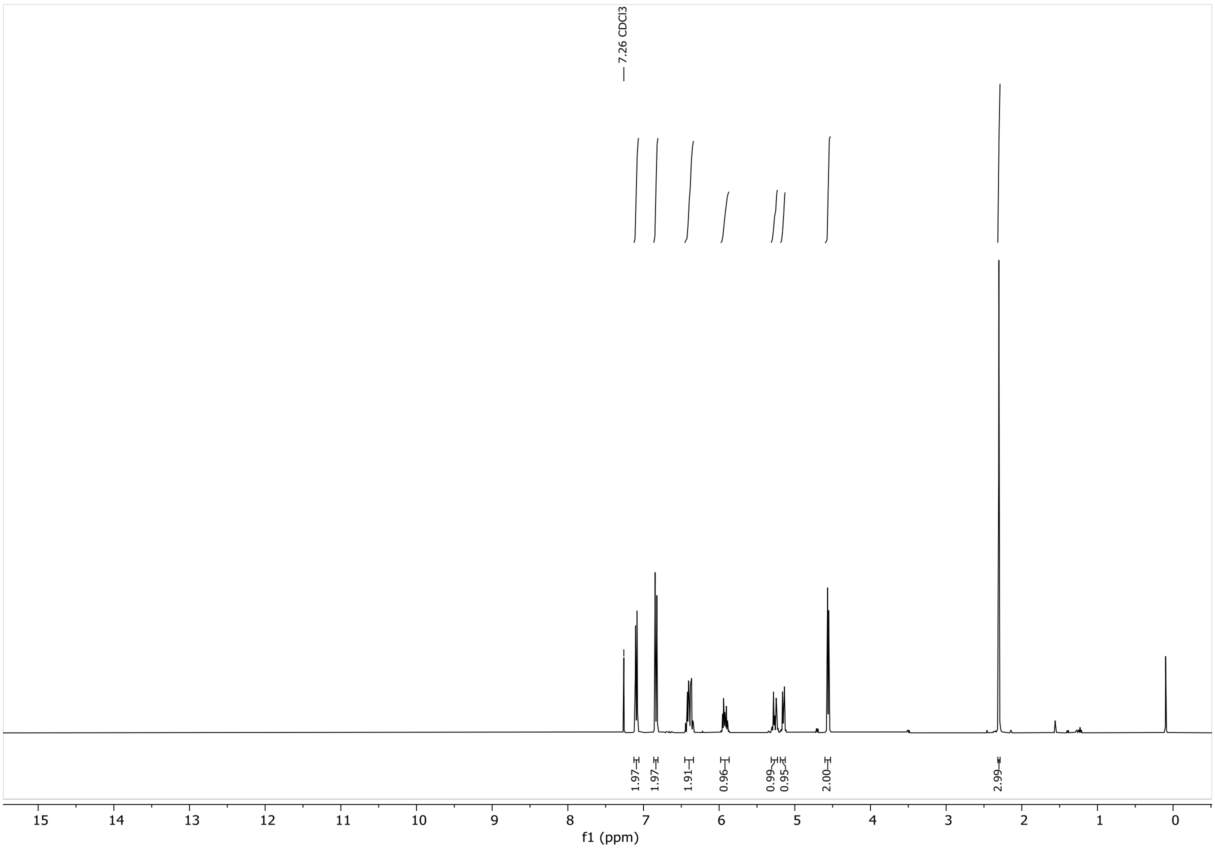

^{13}C-NMR (101 MHz, $CDCl_3$)

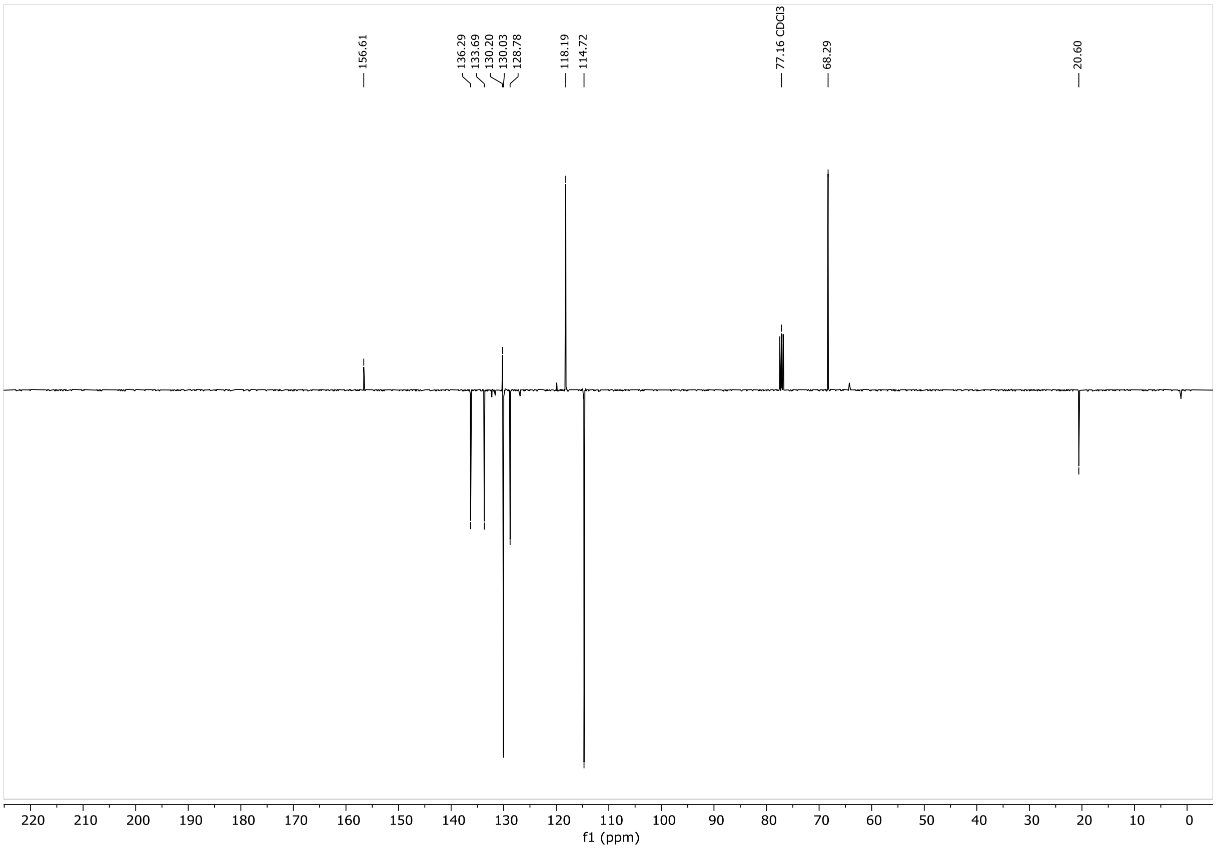

(*E*)-1-(tert-butyl)-4-(penta-2,4-dien-1-yloxy)benzene (8b)

^{1}H NMR (400 MHz, $CDCl_3$)

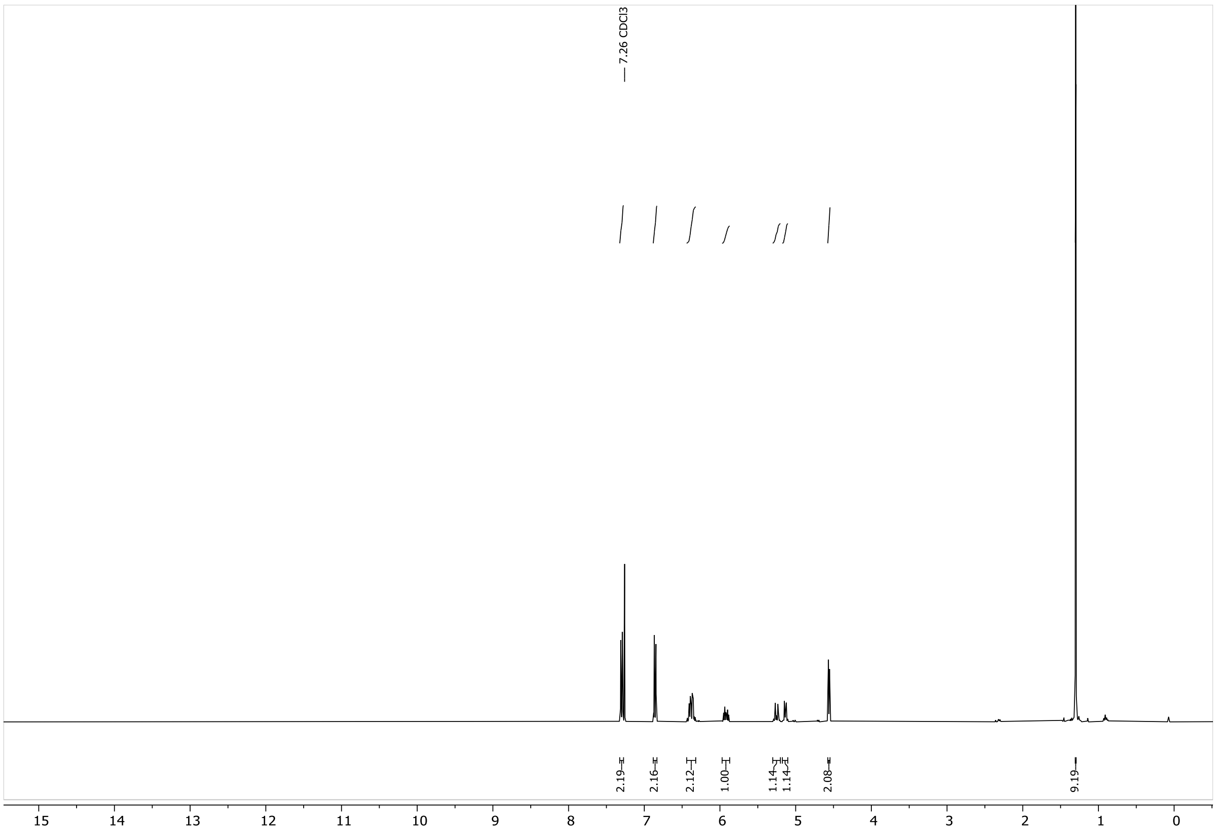

^{13}C-NMR (101 MHz, $CDCl_3$)

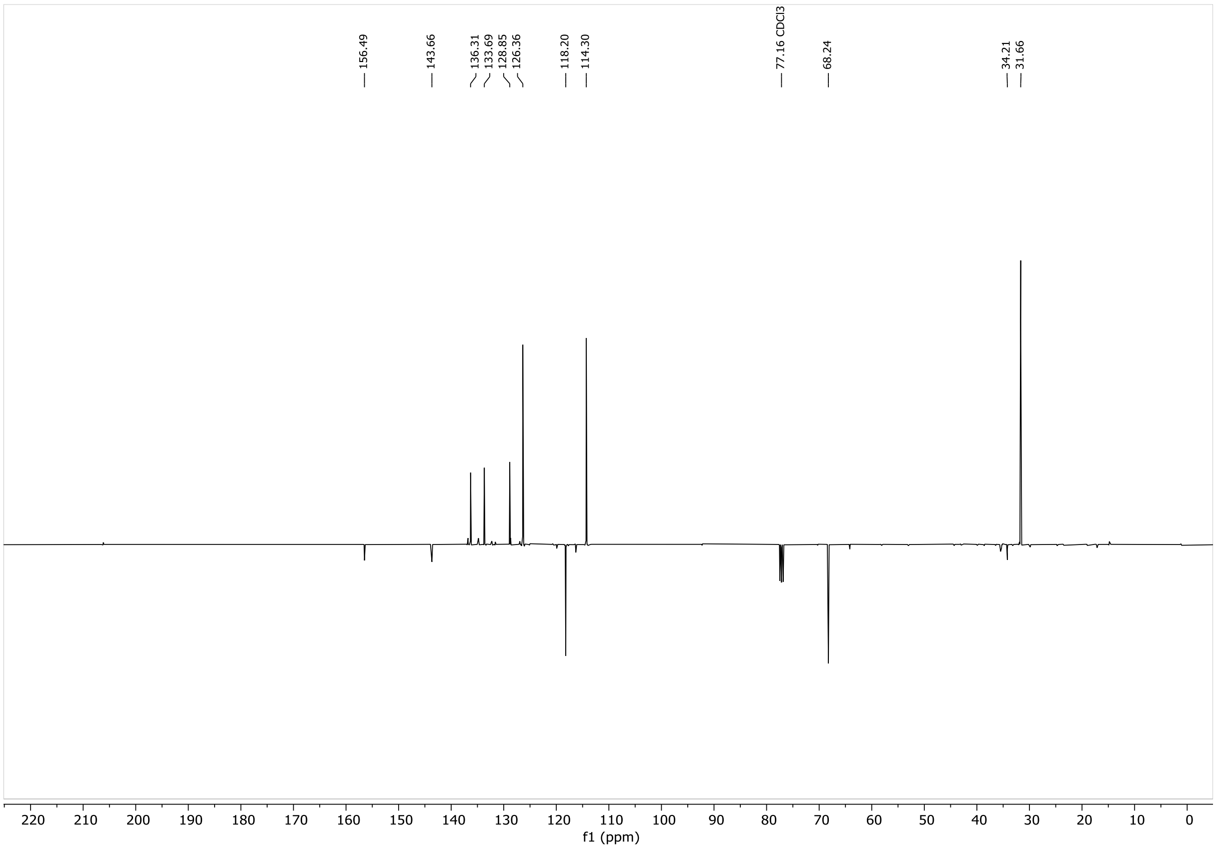

(*E*)-4-(Penta-2,4-dien-1-yloxy)-1,1'-biphenyl (8c)

^{1}H NMR (400 MHz, $CDCl_3$)

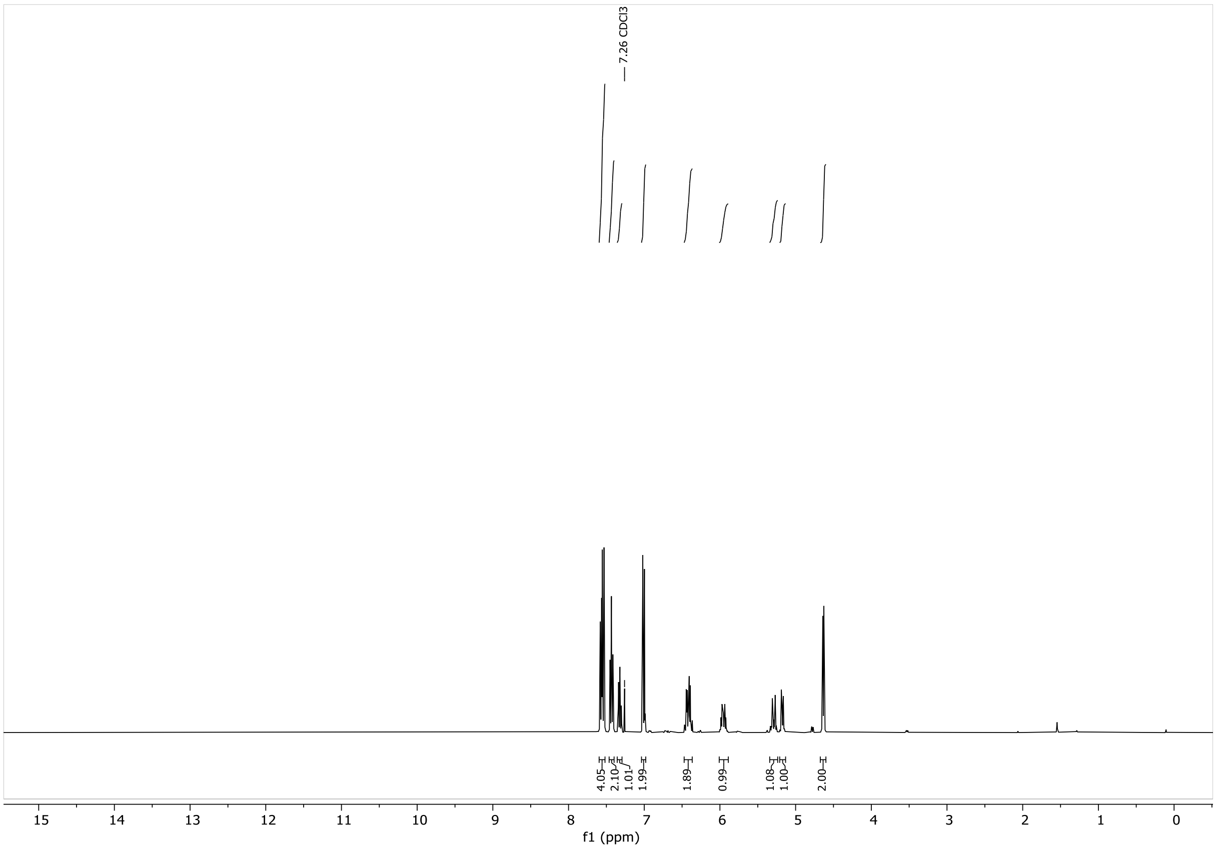

^{13}C-NMR (101 MHz, $CDCl_3$)

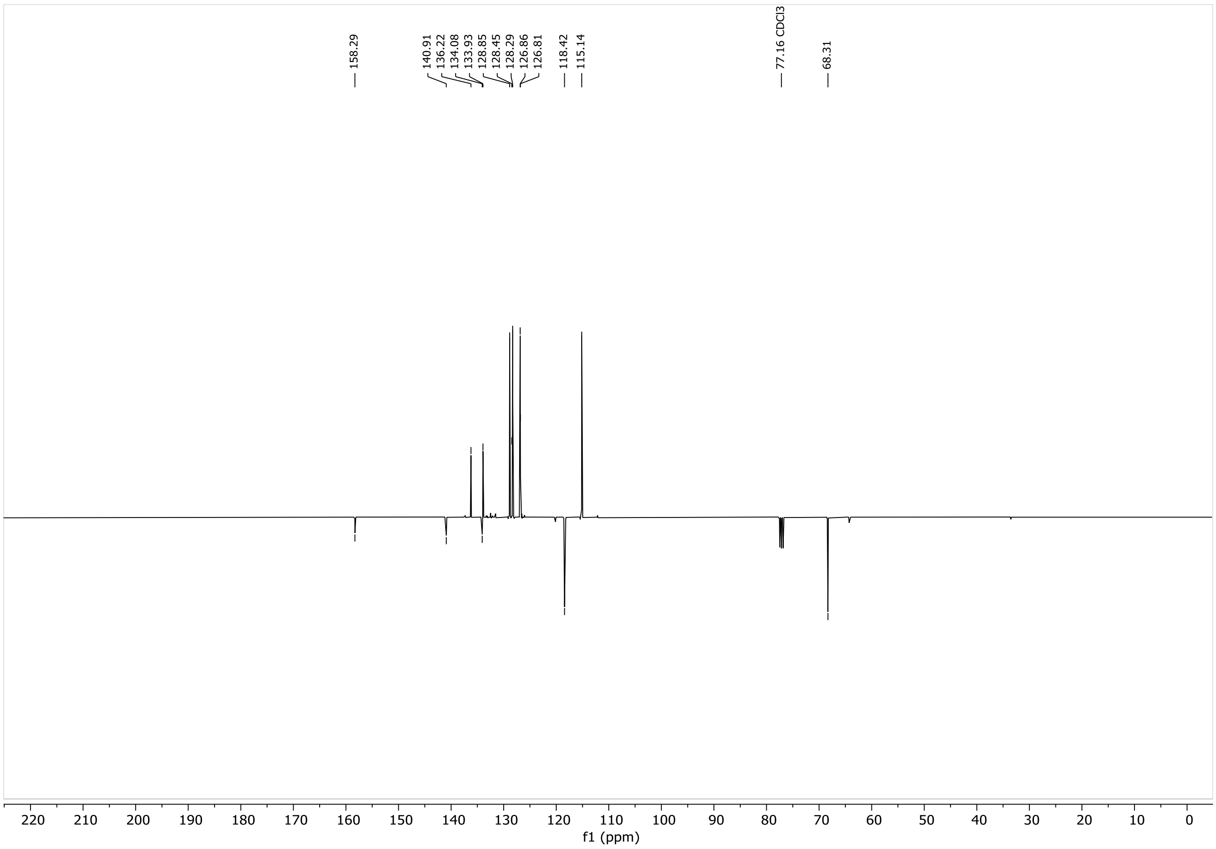

(*E*)-4-allyl-2-methoxy-1-(penta-2,4-dien-1-yloxy)benzene (8d)

^{1}H NMR (400 MHz, $CDCl_3$)

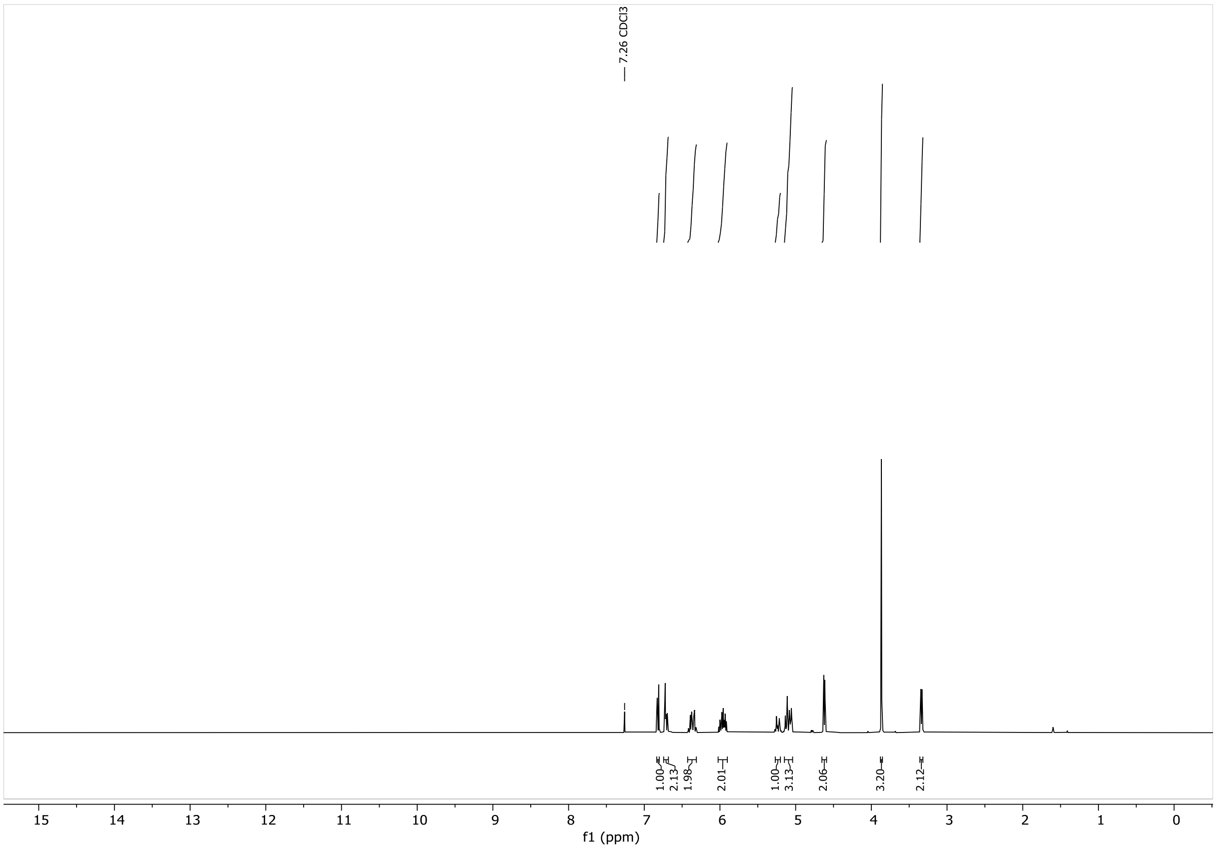

^{13}C-NMR (101 MHz, $CDCl_3$)

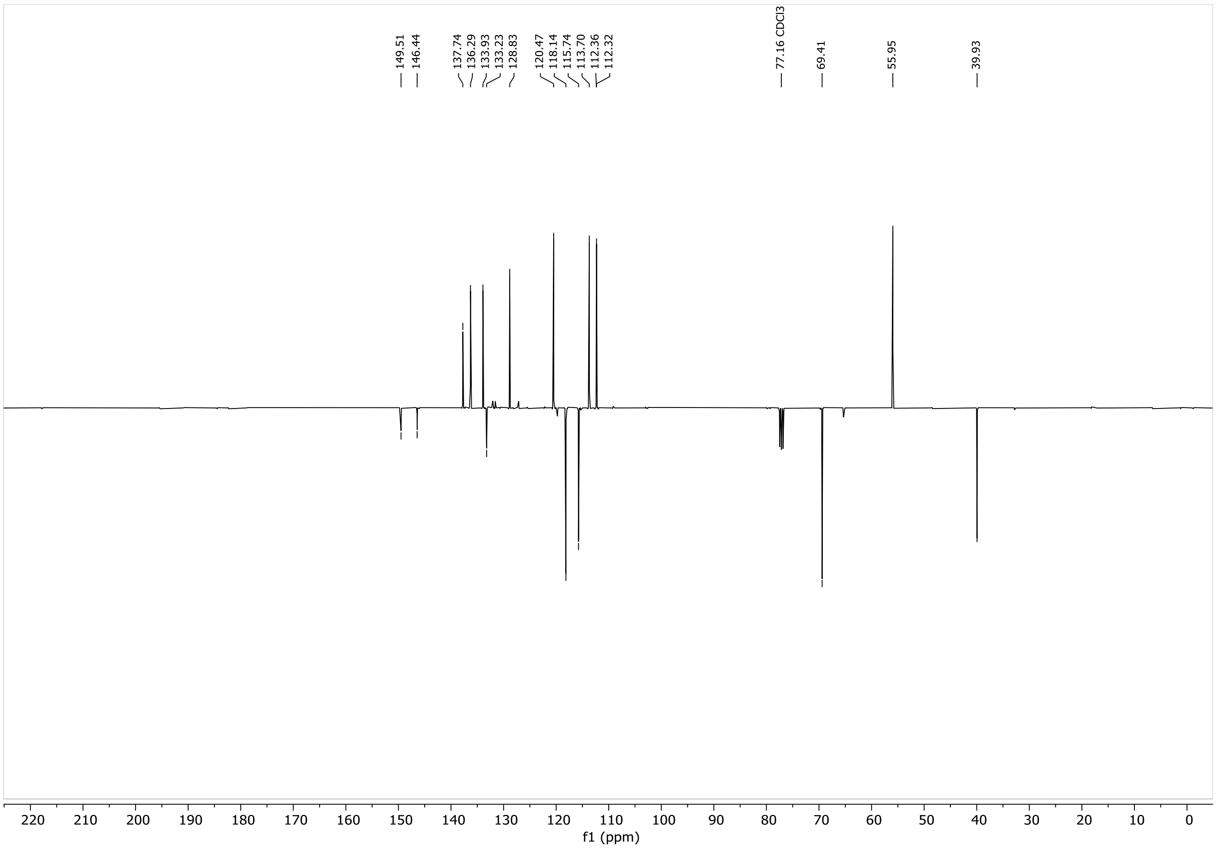

(*E*)-1-methoxy-4-(penta-2,4-dien-1-yloxy)benzene (8e)

^{1}H NMR (600 MHz, $CDCl_3$)

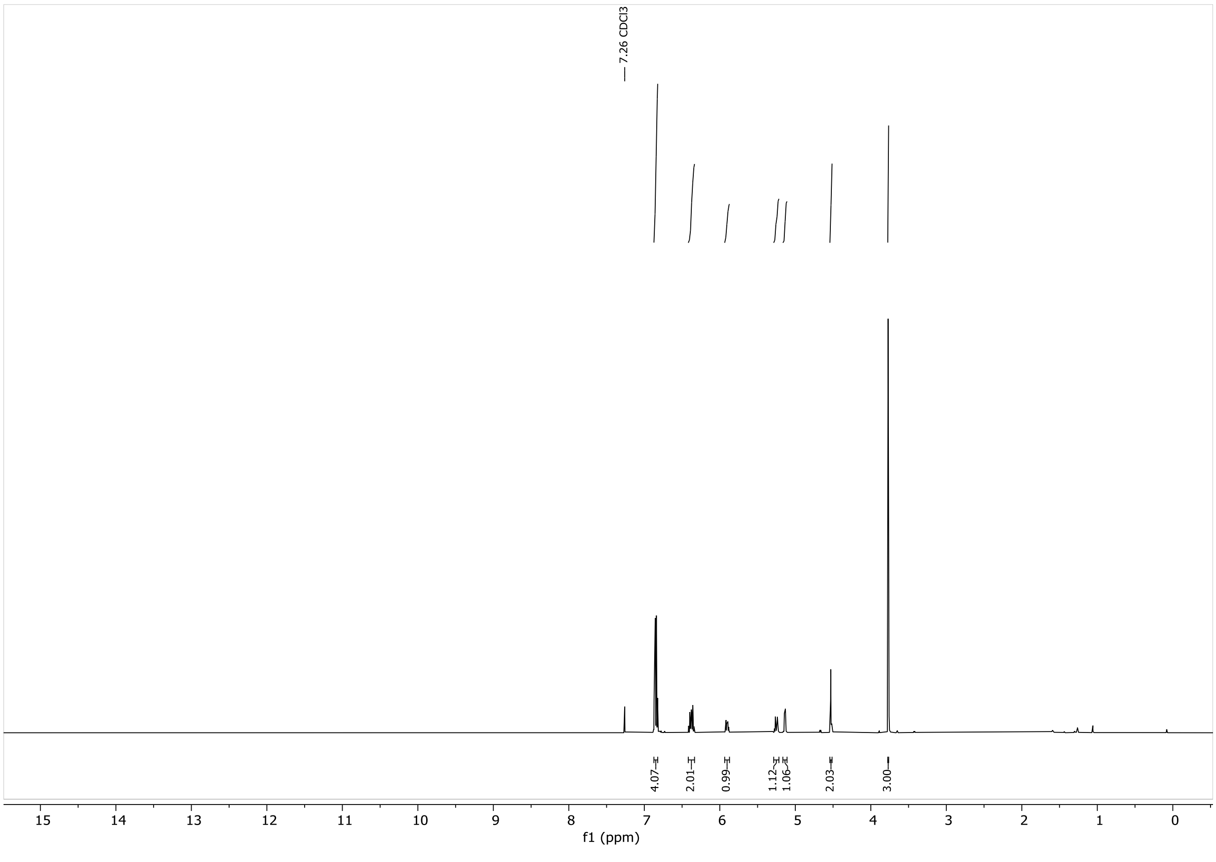

^{13}C-NMR (151 MHz, $CDCl_3$)

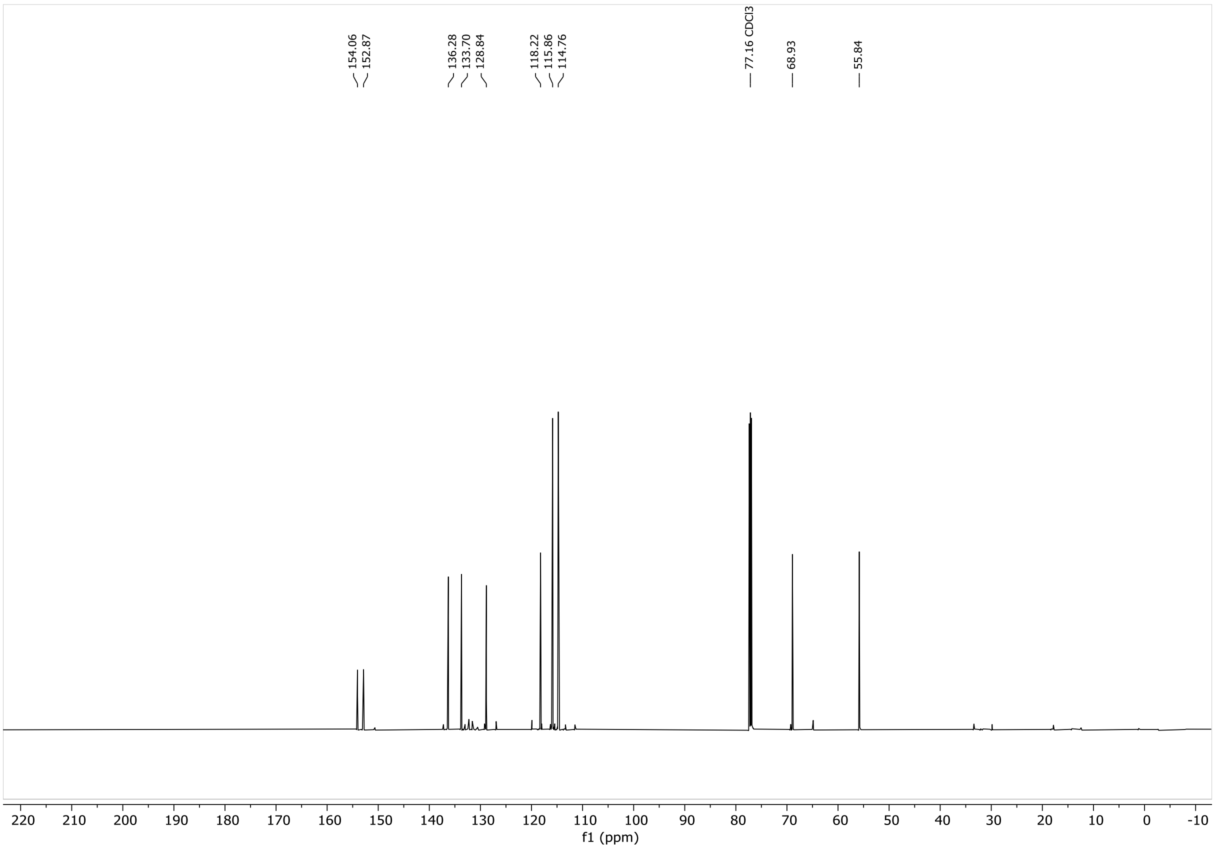

(*E*)-1-(benzyloxy)-4-(penta-2,4-dien-1-yloxy)benzene (8f)

^{1}H NMR (400 MHz, $CDCl_3$)

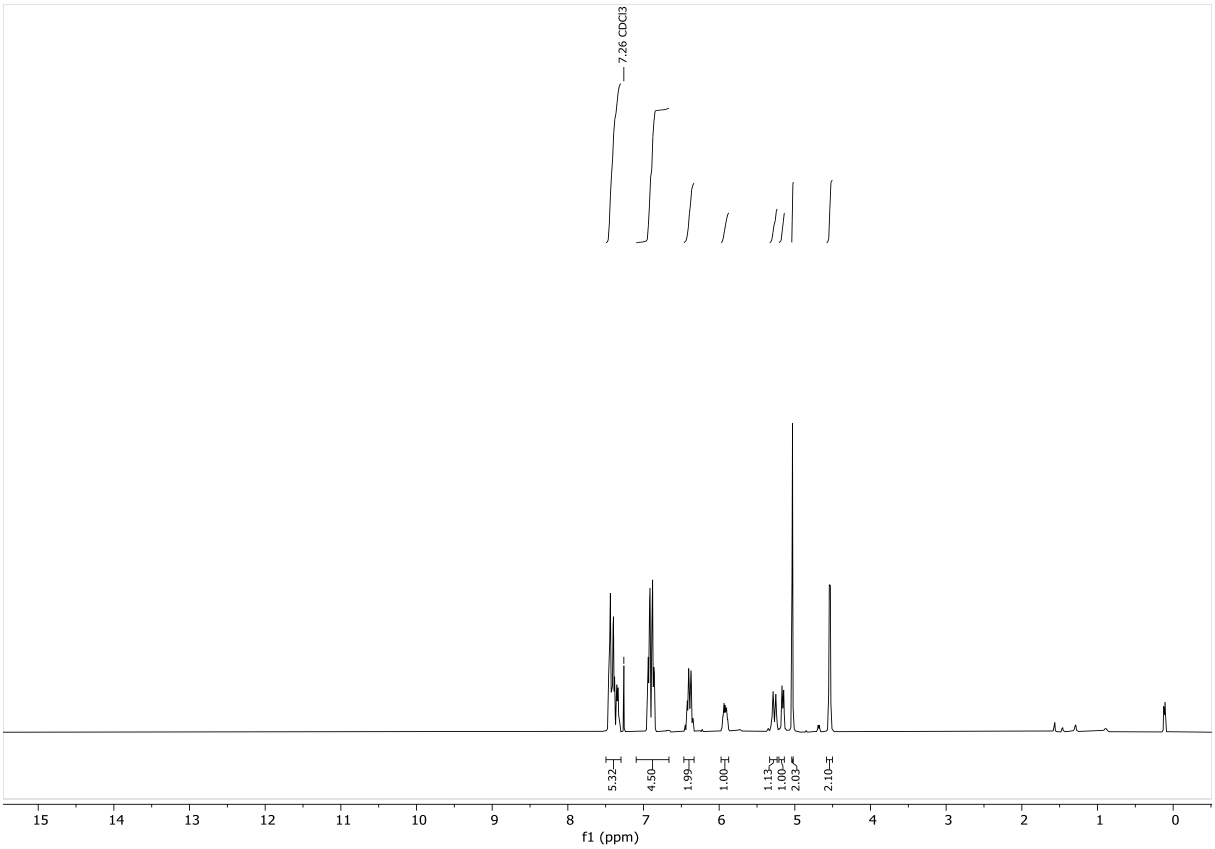

^{13}C-NMR (101 MHz, CDCl$_3$)

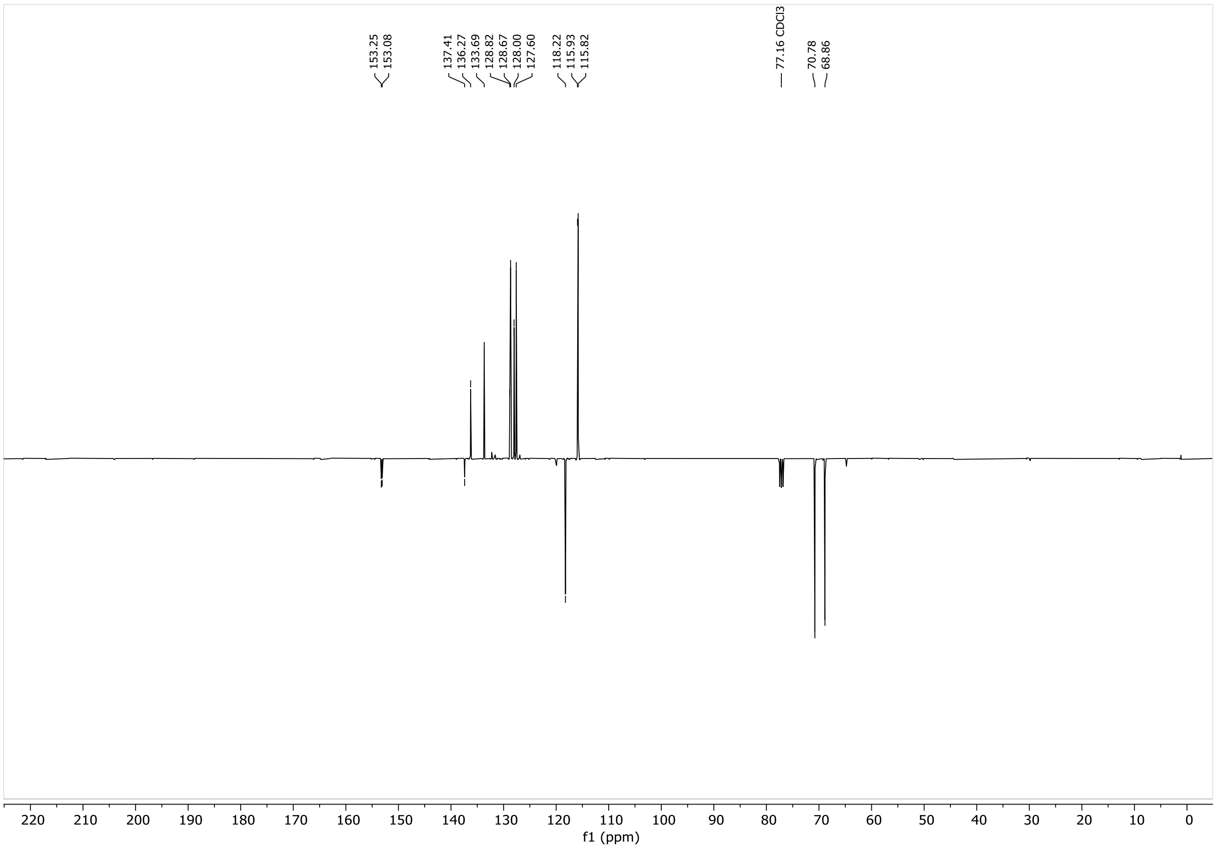

(*E*)-2-chloro-4-methoxy-1-(penta-2,4-dien-1-yloxy)benzene (8g)

^{1}H NMR (400 MHz, CDCl$_3$)

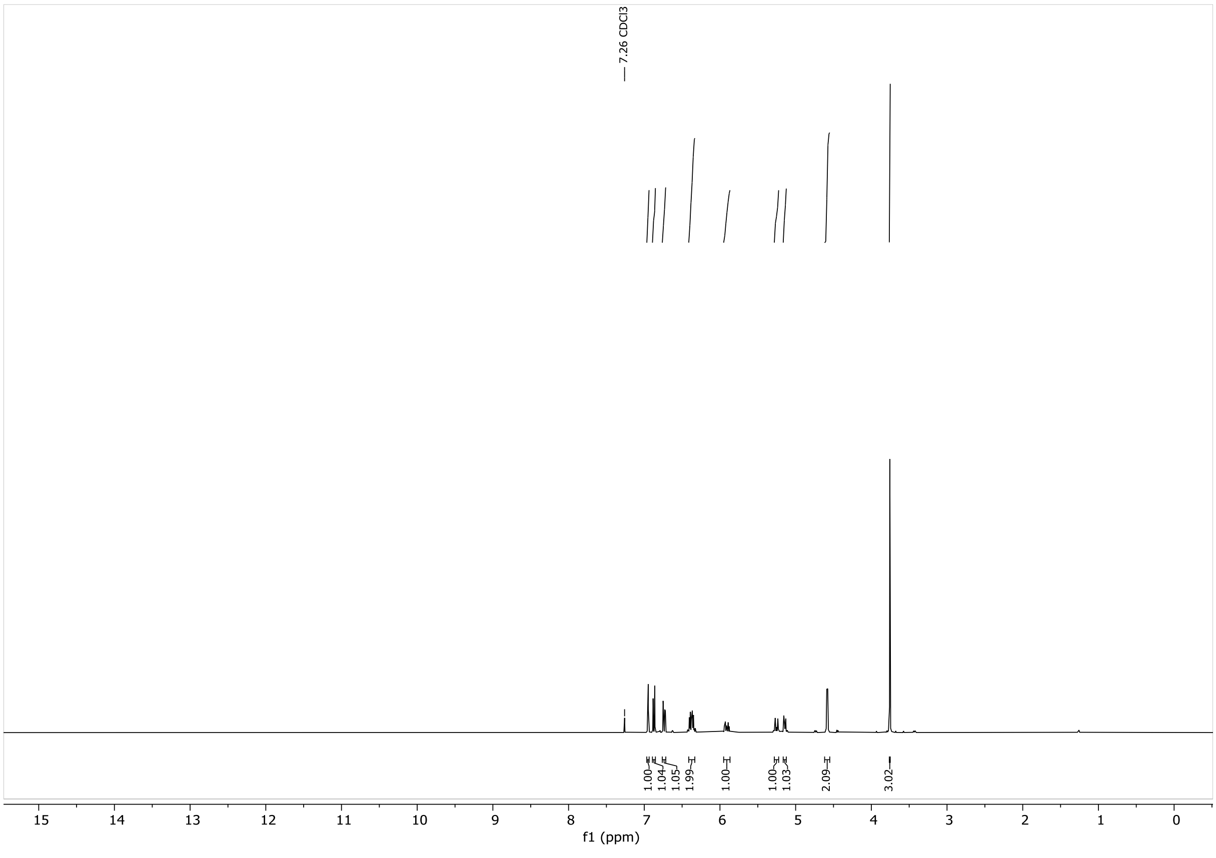

^{13}C-NMR (101 MHz, $CDCl_3$)

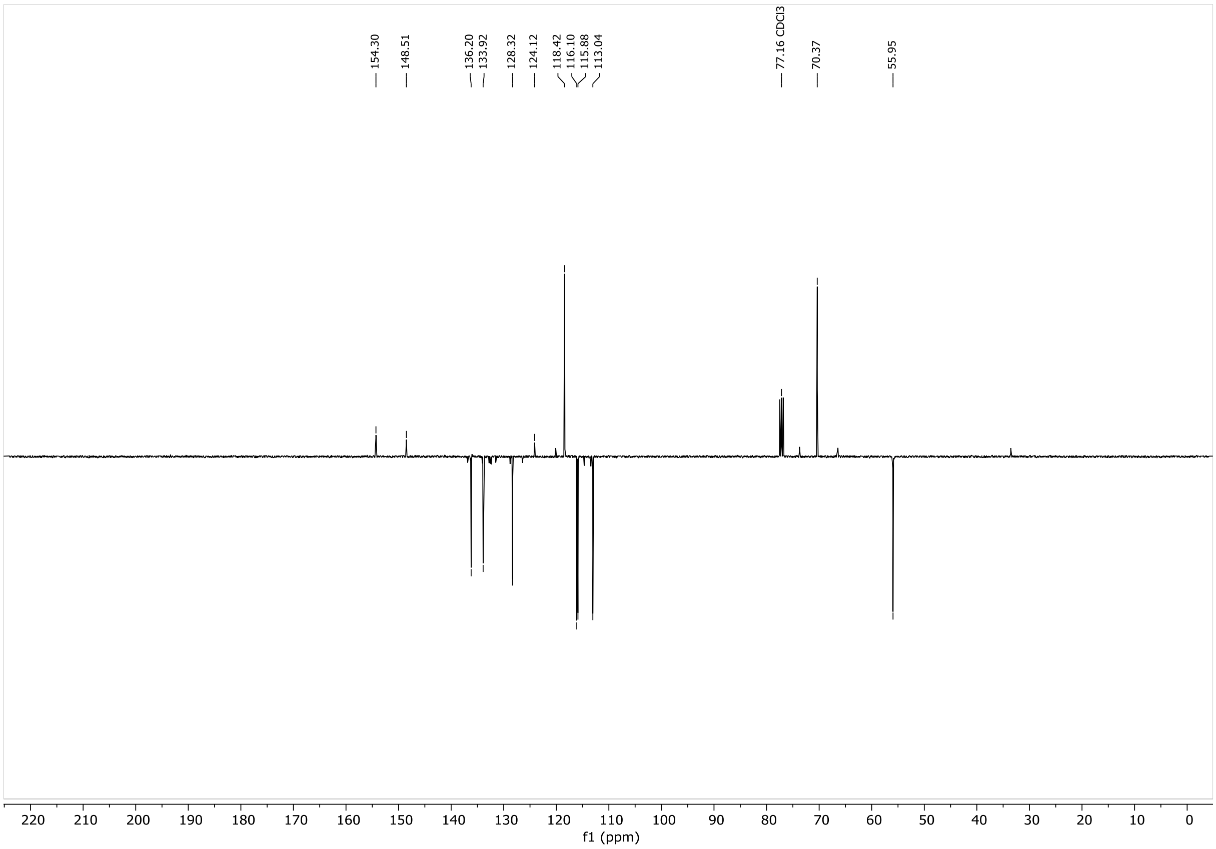

(*E*)-1-chloro-4-(penta-2,4-dien-1-yloxy)benzene (8h)

^{1}H NMR (400 MHz, $CDCl_3$)

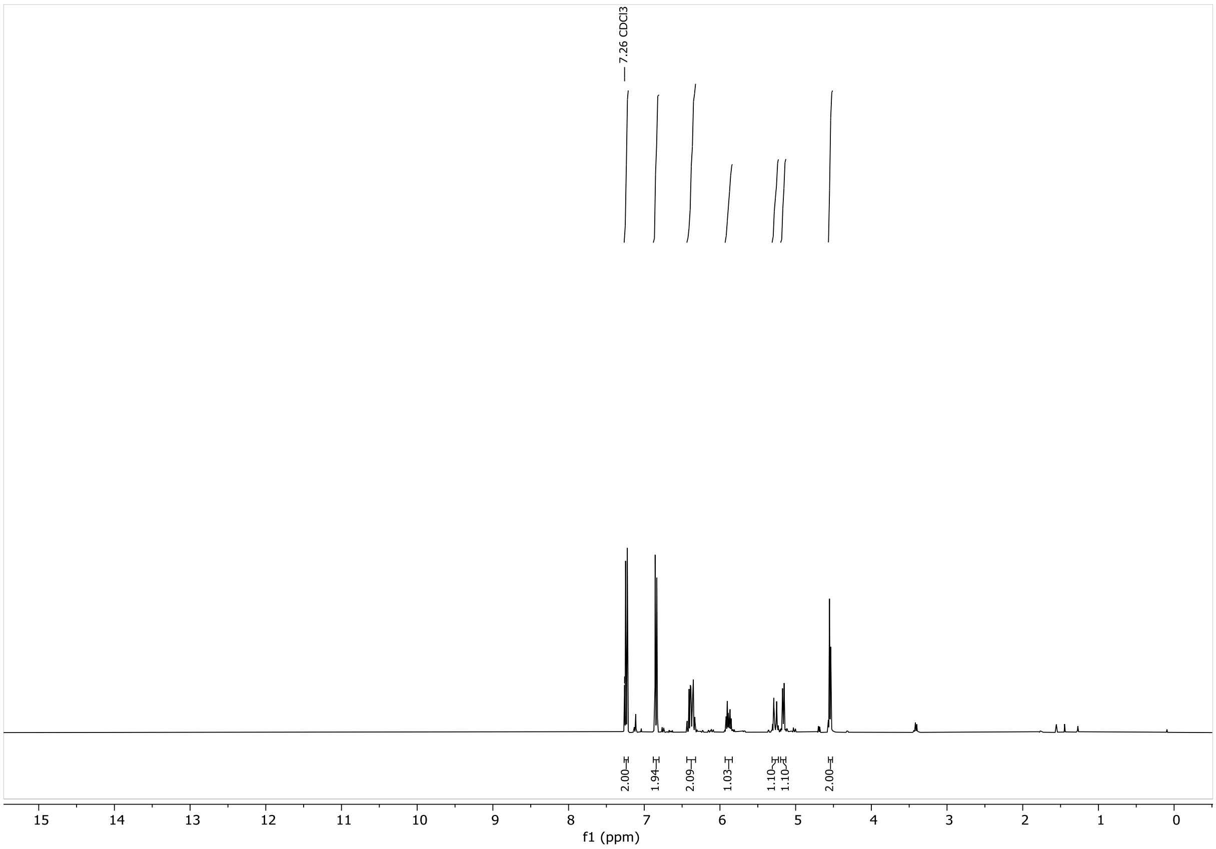

^{13}C-NMR (101 MHz, $CDCl_3$)

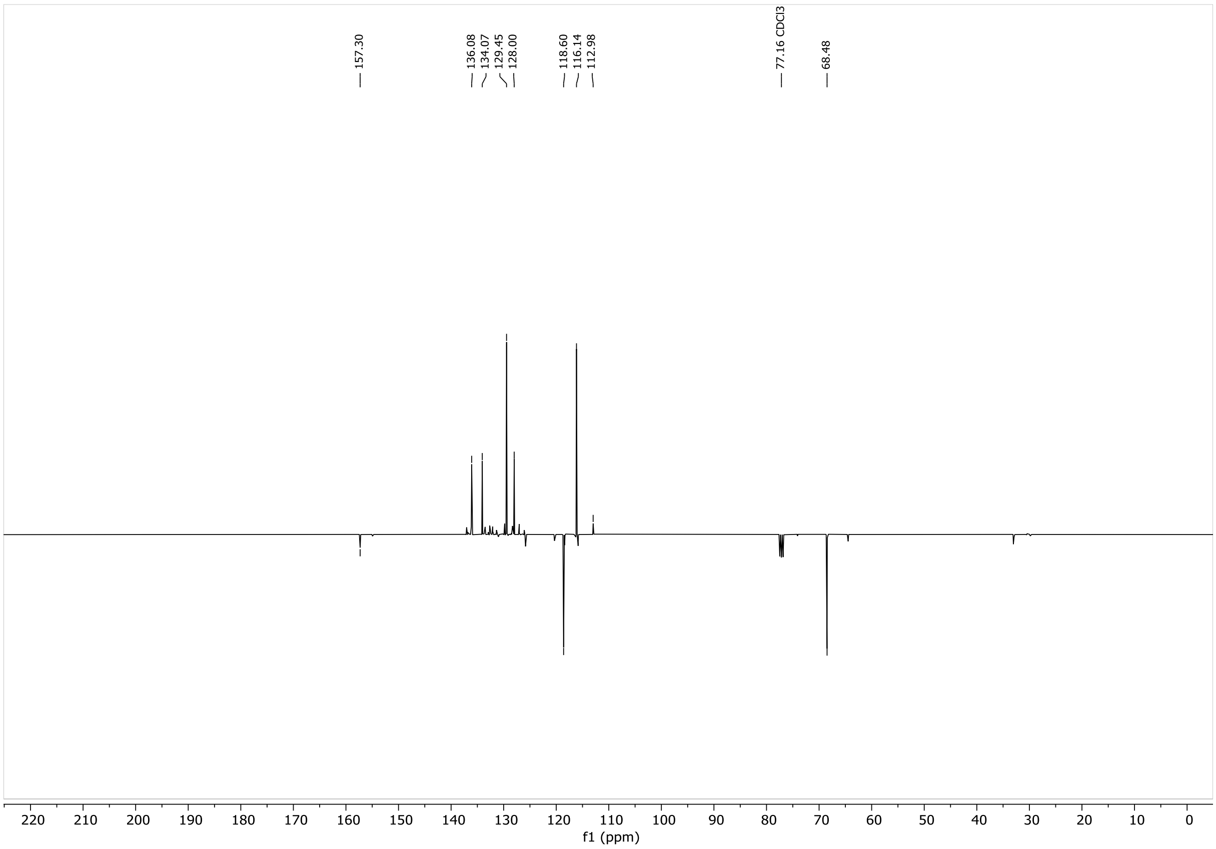

(*E*)-3-methoxy-4-(penta-2,4-dien-1-yloxy)benzaldehyde (8i)

^{1}H NMR (400 MHz, $CDCl_3$)

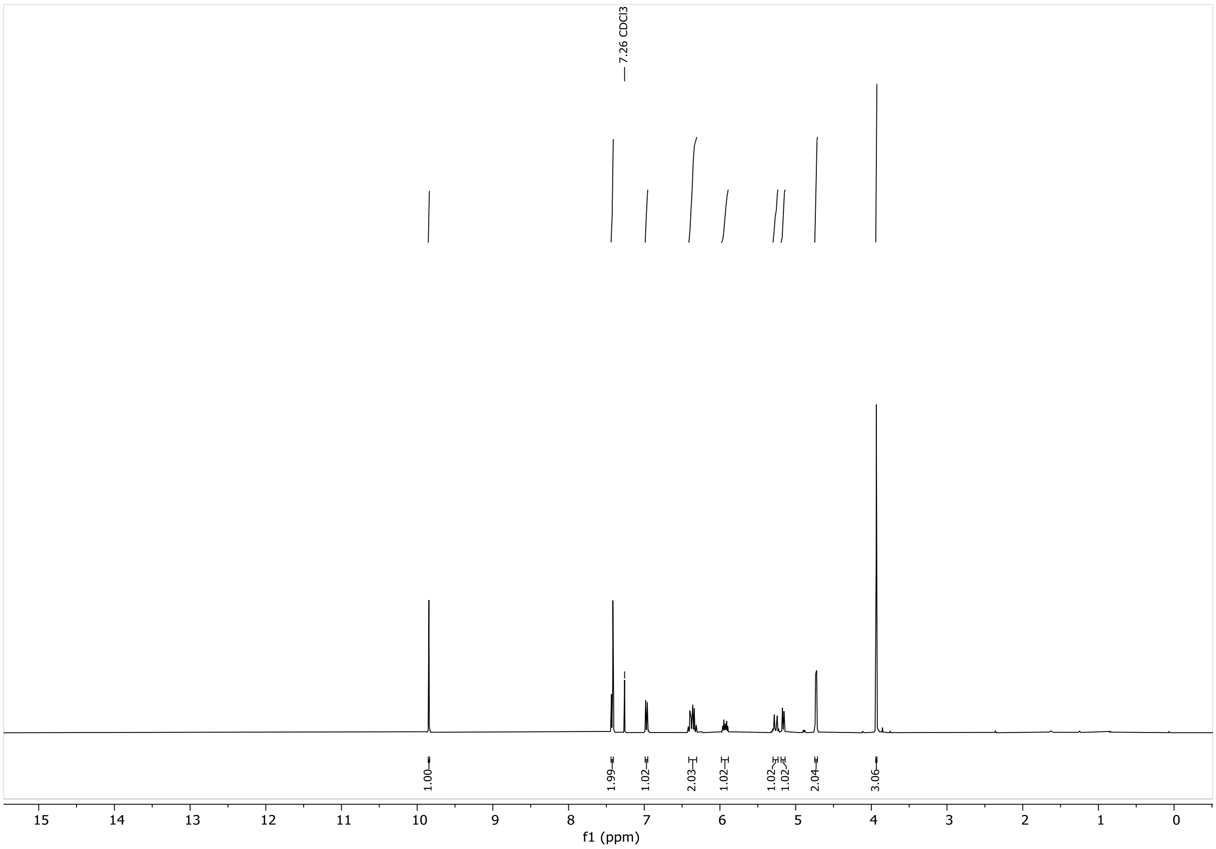

^{13}C-NMR (101 MHz, $CDCl_3$)

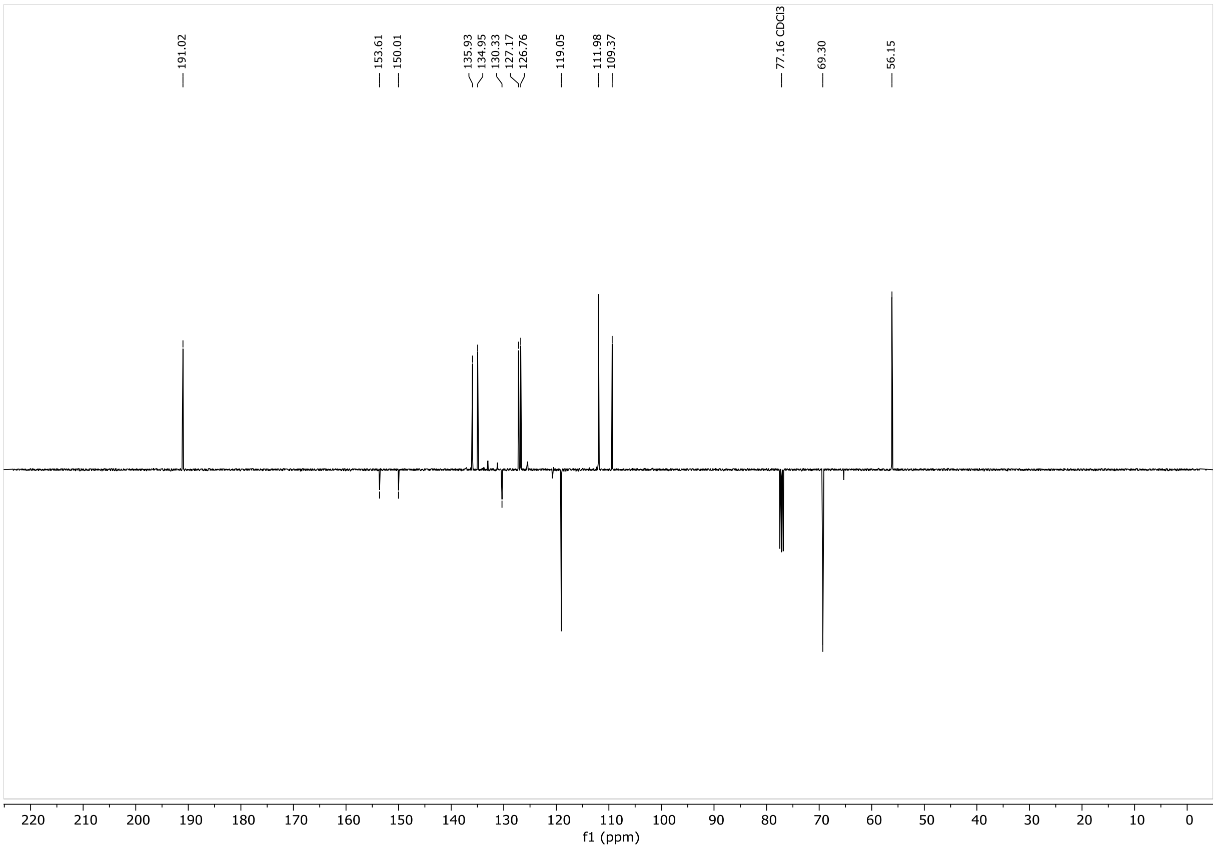

(*E*)-3-Methoxy-4-(penta-2,4-dien-1-yloxy)benzonitrile (8j)

^{1}H NMR (400 MHz, $CDCl_3$)

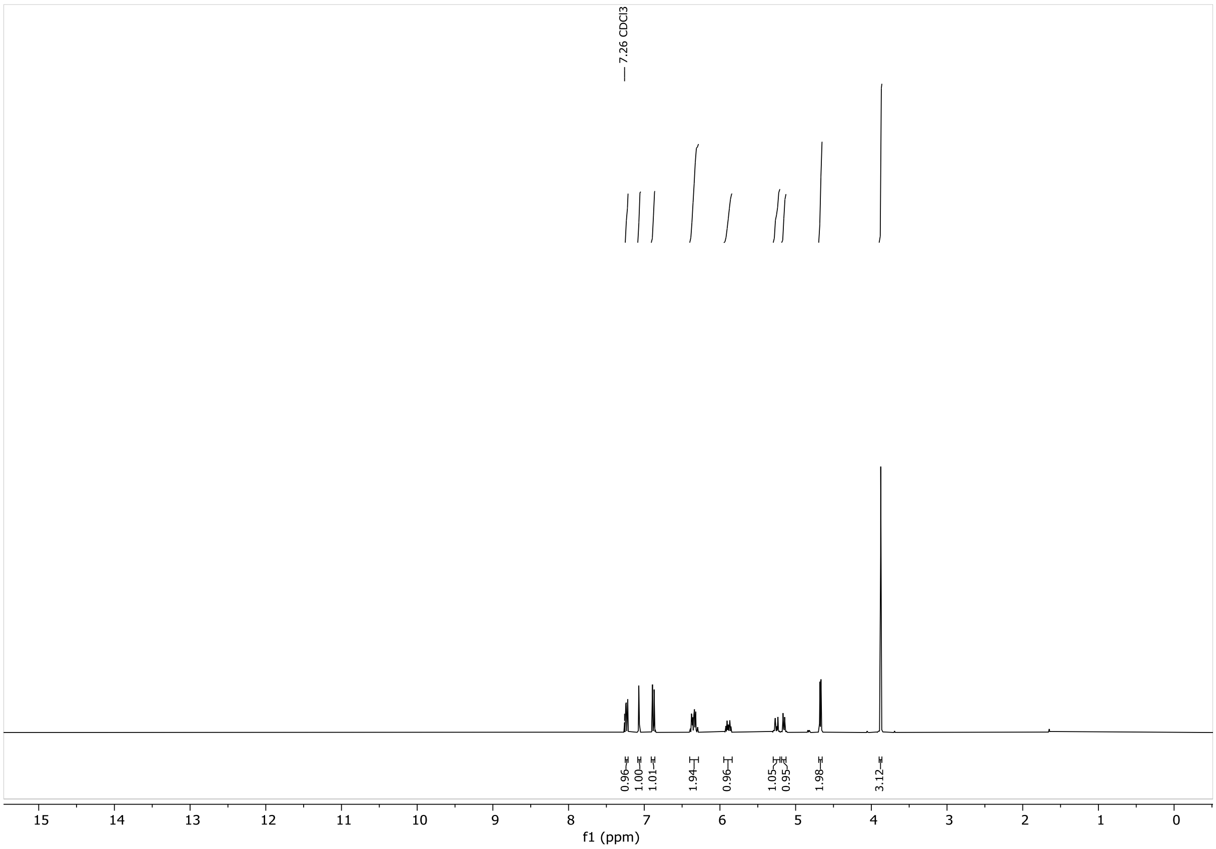

^{13}C-NMR (101 MHz, $CDCl_3$)

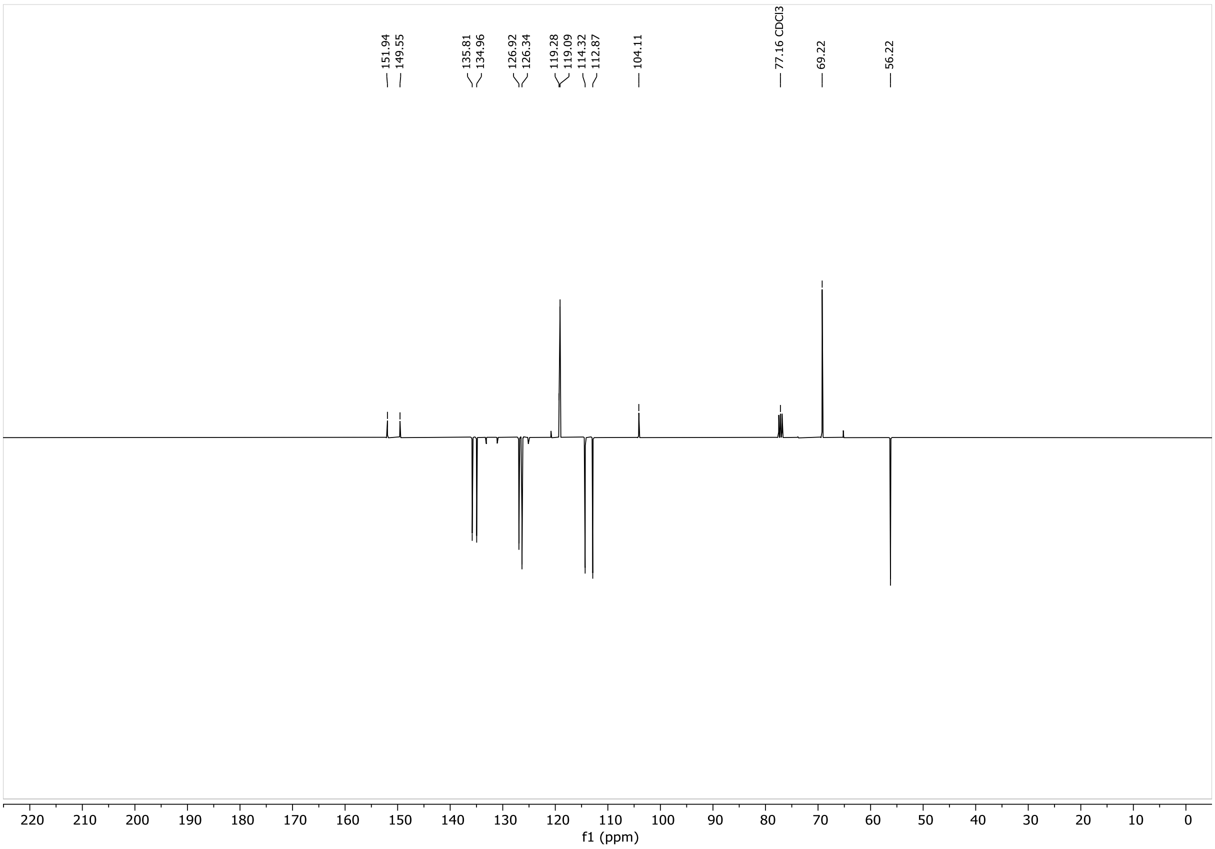

(*E*)-4-(Penta-2,4-dien-1-yloxy)benzaldehyde (8k)

^{1}H NMR (400 MHz, $CDCl_3$)

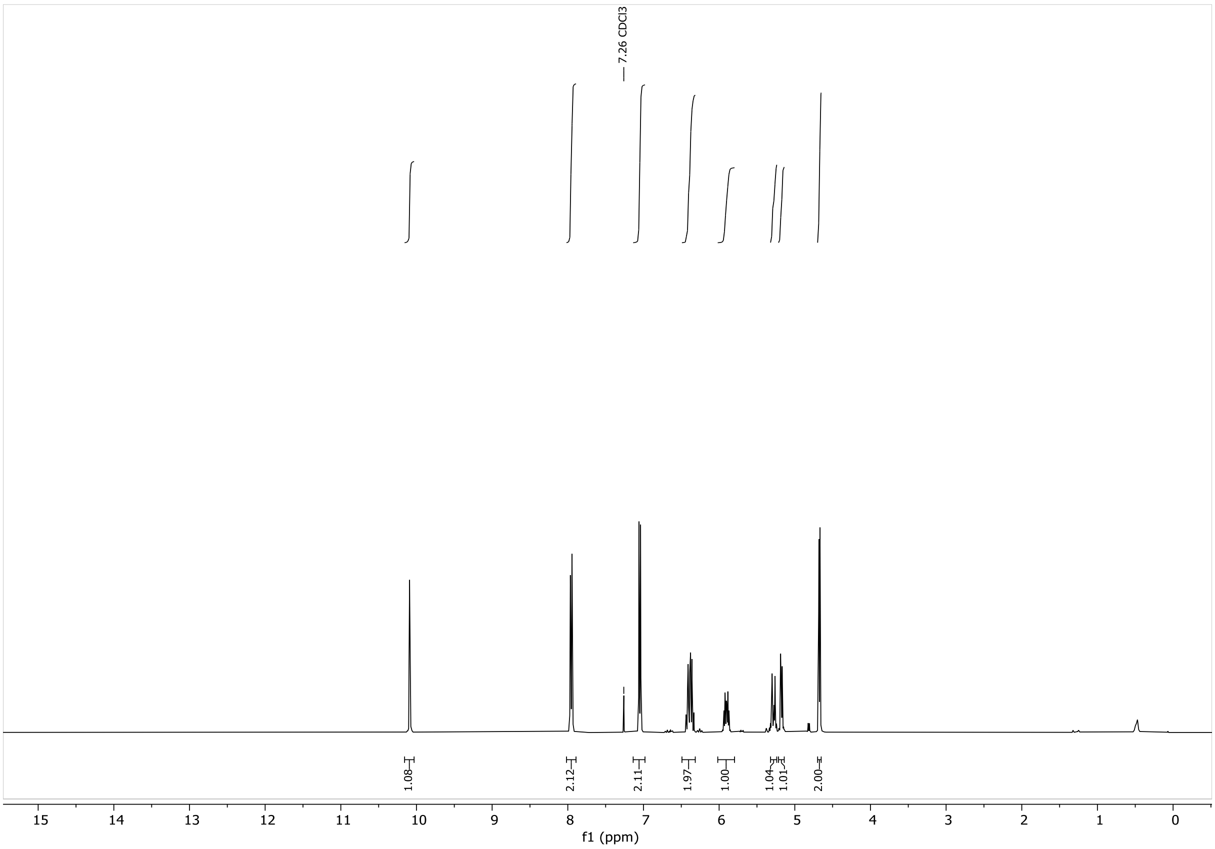

^{13}C-NMR (101 MHz, $CDCl_3$)

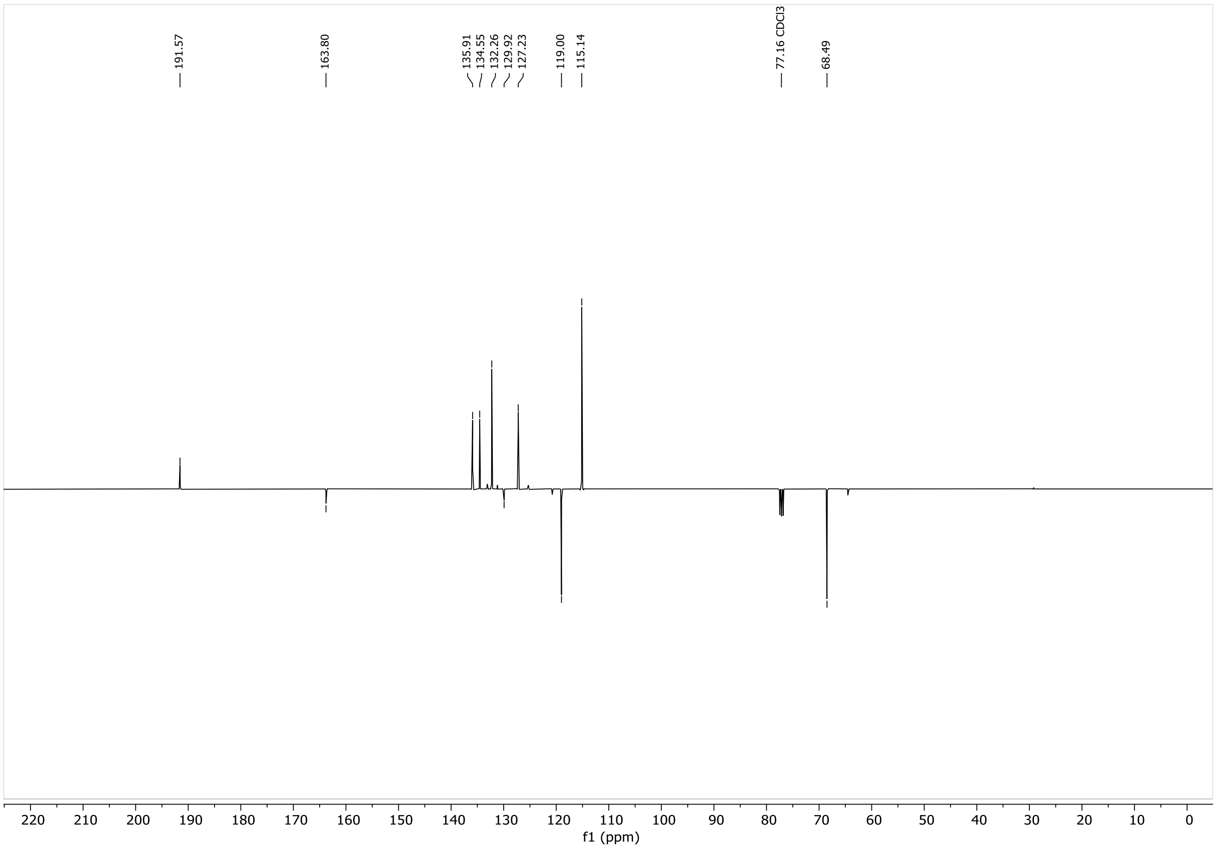

(*E*)-4-(penta-2,4-dien-1-yloxy)benzonitrile (8l)

^{1}H NMR (400 MHz, $CDCl_3$)

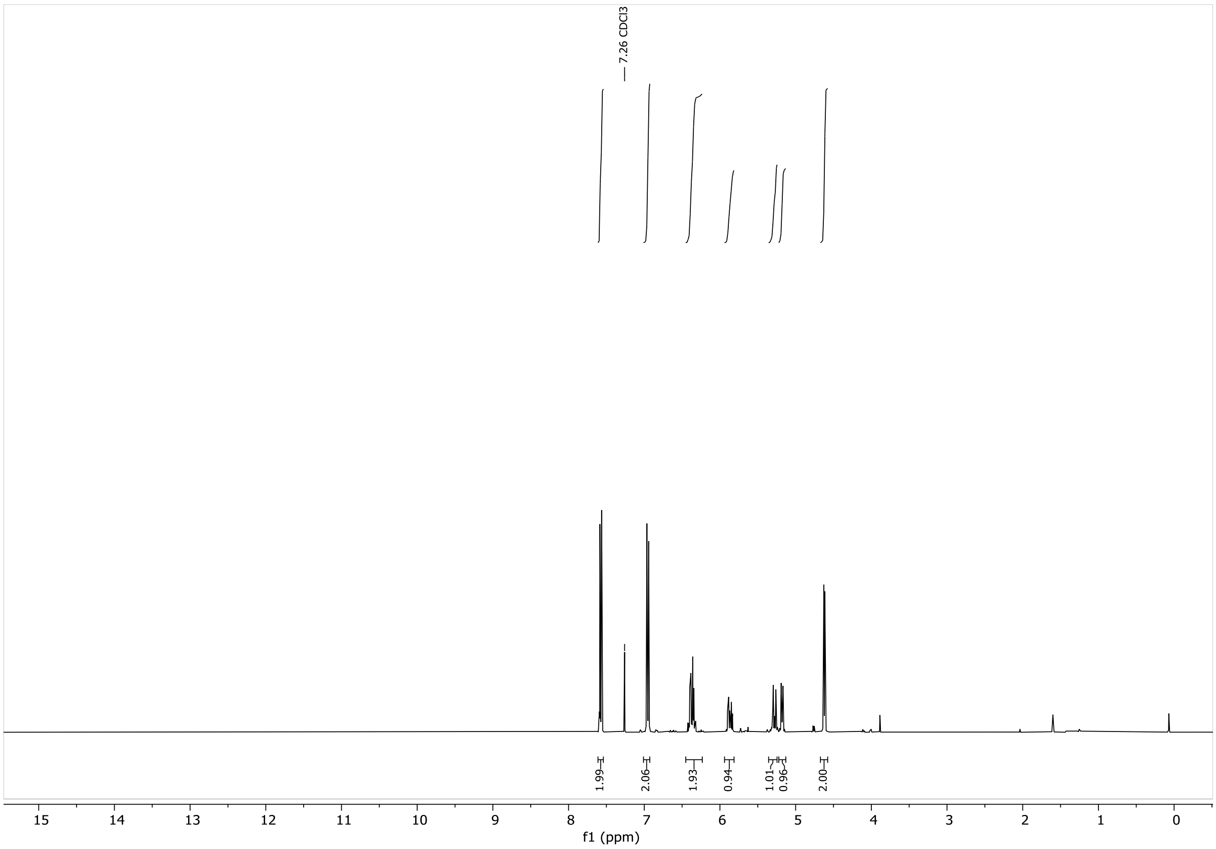

^{13}C-NMR (101 MHz, $CDCl_3$)

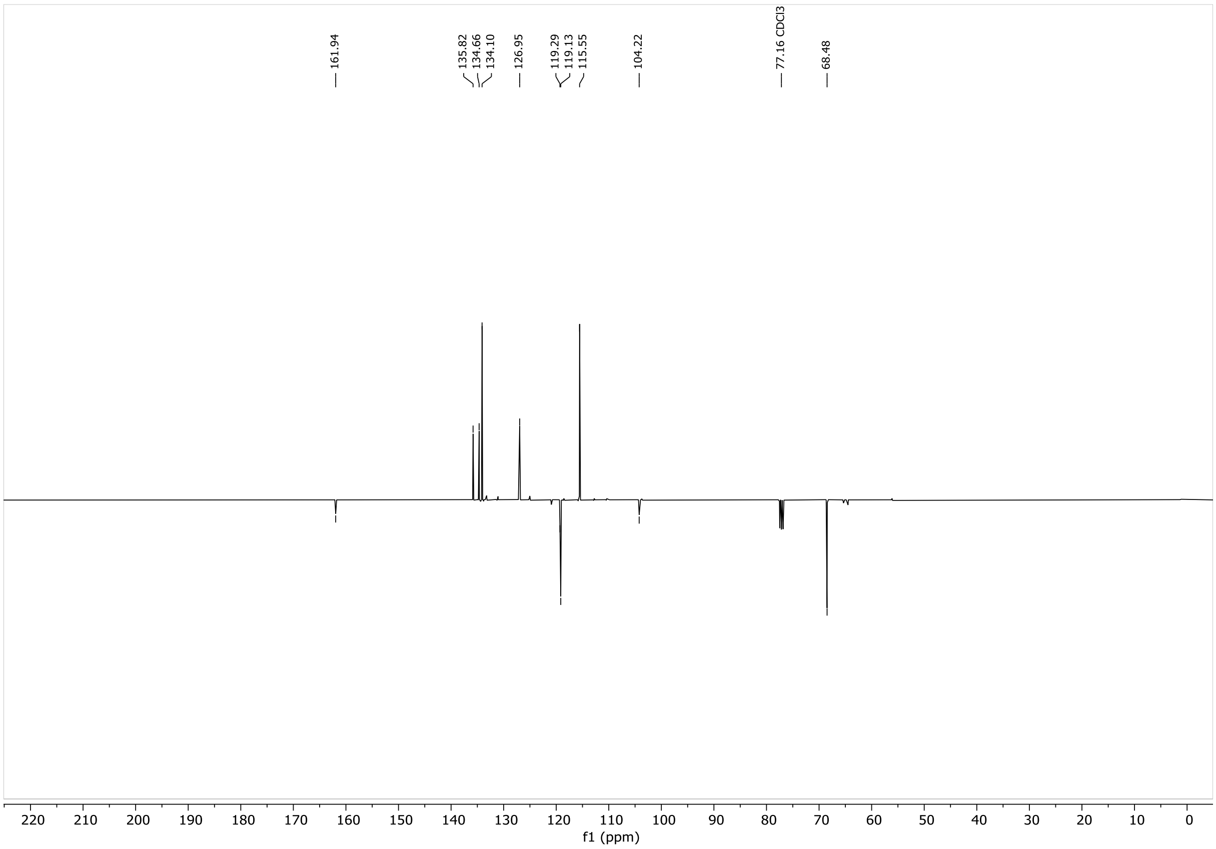

Ethyl (*E*)-4-(penta-2,4-dien-1-yloxy)benzoate (8m)

^{1}H NMR (400 MHz, $CDCl_3$)

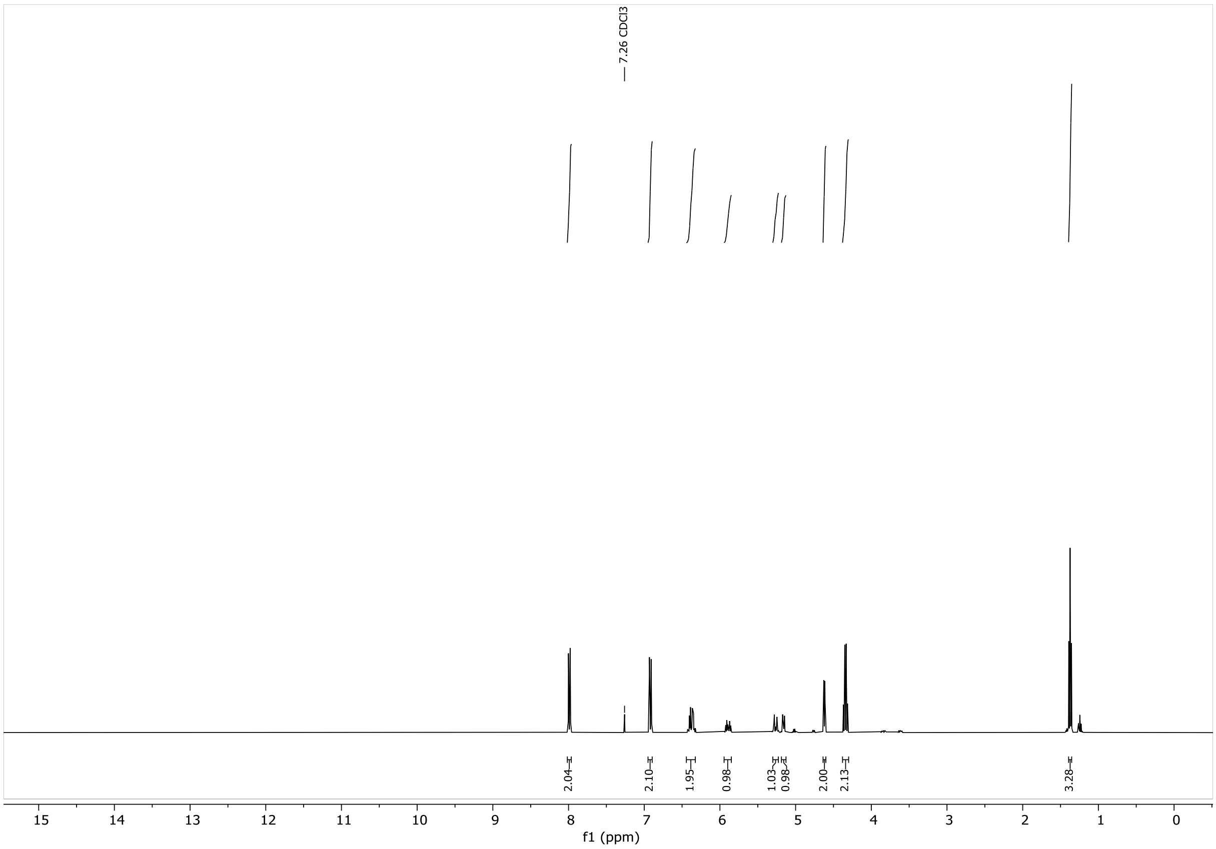

^{13}C-NMR (101 MHz, $CDCl_3$)

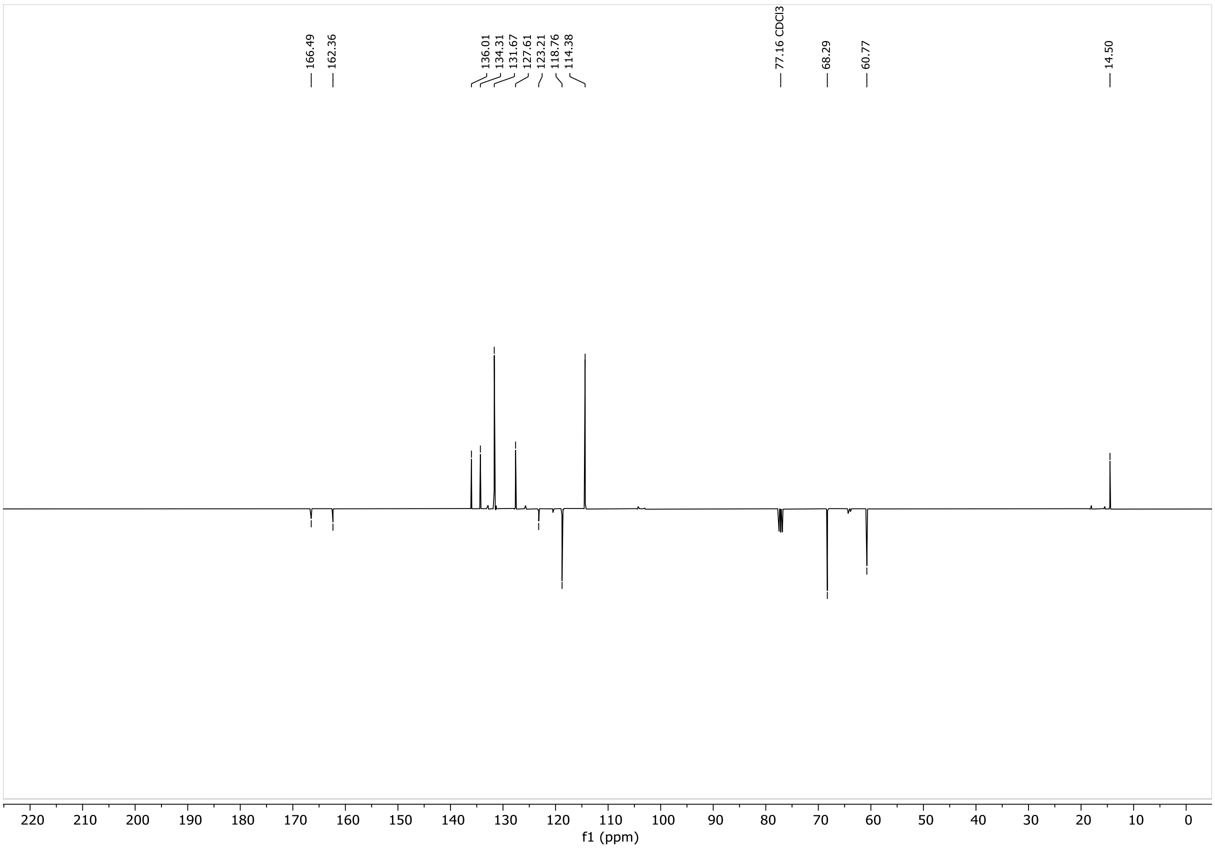

(*E*)-1-(4-(penta-2,4-dien-1-yloxy)phenyl)ethan-1-one (8n)

^{1}H NMR (400 MHz, $CDCl_3$)

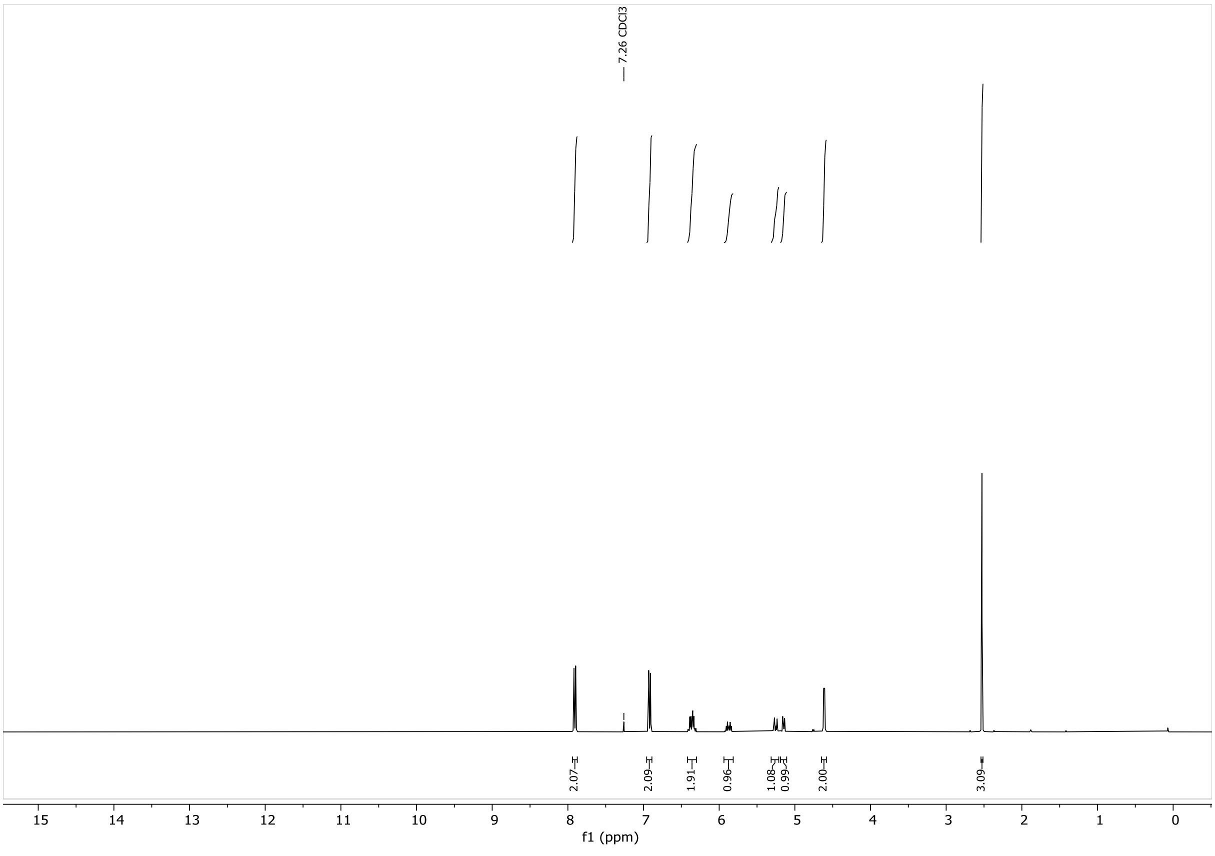

^{13}C-NMR (101 MHz, $CDCl_3$)

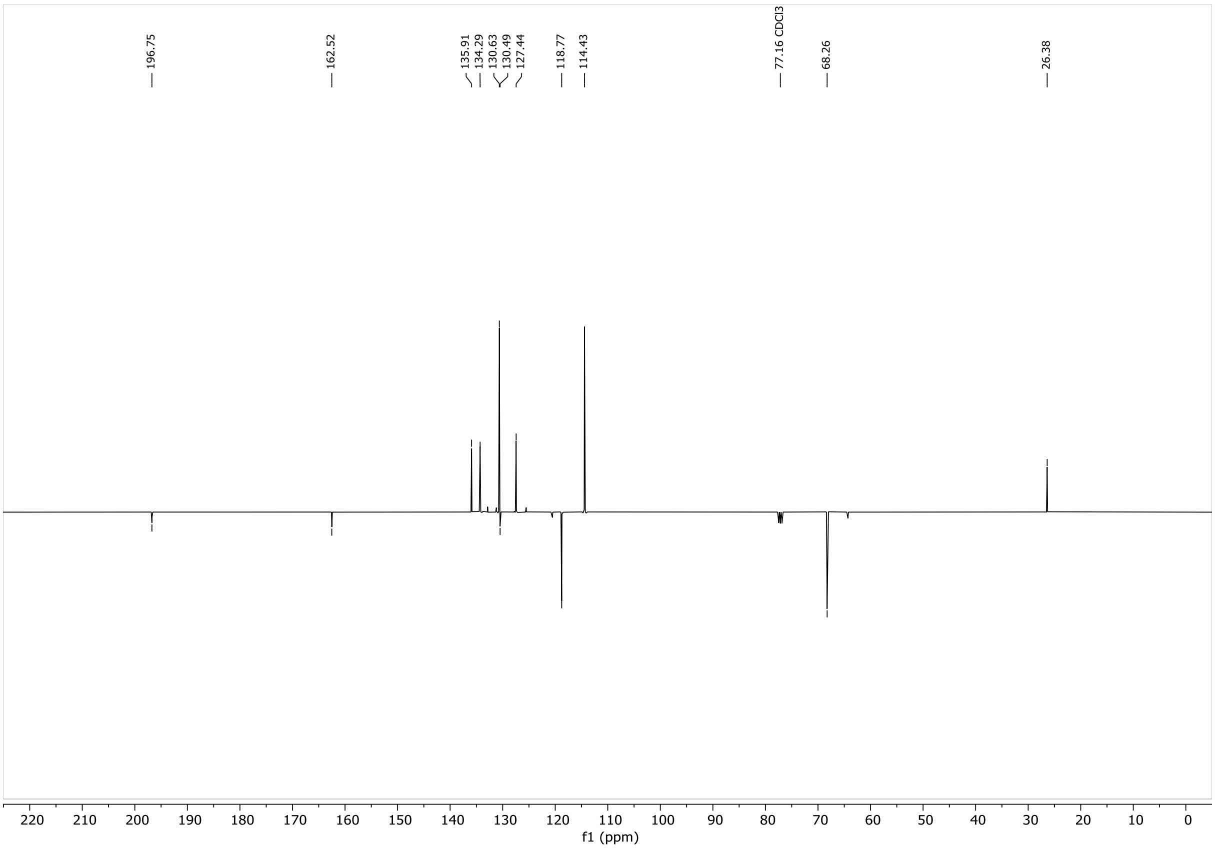

(*E*)-1-nitro-4-(penta-2,4-dien-1-yloxy)benzene (8o)

^{1}H NMR (400 MHz, $CDCl_3$)

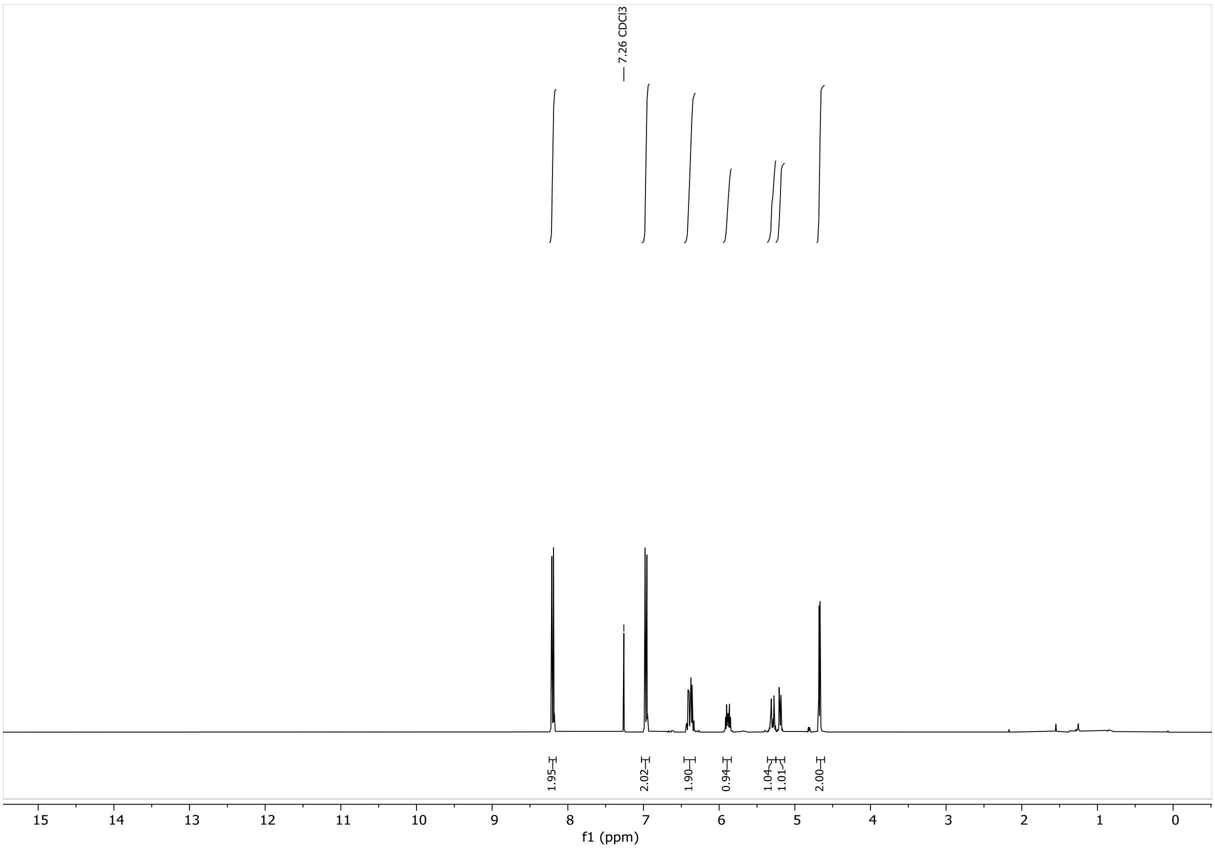

^{13}C-NMR (101 MHz, $CDCl_3$)

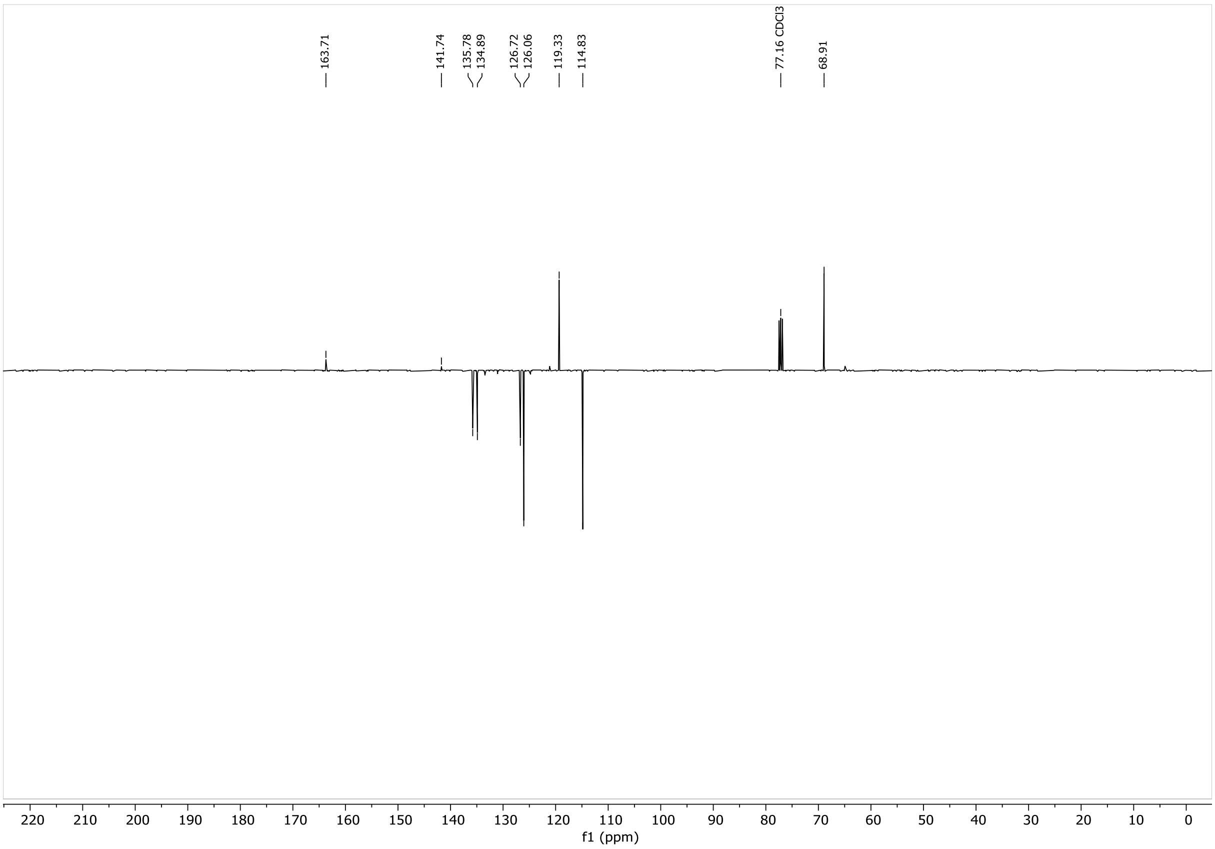

(*E*)-1-Iodo-4-(penta-2,4-dien-1-yloxy)benzene (8p)

^{1}H NMR (400 MHz, $CDCl_3$)

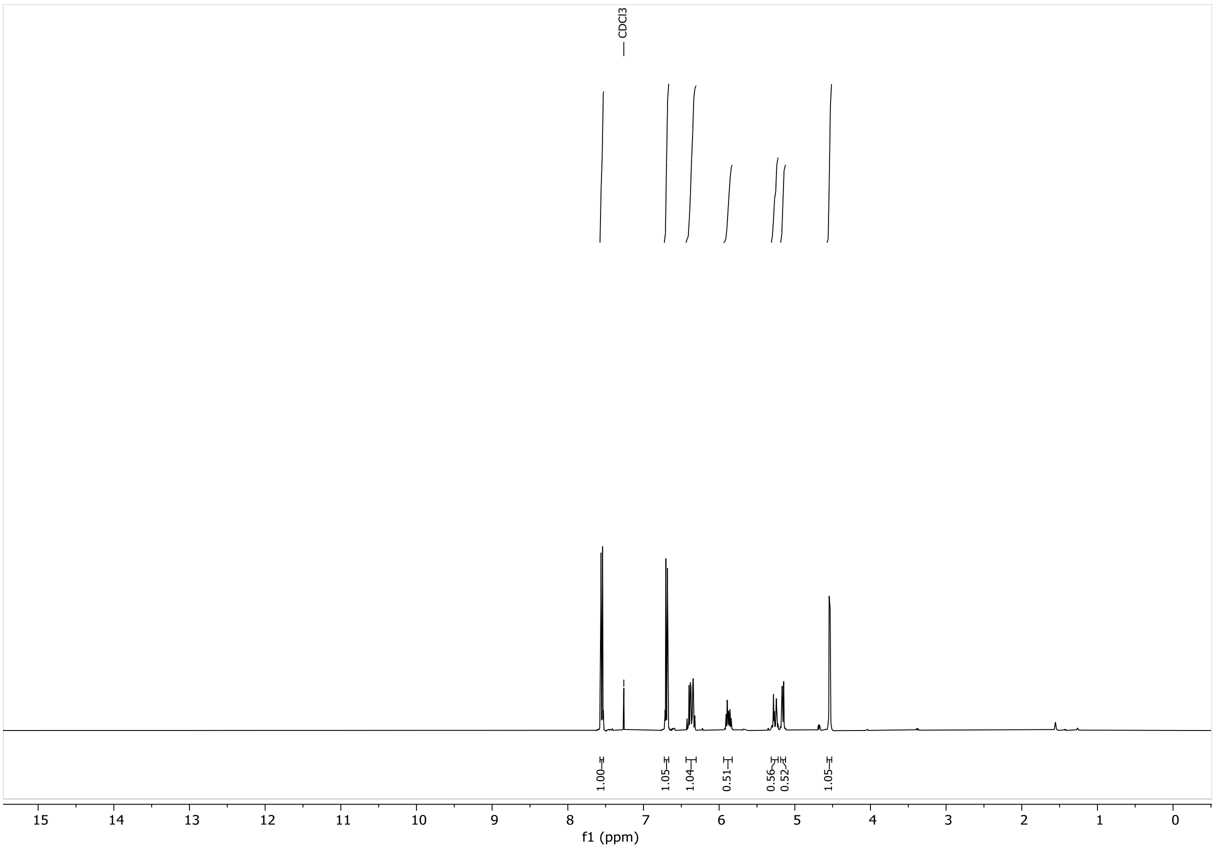

^{13}C-NMR (151 MHz, $CDCl_3$)

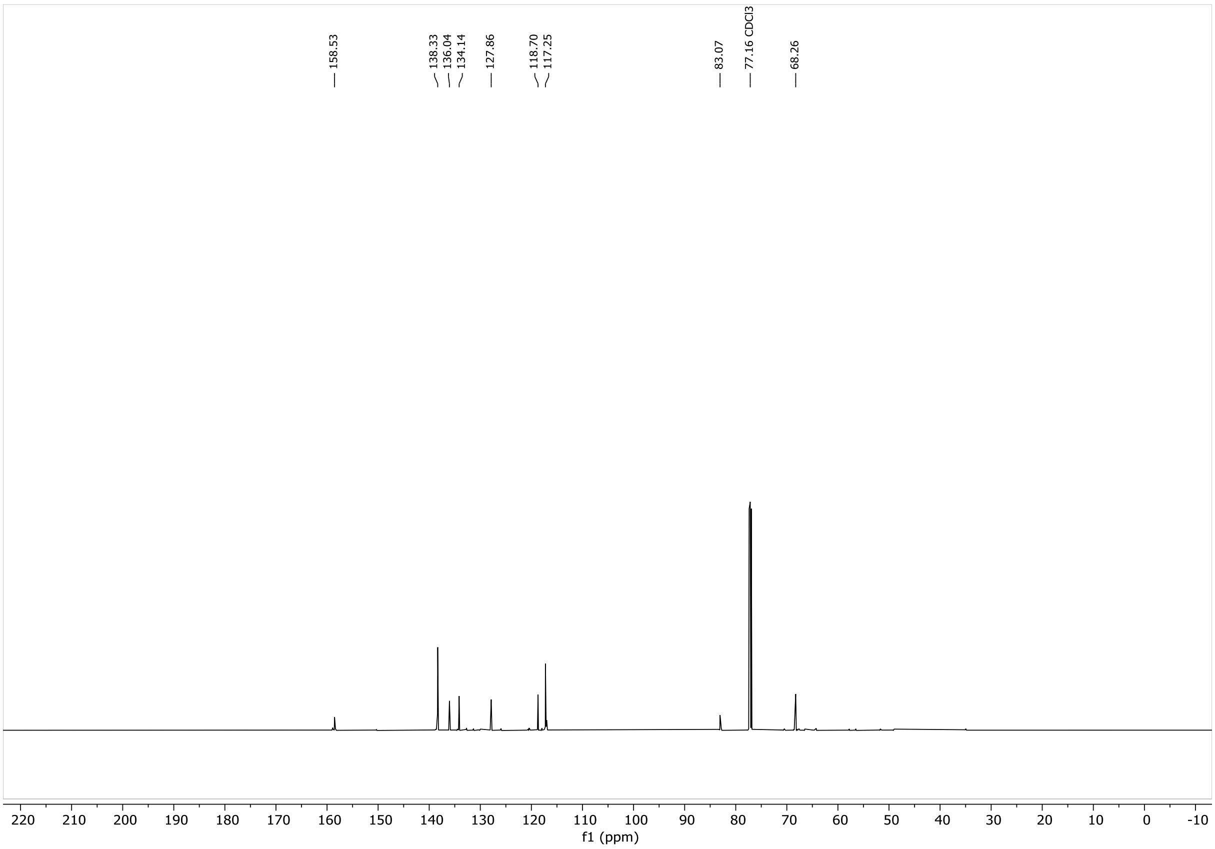

(*E*)-4-iodo-2-methoxy-1-(penta-2,4-dien-1-yloxy)benzene (8q)

^{1}H NMR (400 MHz, $CDCl_3$)

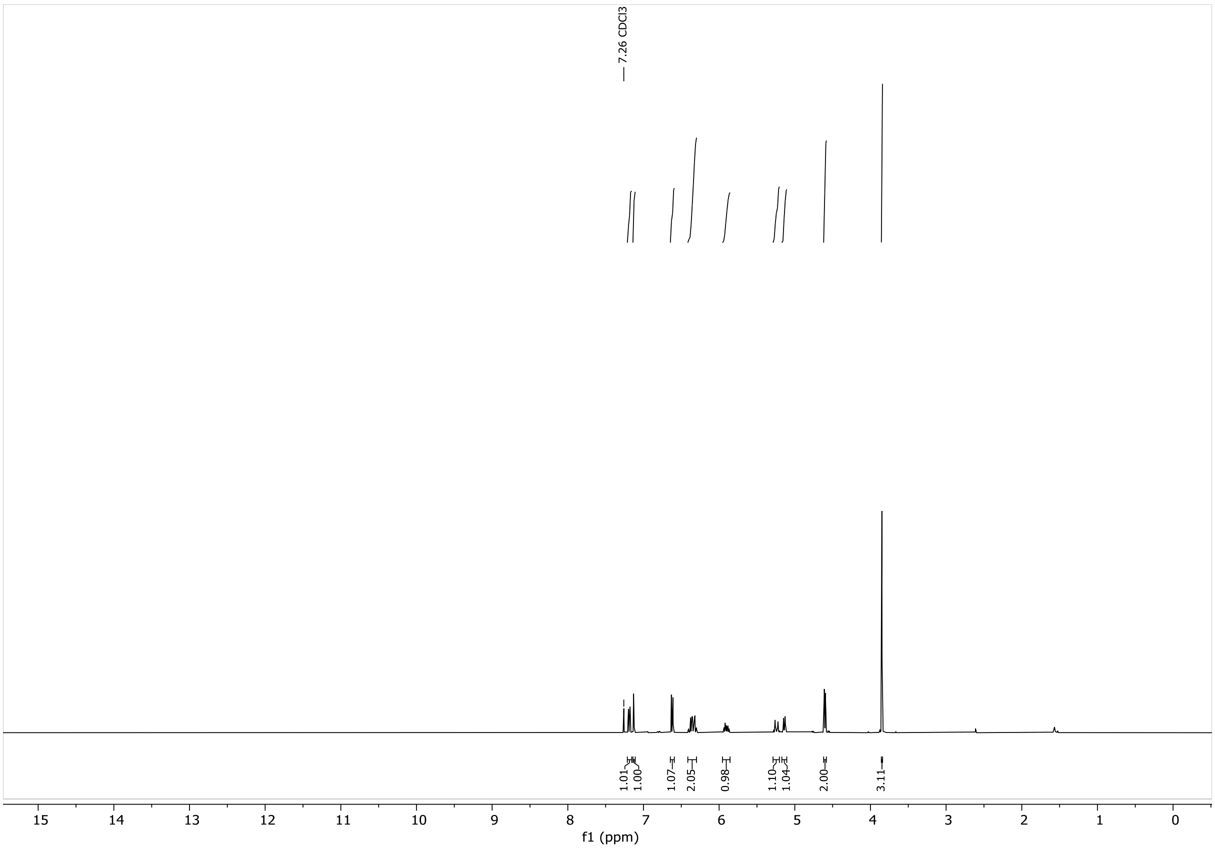

^{13}C-NMR (101 MHz, $CDCl_3$)

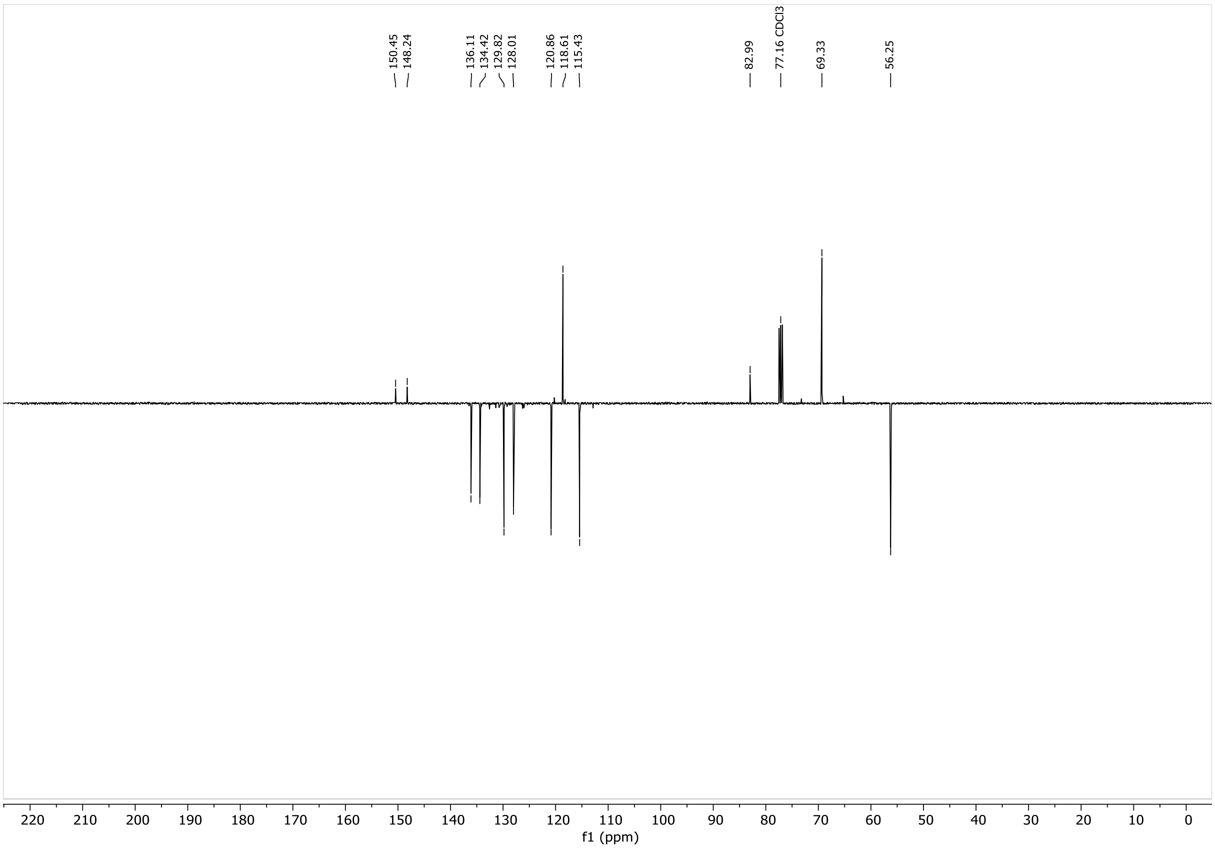

(*E*)-(penta-2,4-dien-1-yloxy)benzene (10a)

^{1}H NMR (400 MHz, $CDCl_3$)

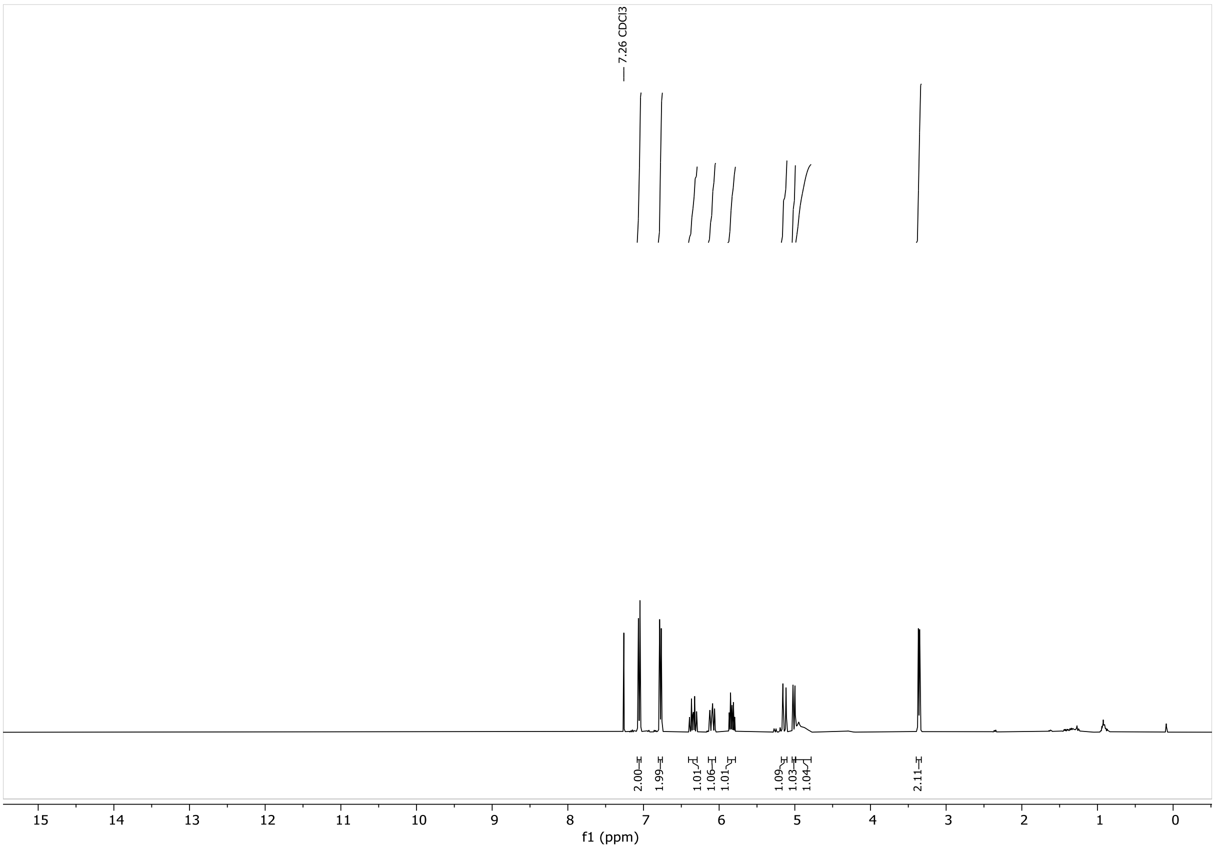

^{13}C-NMR (101 MHz, $CDCl_3$)

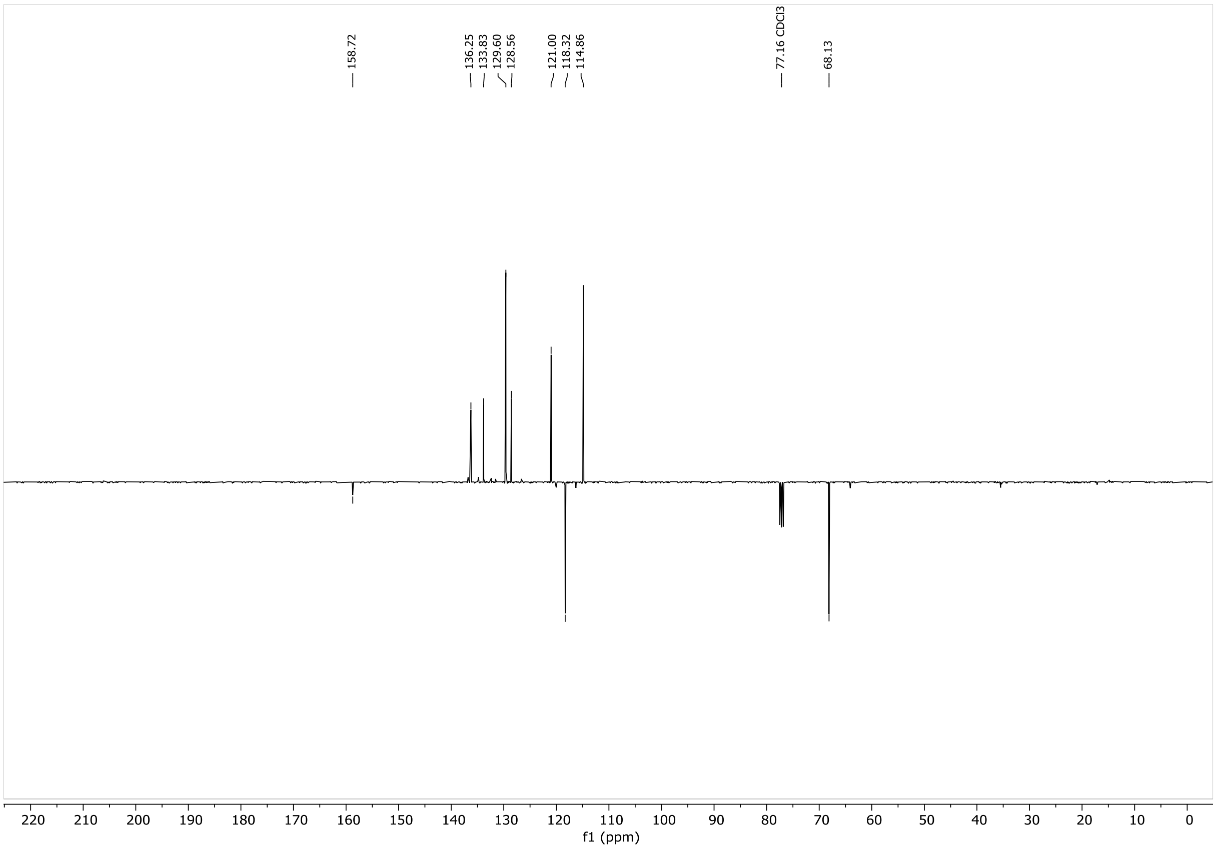

(*E*)-1-methyl-2-(penta-2,4-dien-1-yloxy)benzene (10b)

^{1}H NMR (400 MHz, $CDCl_3$)

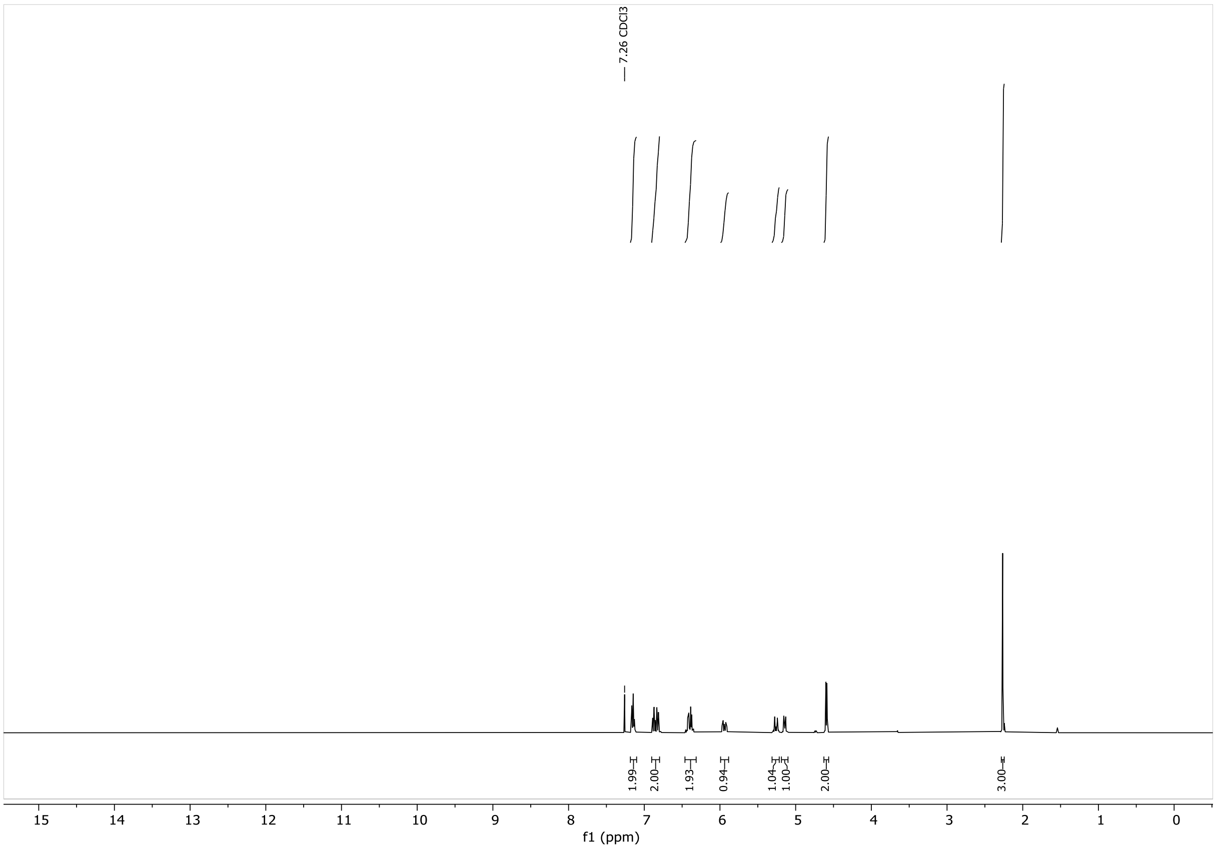

^{13}C-NMR (101 MHz, $CDCl_3$)

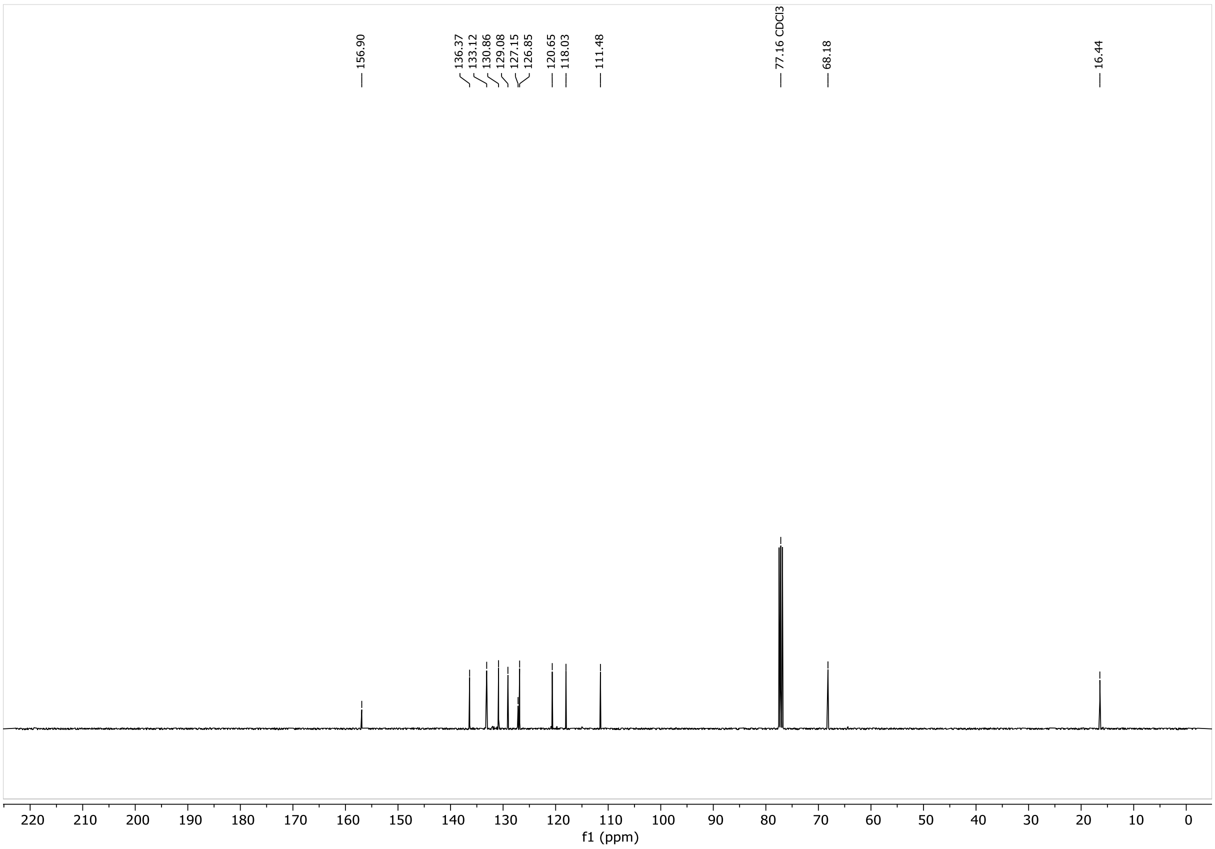

(*E*)-1-methyl-3-(penta-2,4-dien-1-yloxy)benzene (10c)

^{1}H NMR (600 MHz, $CDCl_3$)

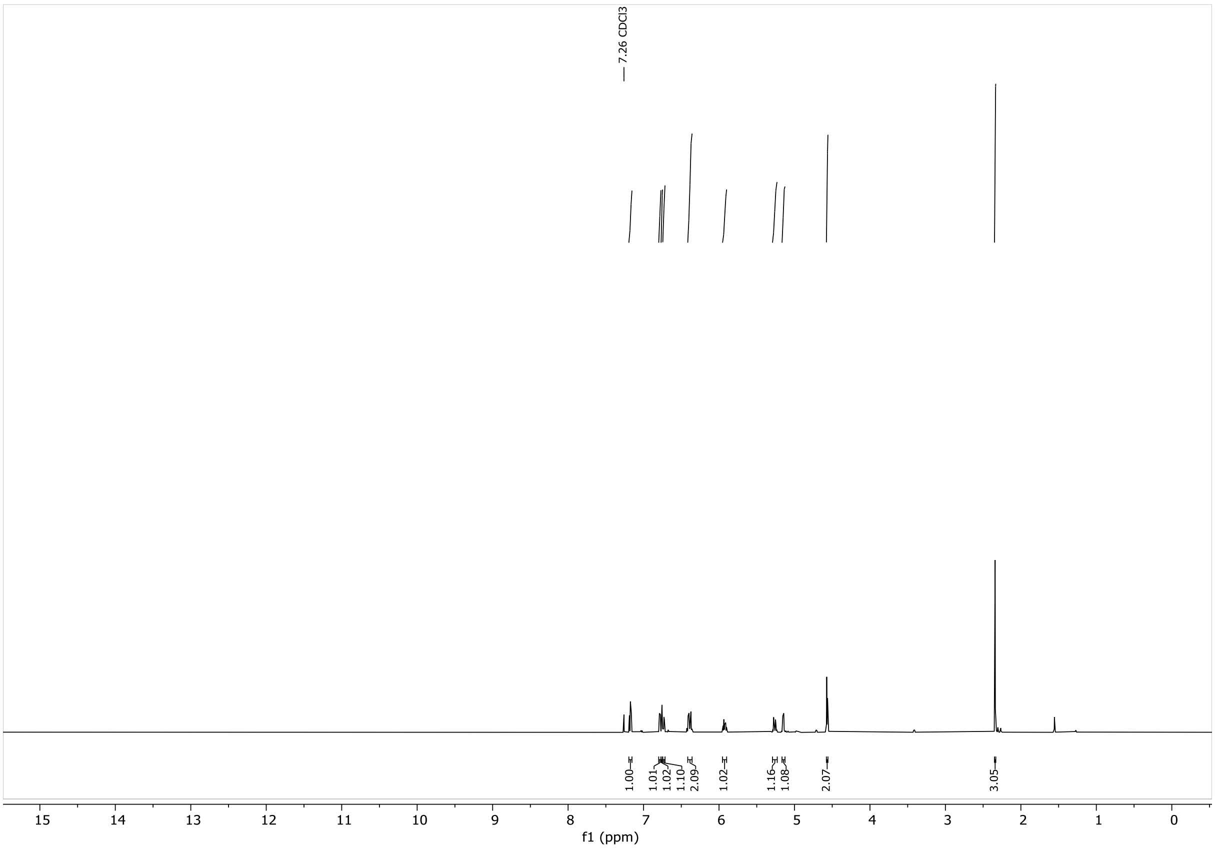

^{13}C-NMR (151 MHz, $CDCl_3$)

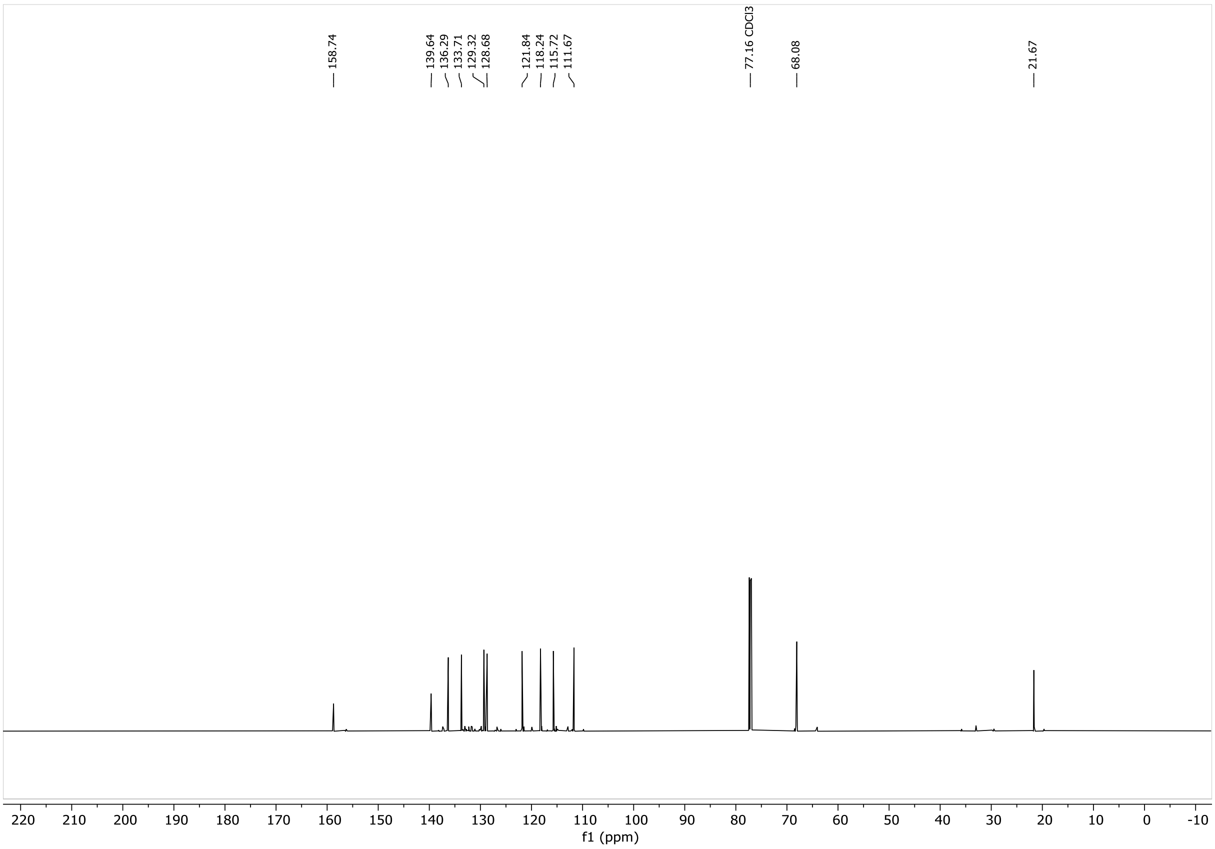

(*E*)-1-isopropyl-4-methyl-2-(penta-2,4-dien-1-yloxy)benzene (10d)

^{1}H NMR (400 MHz, $CDCl_3$)

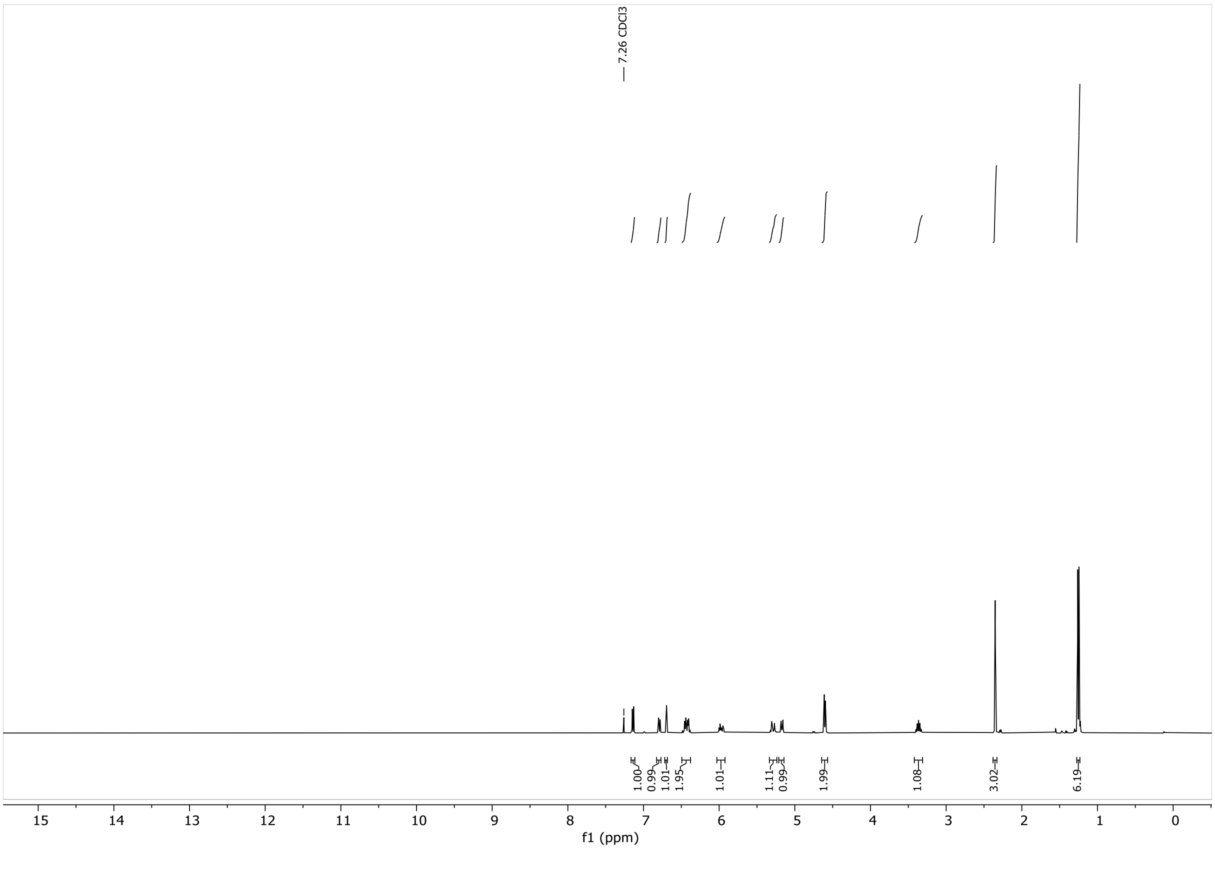

^{13}C-NMR (101 MHz, $CDCl_3$)

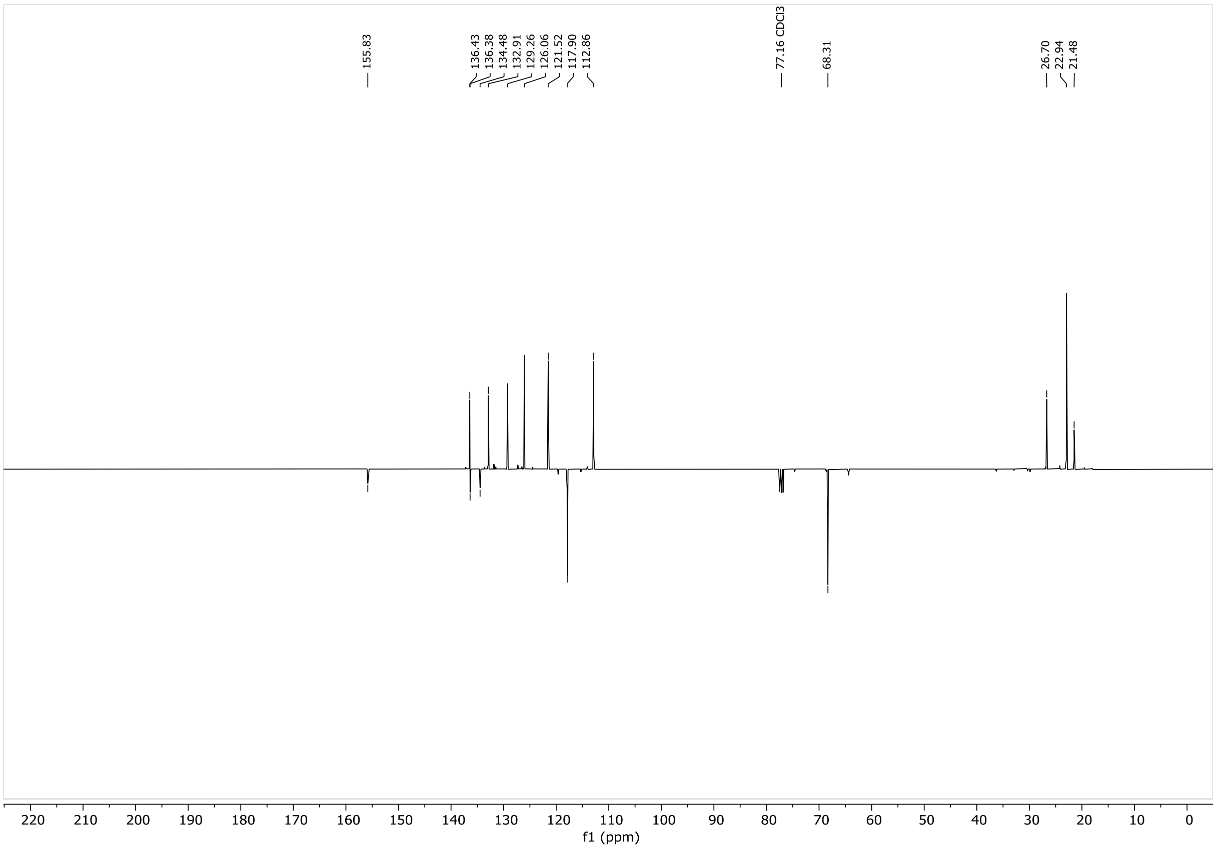

(*E*)-1,3-Dimethyl-2-(penta-2,4-dien-1-yloxy)benzene (10e)

^{1}H NMR (400 MHz, $CDCl_3$)

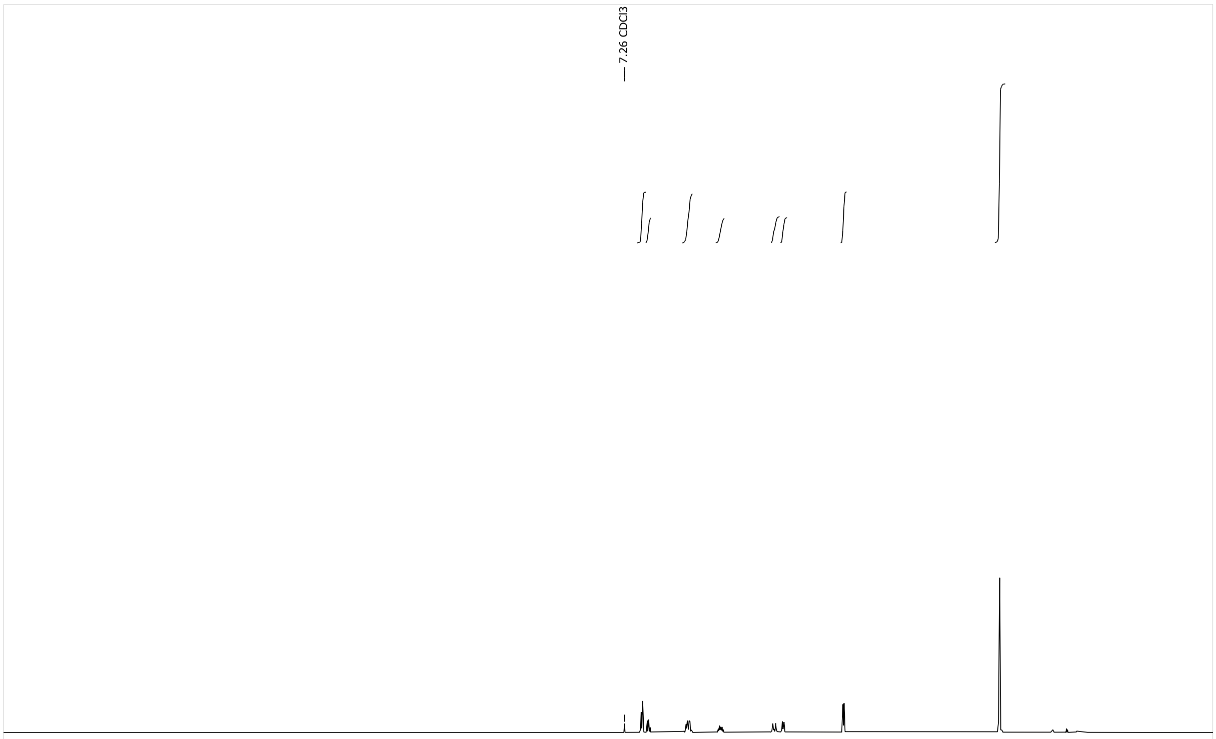

^{13}C-NMR (101 MHz, $CDCl_3$)

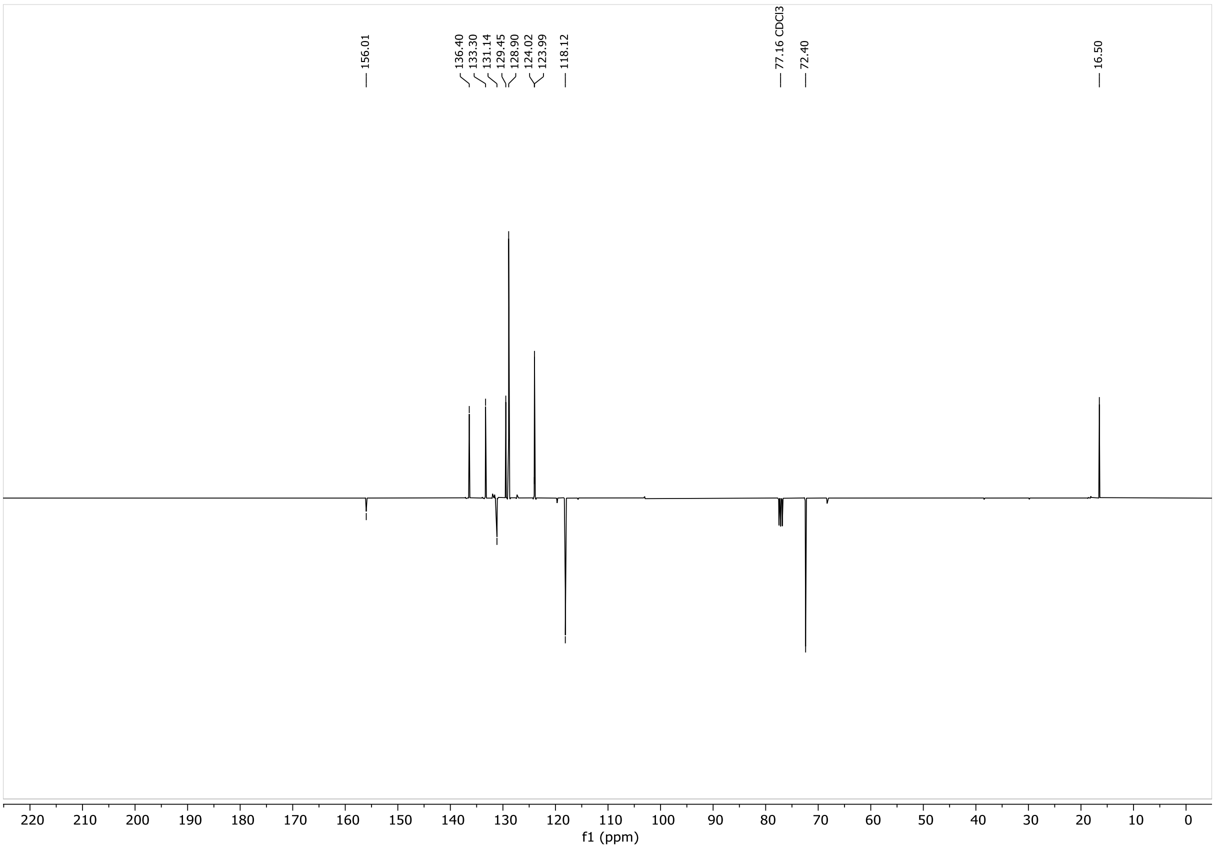

(*E*)-1-methoxy-2-(penta-2,4-dien-1-yloxy)benzene (10f)

^{1}H NMR (400 MHz, $CDCl_3$)

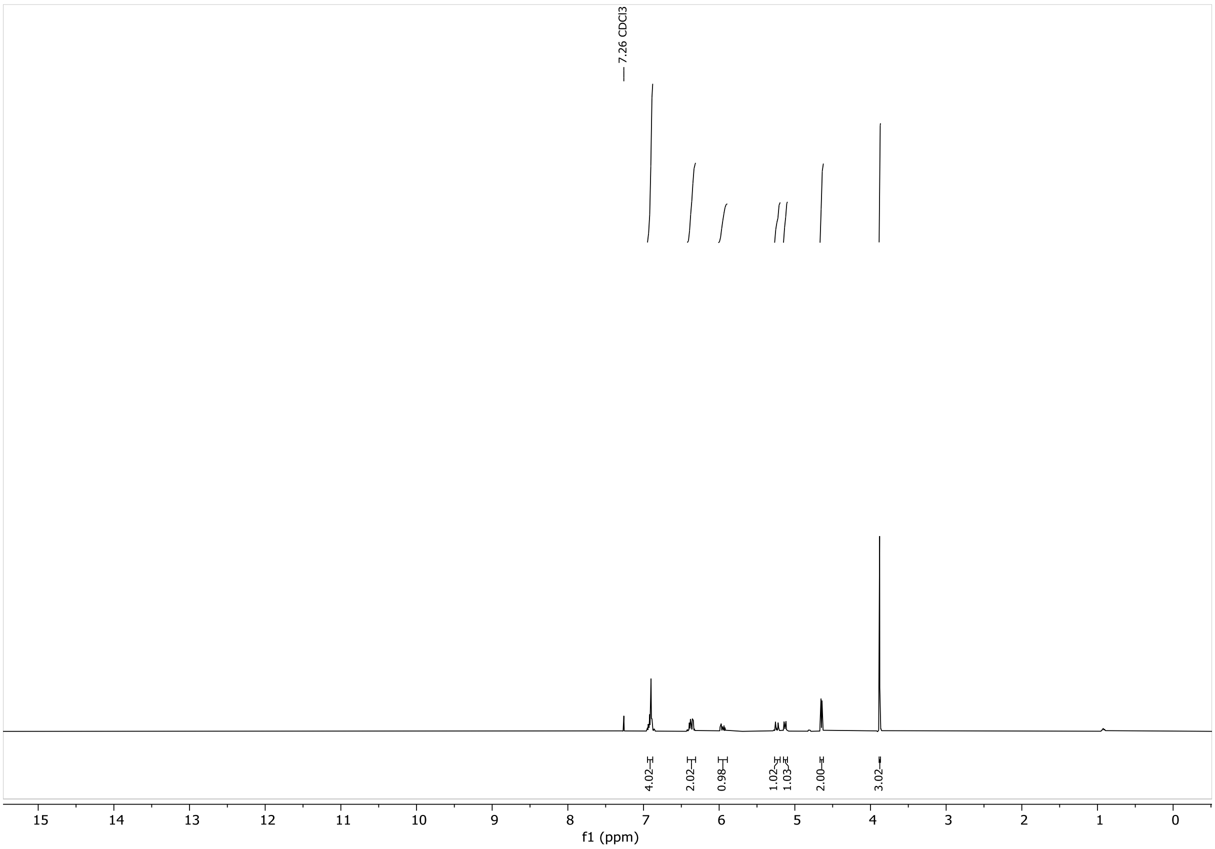

^{13}C-NMR (101 MHz, $CDCl_3$)

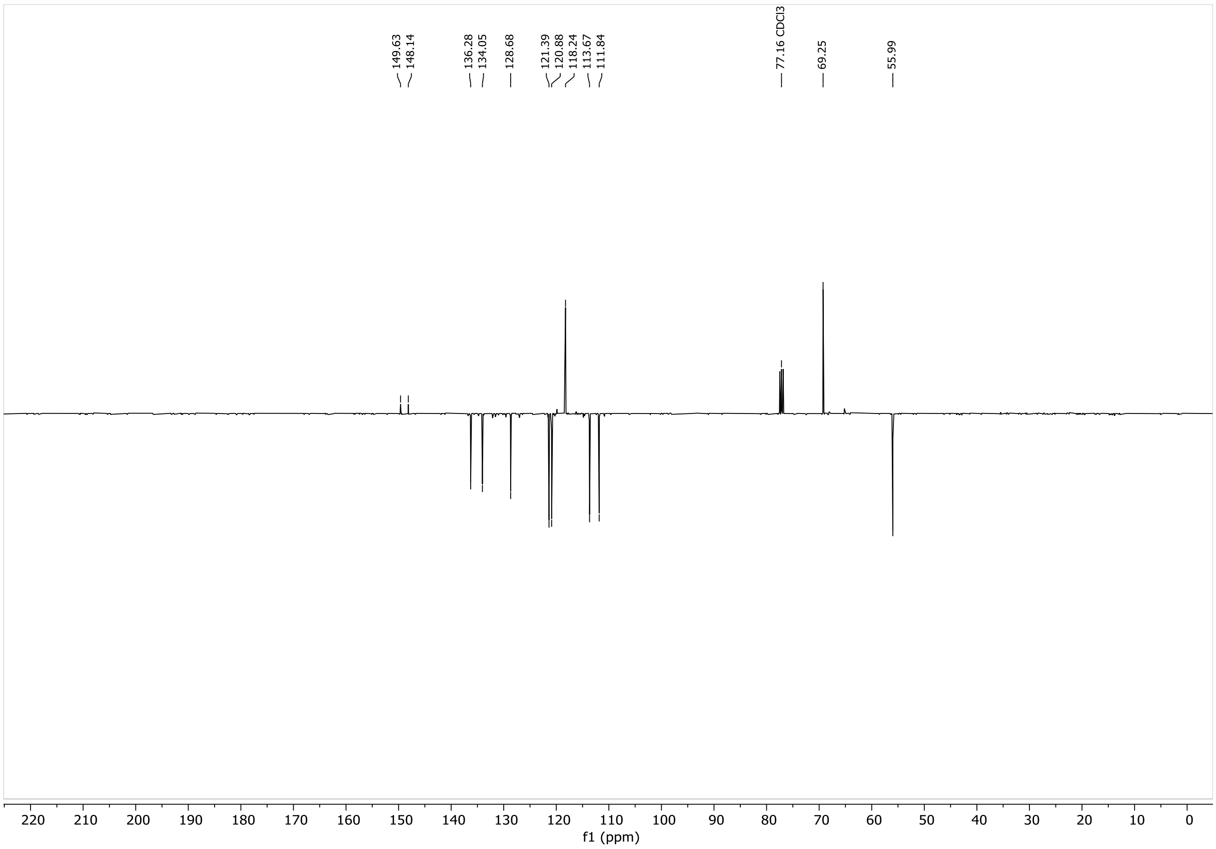

(*E*)-1-methoxy-3-(penta-2,4-dien-1-yloxy)benzene (10g)

^{1}H NMR (400 MHz, $CDCl_3$)

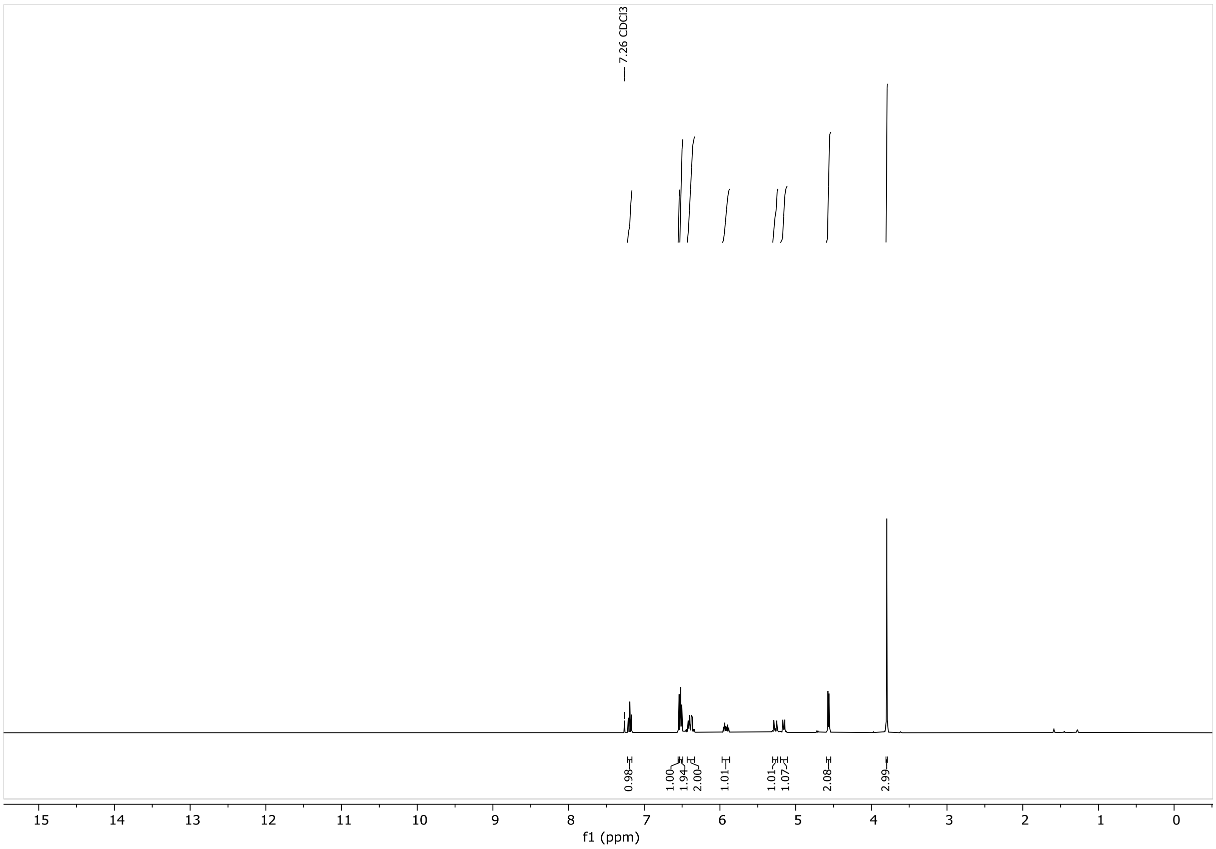

^{13}C-NMR (101 MHz, $CDCl_3$)

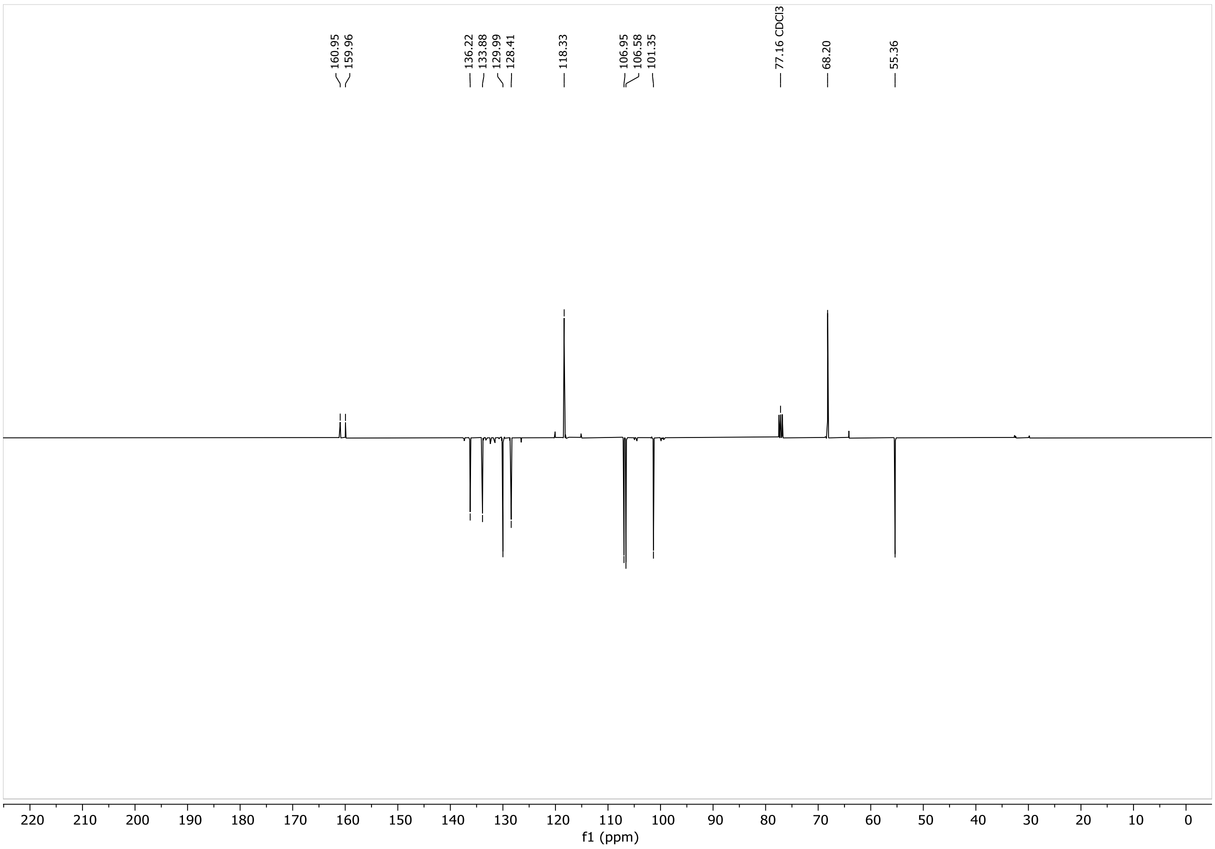

(*E*)-triisopropyl(3-(penta-2,4-dien-1-yloxy)phenoxy)silane (10h)

^{1}H NMR (400 MHz, $CDCl_3$)

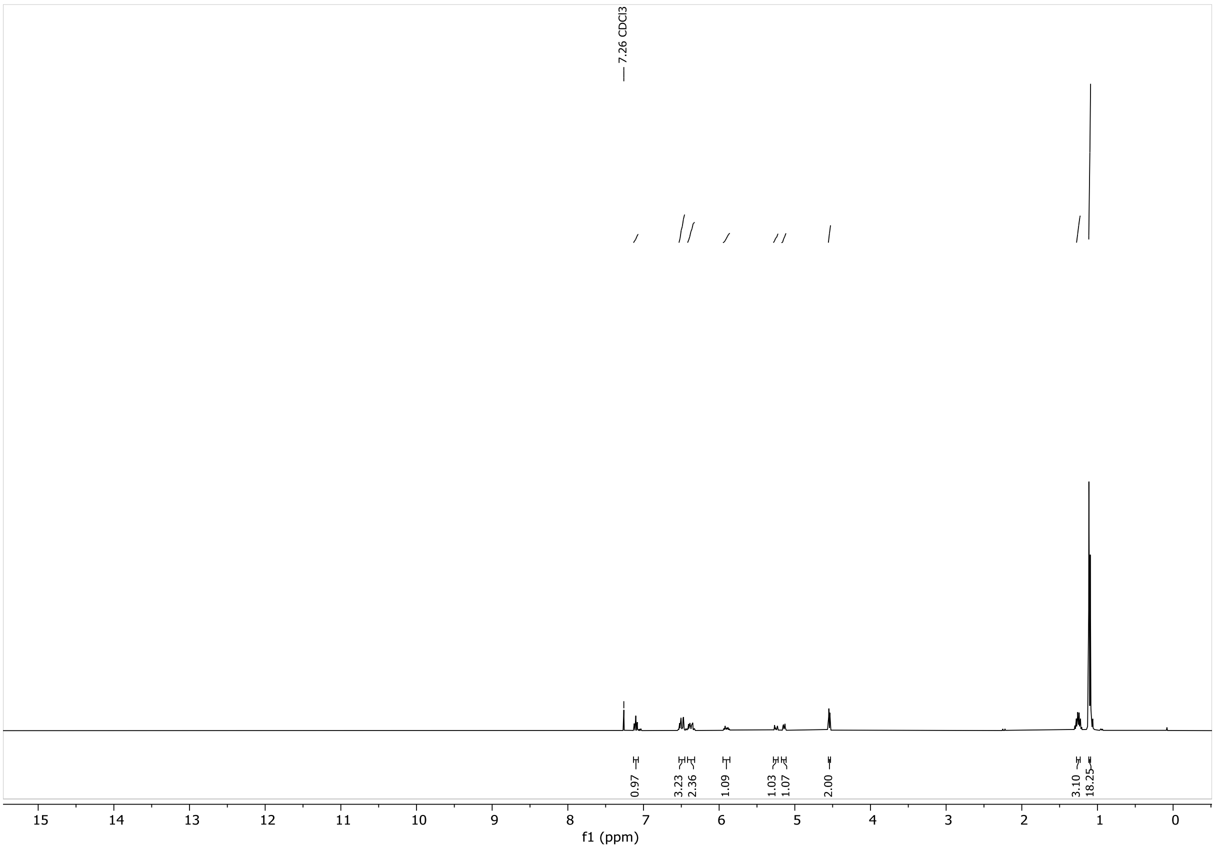

^{13}C-NMR (101 MHz, $CDCl_3$)

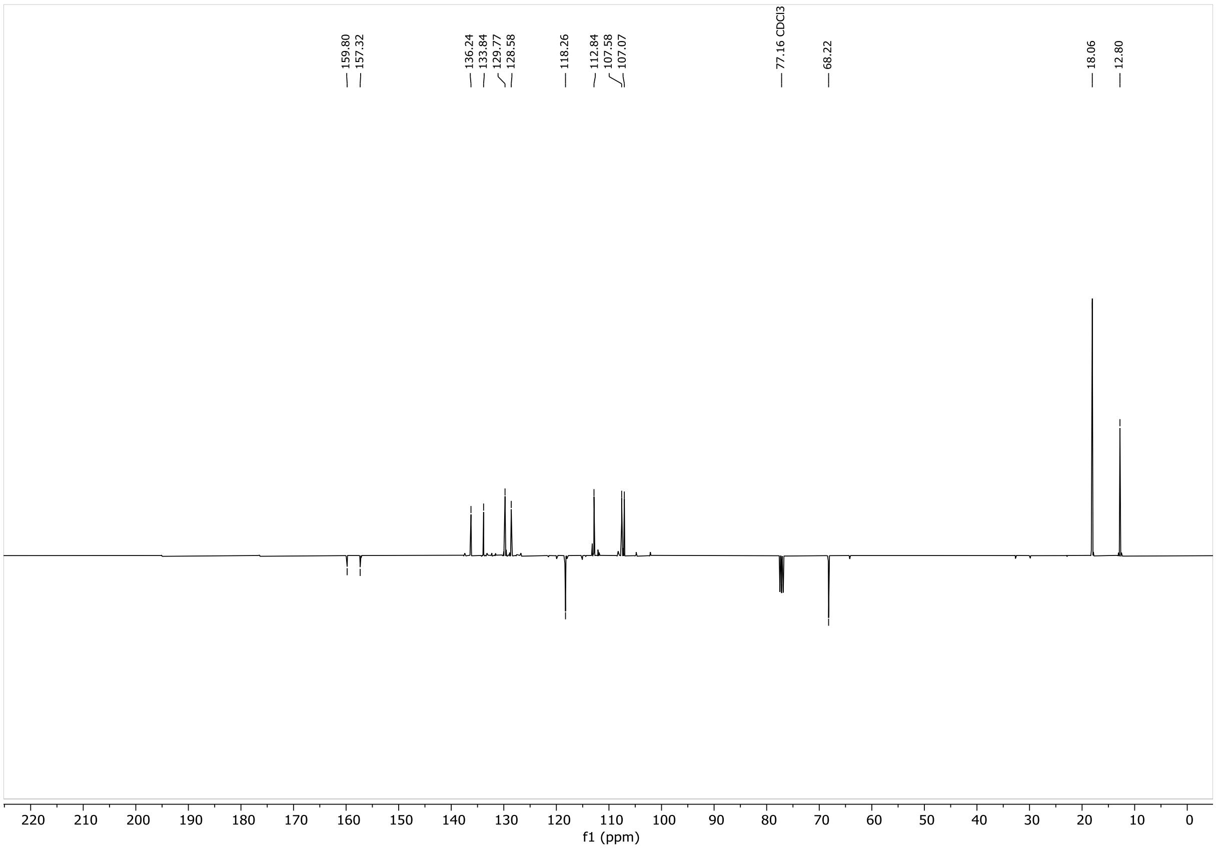

(*E*)-1-(benzyloxy)-2-(penta-2,4-dien-1-yloxy)benzene (10i)

^{1}H NMR (400 MHz, $CDCl_3$)

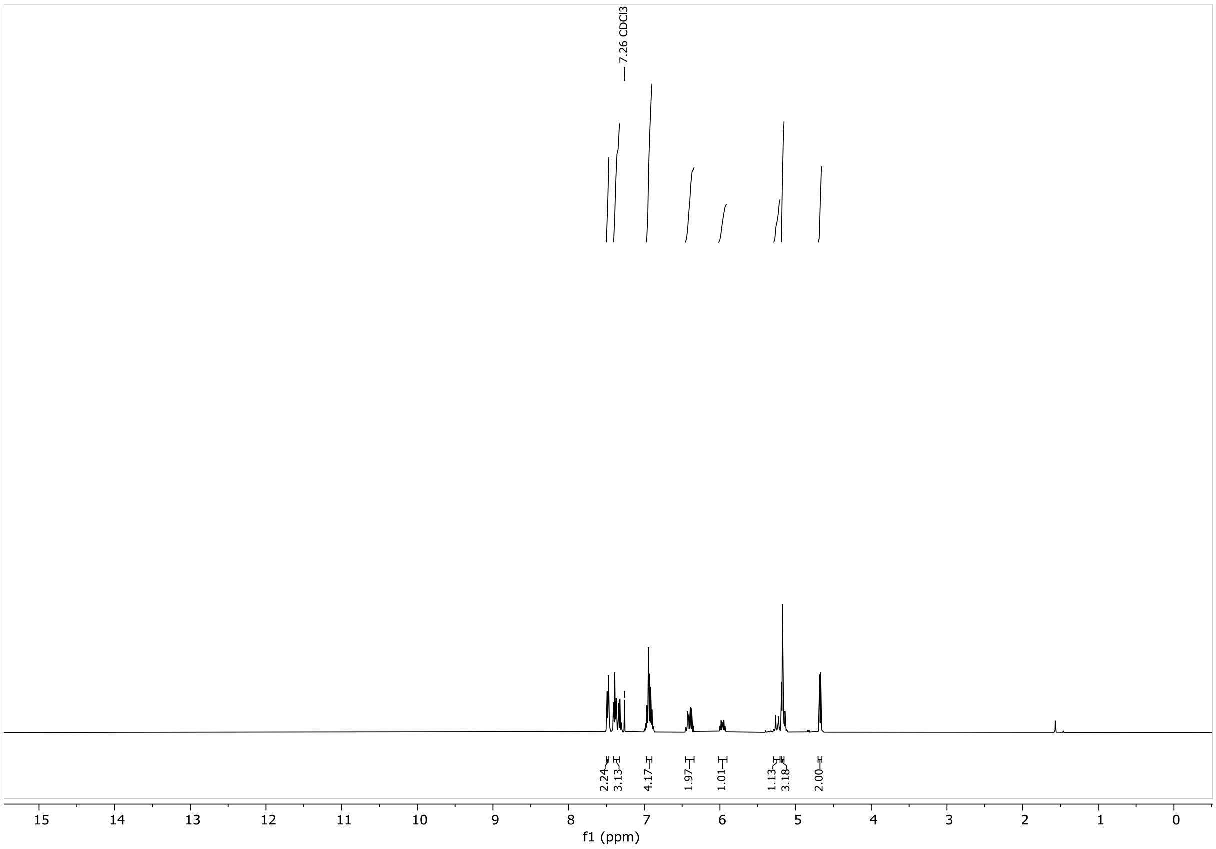

^{13}C-NMR (101 MHz, CDCl$_3$)

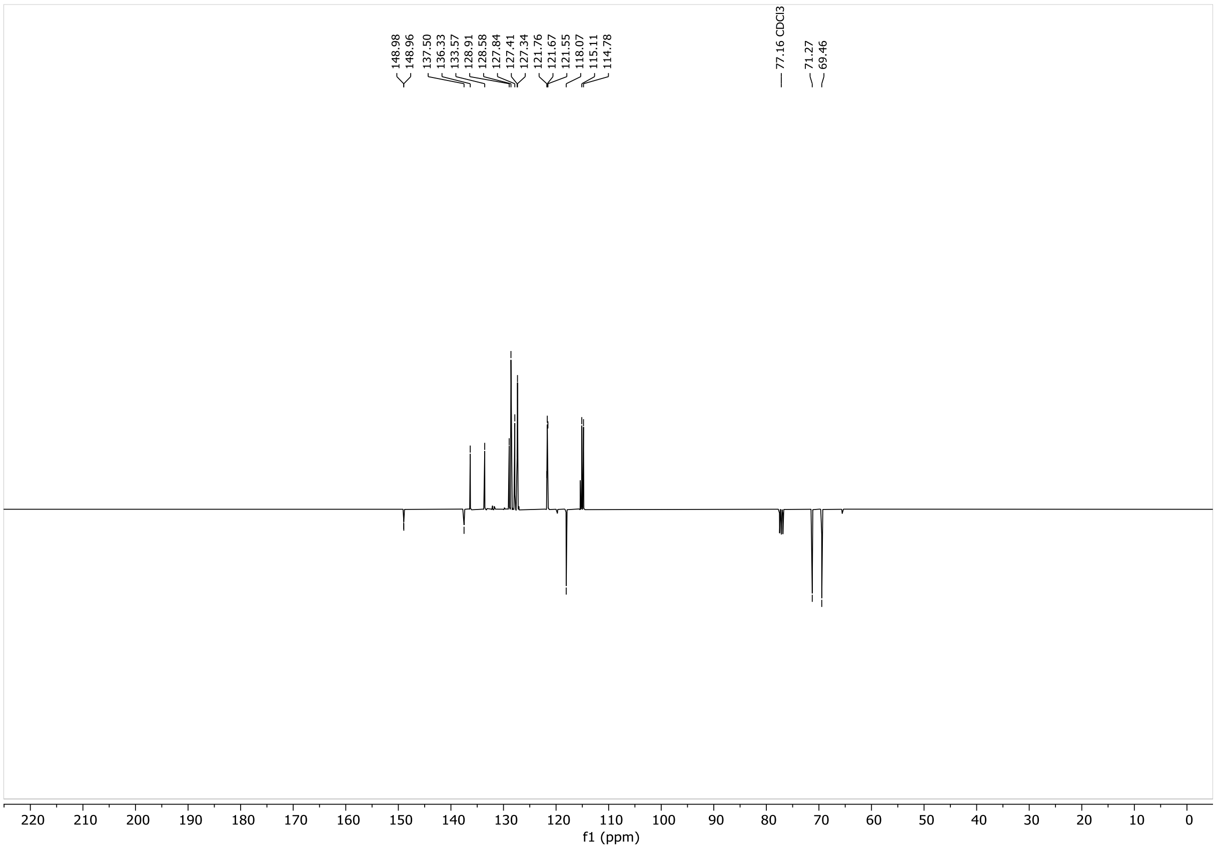

(*E*)-2-(4-methoxy-3-(penta-2,4-dien-1-yloxy)phenyl)-1,3-dioxolane (10j)

^{1}H NMR (400 MHz, CDCl$_3$)

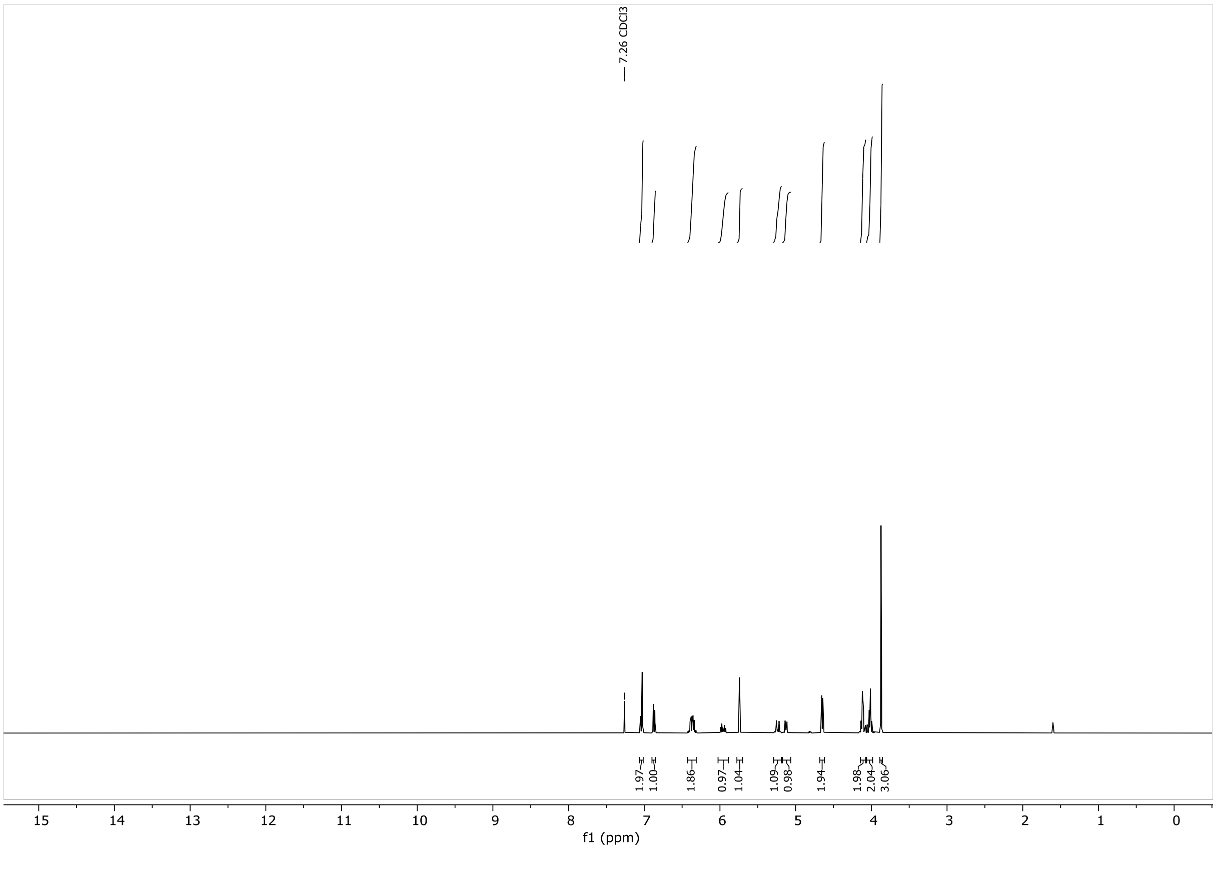

^{13}C-NMR (101 MHz, $CDCl_3$)

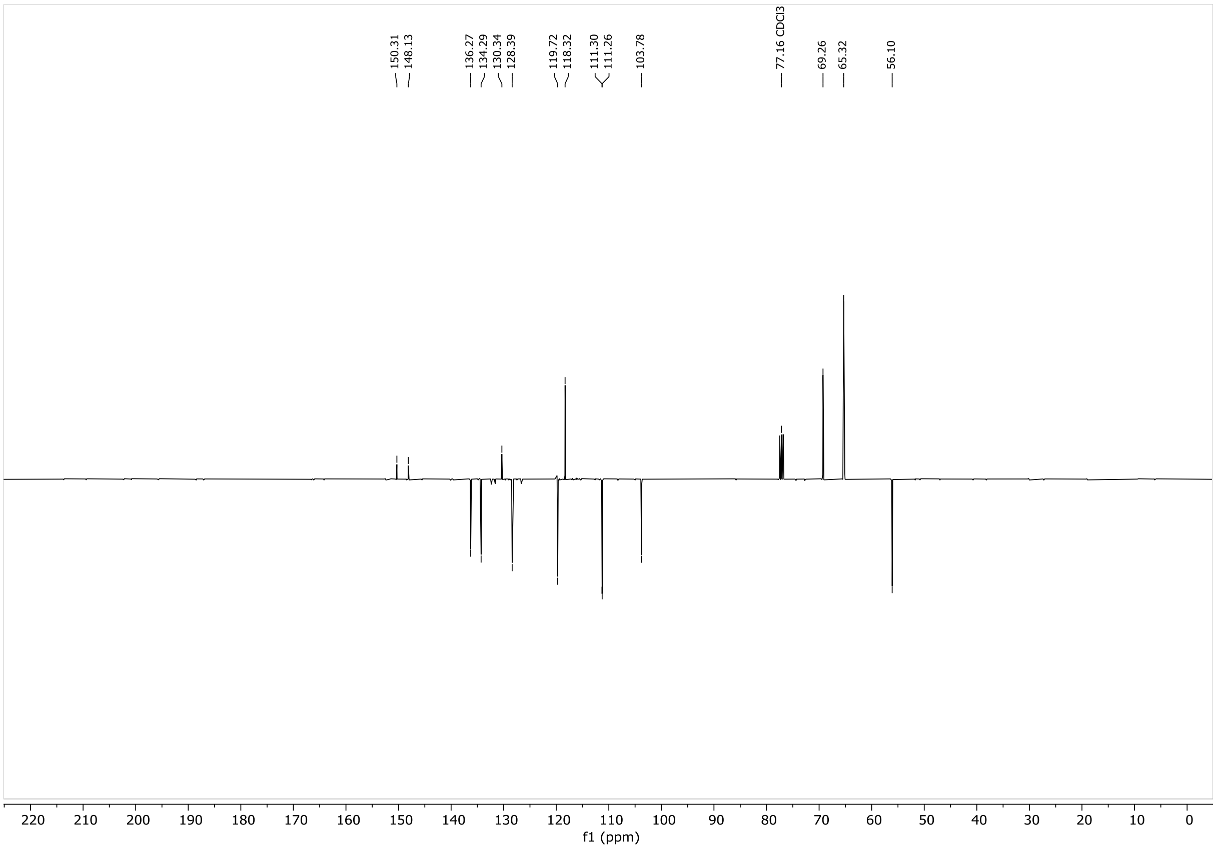

(*E*)-1-fluoro-2-(penta-2,4-dien-1-yloxy)benzene (10k)

^{1}H NMR (400 MHz, $CDCl_3$)

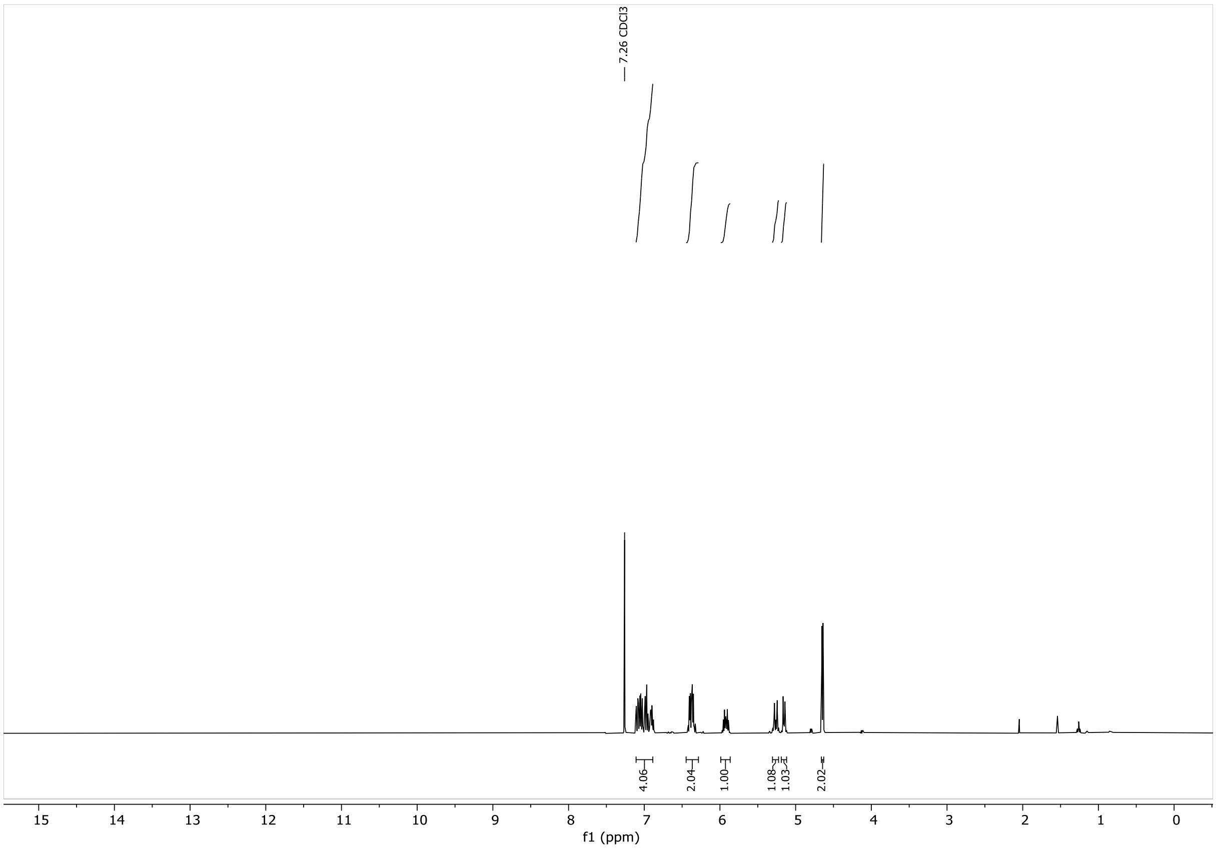

^{13}C-NMR (101 MHz, $CDCl_3$)

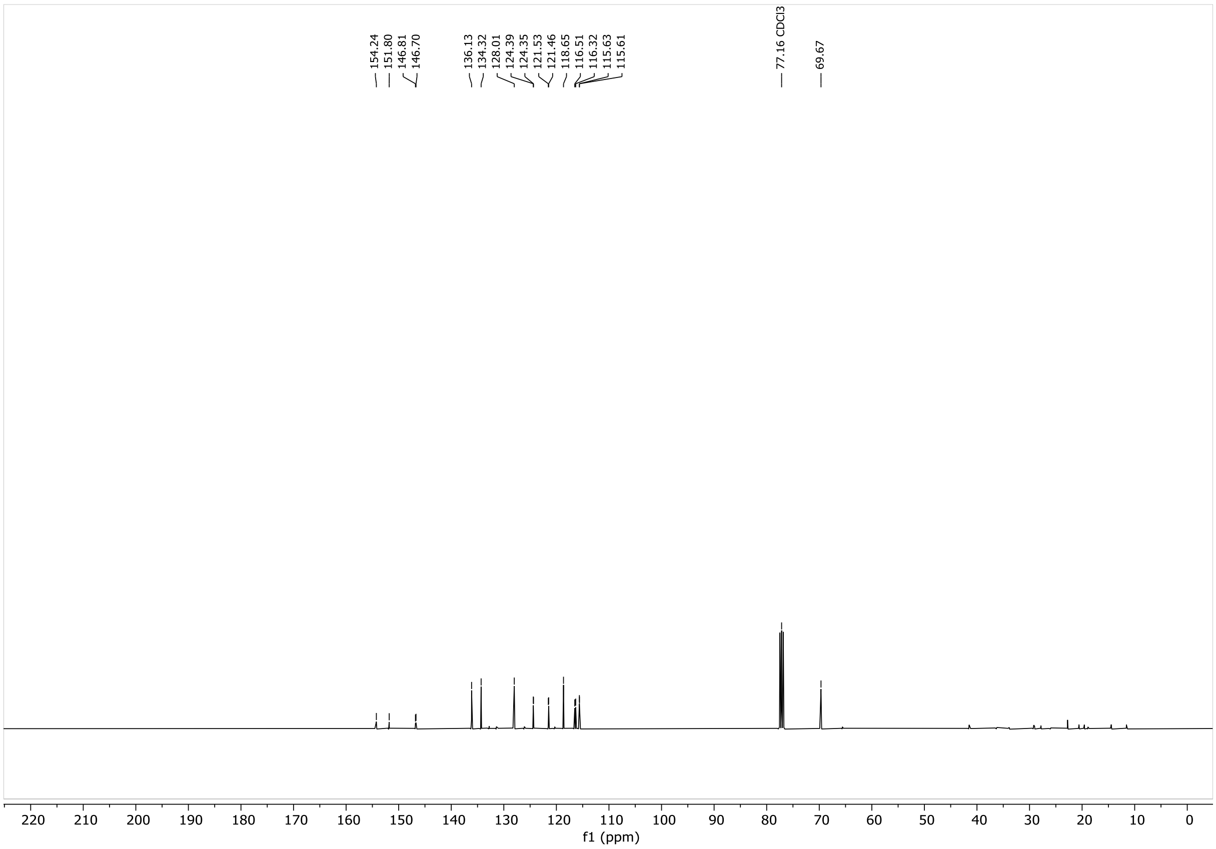

^{19}F-NMR (376 MHz, $CDCl_3$)

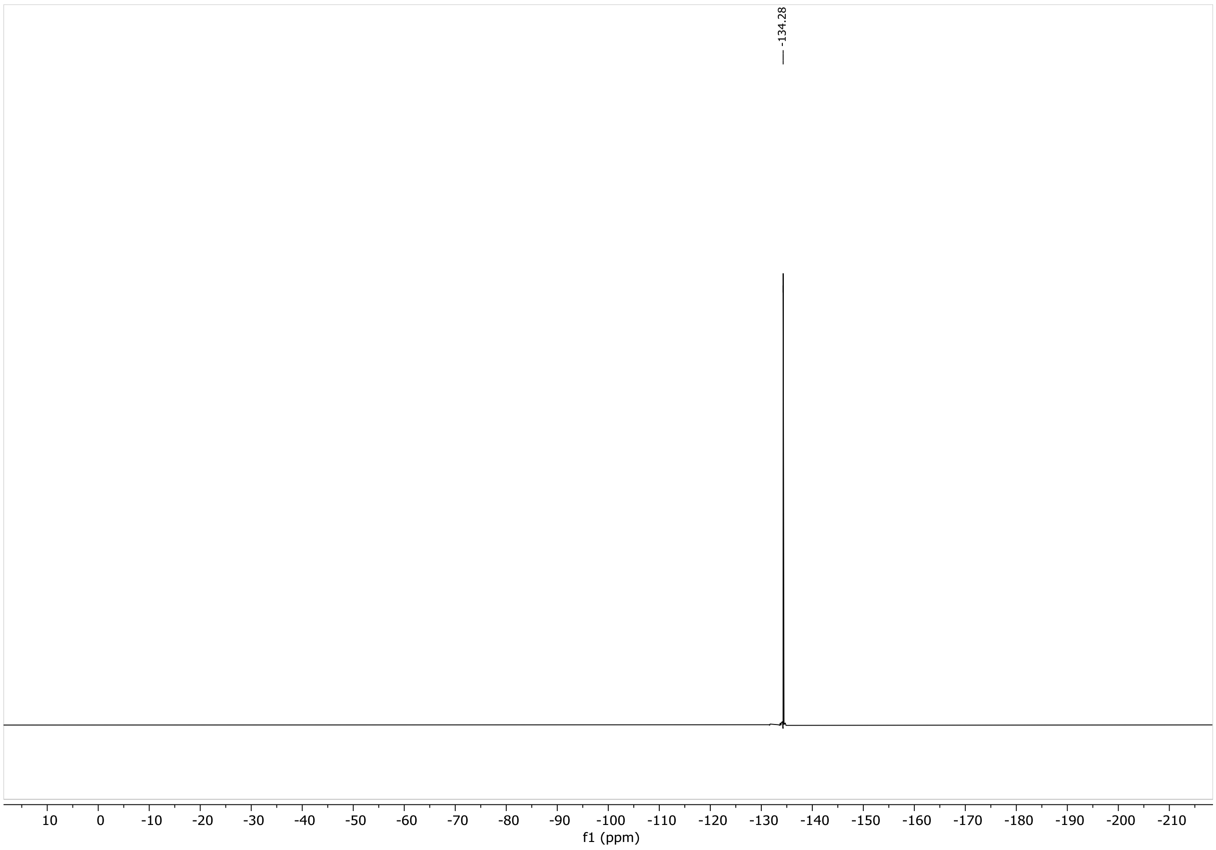

(*E*)-1-bromo-2-(penta-2,4-dien-1-yloxy)benzene (10l)

^{1}H NMR (400 MHz, $CDCl_3$)

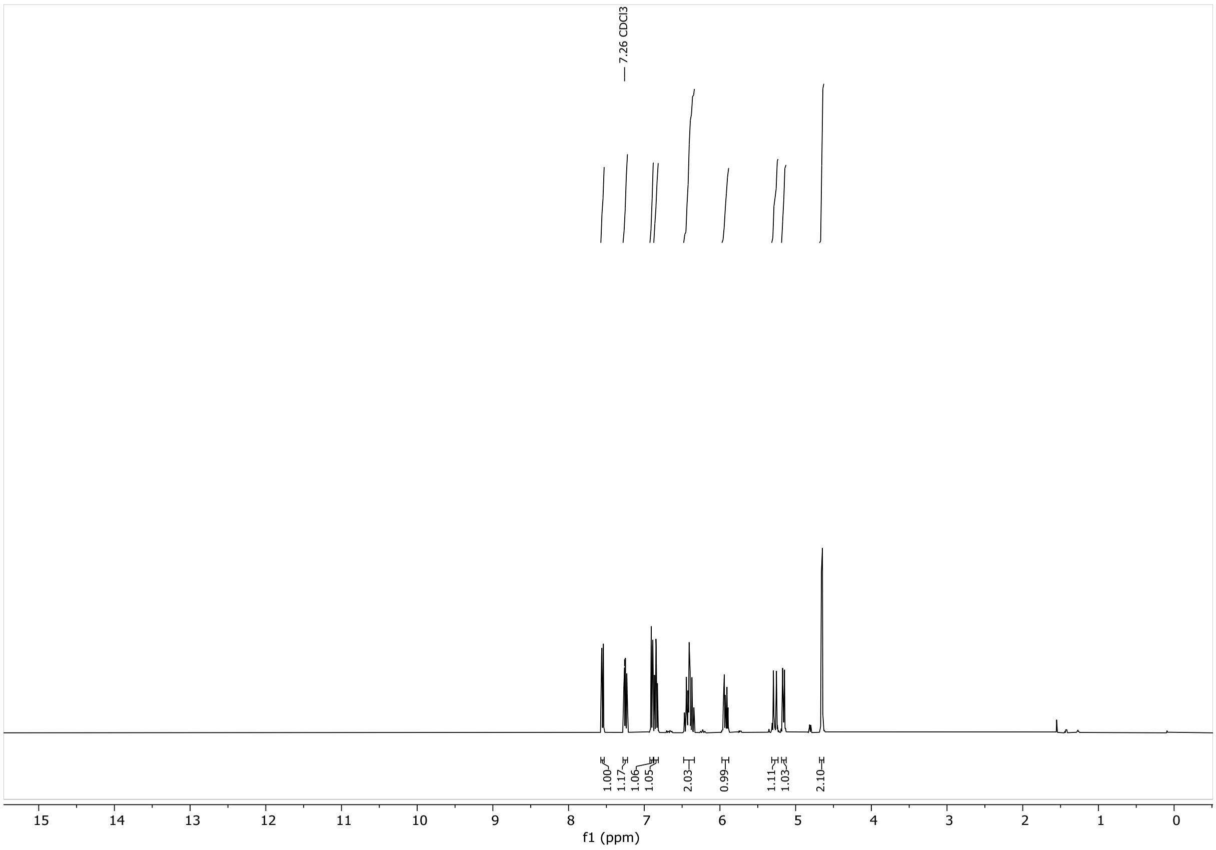

^{13}C-NMR (101 MHz, $CDCl_3$)

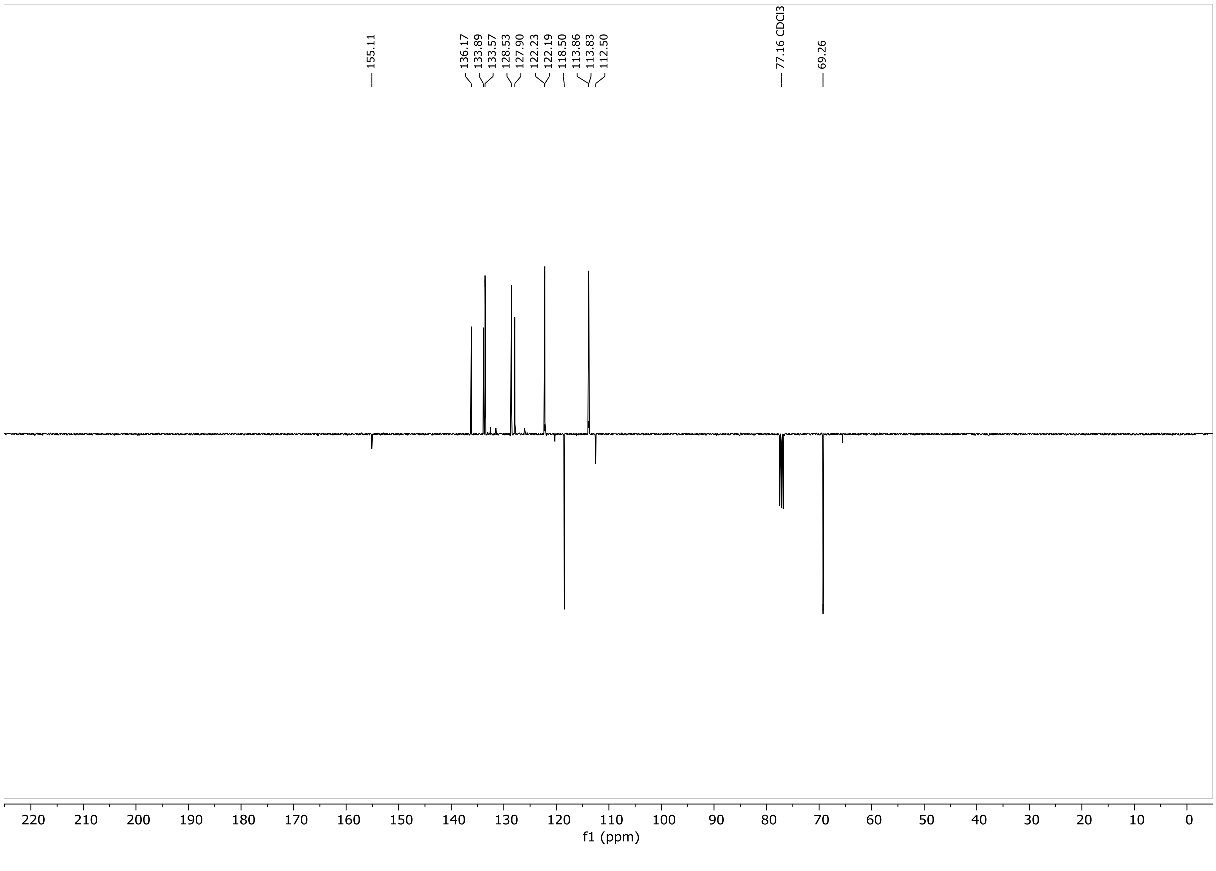

(*E*)-4-methoxy-3-(penta-2,4-dien-1-yloxy)benzaldehydebenzene (10m)

^{1}H NMR (400 MHz, $CDCl_3$)

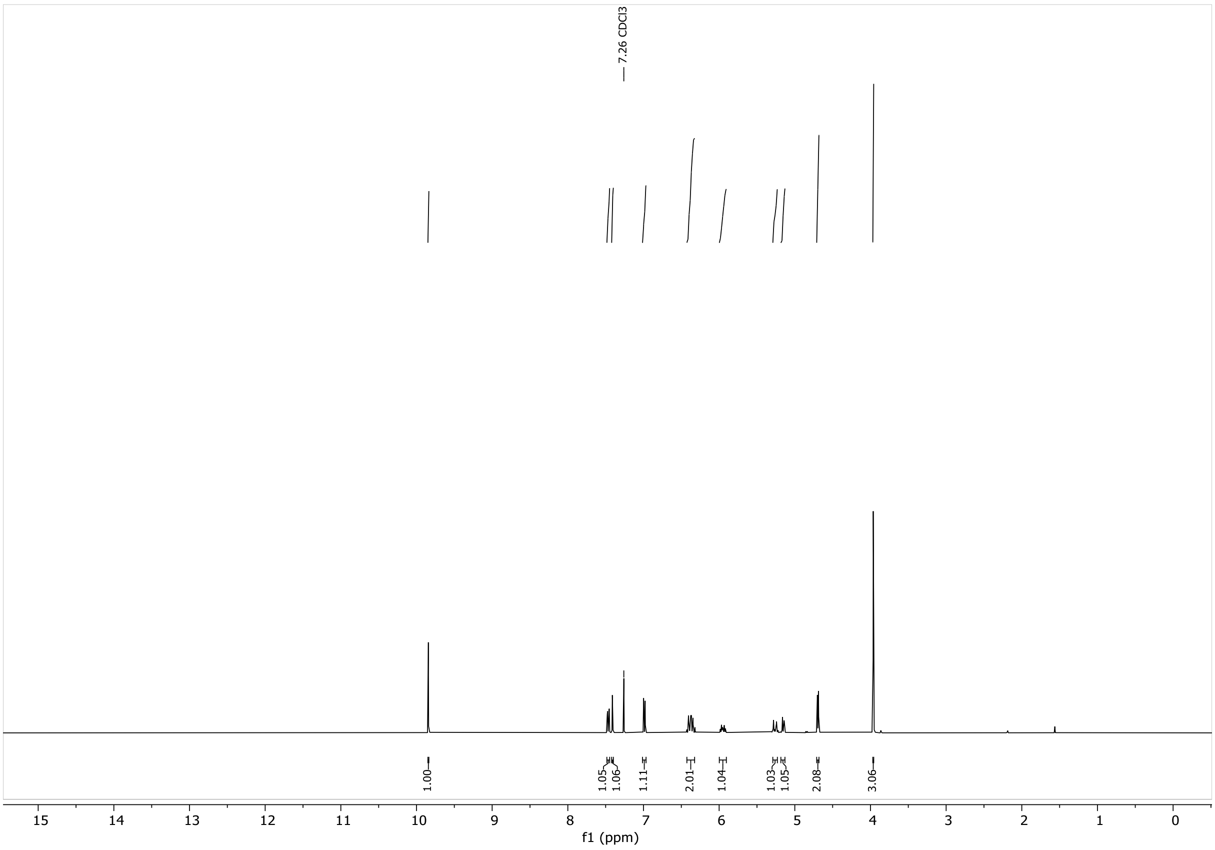

^{13}C-NMR (101 MHz, $CDCl_3$)

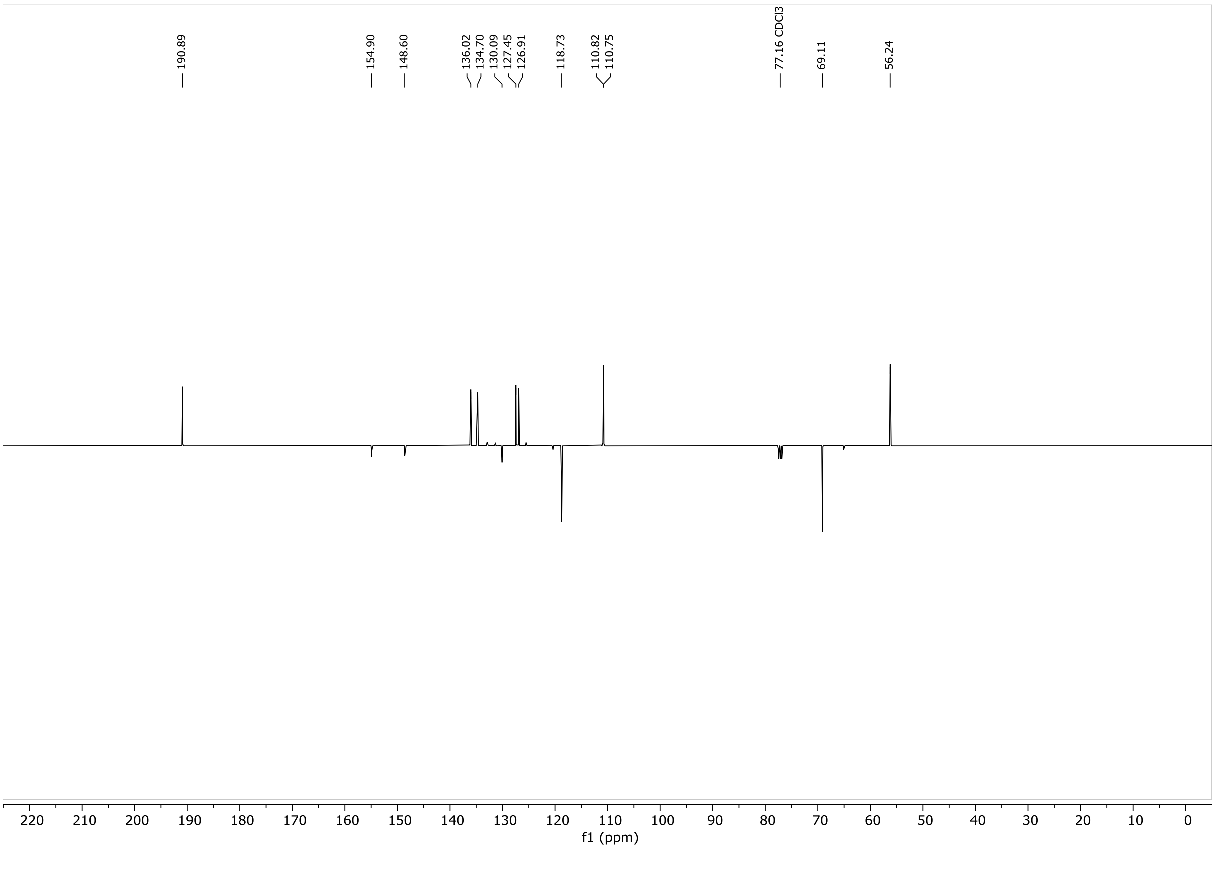

(*E*)-(2-(penta-2,4-dien-1-yloxy)phenyl)(phenyl)methanone (10n)

^{1}H NMR (400 MHz, CDCl$_3$)

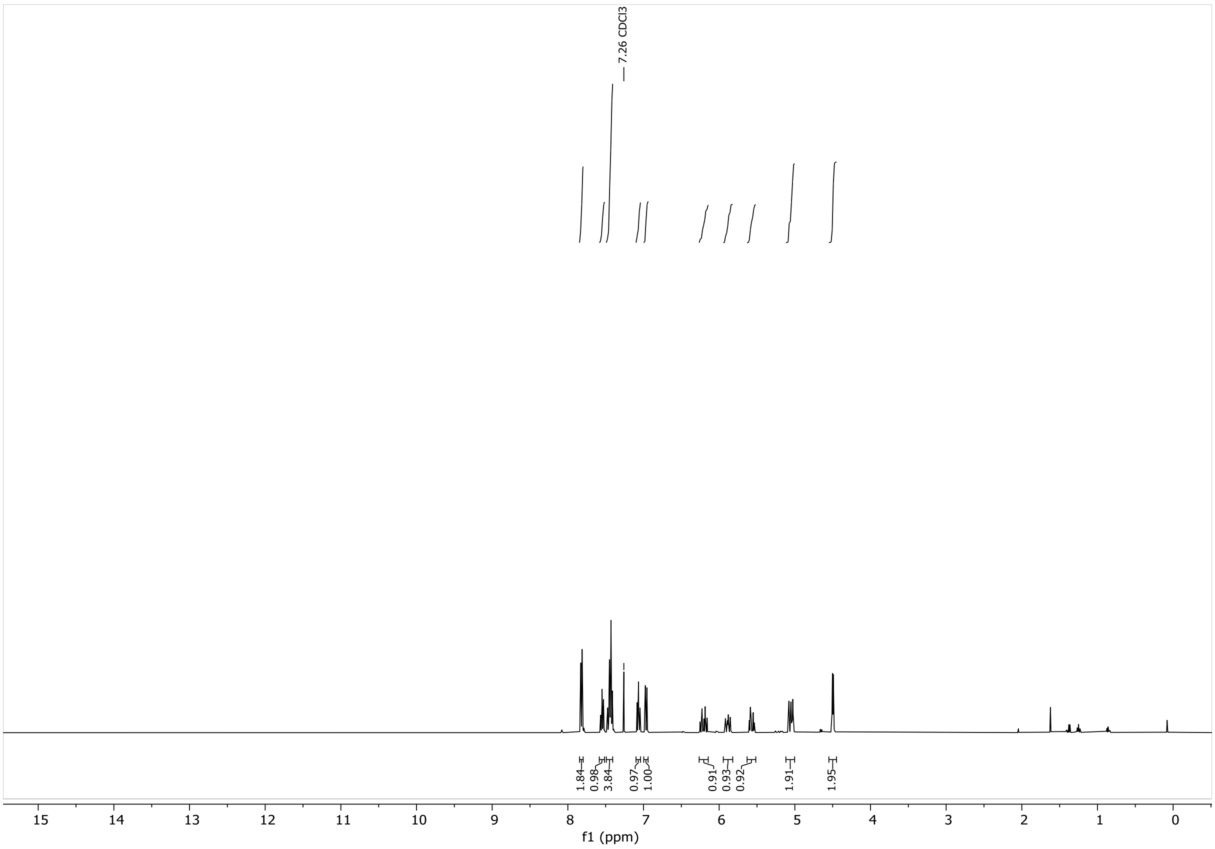

^{13}C-NMR (101 MHz, CDCl$_3$)

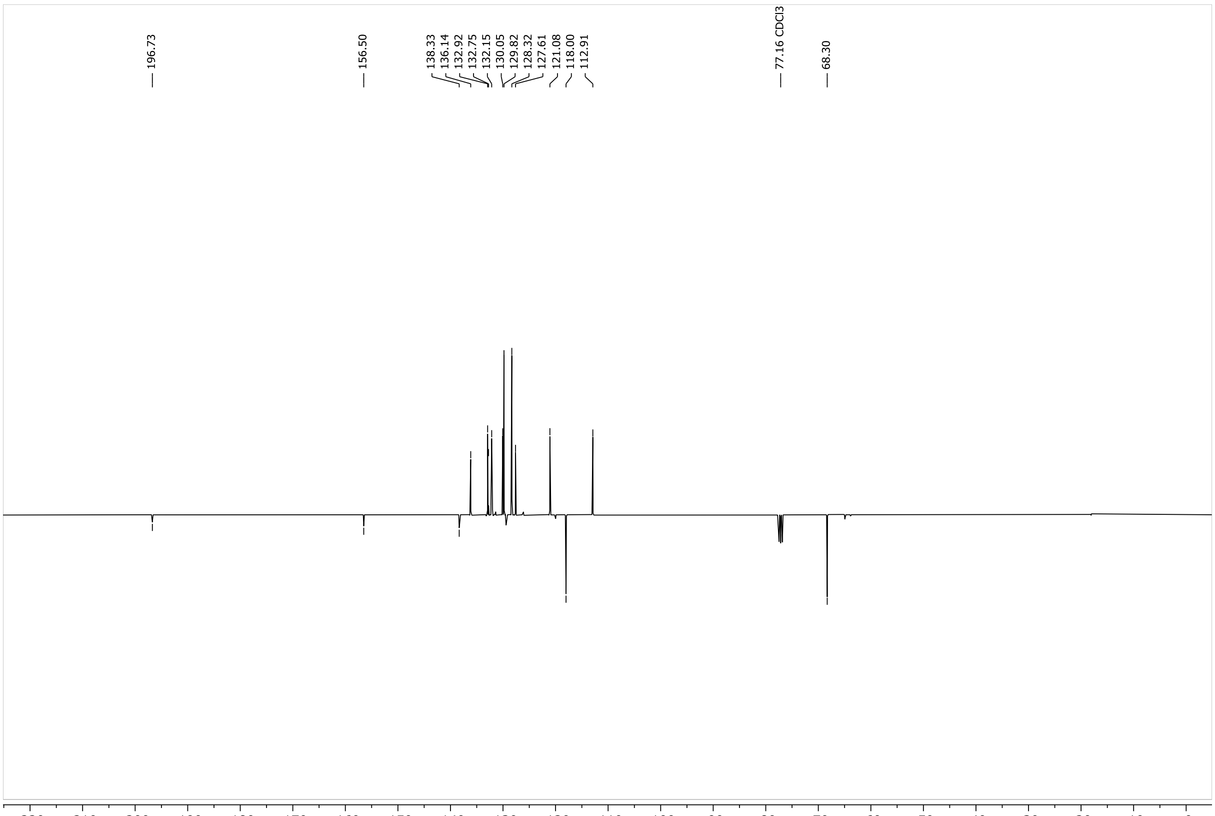

(*E*)-2-(penta-2,4-dien-1-yloxy)phenol (10o)

^{1}H NMR (400 MHz, $CDCl_3$)

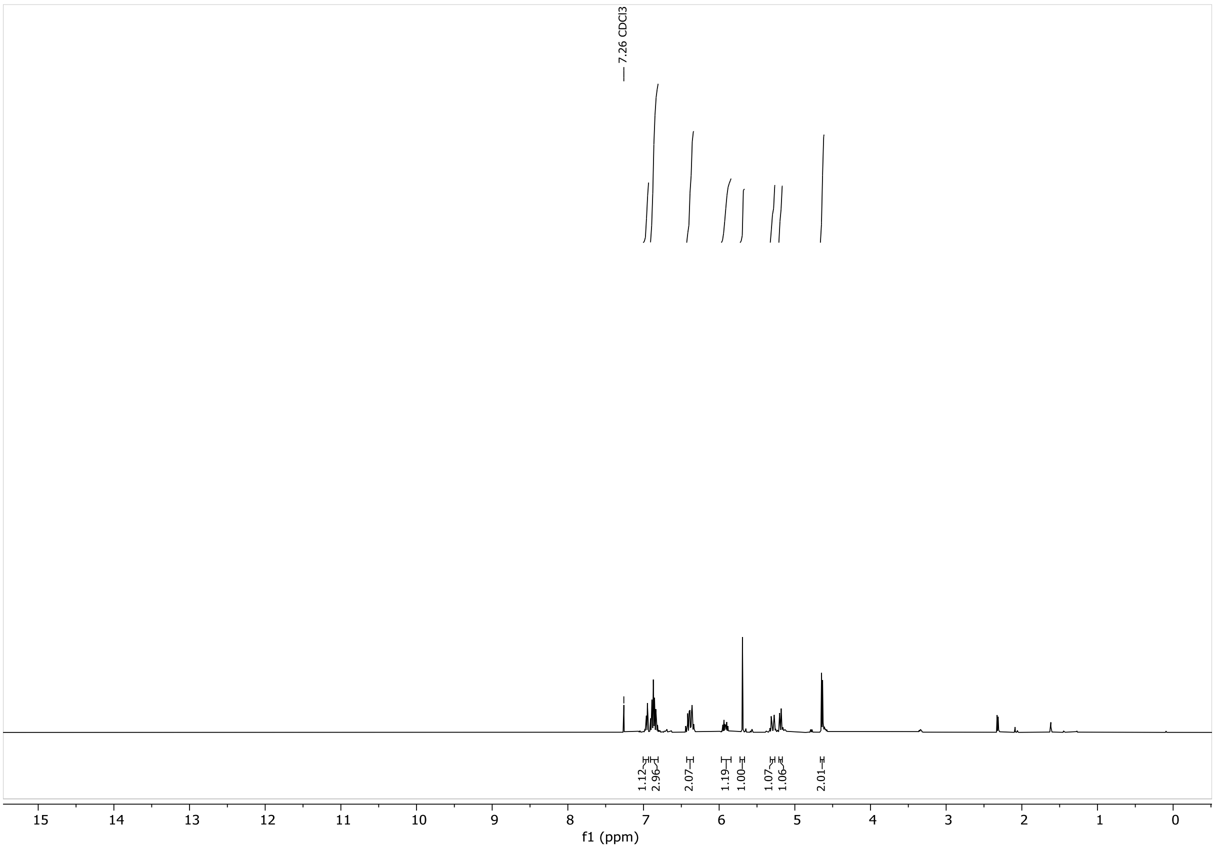

^{13}C-NMR (101 MHz, $CDCl_3$)

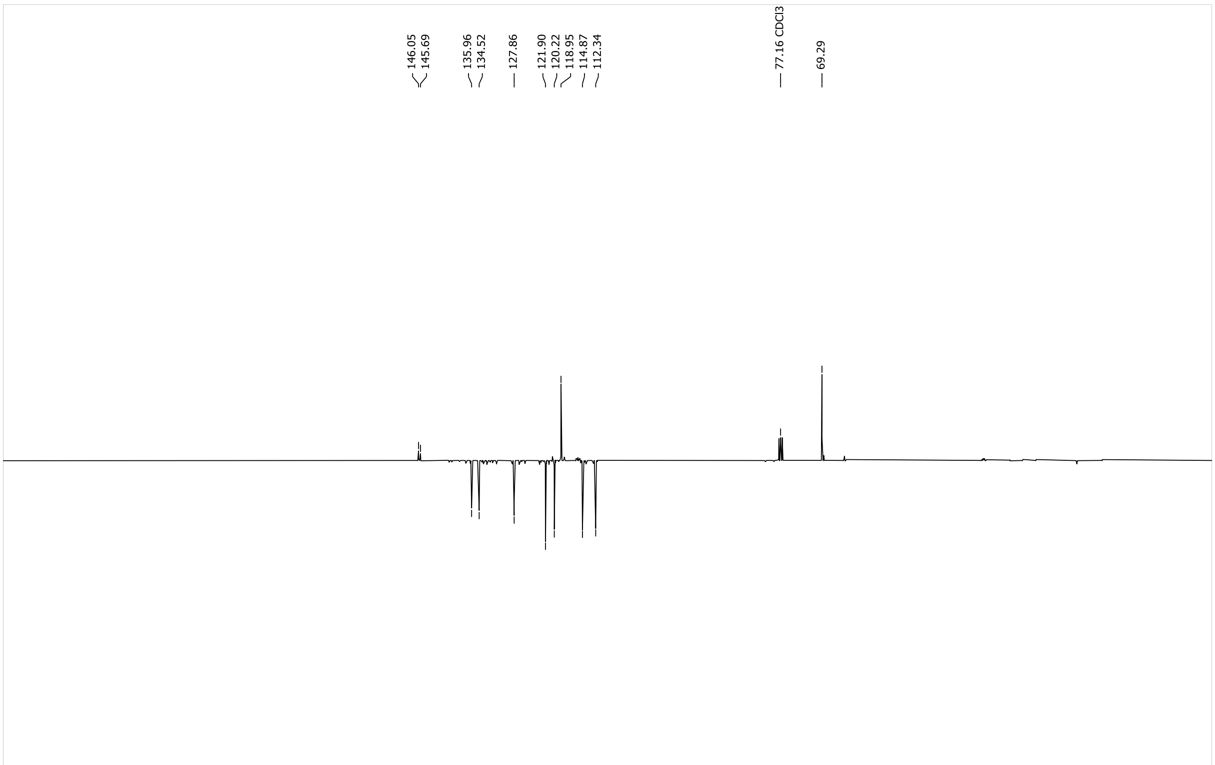

(*E*)-1-nitro-2-(penta-2,4-dien-1-yloxy)benzene (10p)

^{1}H NMR (400 MHz, $CDCl_3$)

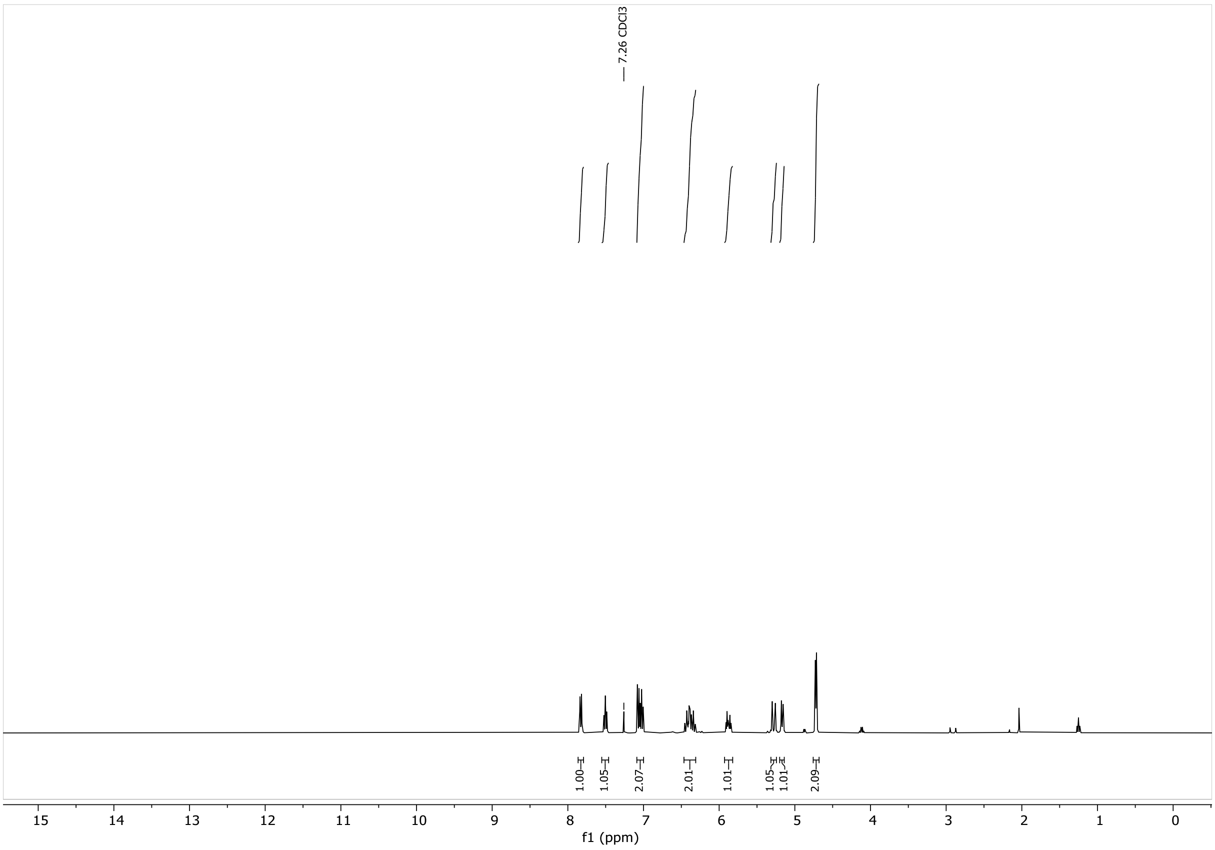

^{13}C-NMR (101 MHz, $CDCl_3$)

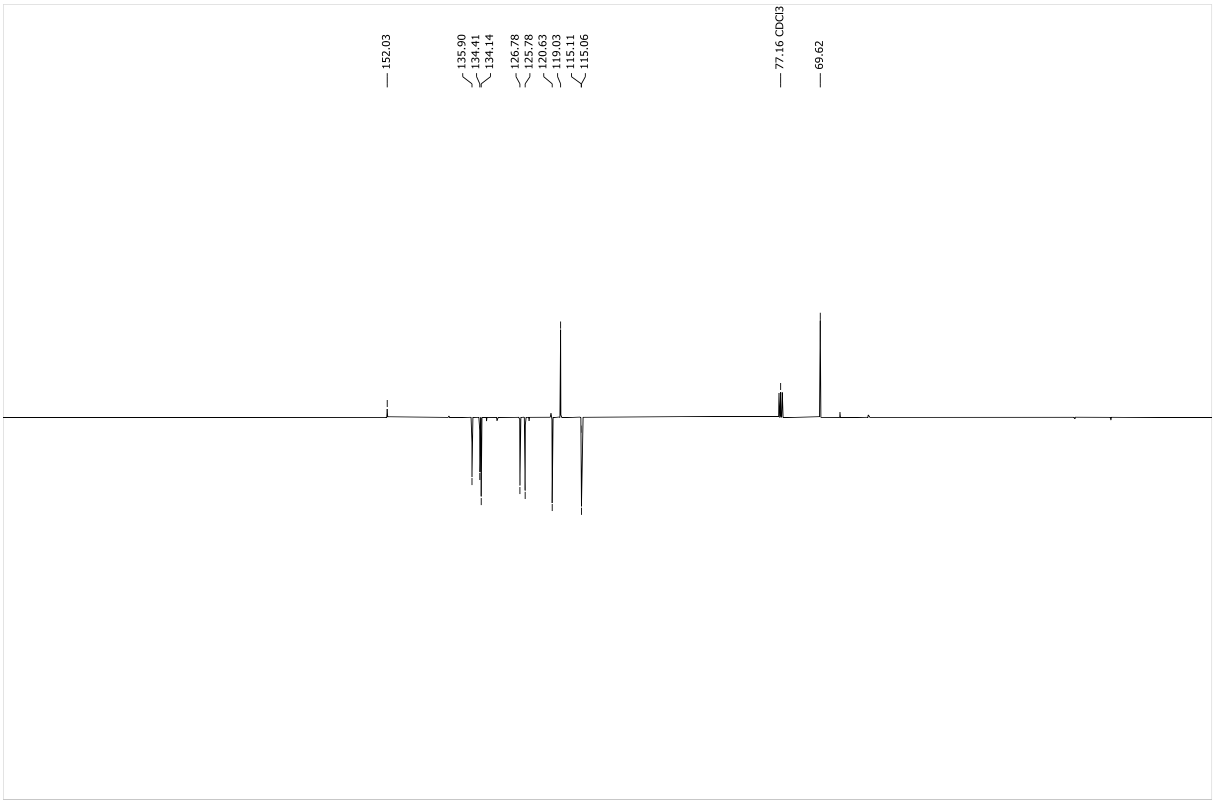

(*E*)-1-nitro-3-(penta-2,4-dien-1-yloxy)benzene (10q)

^{1}H NMR (400 MHz, $CDCl_3$)

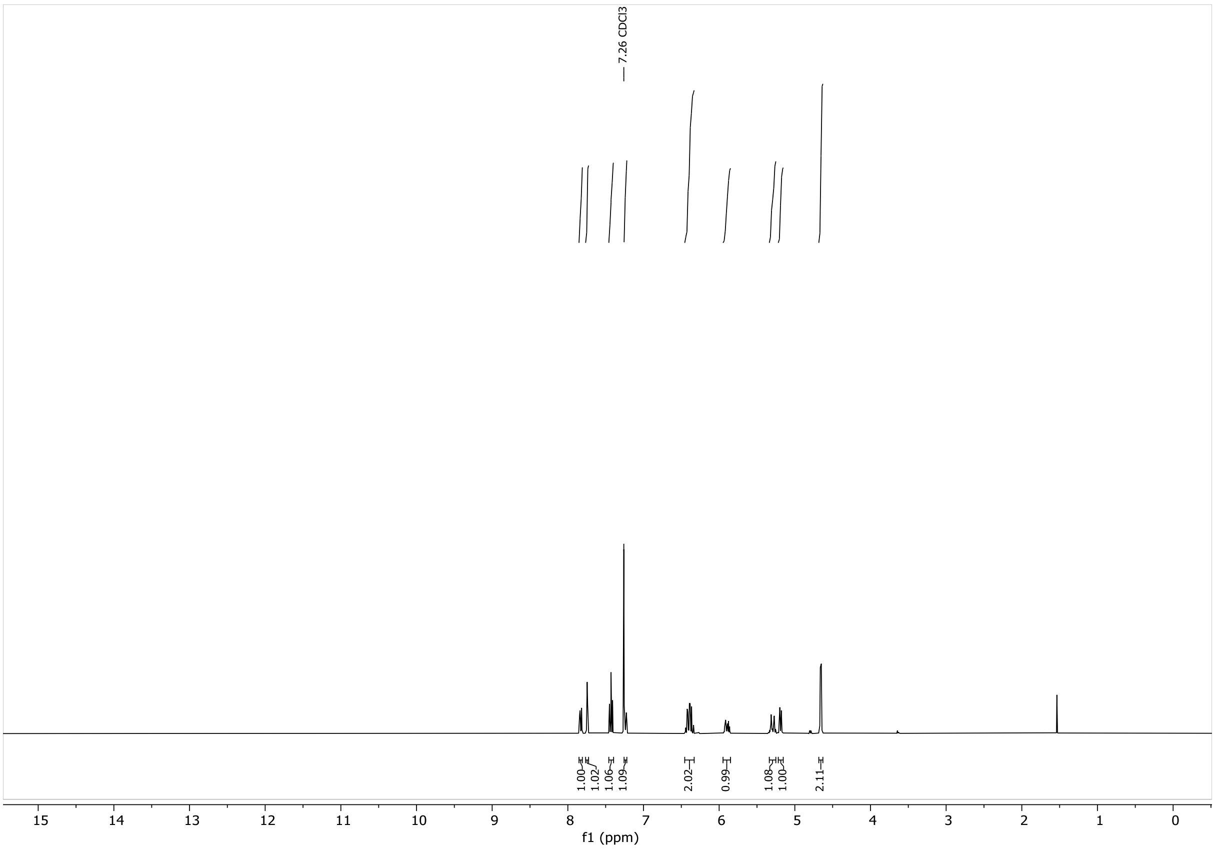

^{13}C-NMR (101 MHz, $CDCl_3$)

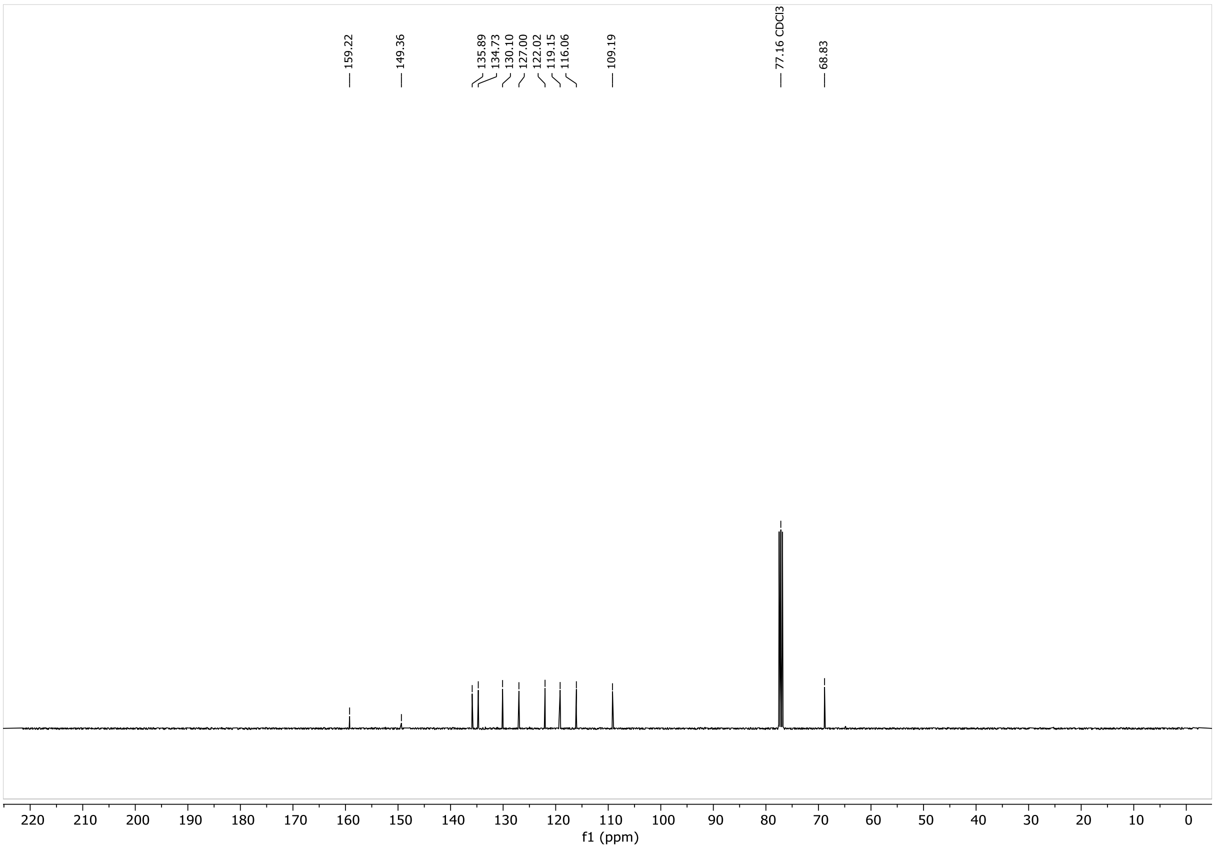

4-Methyl-2-(penta-1,4-dien-3-yl)phenol (9a)

^{1}H NMR (400 MHz, $CDCl_3$)

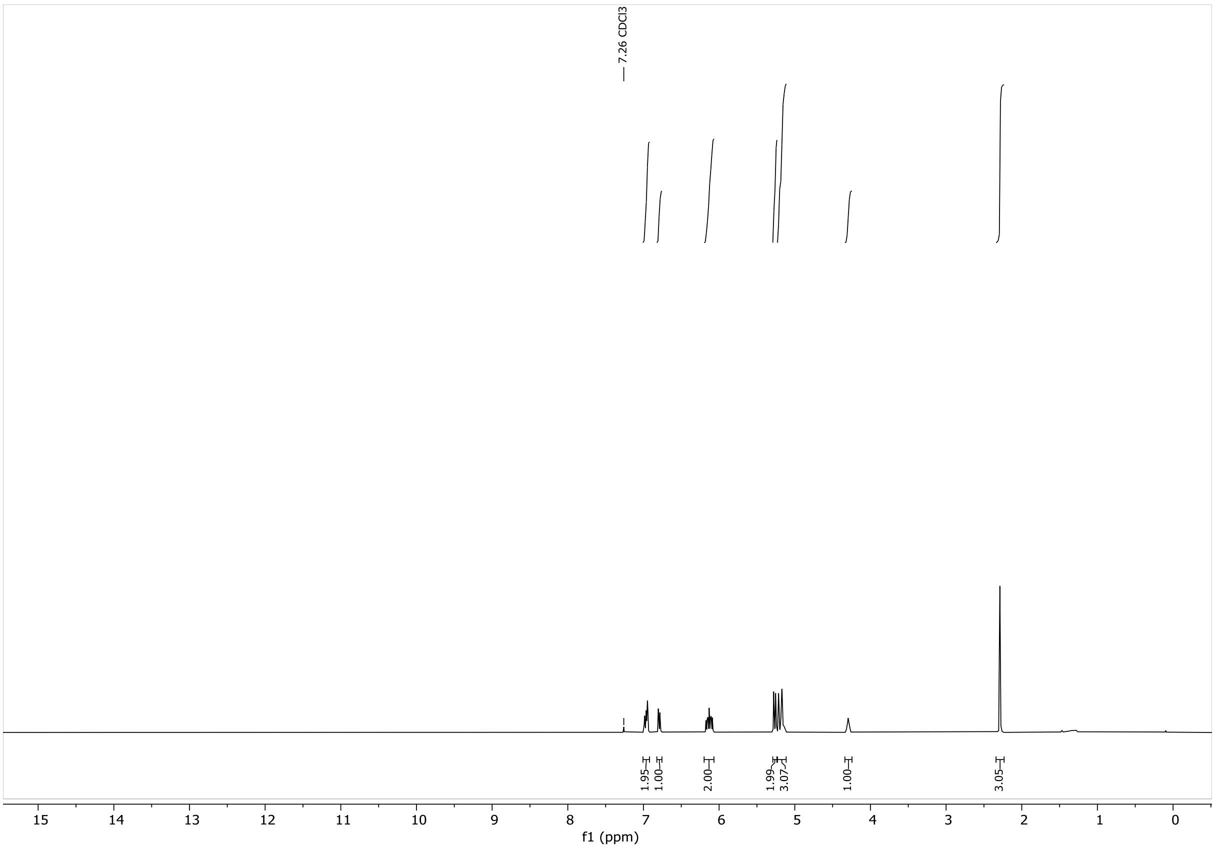

^{13}C-NMR (101 MHz, $CDCl_3$)

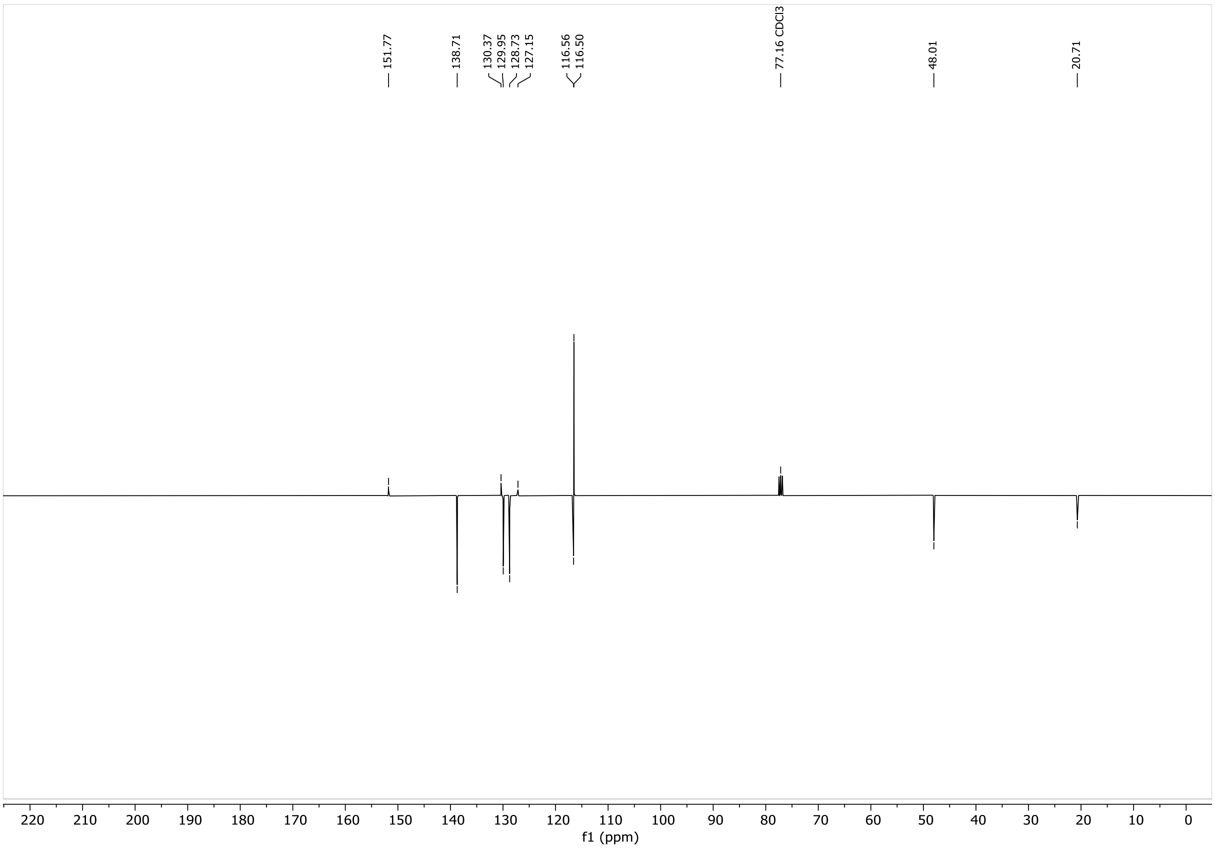

4-(tert-butyl)-2-(penta-1,4-dien-3-yl)phenol (9b)

^{1}H NMR (400 MHz, $CDCl_3$)

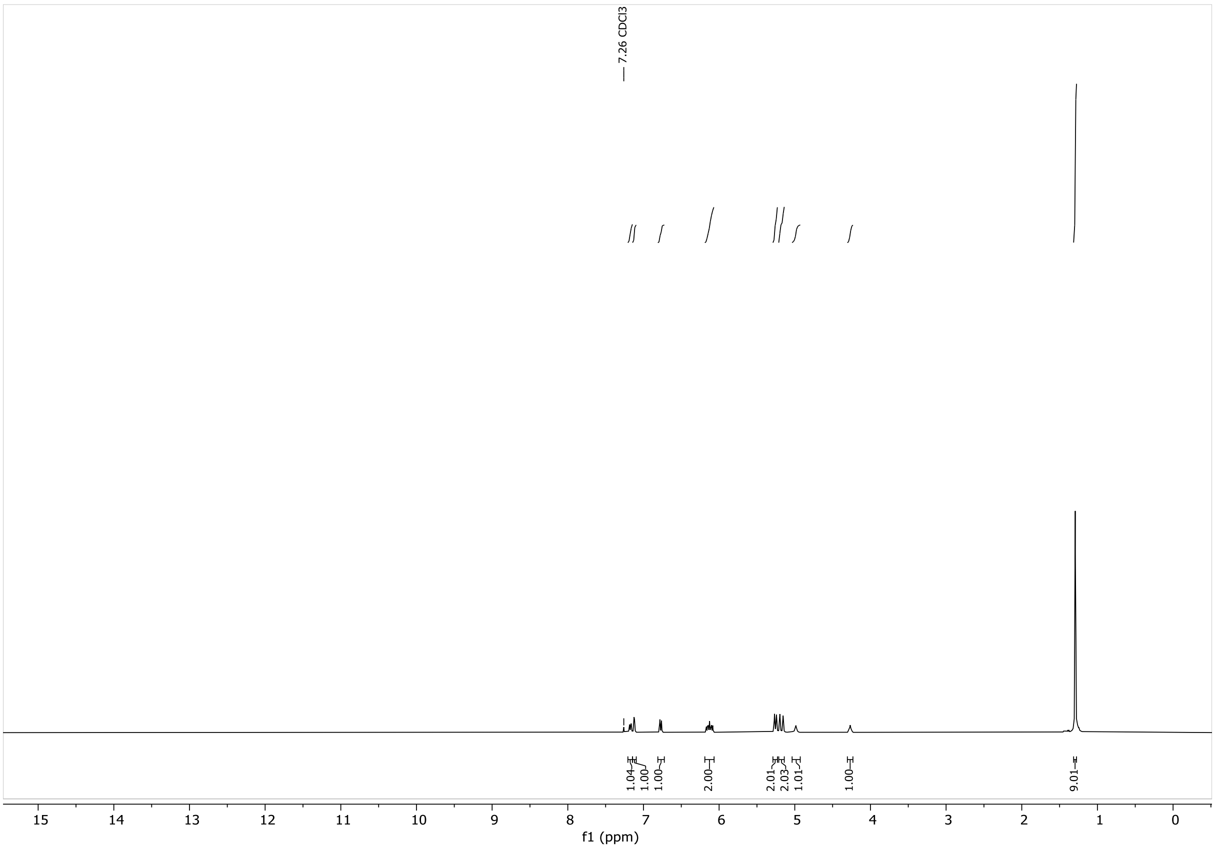

^{13}C-NMR (101 MHz, $CDCl_3$)

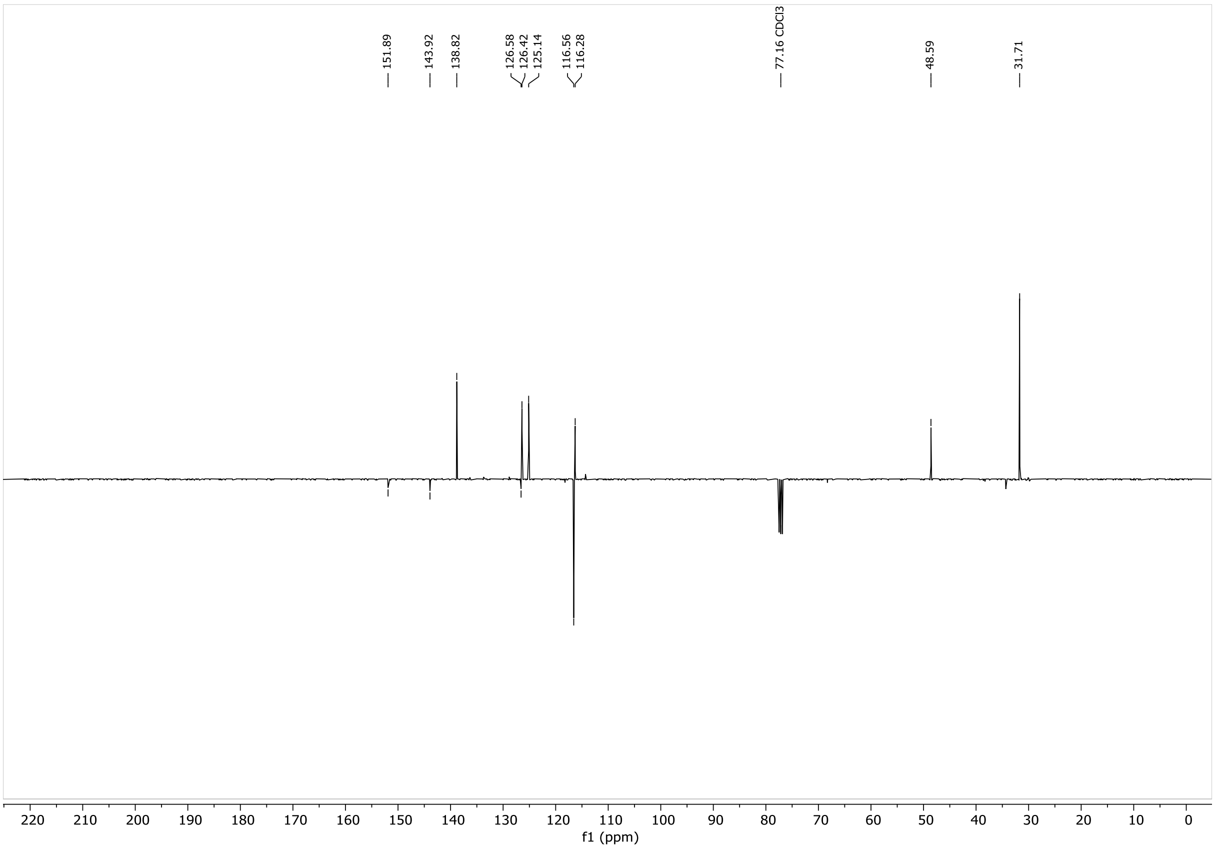

3-(Penta-1,4-dien-3-yl)-[1,1'-biphenyl]-4-ol (9c)

^{1}H NMR (400 MHz, $CDCl_3$)

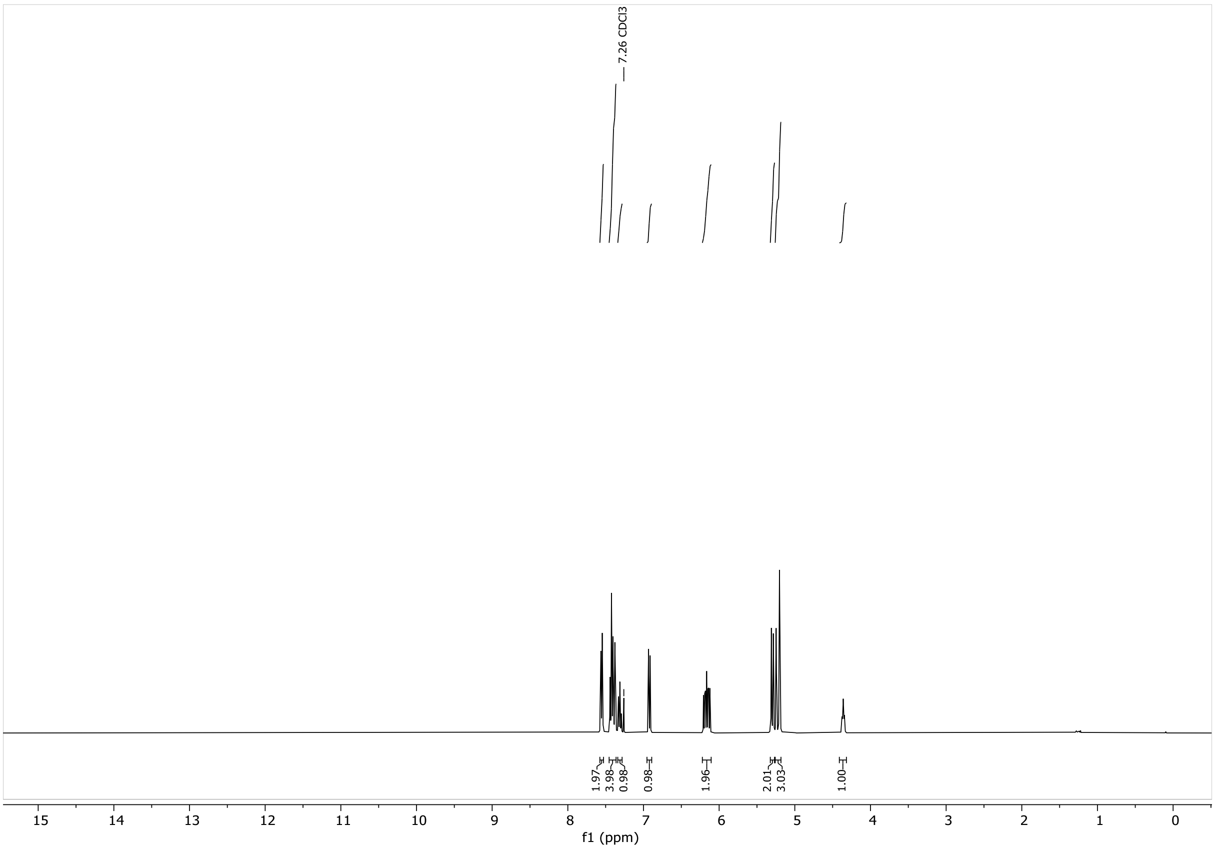

^{13}C-NMR (101 MHz, $CDCl_3$)

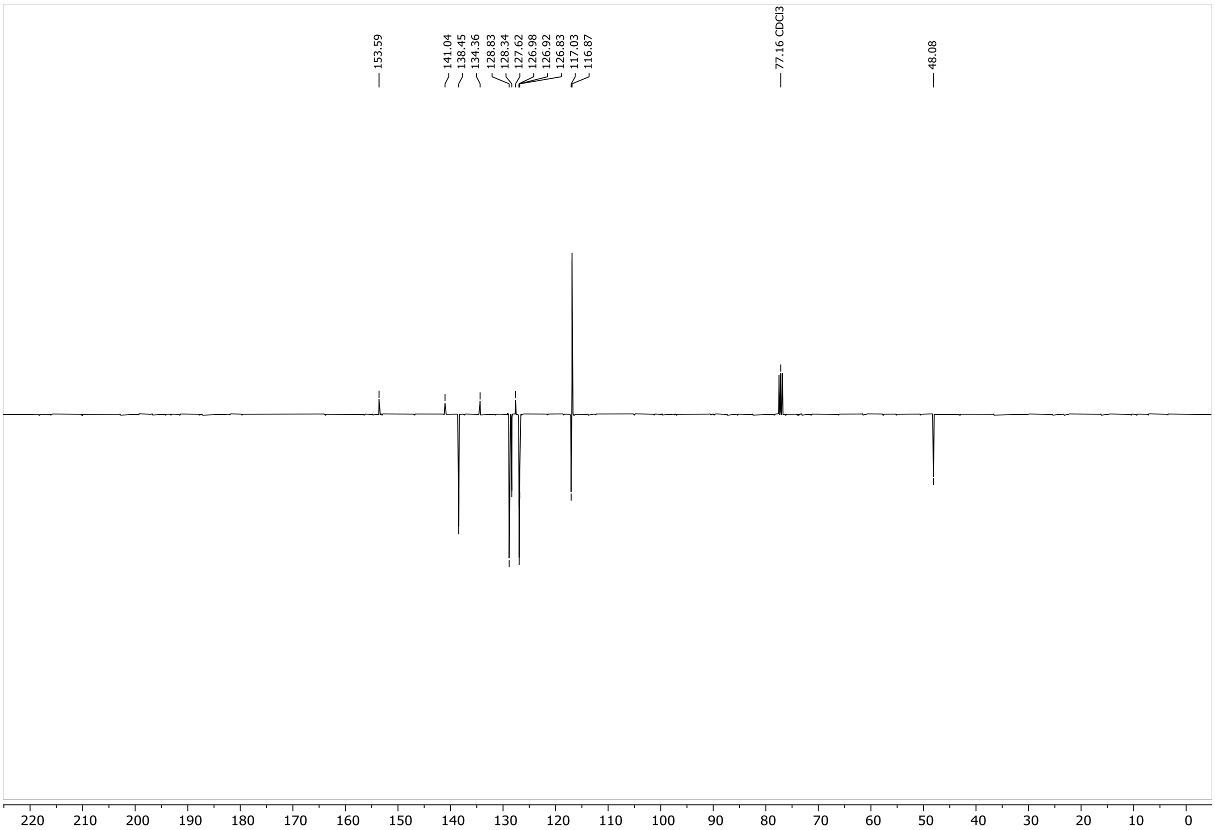

4-Allyl-2-methoxy-6-(penta-1,4-dien-3-yl)phenol (9d)

^{1}H NMR (400 MHz, $CDCl_3$)

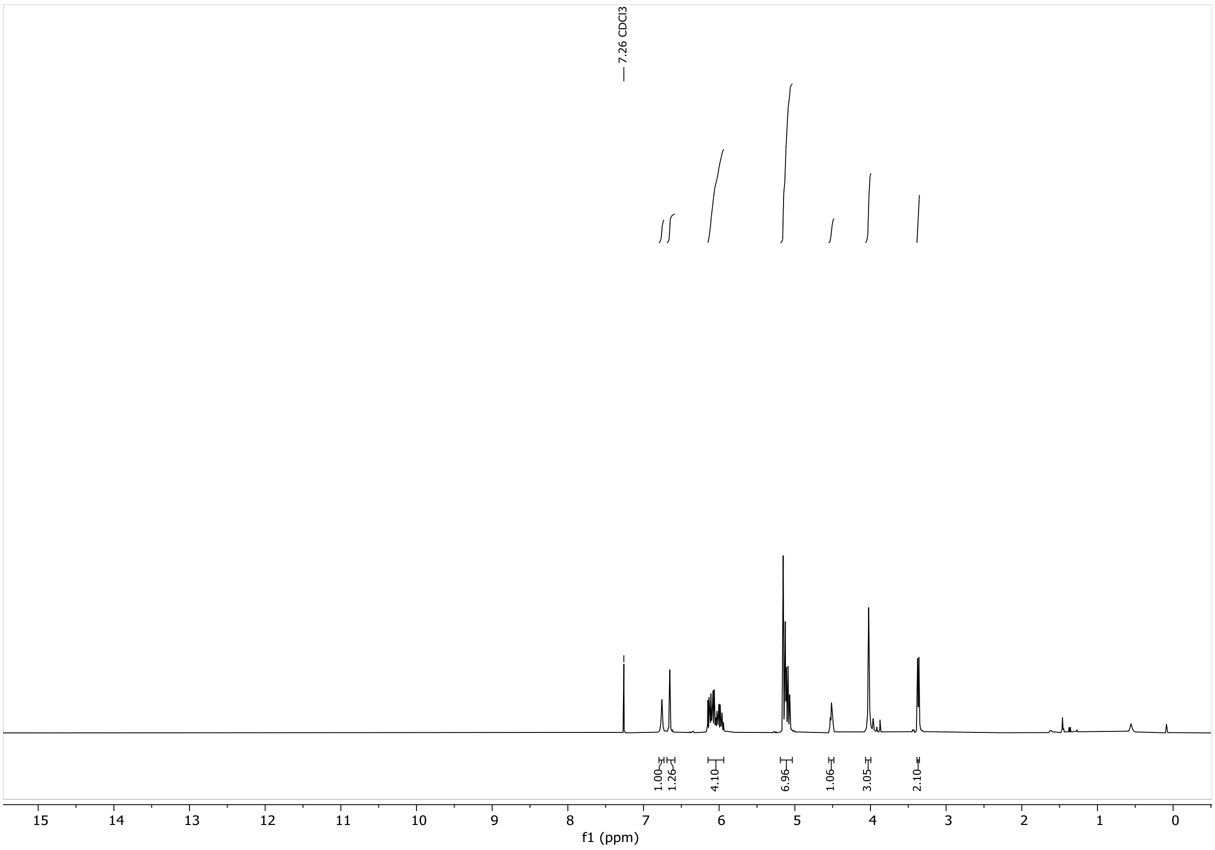

^{13}C-NMR (101 MHz, $CDCl_3$)

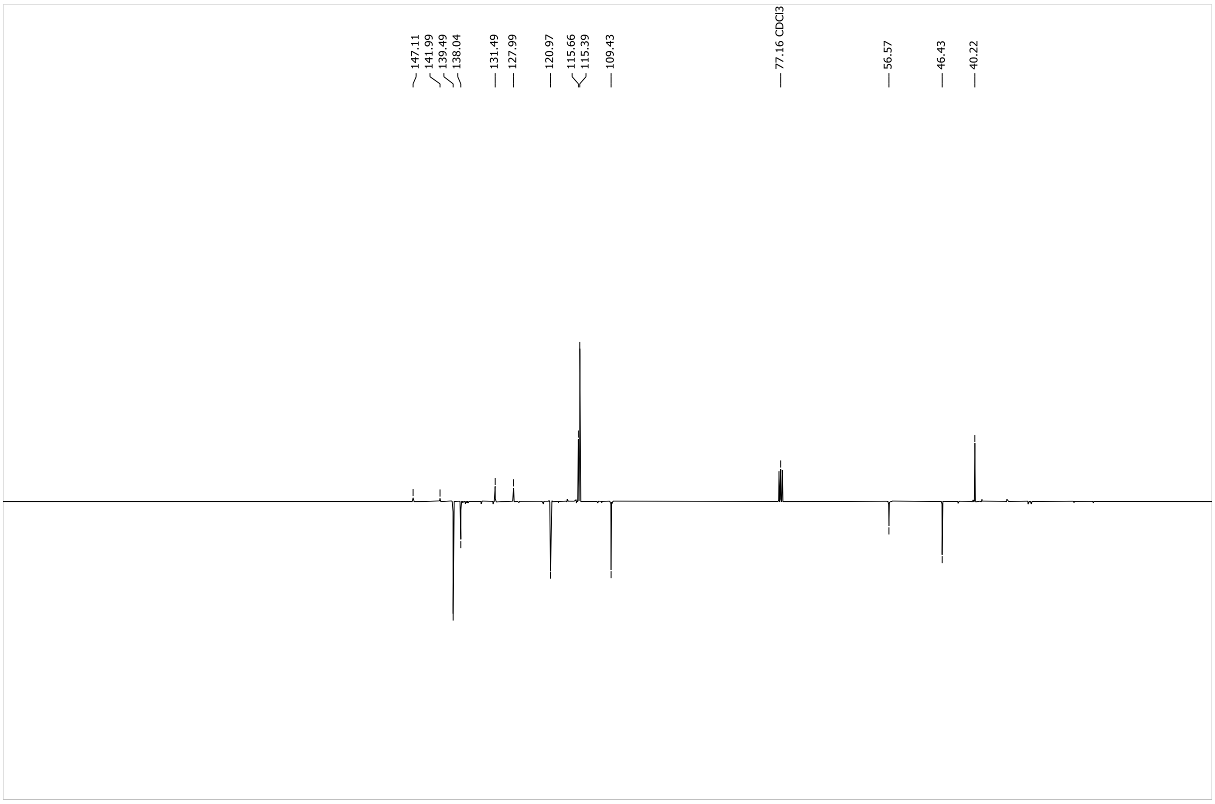

4-Methoxy-2-(penta-1,4-dien-3-yl)phenol (9e)

^{1}H NMR (400 MHz, $CDCl_3$)

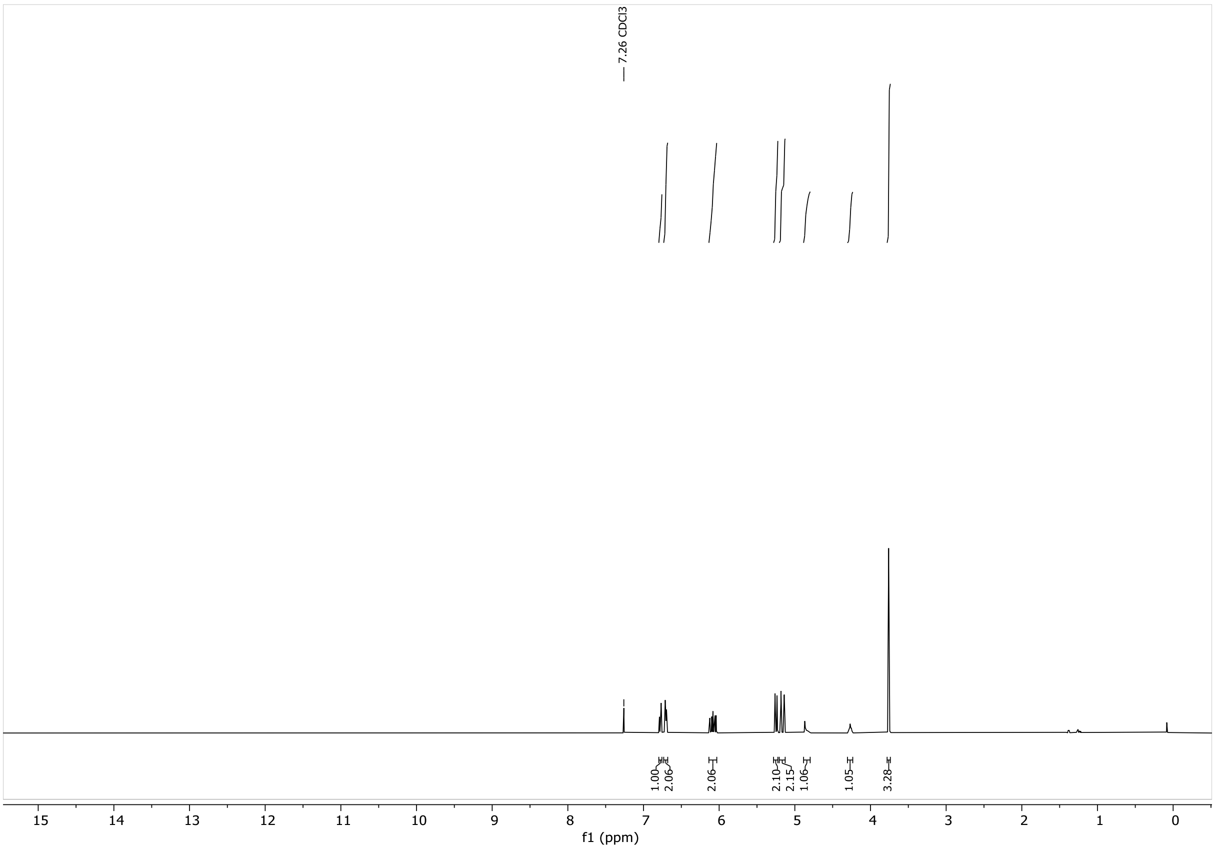

^{13}C-NMR (101 MHz, $CDCl_3$)

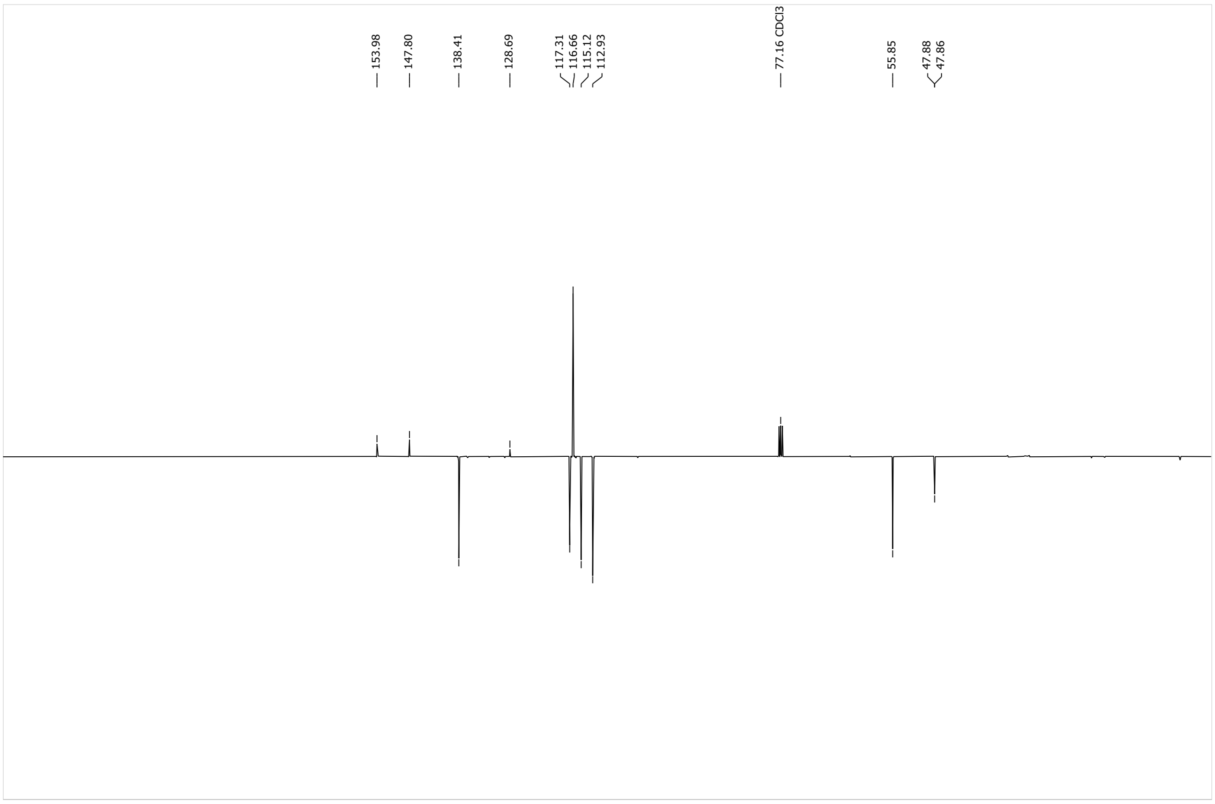

4-(Benzyloxy)-2-(penta-1,4-dien-3-yl)phenol (9f)

^{1}H NMR (400 MHz, $CDCl_3$)

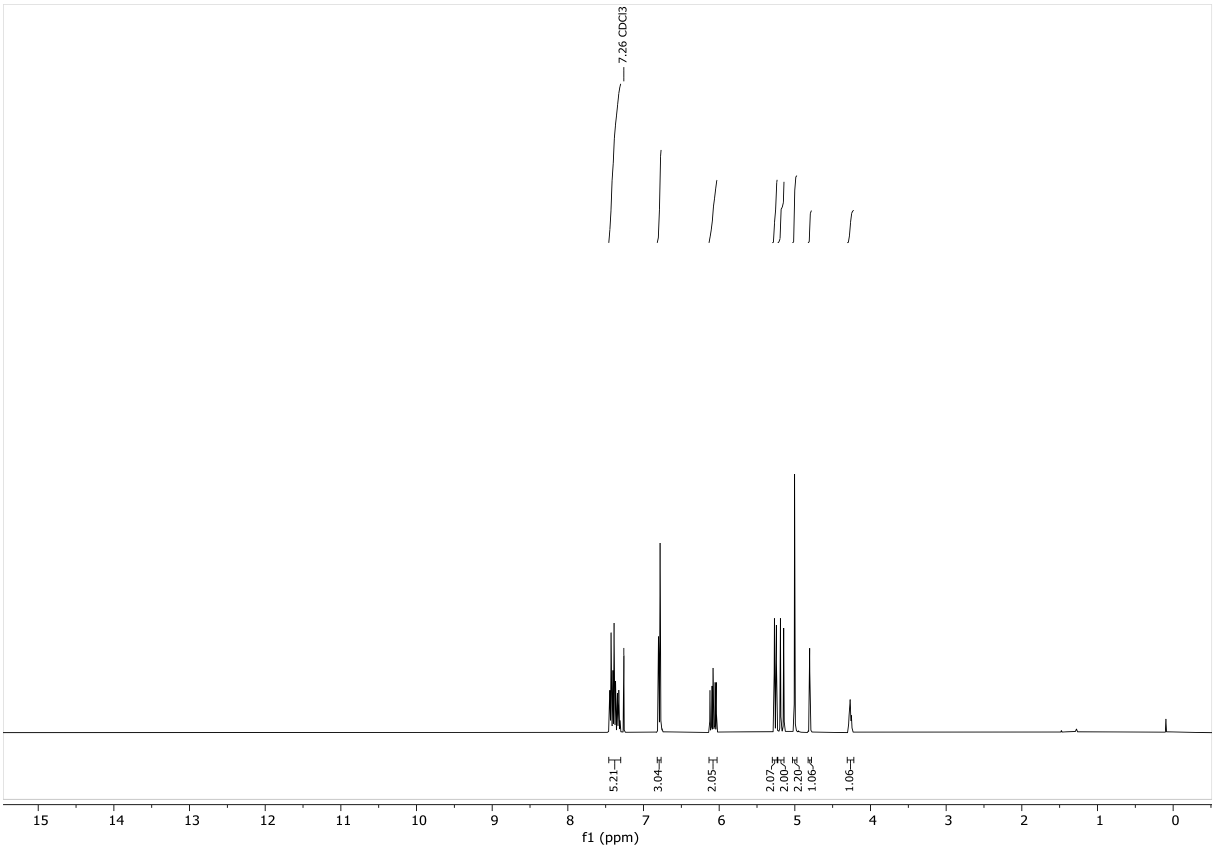

^{13}C-NMR (101 MHz, $CDCl_3$)

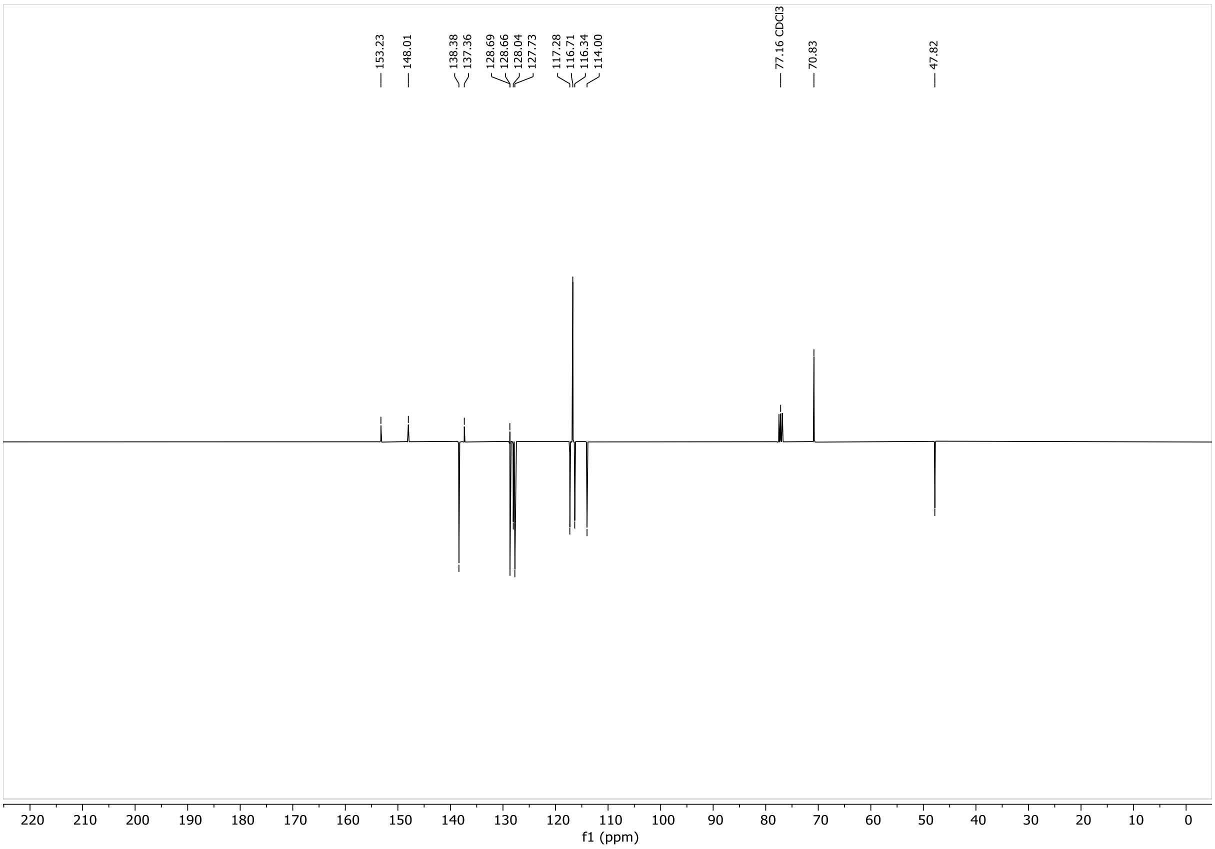

2-Chloro-4-methoxy-6-(penta-1,4-dien-3-yl)phenol (9g)

^{1}H NMR (400 MHz, $CDCl_3$)

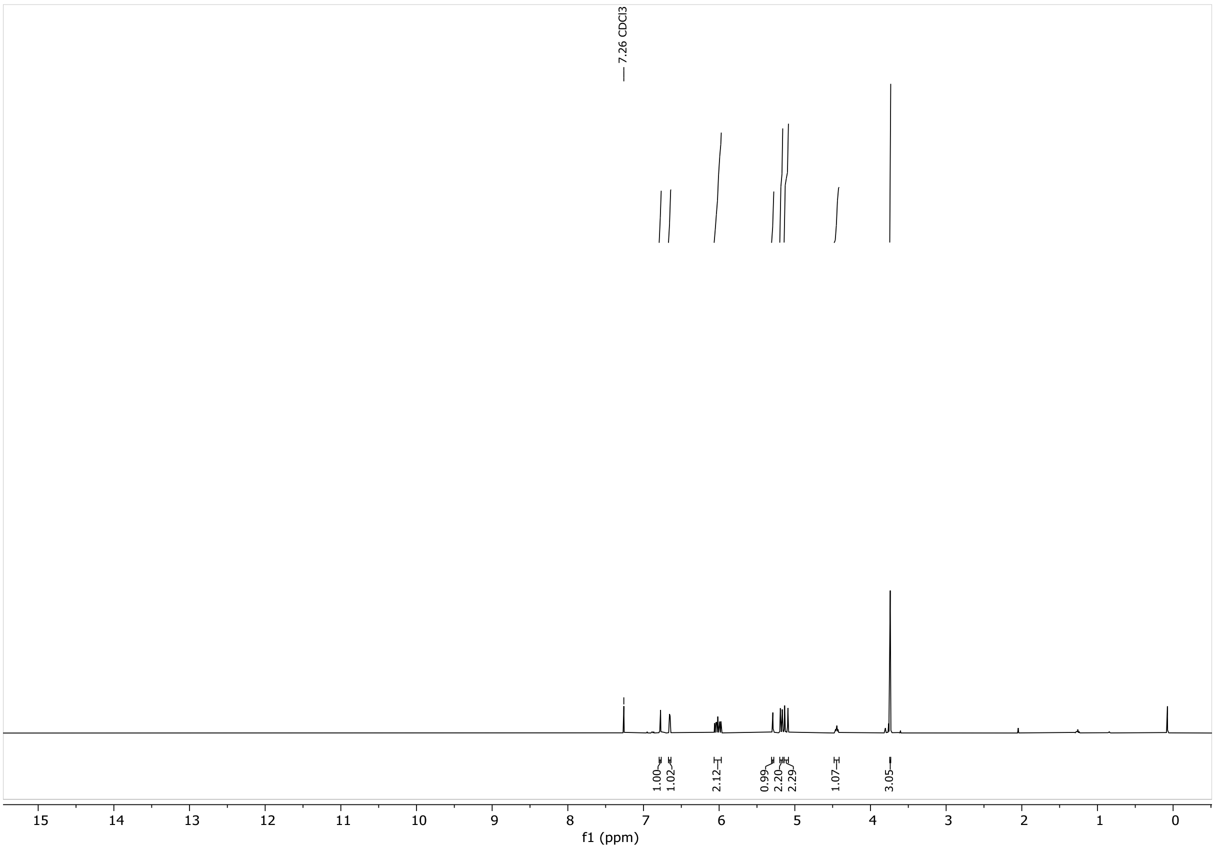

^{13}C-NMR (101 MHz, $CDCl_3$)

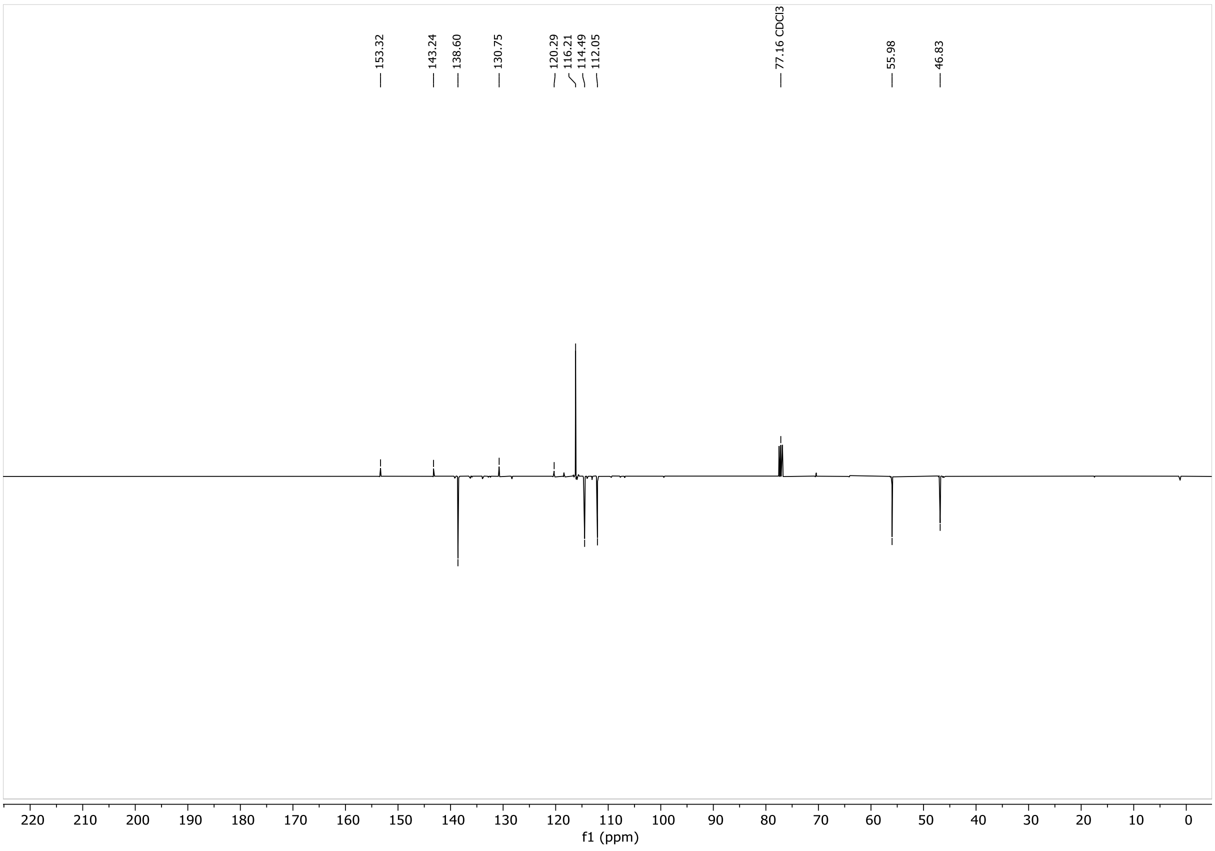

4-Chloro-2-(penta-1,4-dien-3-yl)phenol (9h)

^{1}H NMR (400 MHz, $CDCl_3$)

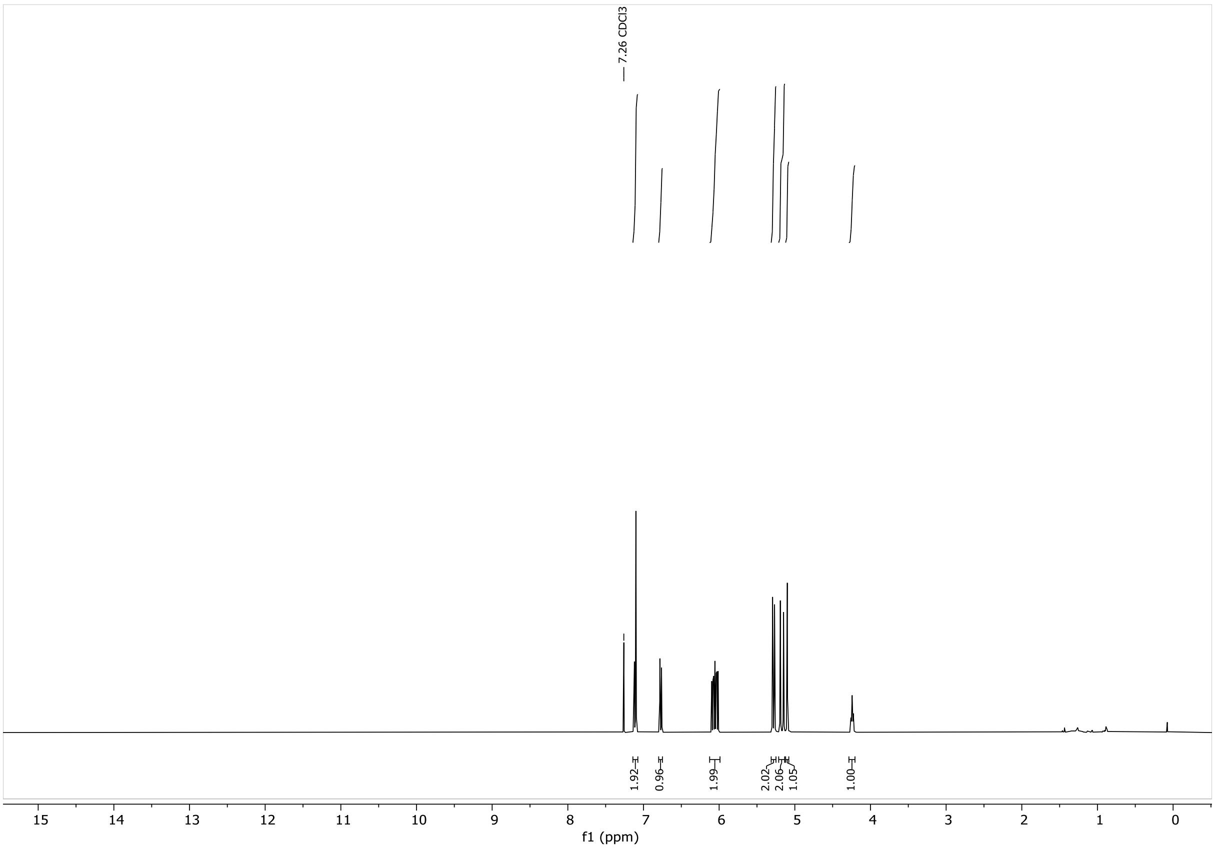

^{13}C-NMR (101 MHz, $CDCl_3$)

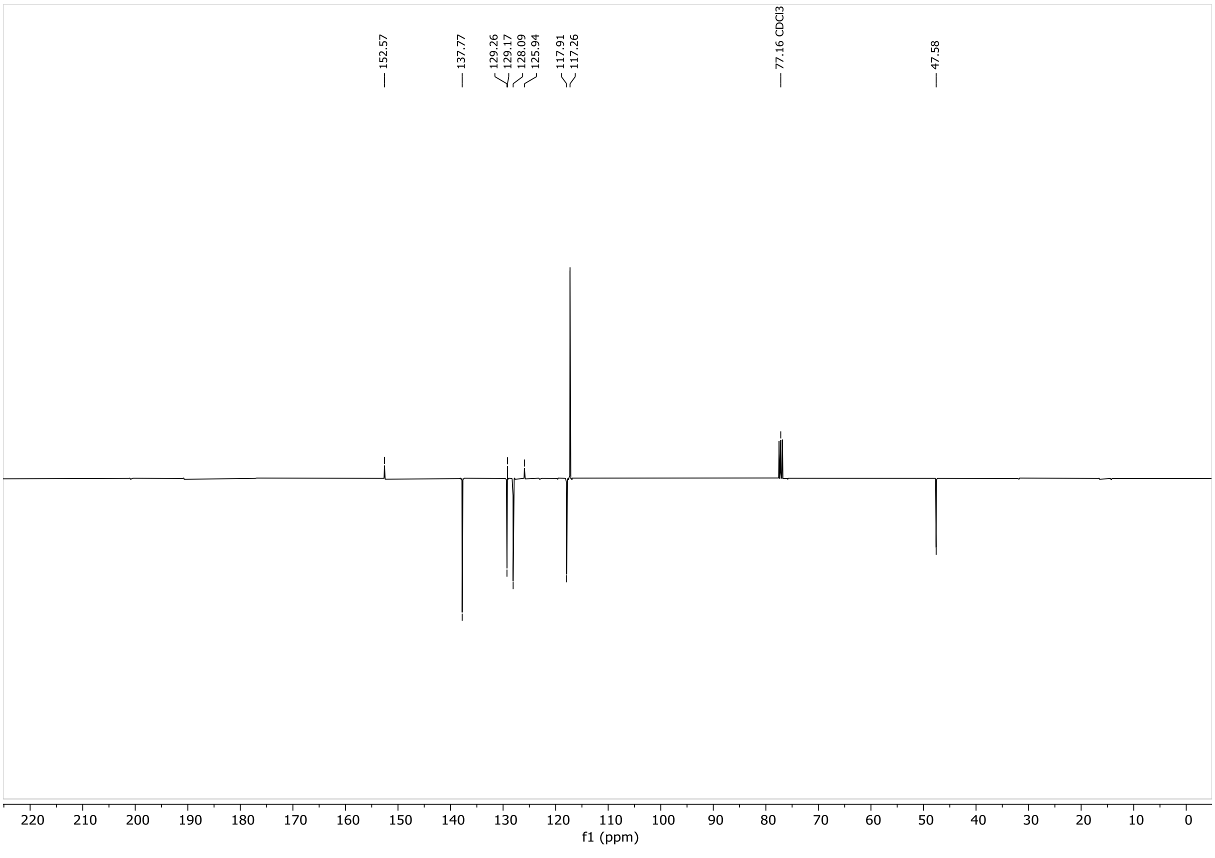

4-Hydroxy-3-methoxy-5-(penta-1,4-dien-3-yl)benzaldehyde (9i)

^{1}H NMR (400 MHz, $CDCl_3$)

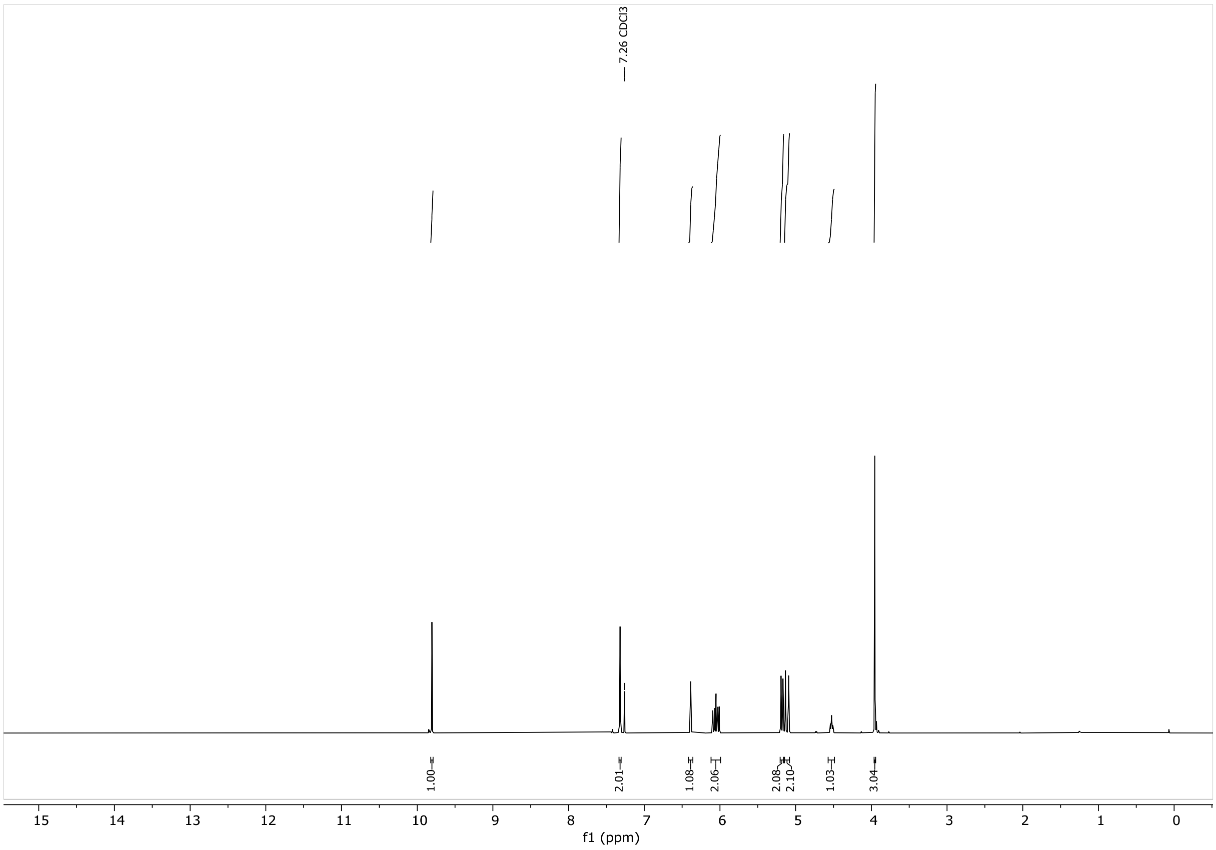

^{13}C-NMR (101 MHz, $CDCl_3$)

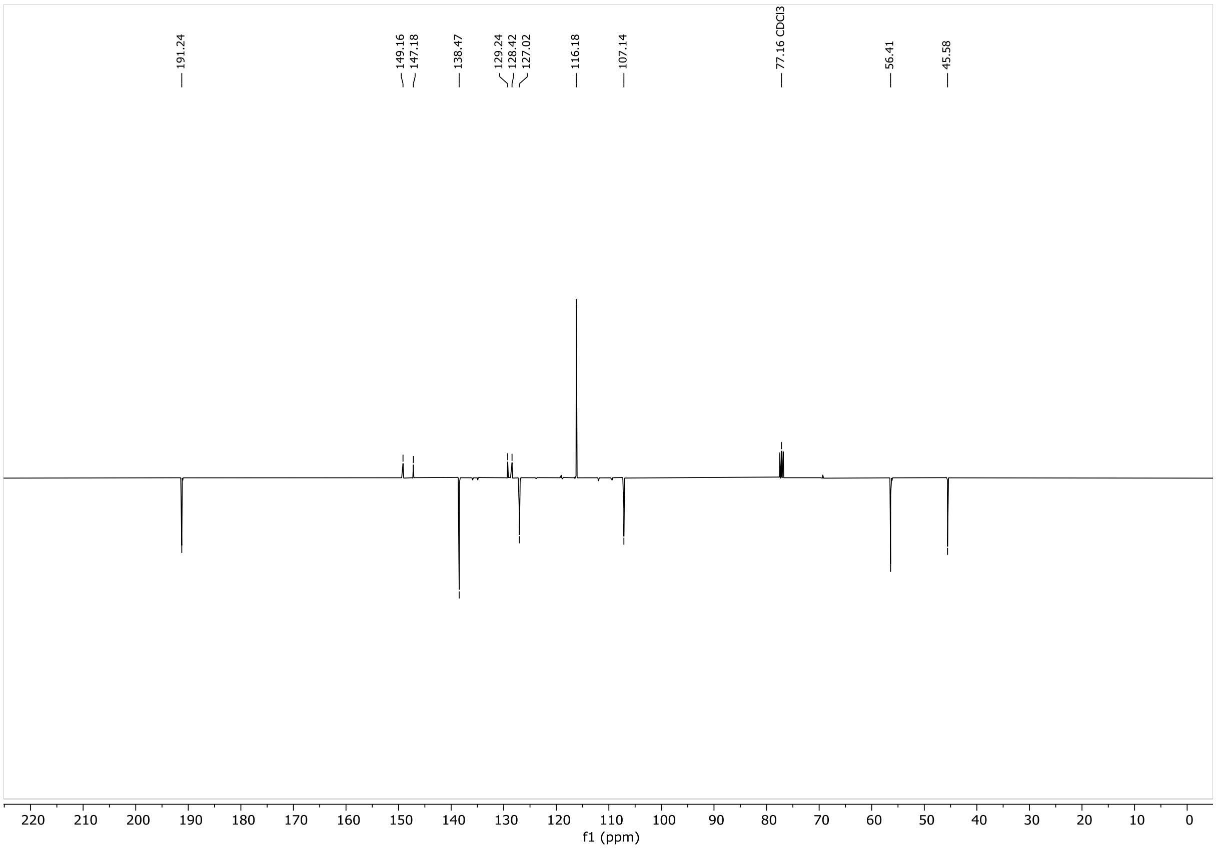

4-Hydroxy-3-methoxy-5-(penta-1,4-dien-3-yl)benzonitrile(9j)

^{1}H NMR (400 MHz, $CDCl_3$)

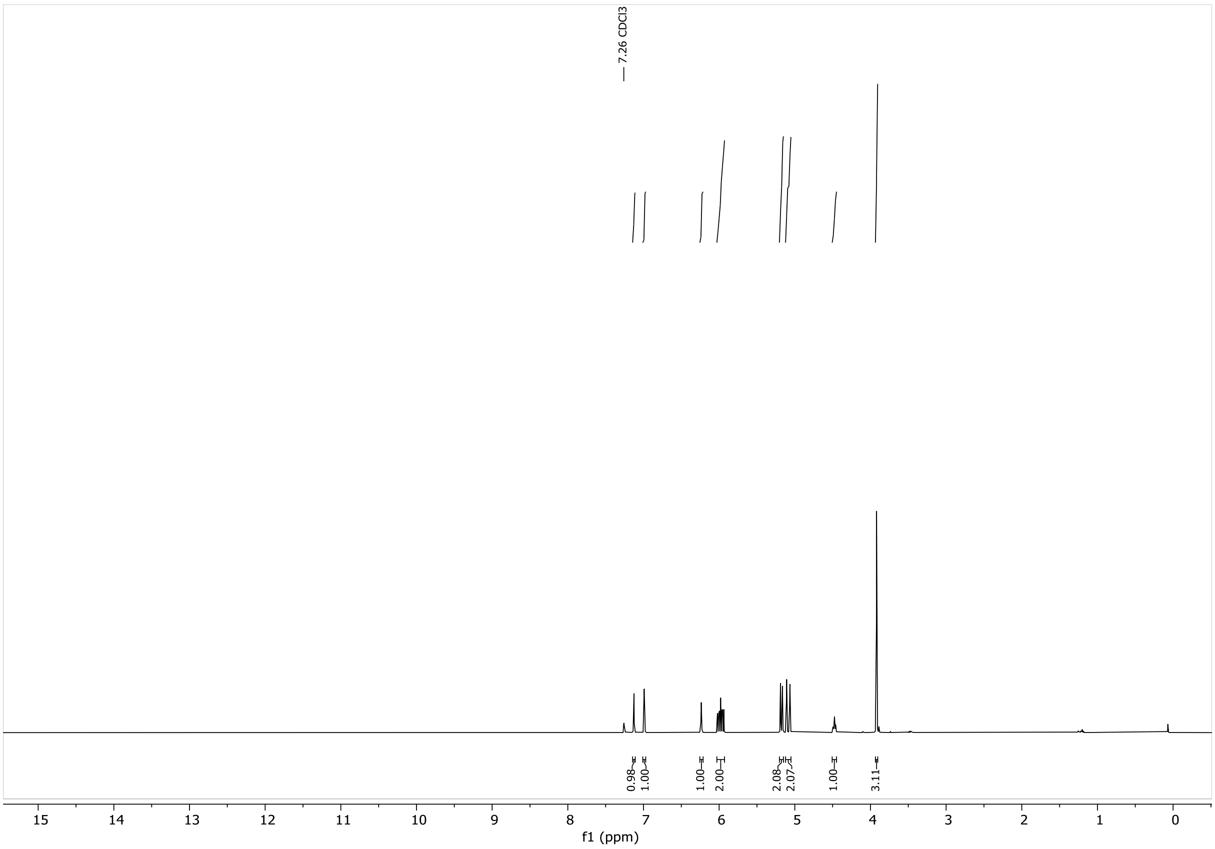

^{13}C-NMR (101 MHz, $CDCl_3$)

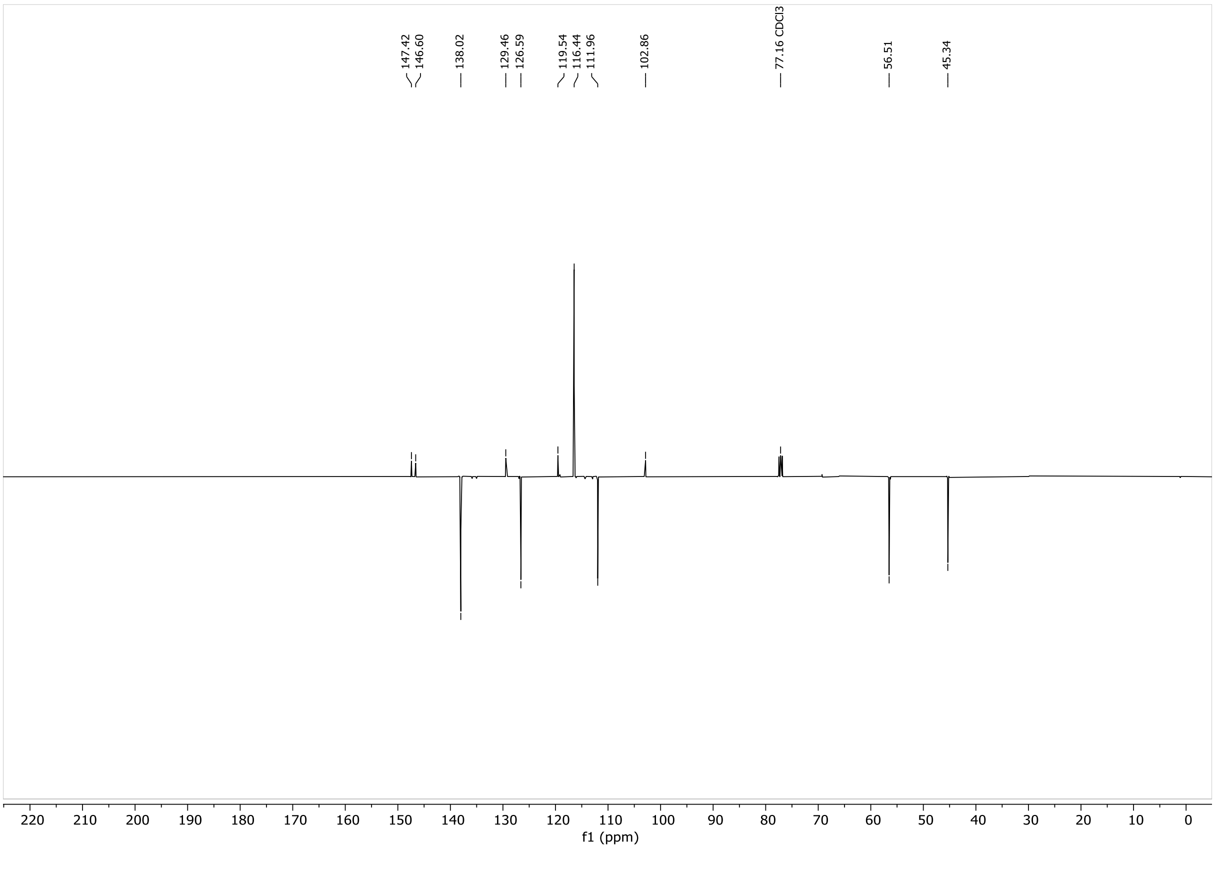

(*E*)-4-(penta-2,4-dien-1-yl)phenol (11a)

^{1}H NMR (400 MHz, $CDCl_3$)

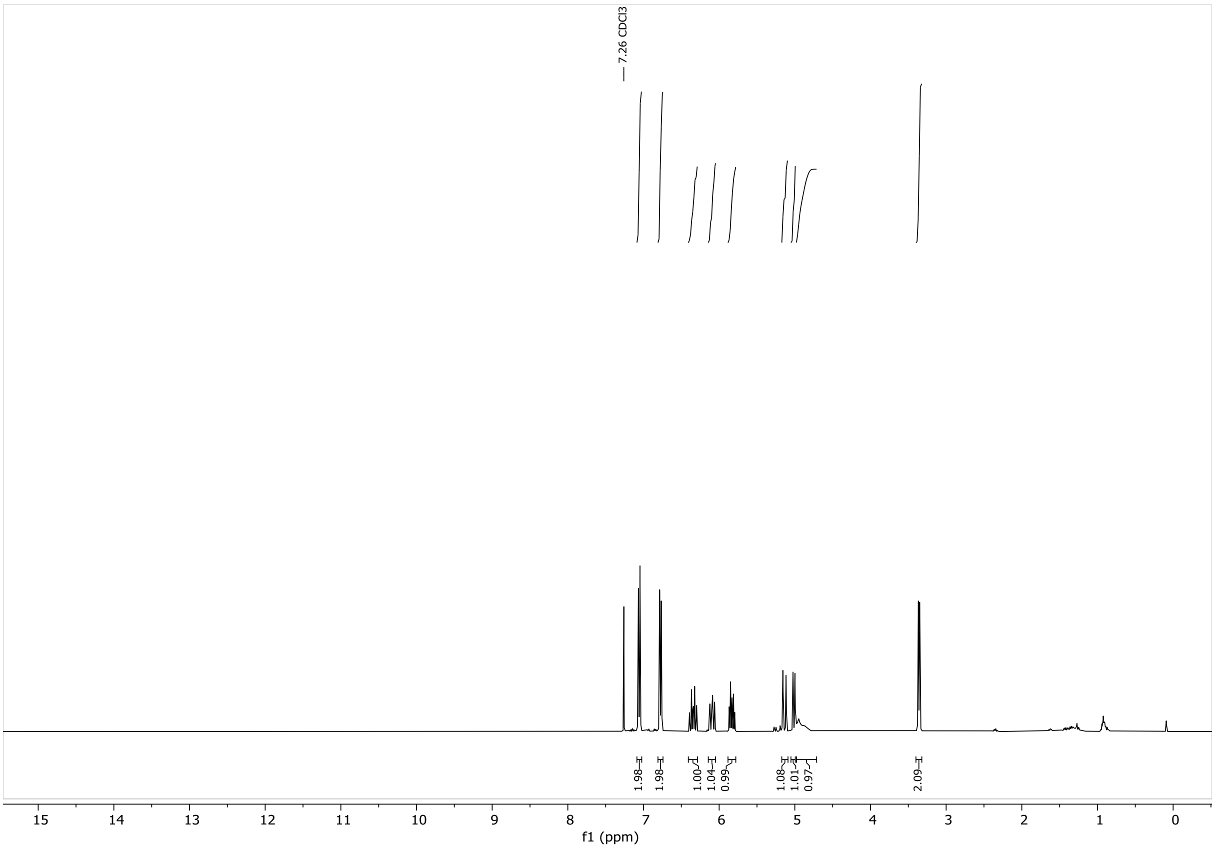

^{13}C-NMR (101 MHz, $CDCl_3$)

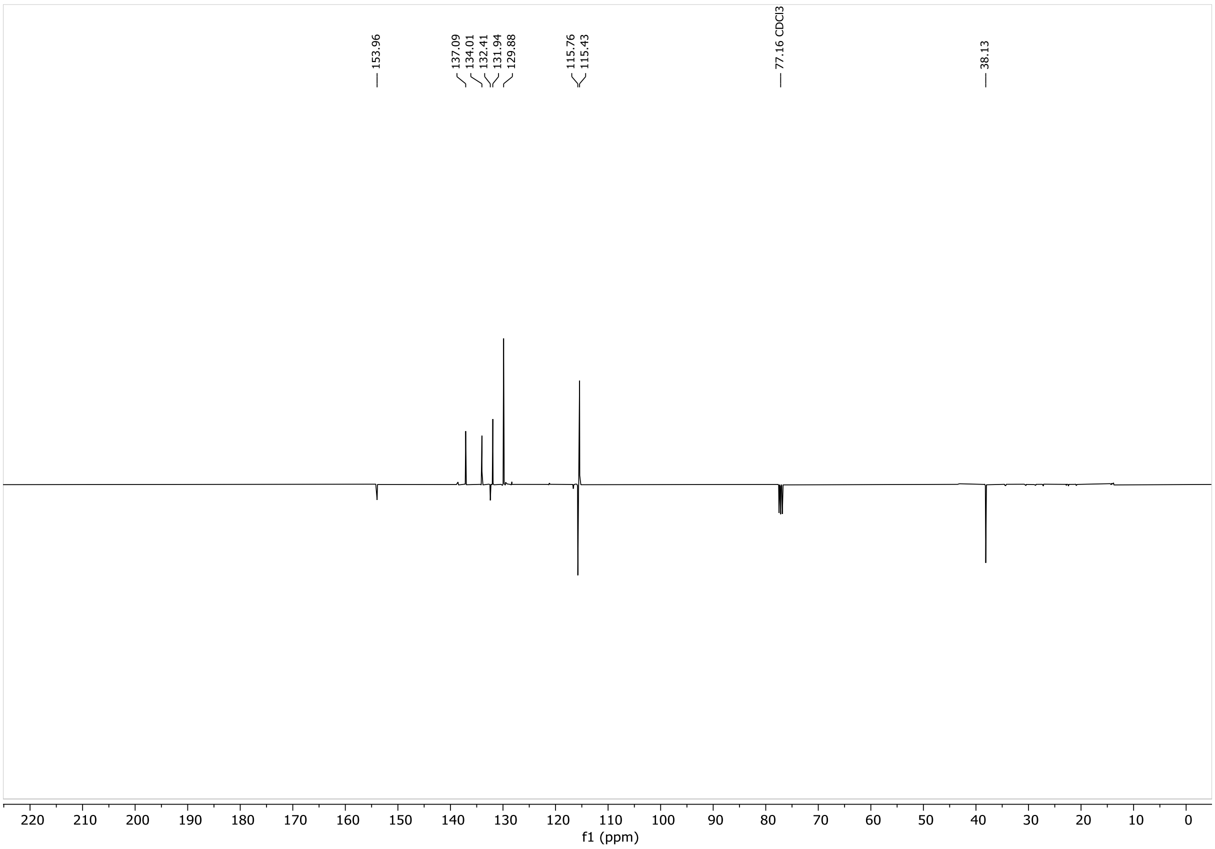

(*E*)-2-methyl-4-(penta-2,4-dien-1-yl)phenol (11b)

^{1}H NMR (400 MHz, $CDCl_3$)

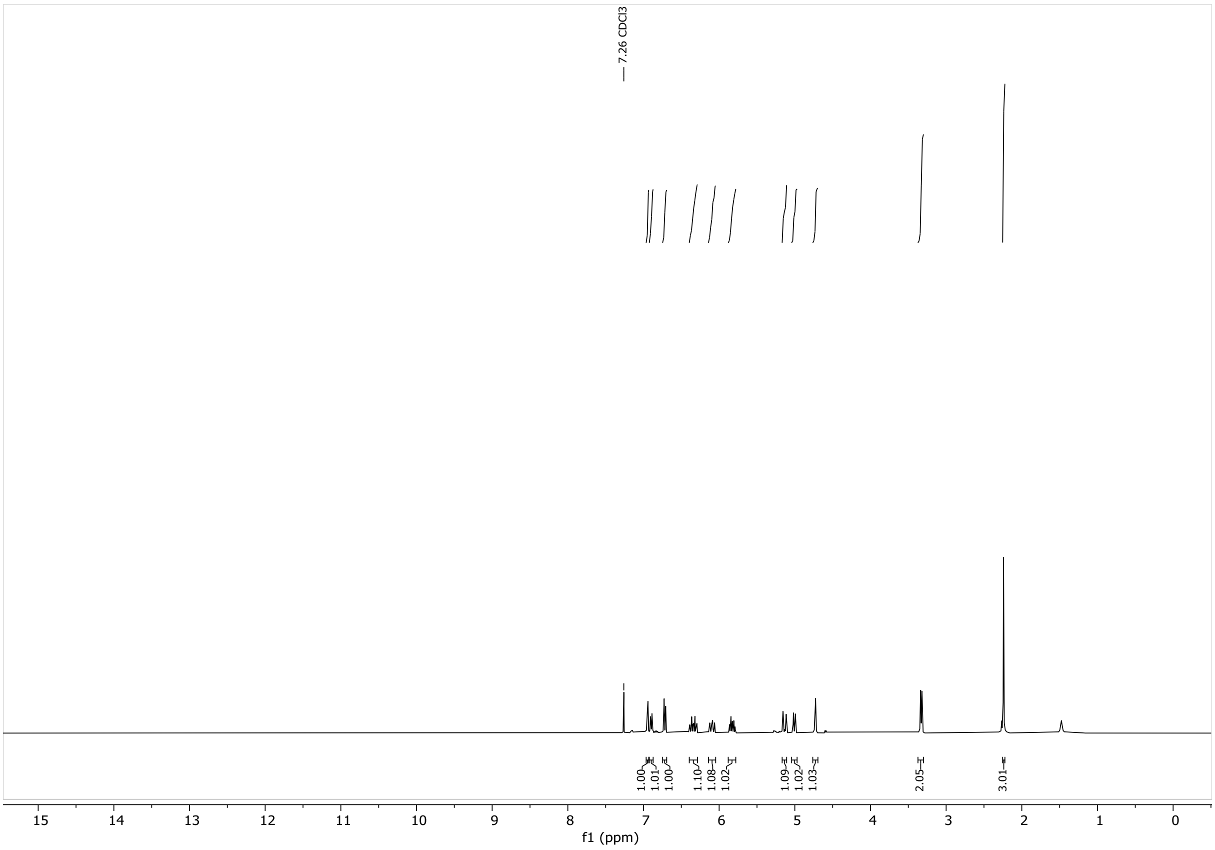

^{13}C-NMR (101 MHz, $CDCl_3$)

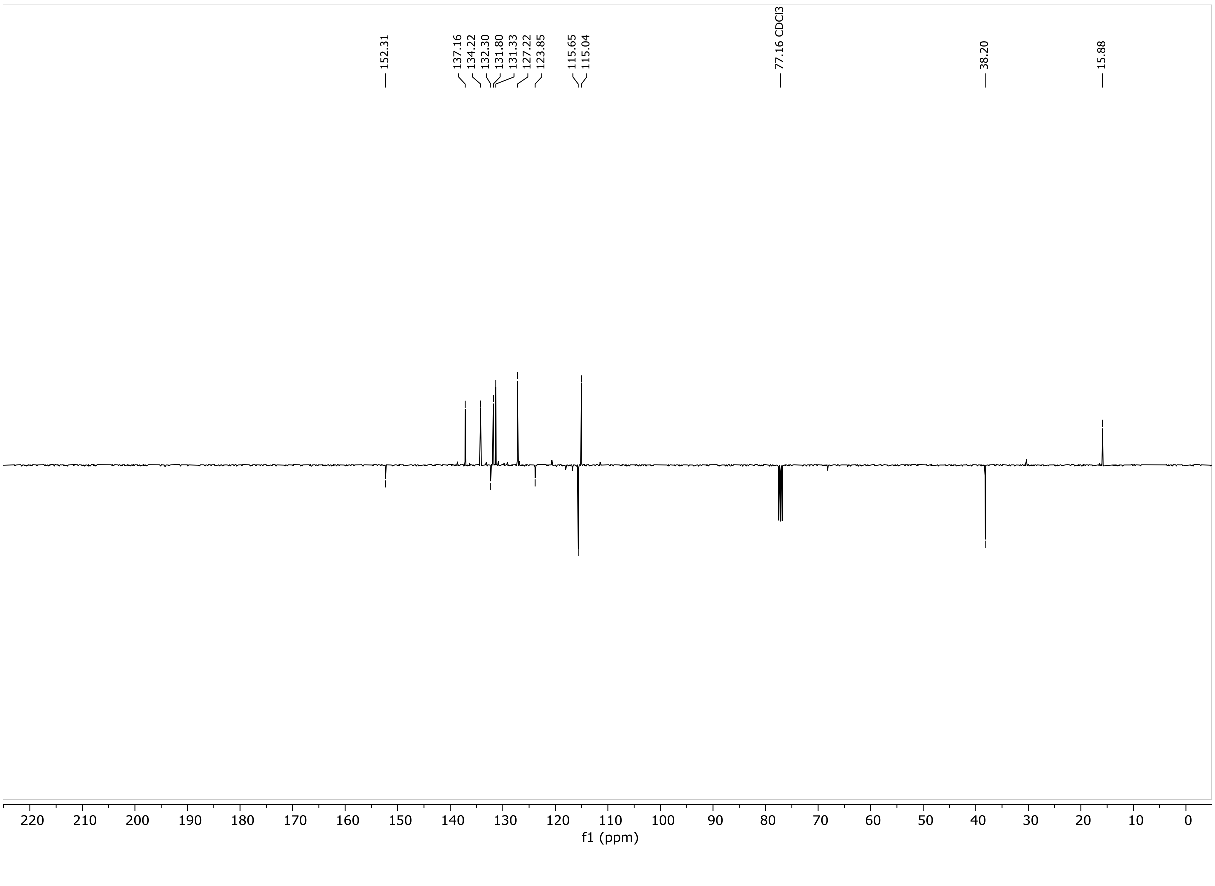

(*E*)-3-methyl-4-(penta-2,4-dien-1-yl)phenol (11c)

^{1}H NMR (400 MHz, $CDCl_3$)

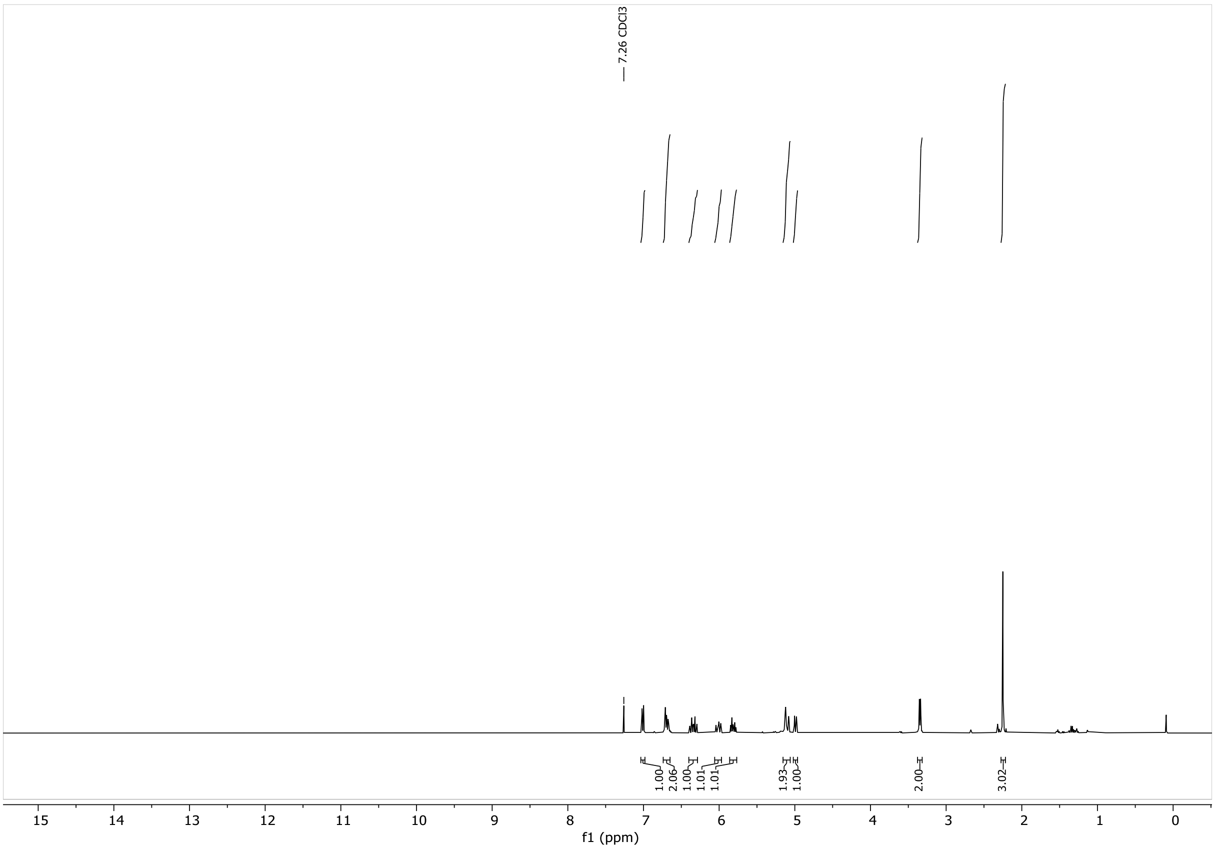

^{13}C-NMR (101 MHz, $CDCl_3$)

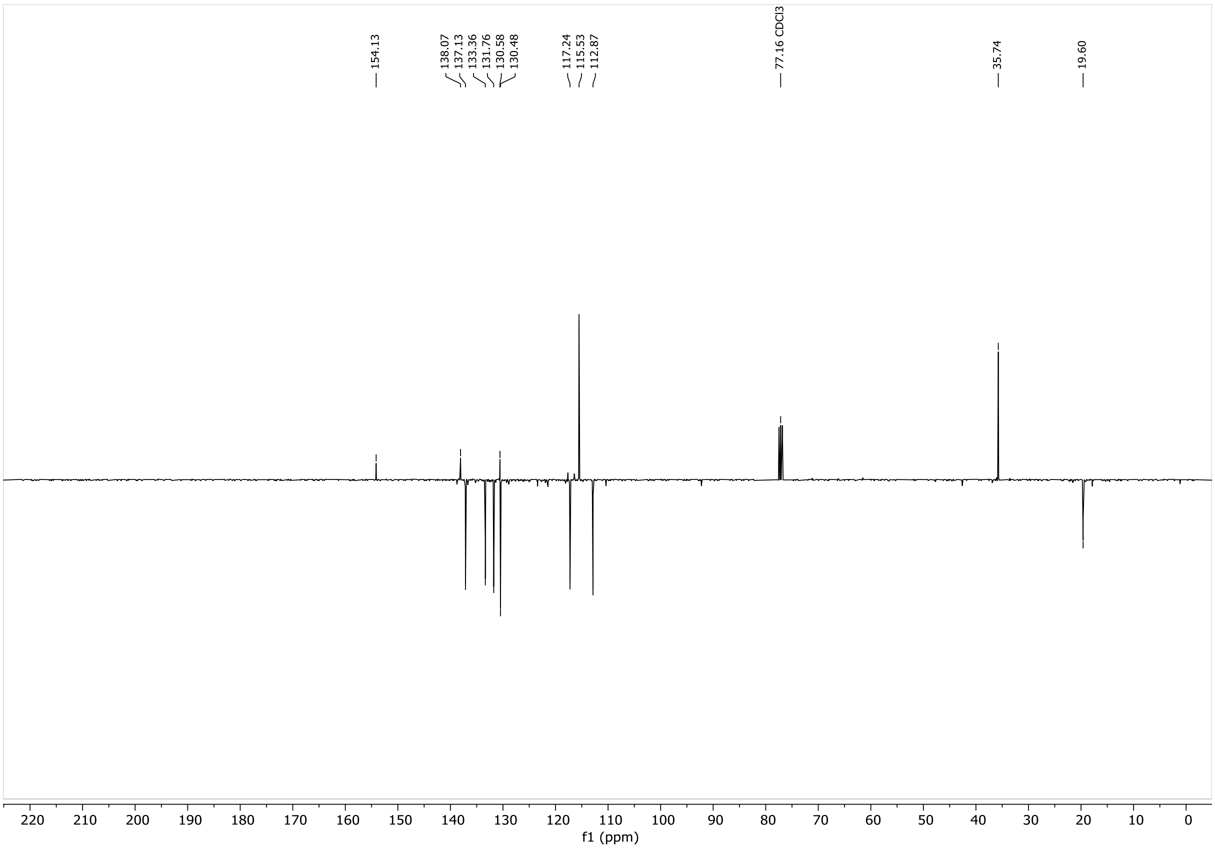

(*E*)-2-isopropyl-5-methyl-4-(penta-2,4-dien-1-yl)phenol (11d)

^{1}H NMR (400 MHz, $CDCl_3$)

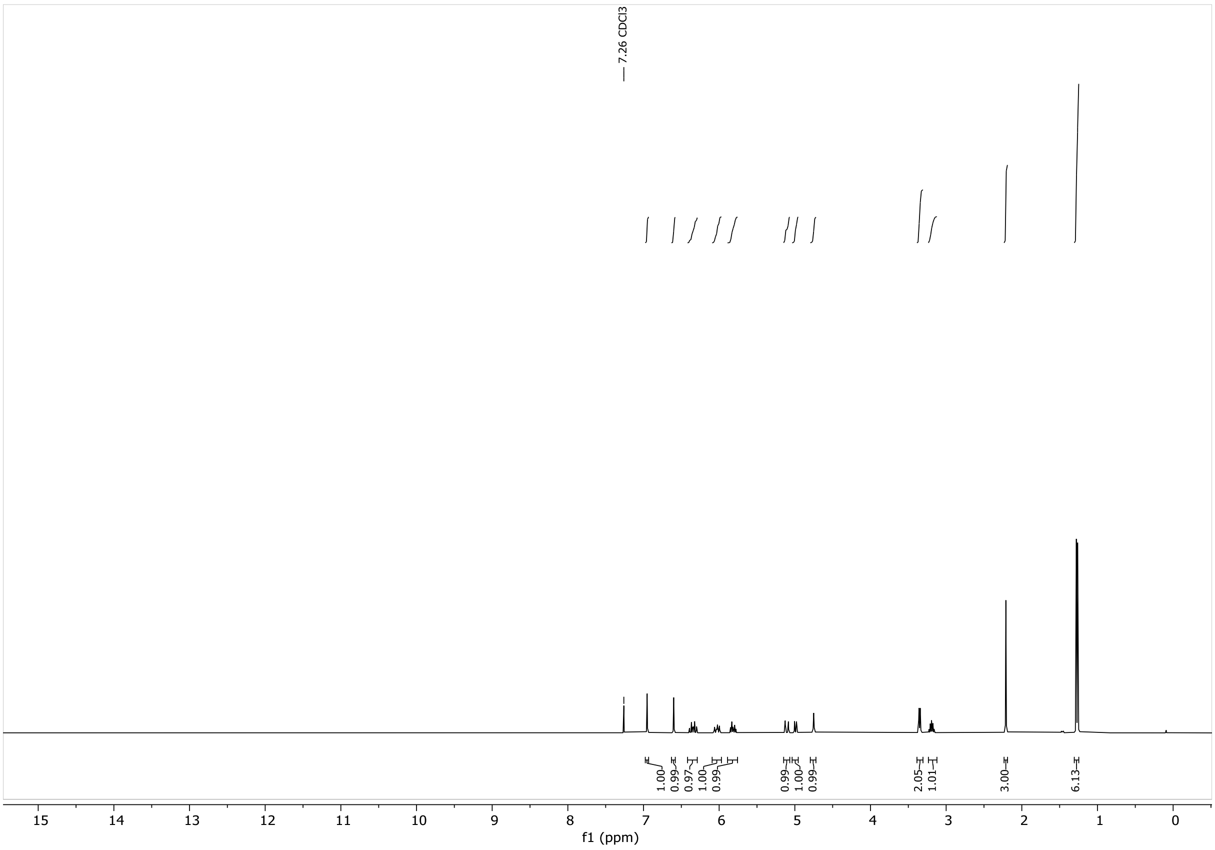

^{13}C-NMR (101 MHz, $CDCl_3$)

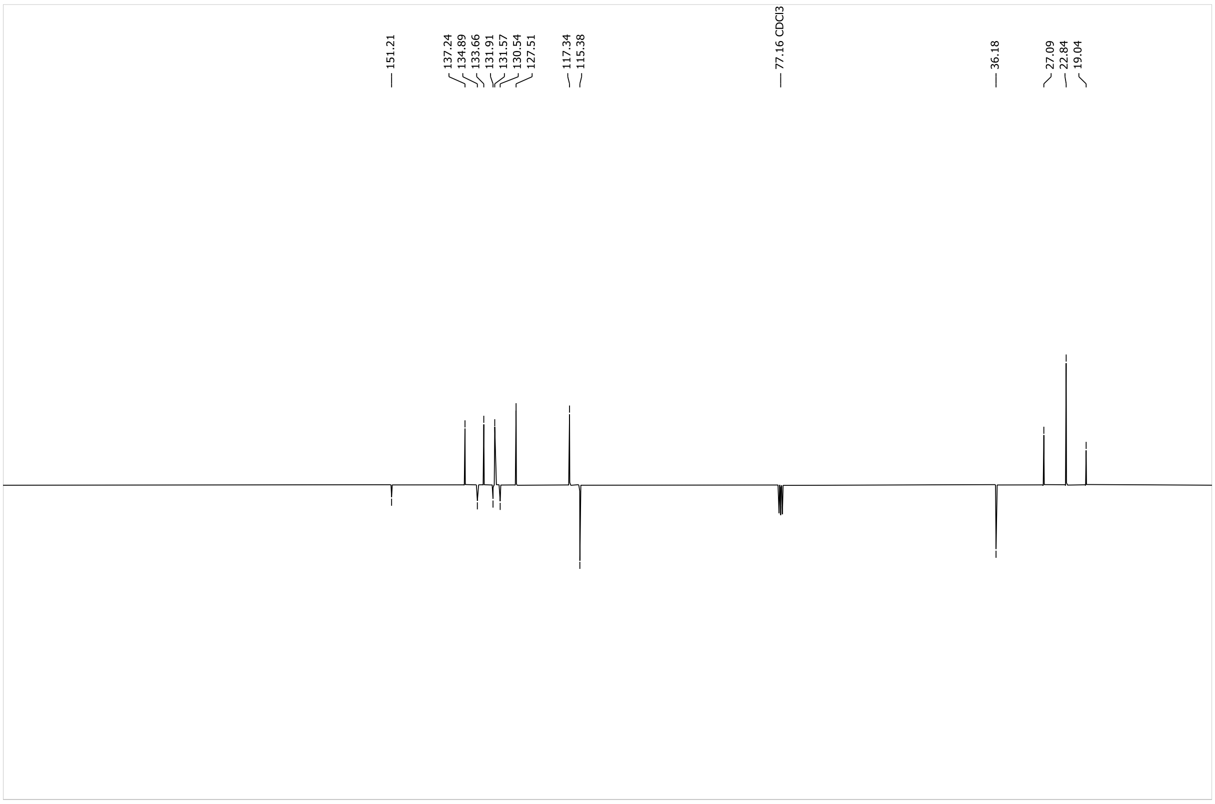

(*E*)-2,6-Dimethyl-4-(penta-2,4-dien-1-yl)phenol (11e)

^{1}H NMR (400 MHz, $CDCl_3$)

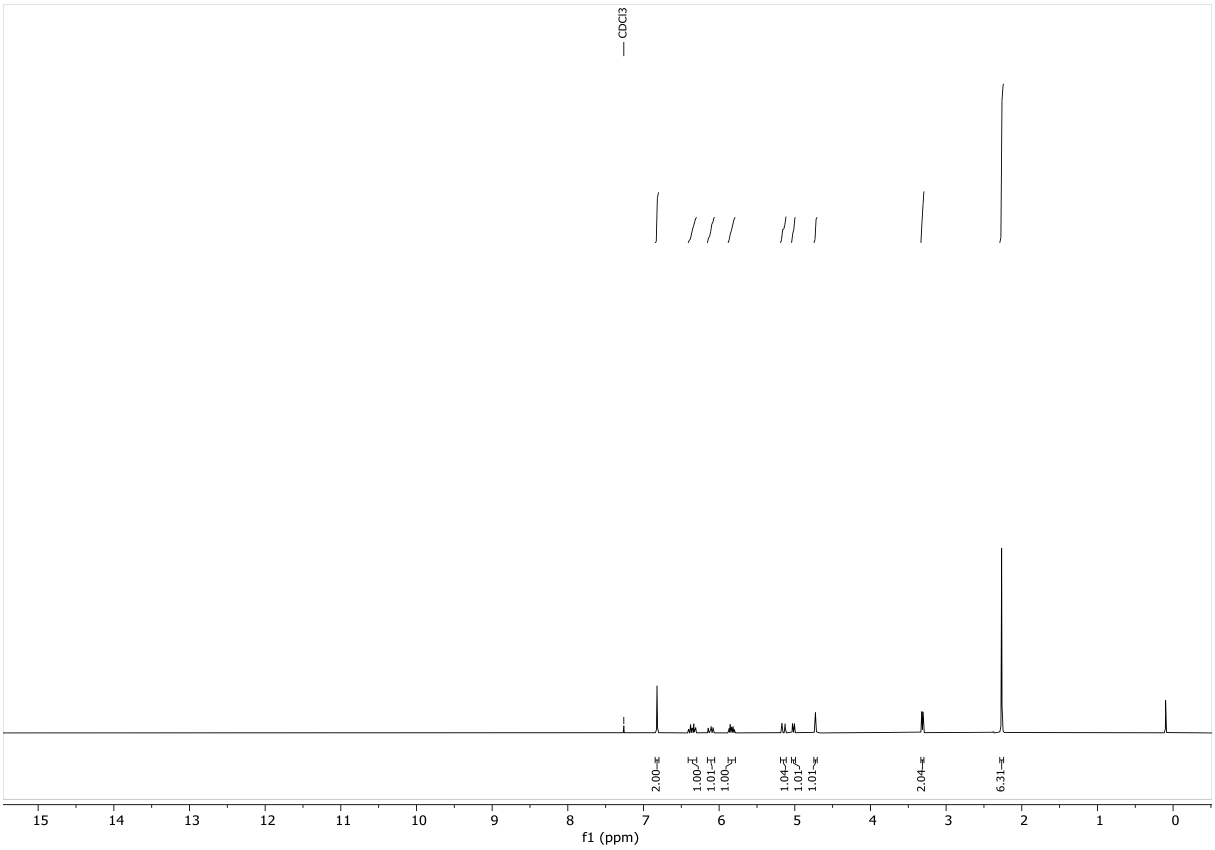

^{13}C-NMR (101 MHz, $CDCl_3$)

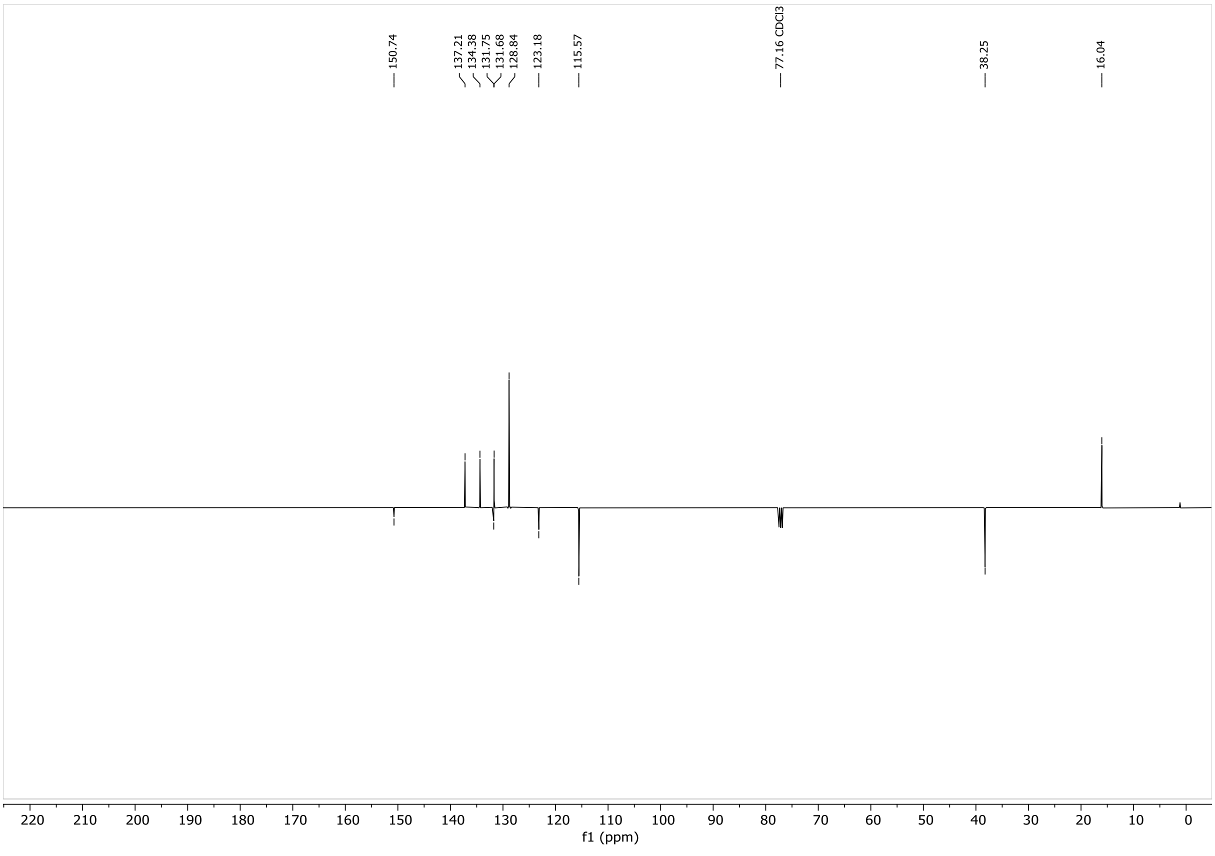

(*E*)-2-methoxy-4-(penta-2,4-dien-1-yl)phenol (11f)

^{1}H NMR (400 MHz, $CDCl_3$)

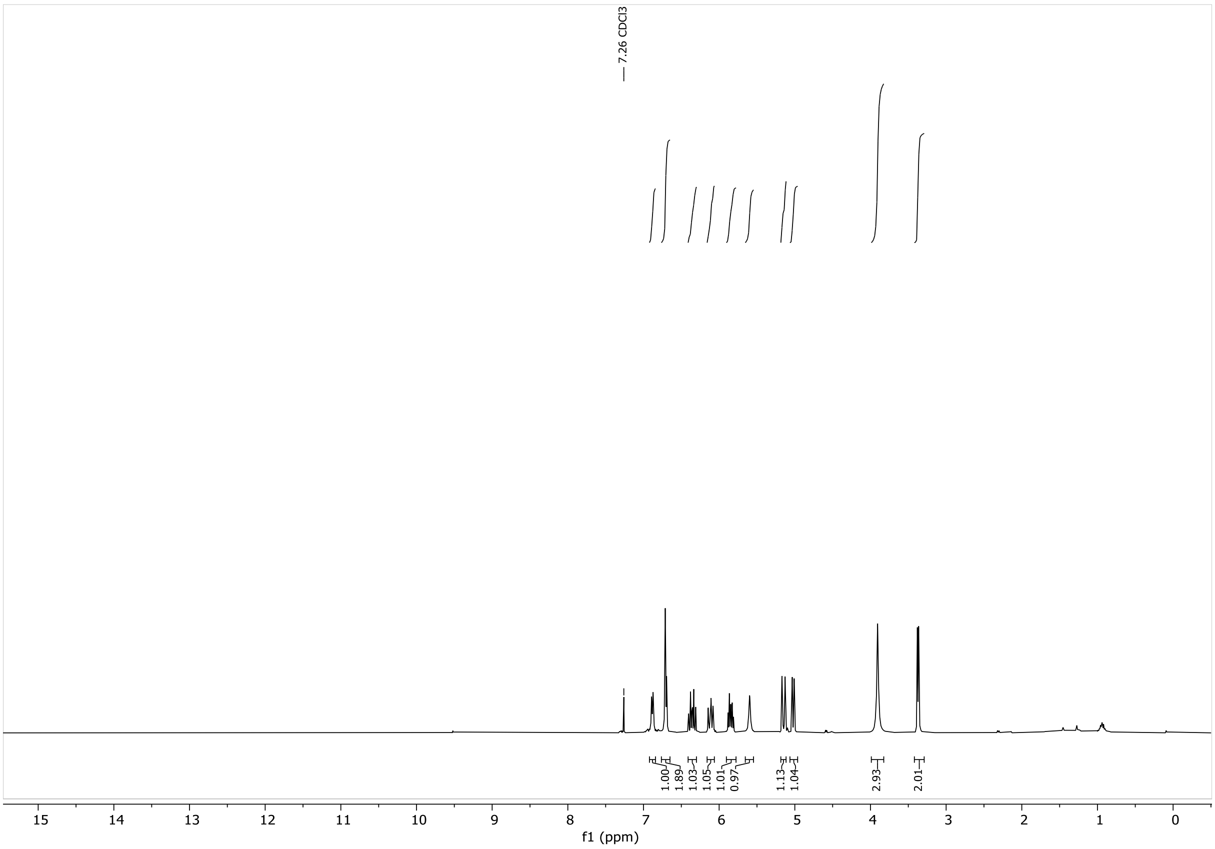

^{13}C-NMR (101 MHz, $CDCl_3$)

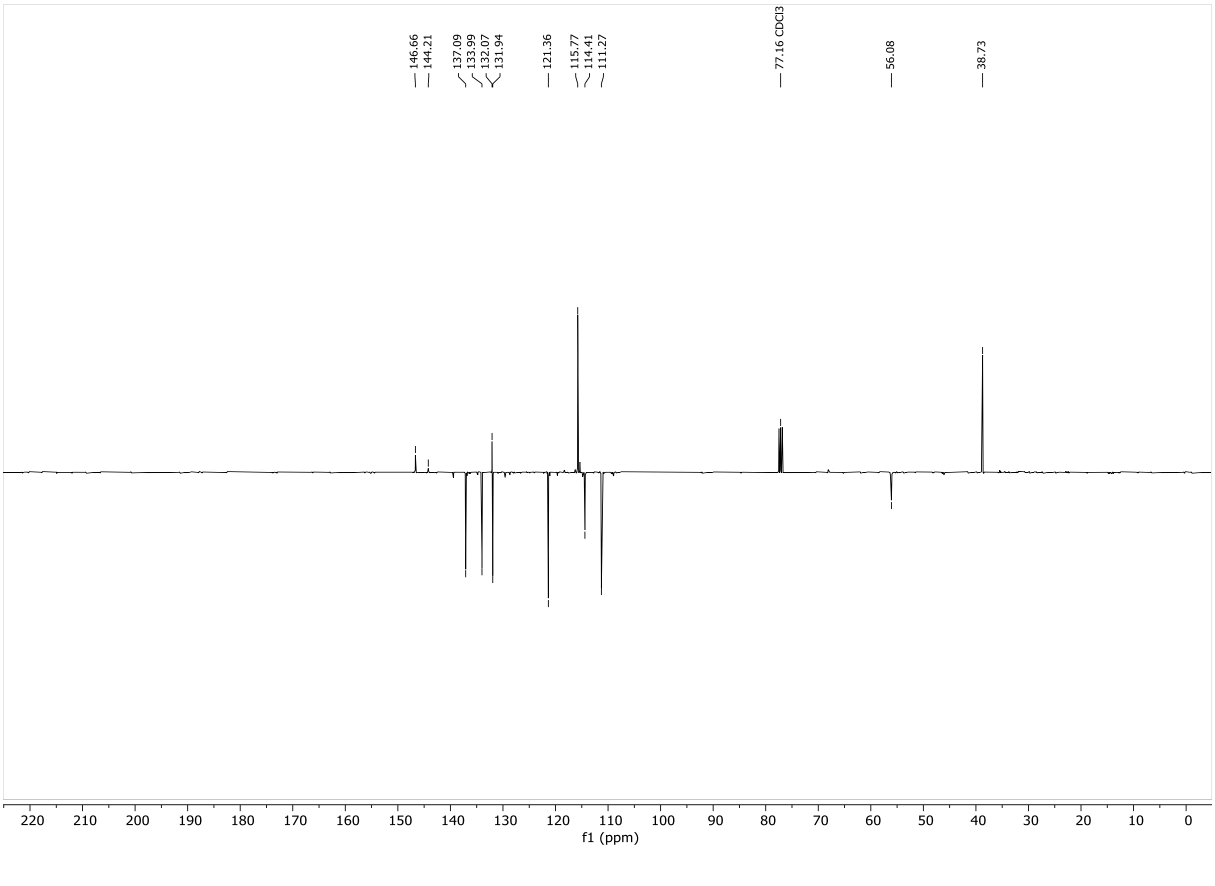

(*E*)-3-methoxy-4-(penta-2,4-dien-1-yl)phenol (11g)

^{1}H NMR (400 MHz, $CDCl_3$)

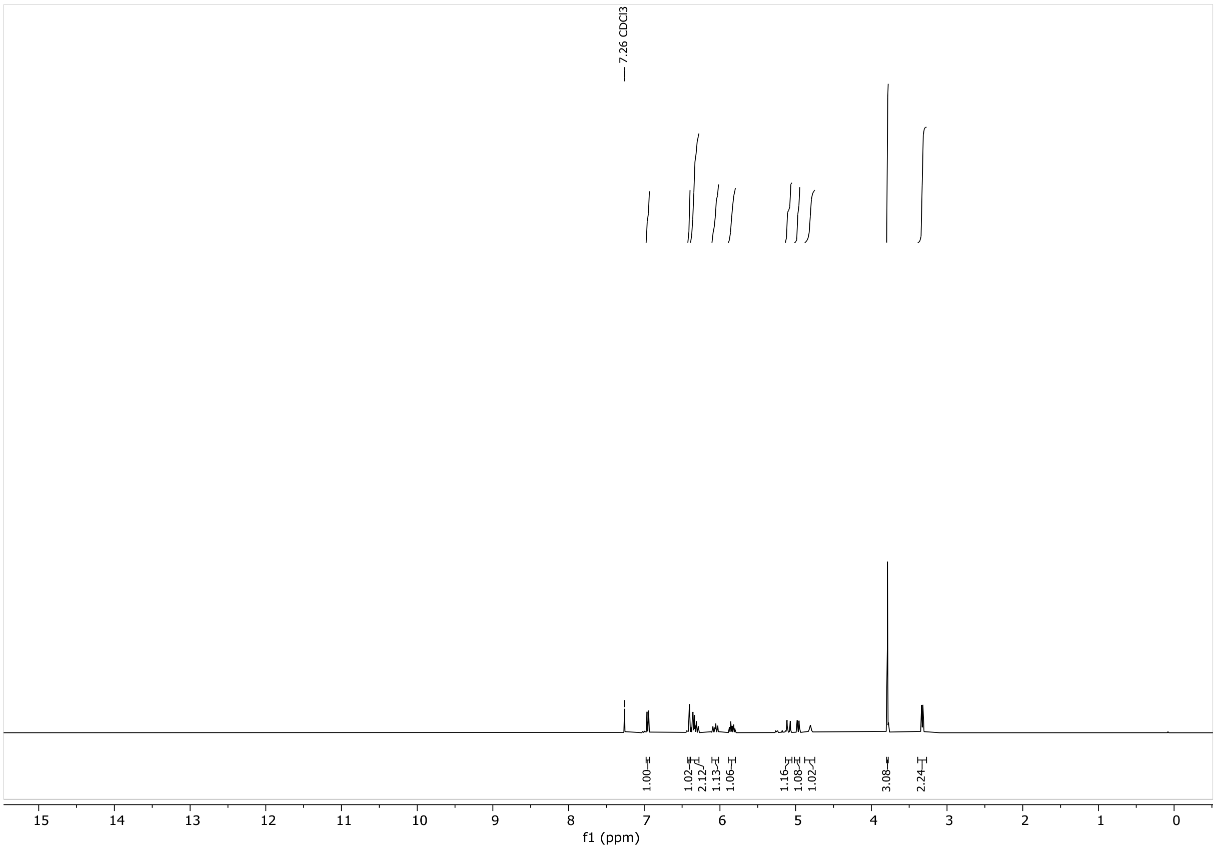

^{13}C-NMR (101 MHz, $CDCl_3$)

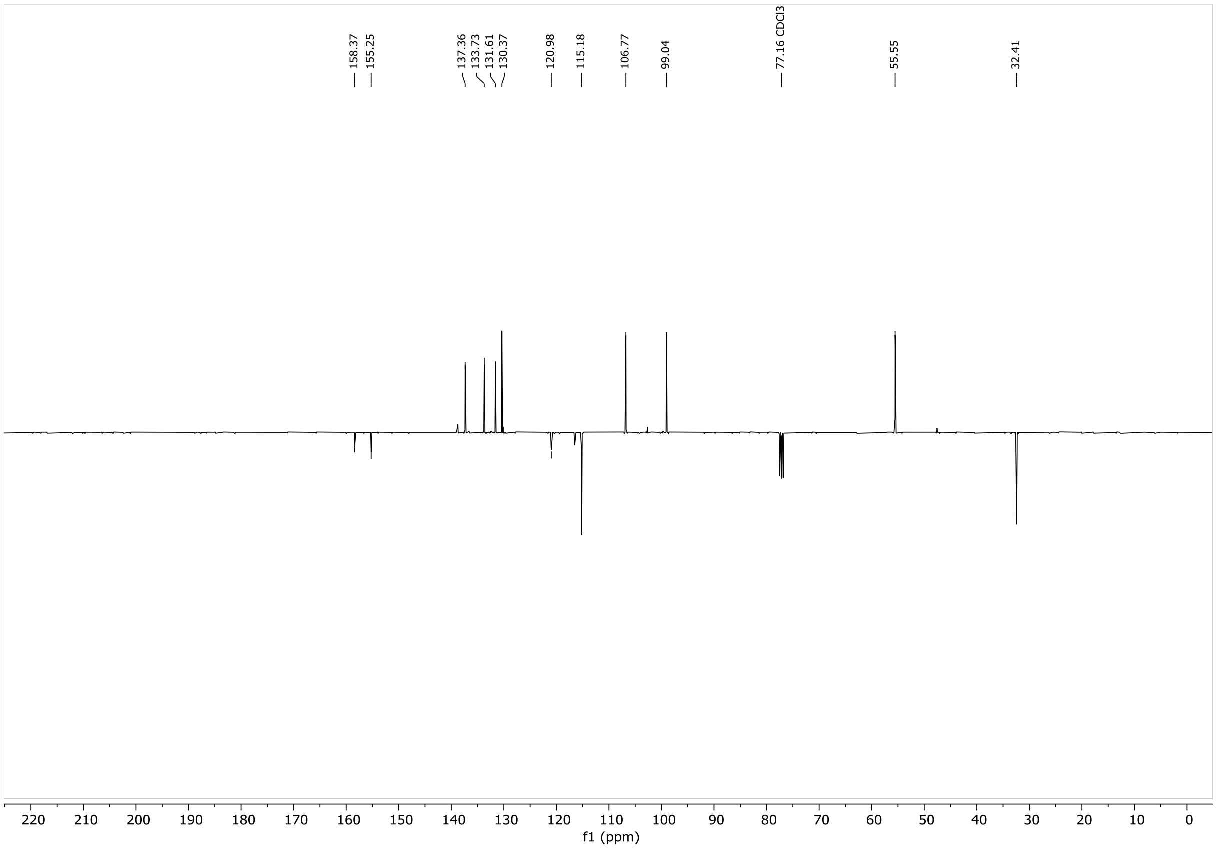

(*E*)-4-(penta-2,4-dien-1-yl)-3-((triisopropylsilyl)oxy)phenol (11h)

^{1}H NMR (400 MHz, $CDCl_3$)

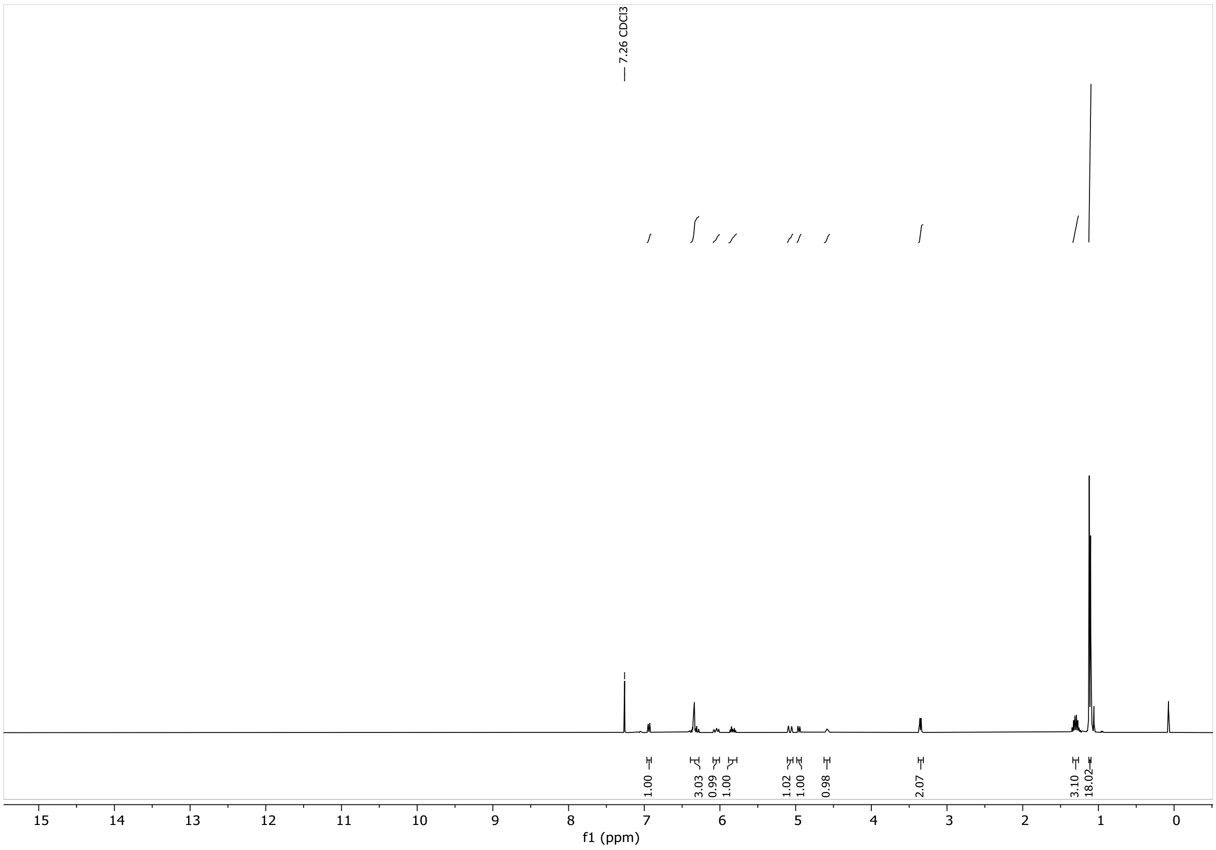

^{13}C-NMR (101 MHz, $CDCl_3$)

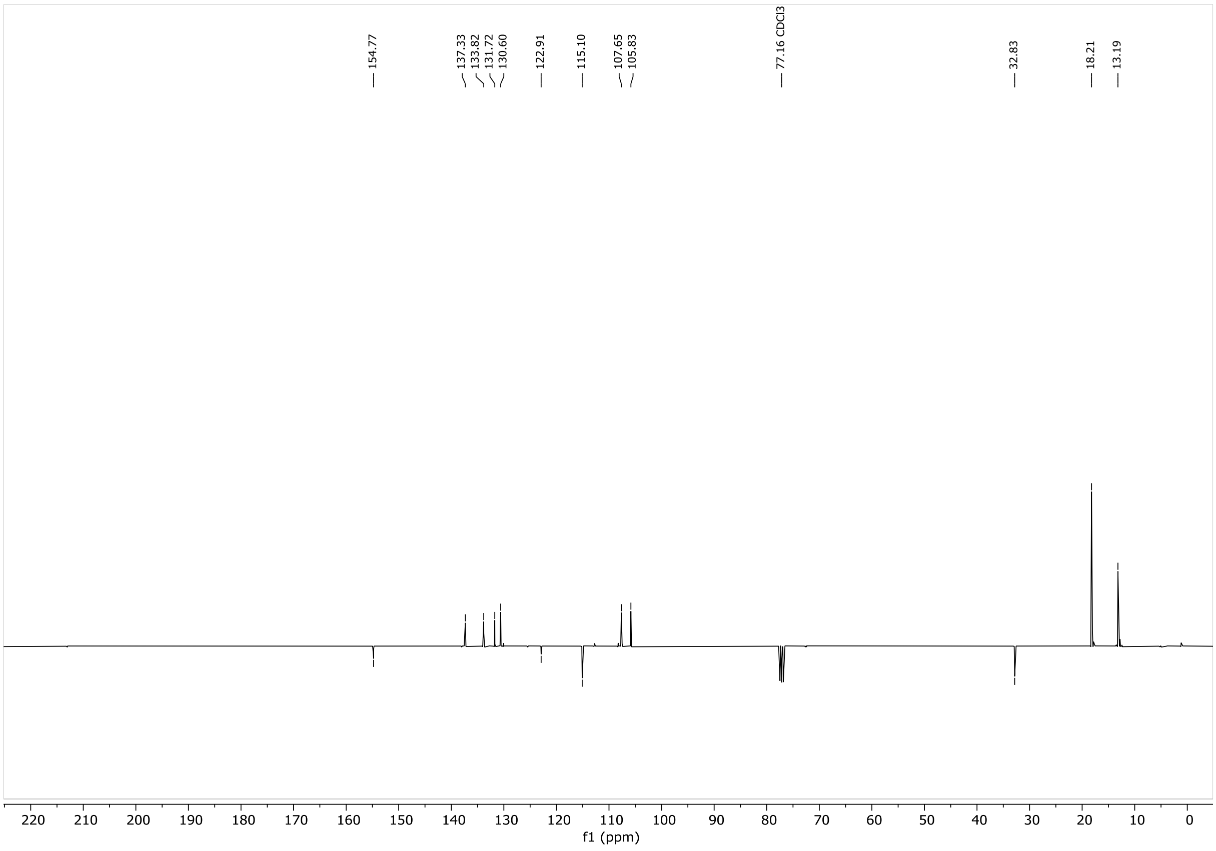

(*E*)-2-(benzyloxy)-4-(penta-2,4-dien-1-yl)phenol (11i)

^{1}H NMR (400 MHz, $CDCl_3$)

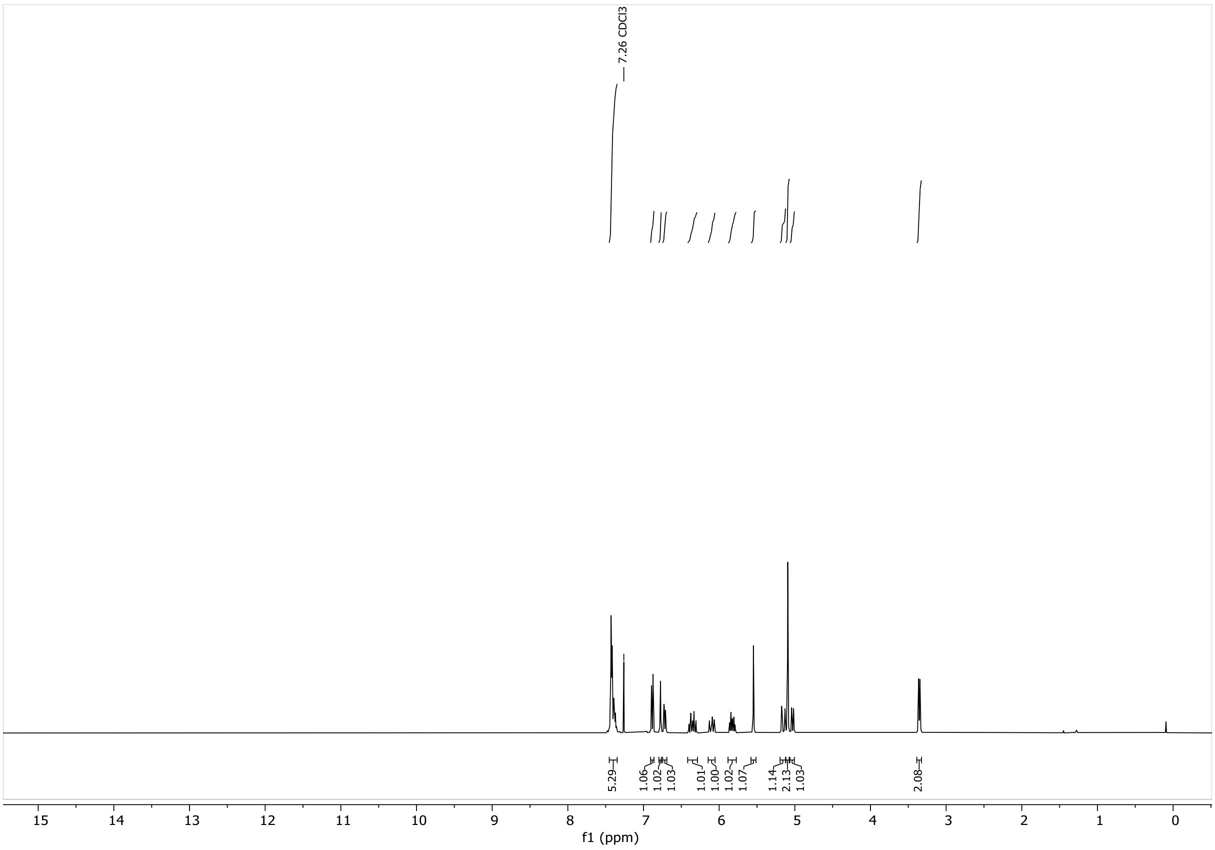

^{13}C-NMR (101 MHz, $CDCl_3$)

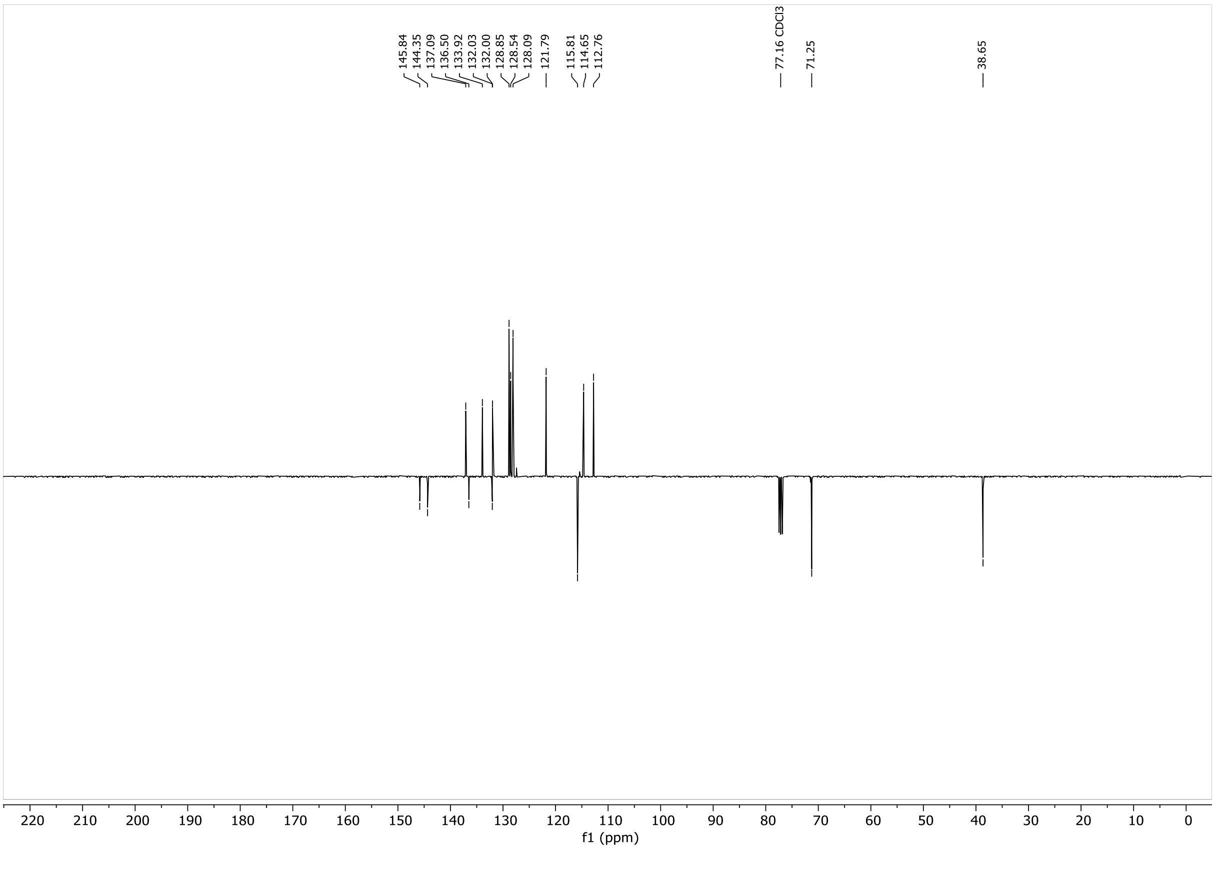

(*E*)-5-(1,3-dioxolan-2-yl)-2-methoxy-4-(penta-2,4-dien-1-yl)phenol (11j)

^{1}H NMR (400 MHz, $CDCl_3$)

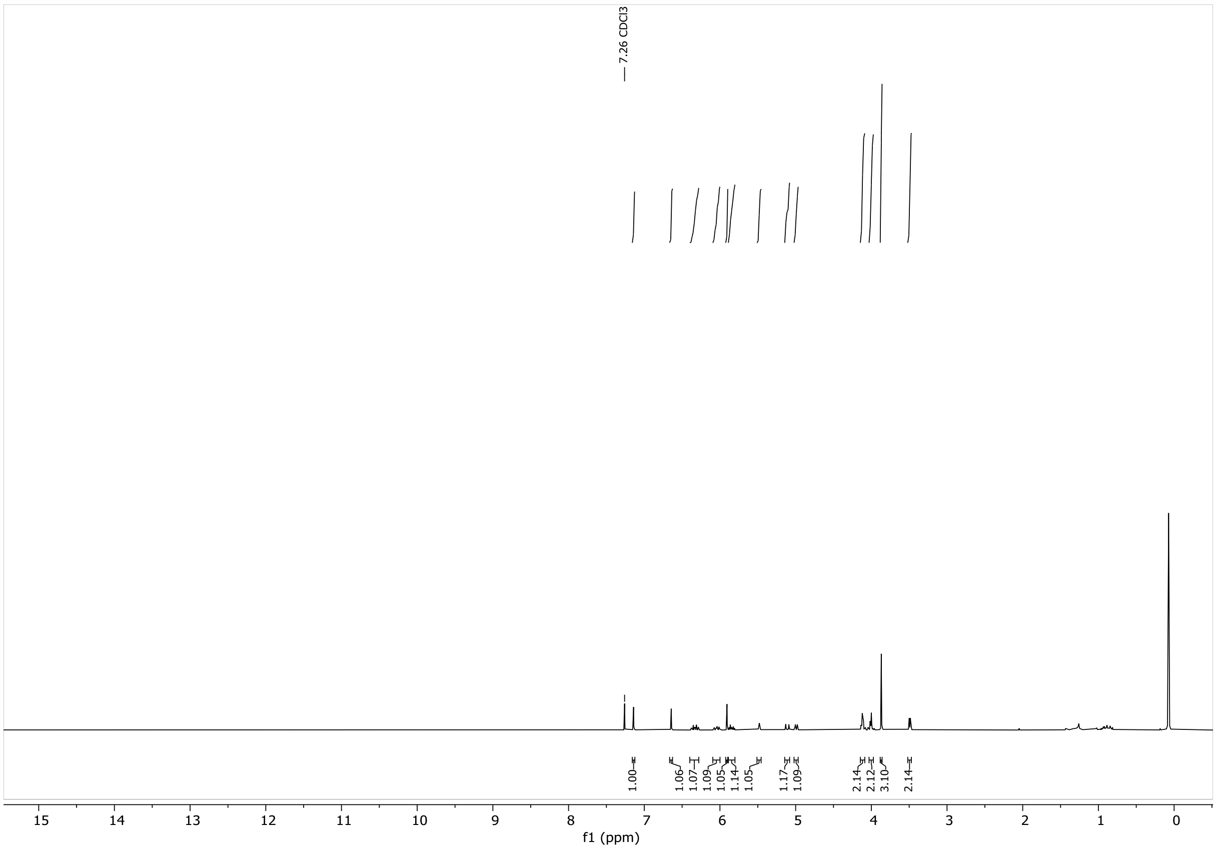

^{13}C-NMR (101 MHz, $CDCl_3$)

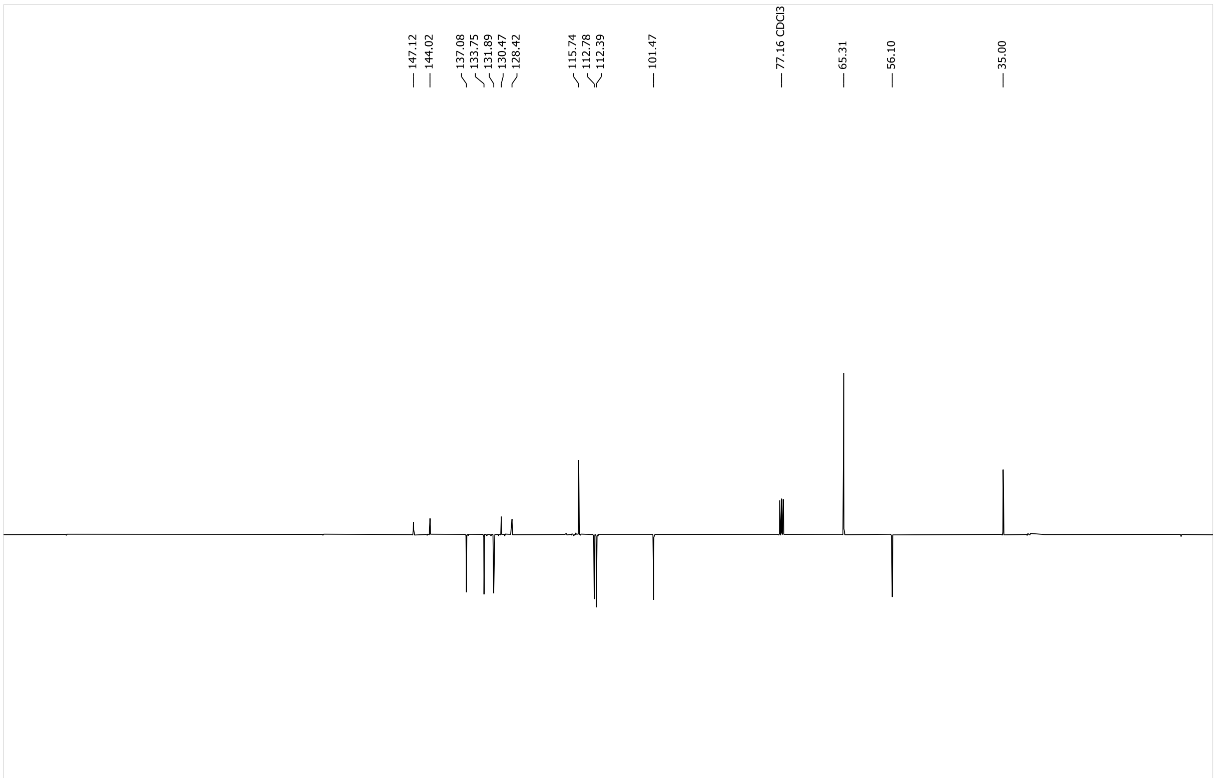

(*E*)-2-fluoro-4-(penta-2,4-dien-1-yl)phenol (11k)

^{1}H NMR (400 MHz, $CDCl_3$)

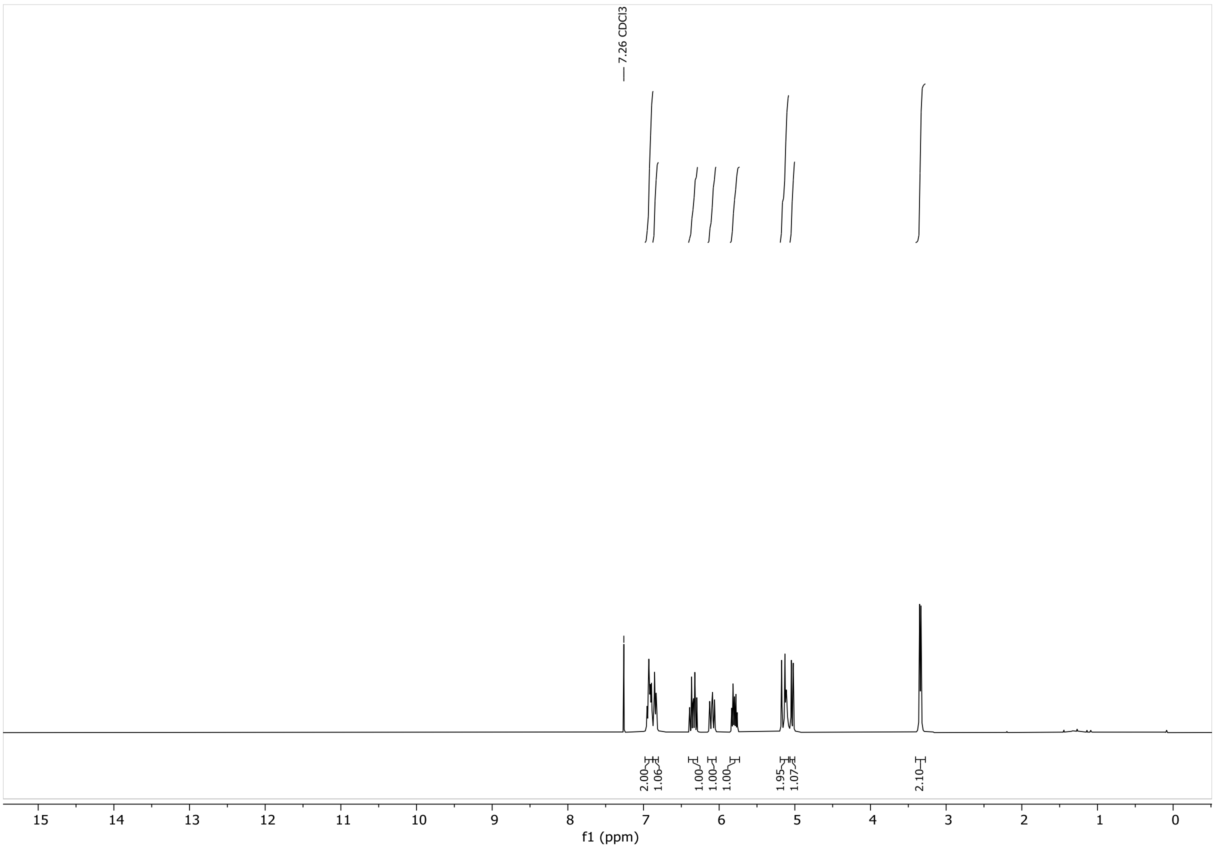

^{13}C-NMR (101 MHz, $CDCl_3$)

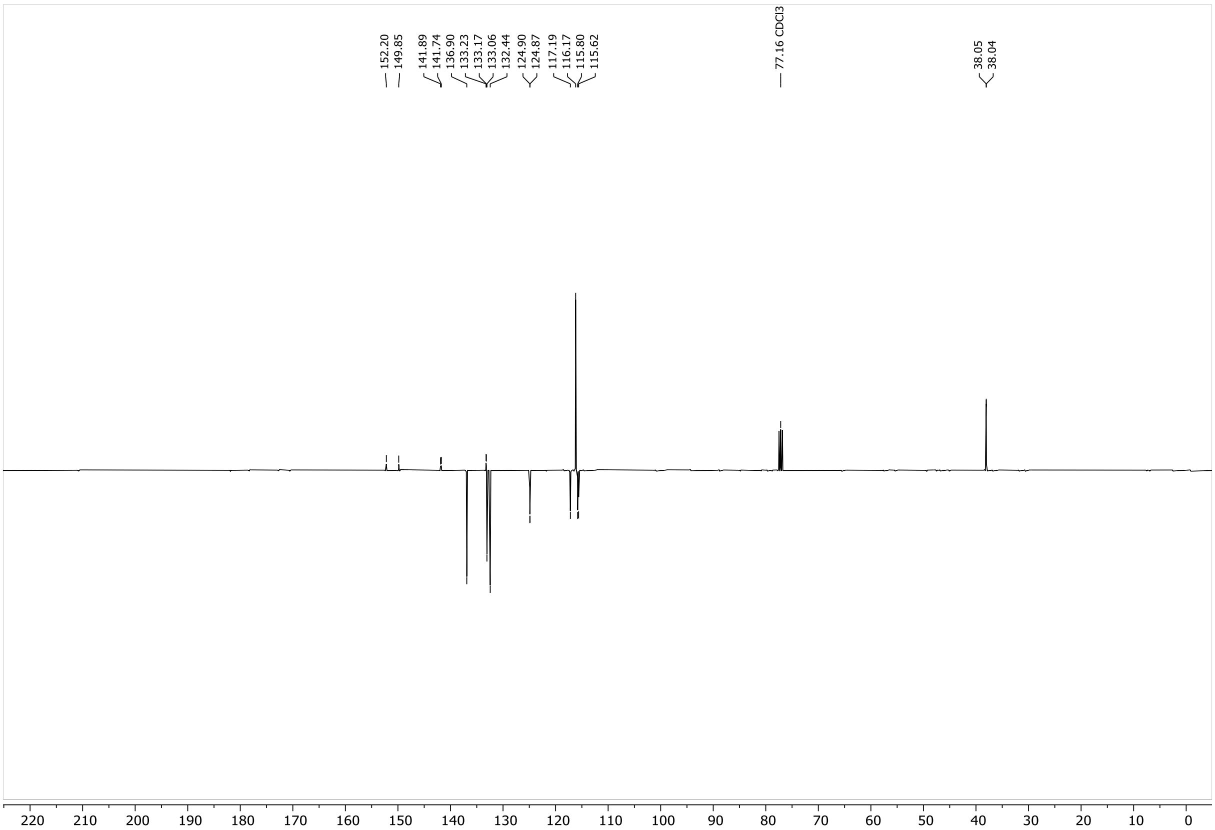

^{19}F-NMR (376 MHz, $CDCl_3$)

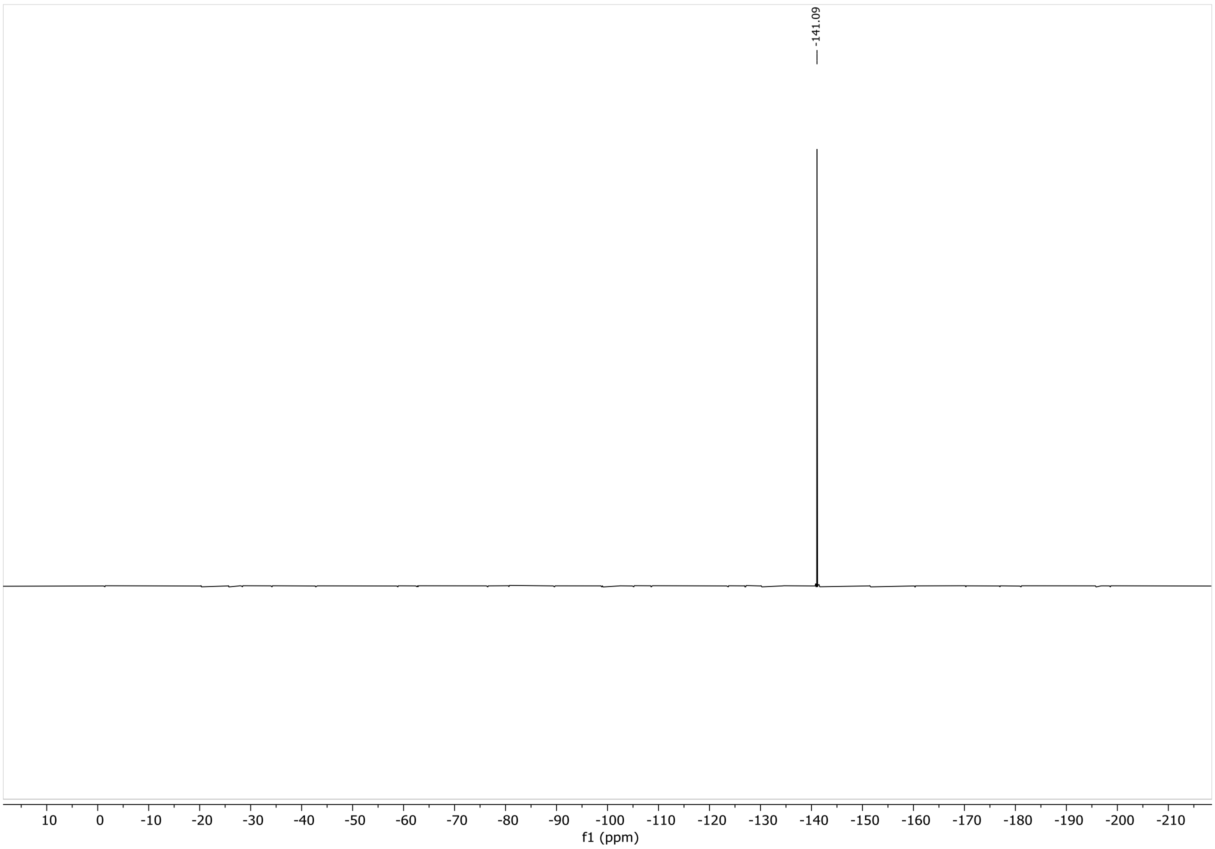

(*E*)-2-bromo-4-(penta-2,4-dien-1-yl)phenol (11l)

^{1}H NMR (400 MHz, $CDCl_3$)

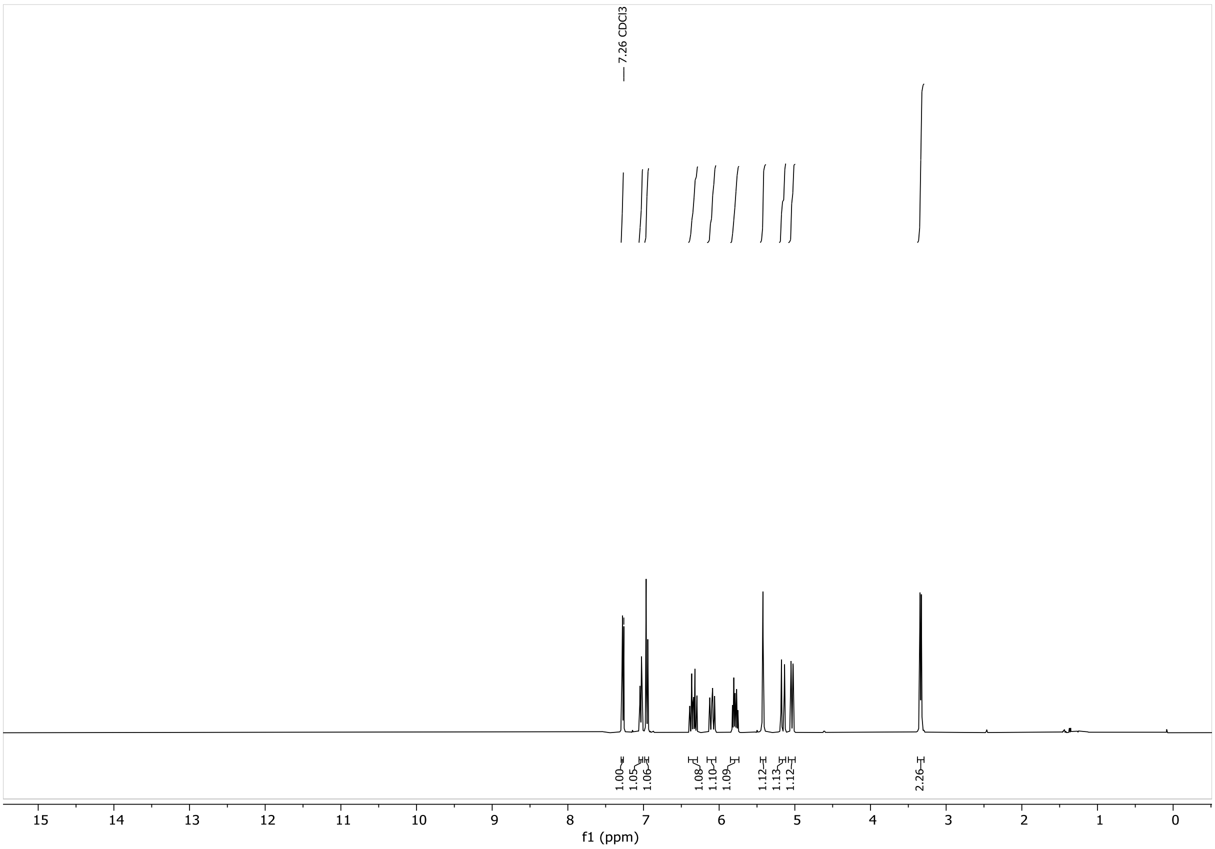

^{13}C-NMR (101 MHz, $CDCl_3$)

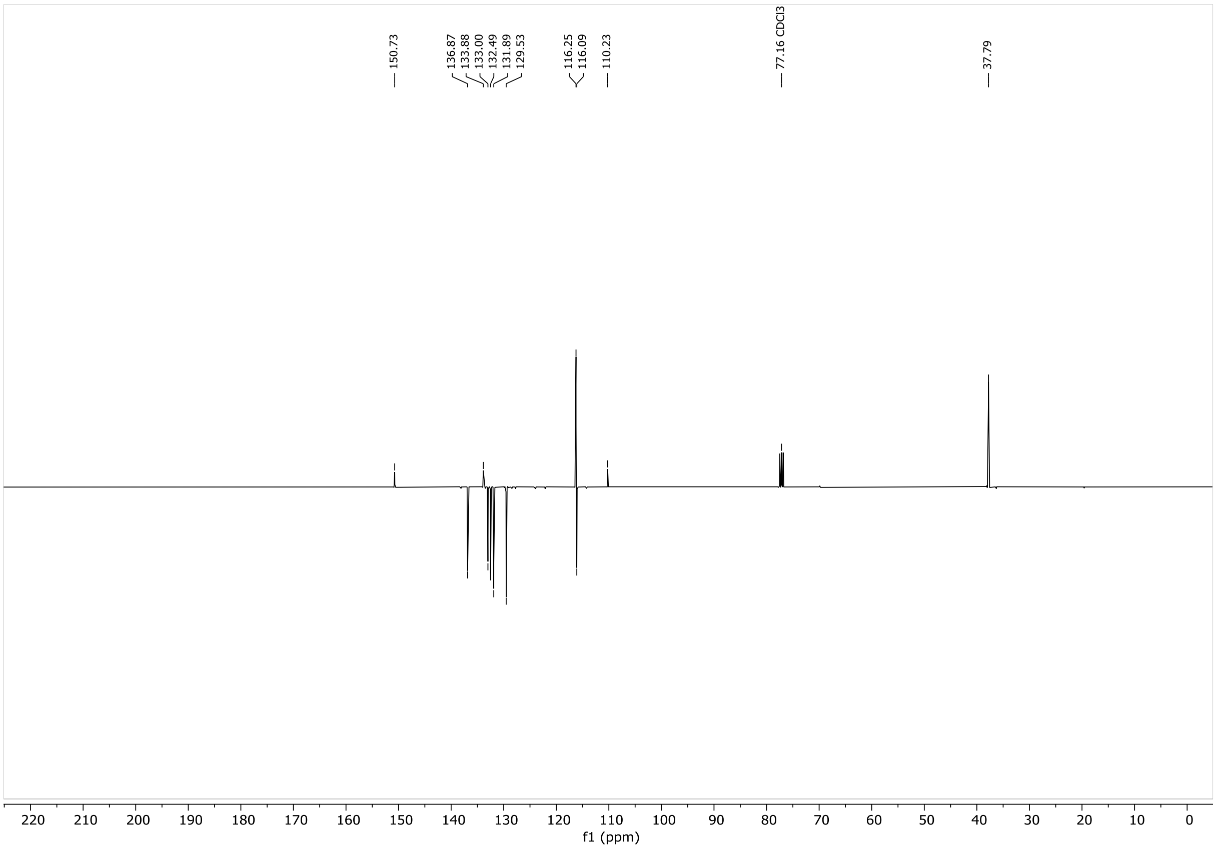

(*E*)-5-hydroxy-4-methoxy-2-(penta-2,4-dien-1-yl)benzaldehyde (11m)

^{1}H NMR (400 MHz, $CDCl_3$)

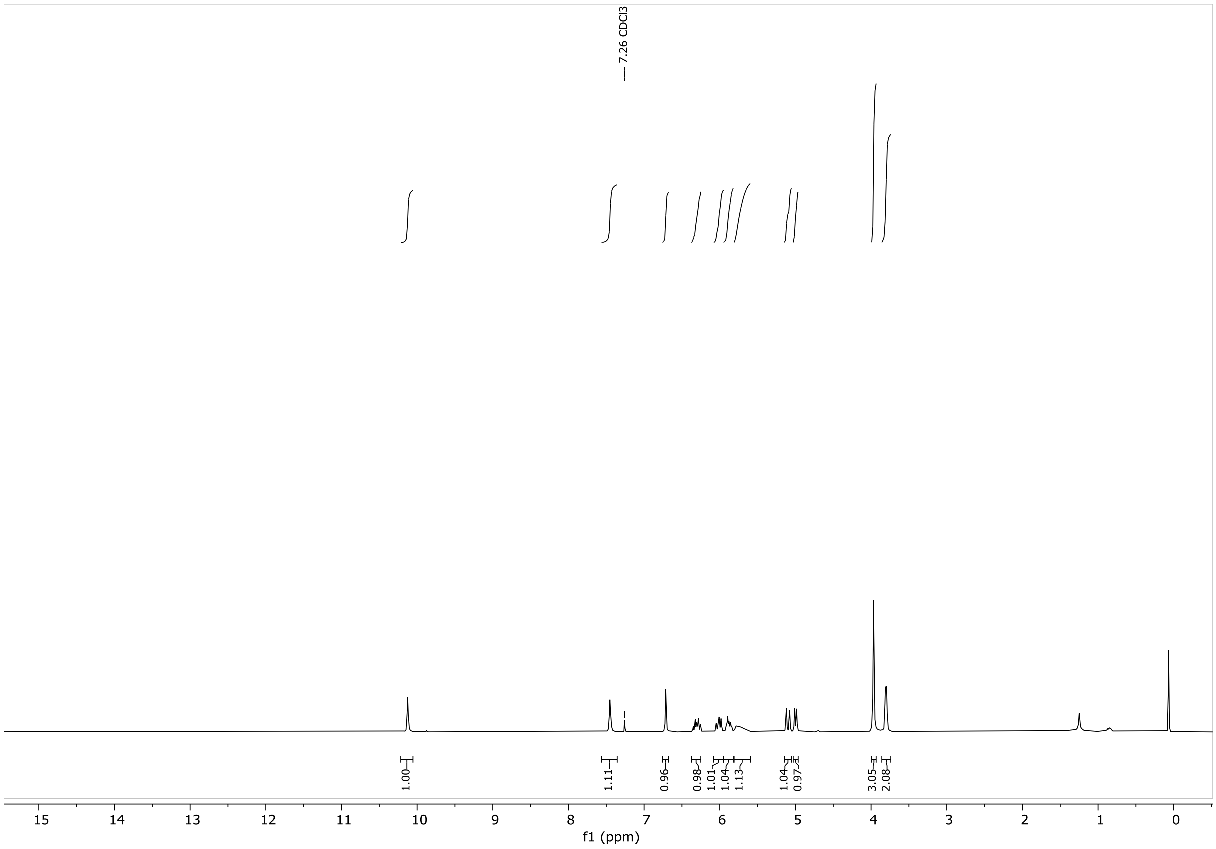

^{13}C-NMR (101 MHz, $CDCl_3$)

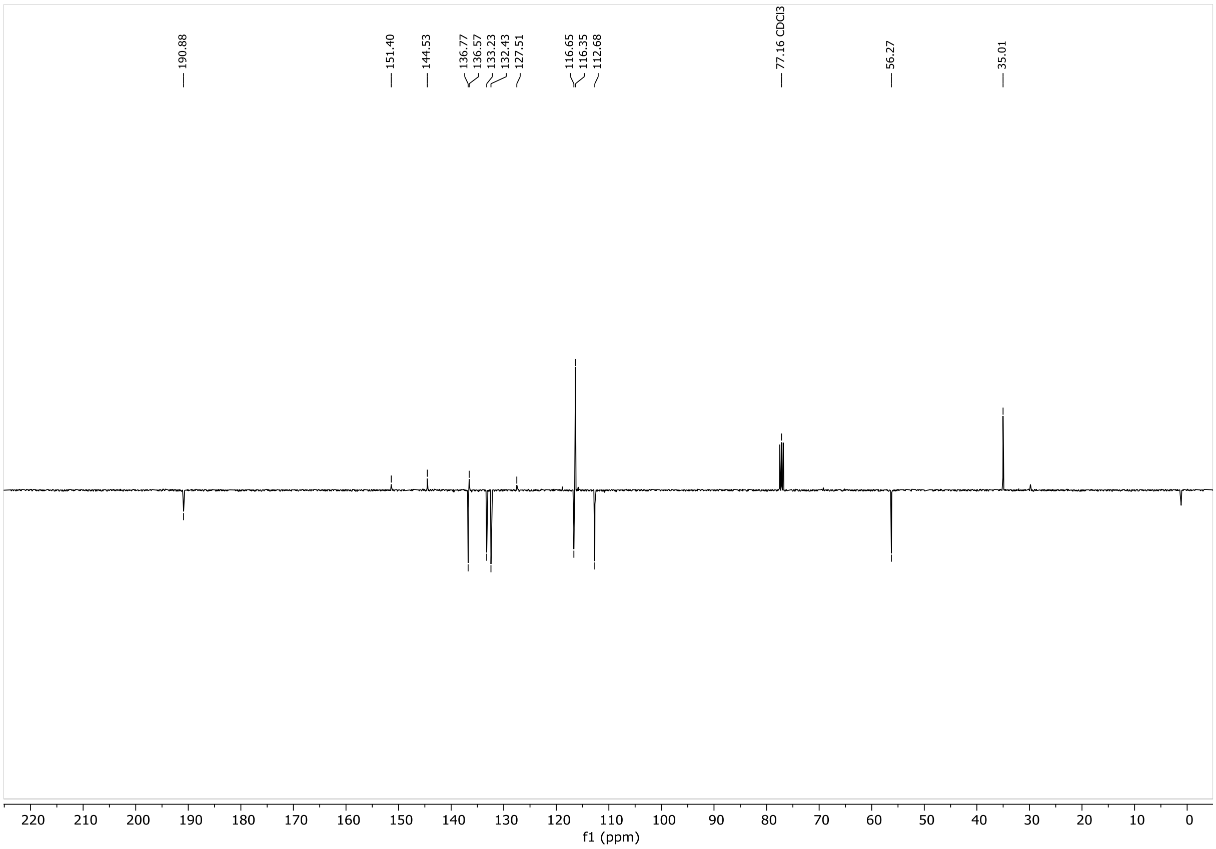

(*E*)-(2-hydroxy-5-(penta-2,4-dien-1-yl)phenyl)(phenyl)methanone (11n)

^{1}H NMR (400 MHz, $CDCl_3$)

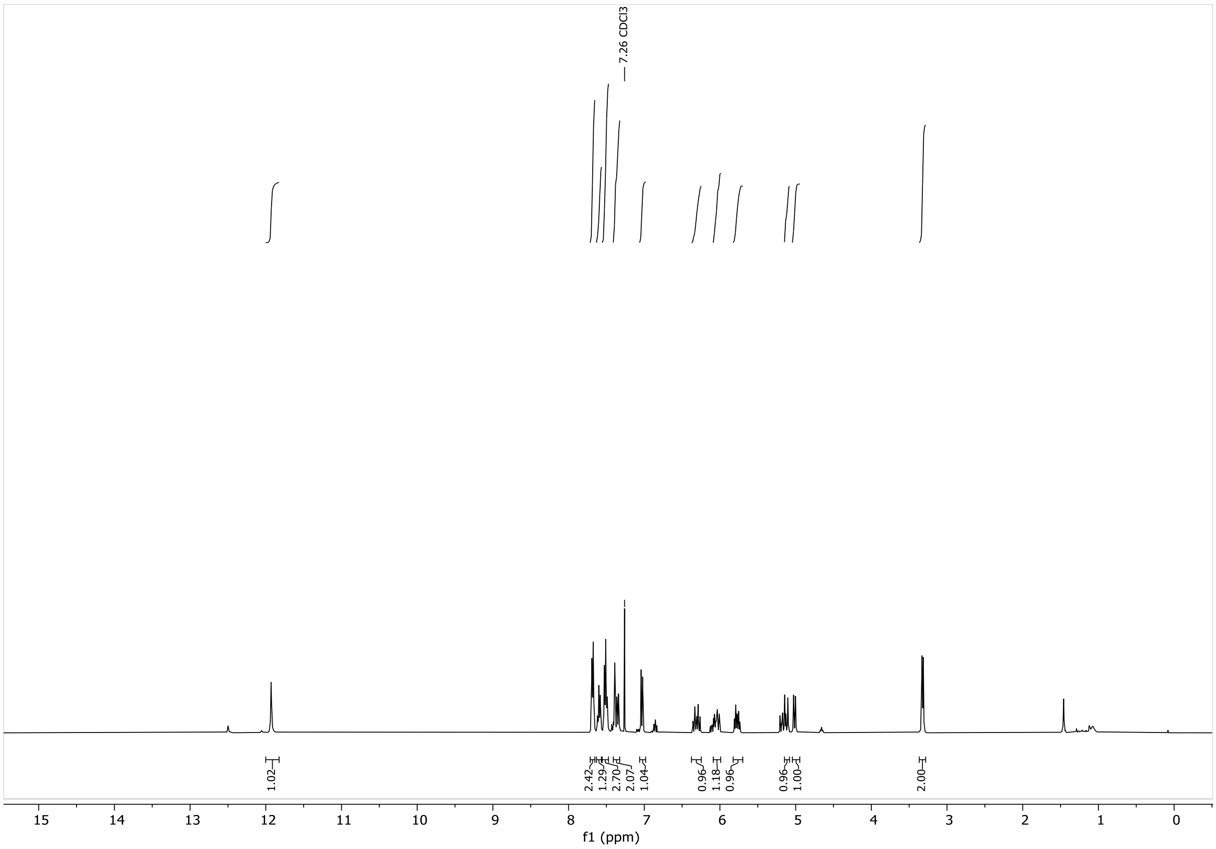

^{13}C-NMR (101 MHz, $CDCl_3$)

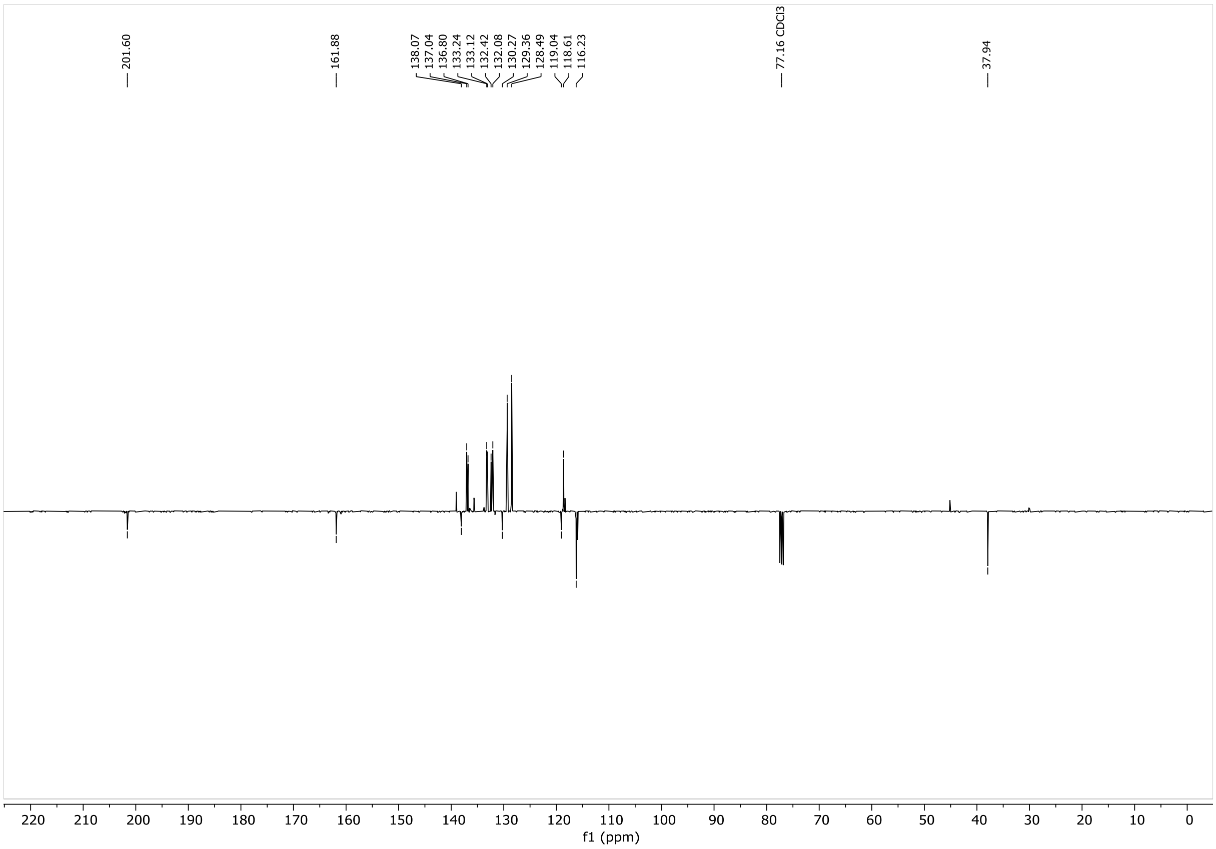

(*E*)-4-(penta-2,4-dien-1-yl)benzene-1,2-diol (11o)

^{1}H NMR (400 MHz, $CDCl_3$)

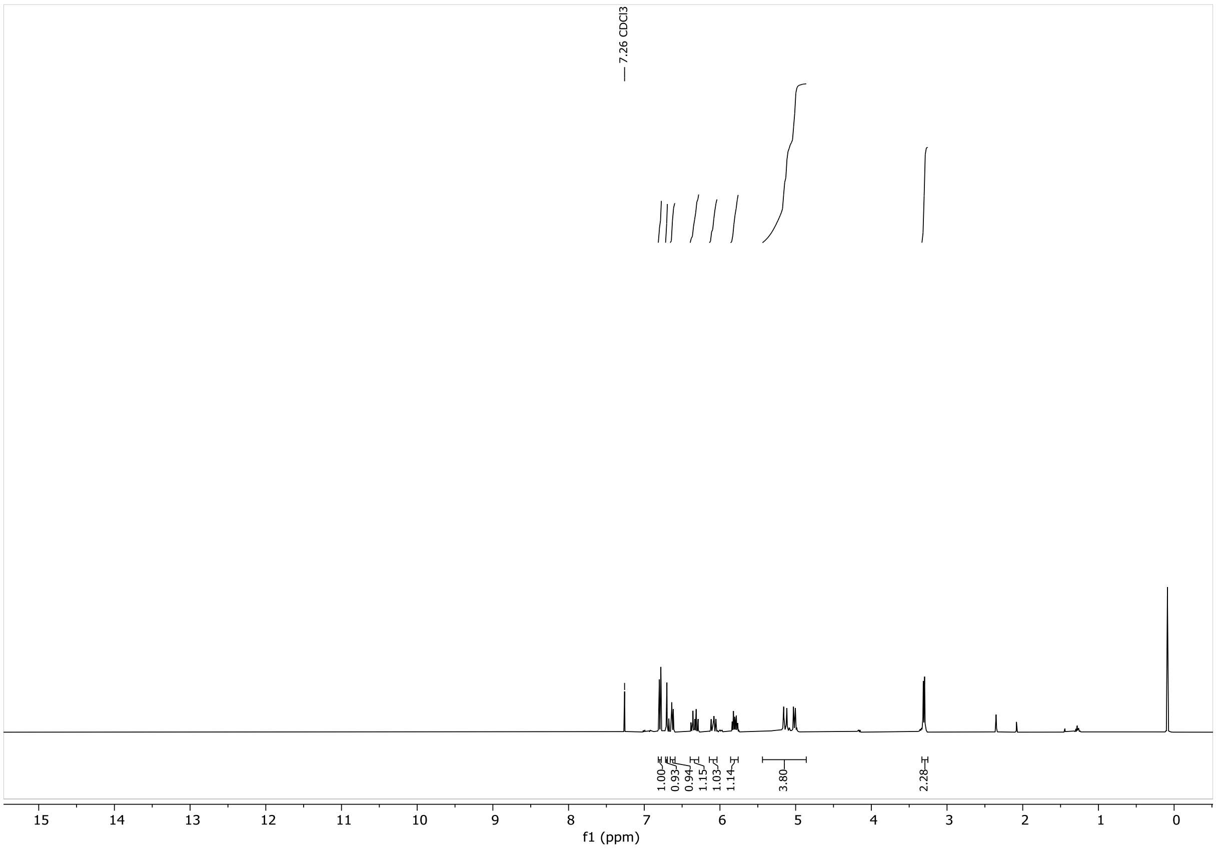

^{13}C-NMR (101 MHz, $CDCl_3$)

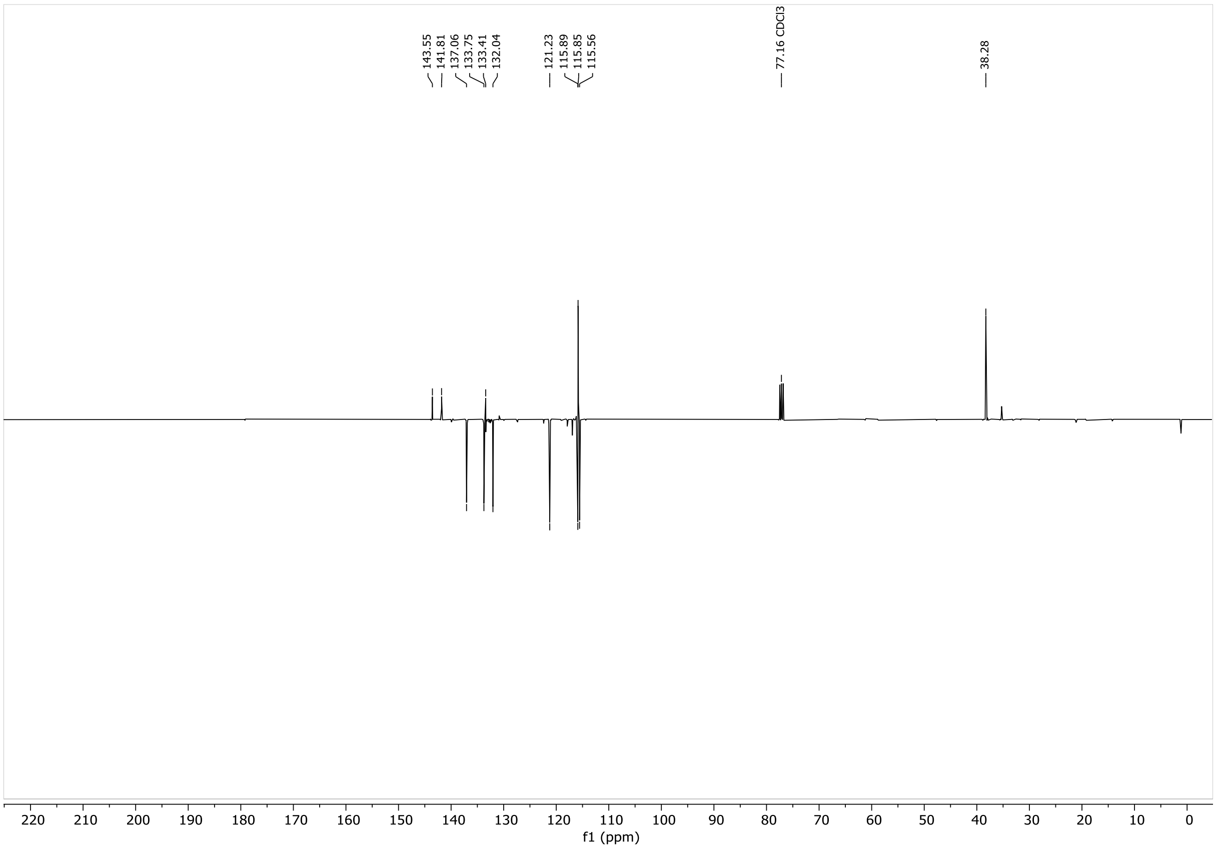

(*E*)-2-nitro-4-(penta-2,4-dien-1-yl)phenol (11p)

^{1}H NMR (400 MHz, $CDCl_3$)

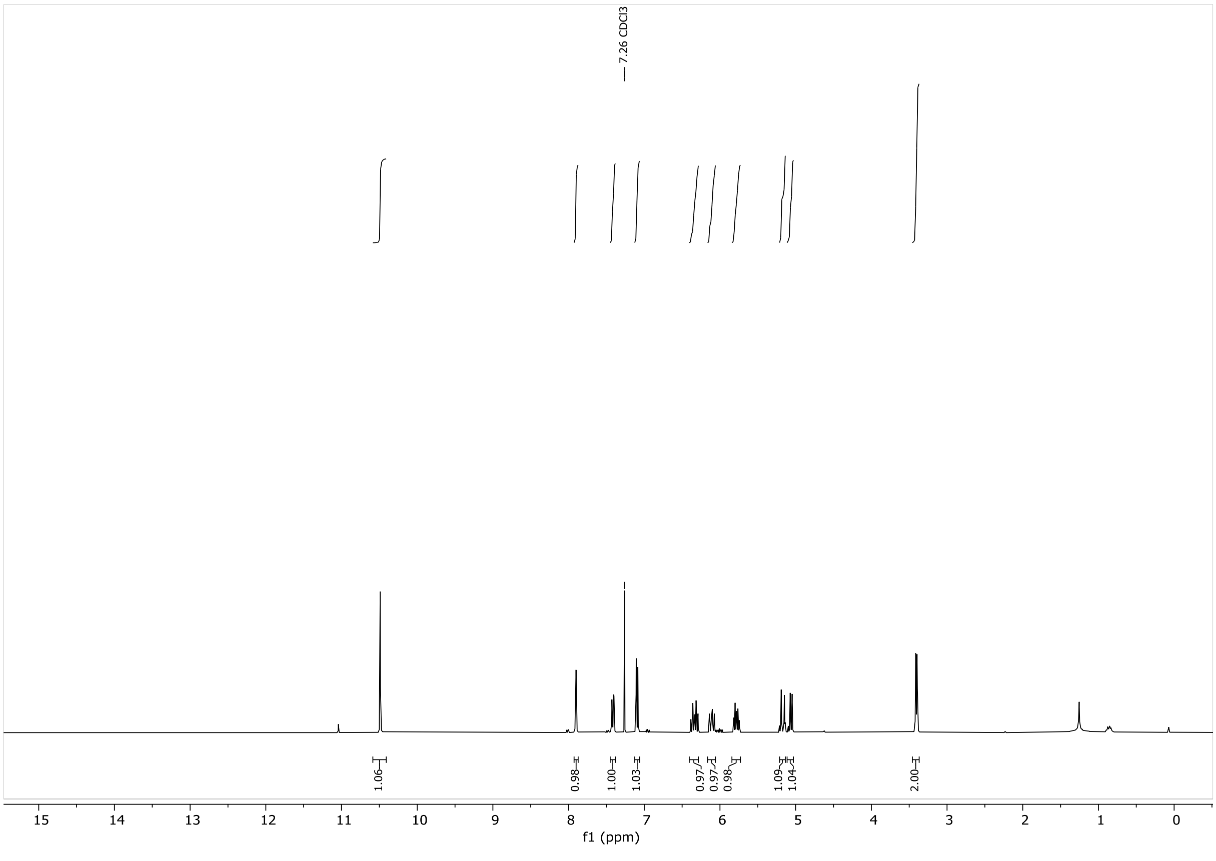

^{13}C-NMR (101 MHz, $CDCl_3$)

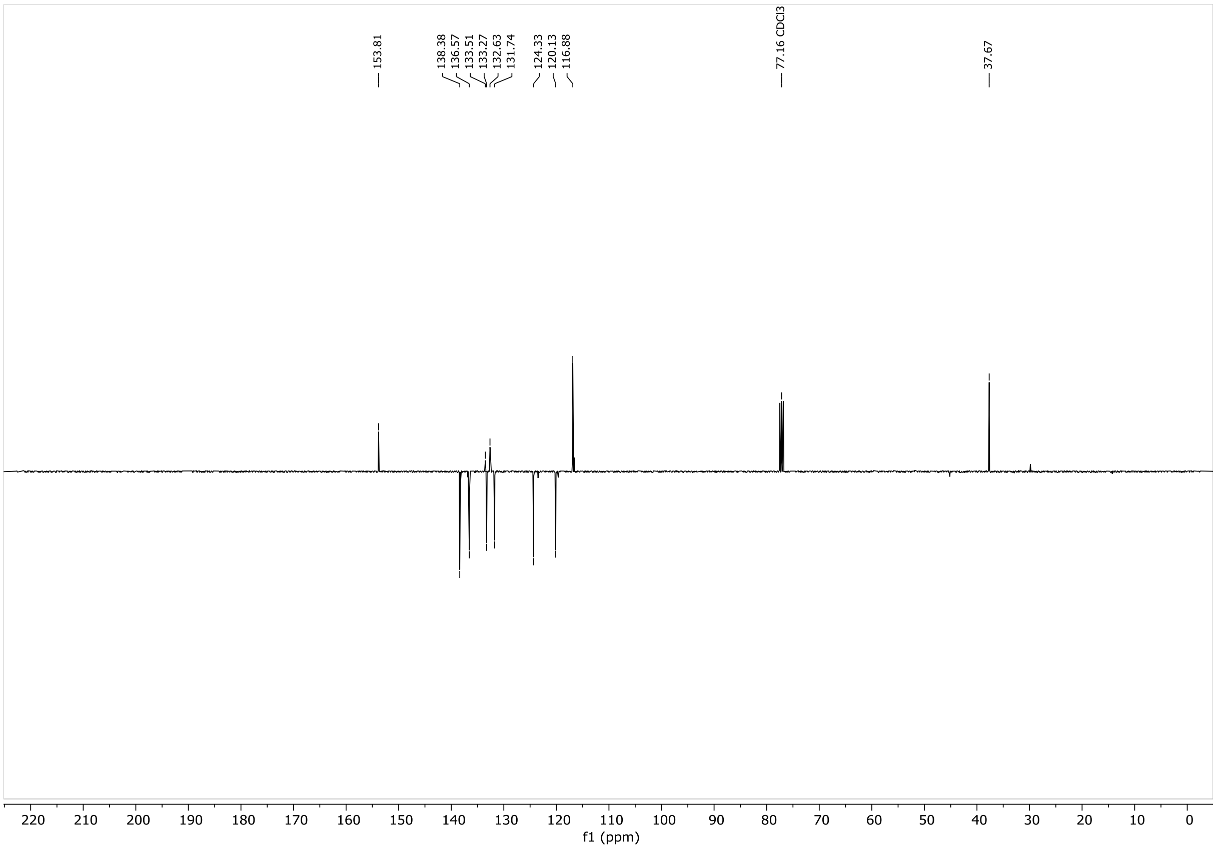

(*E*)-3-nitro-4-(penta-2,4-dien-1-yl)phenol (11q)

^{1}H NMR (400 MHz, $CDCl_3$)

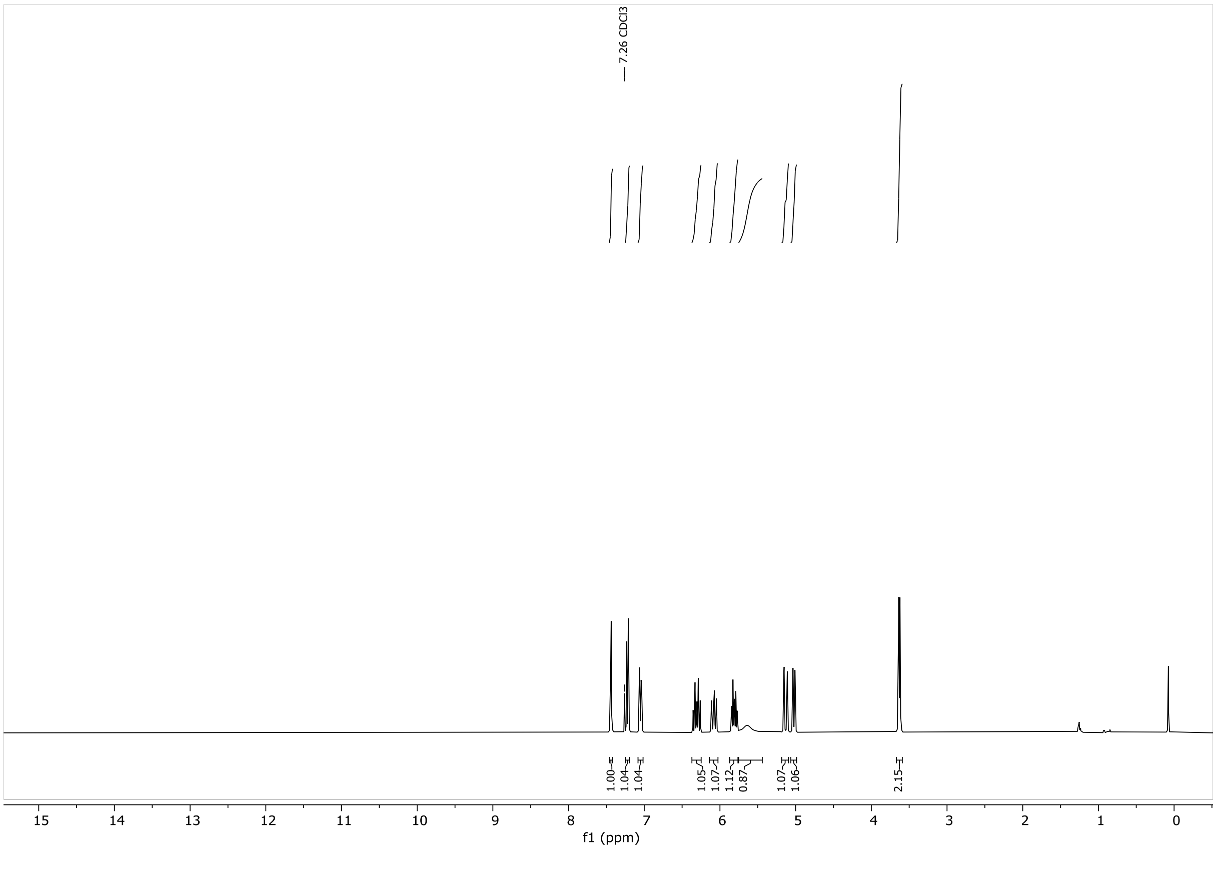

^{13}C-NMR (101 MHz, $CDCl_3$)

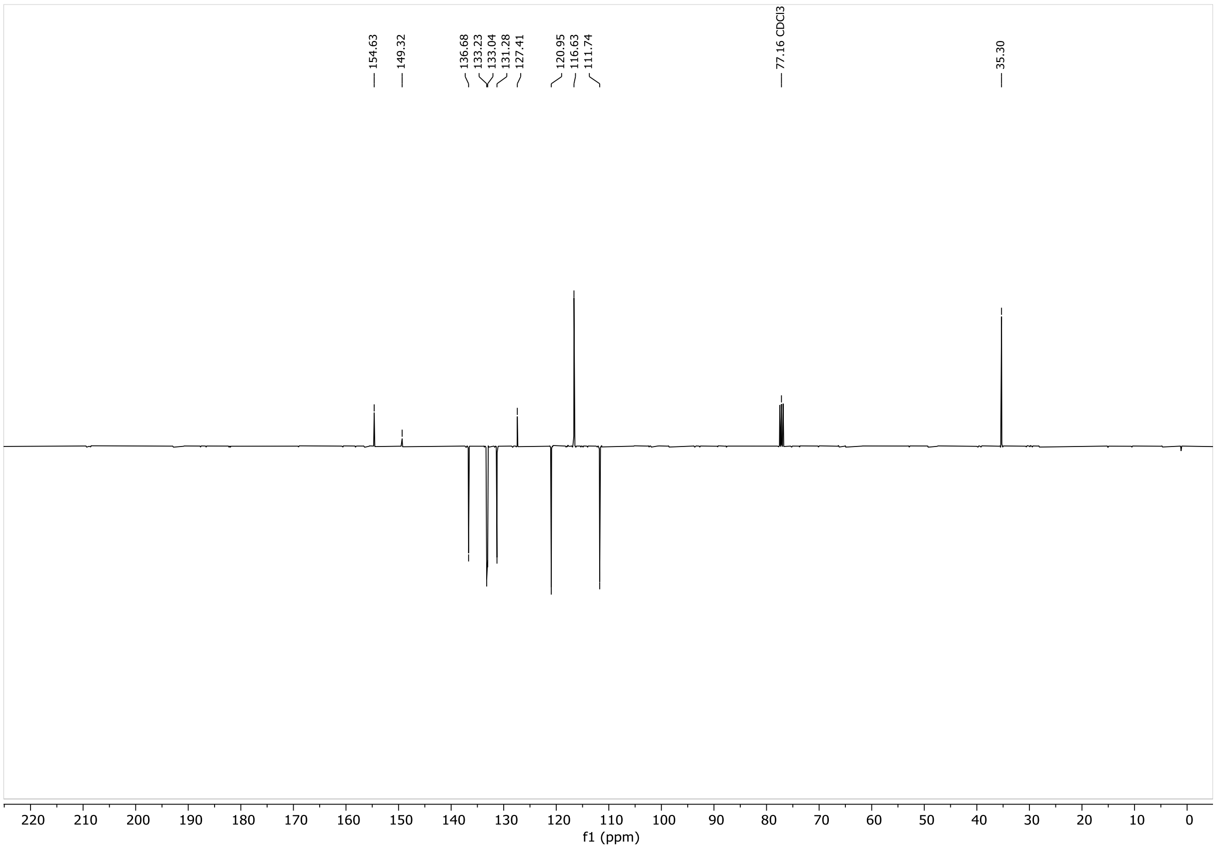

(*E*)-penta-2,4-dien-1,1-d2-1-ol (S1)

^{1}H NMR (600 MHz, $CDCl_3$)

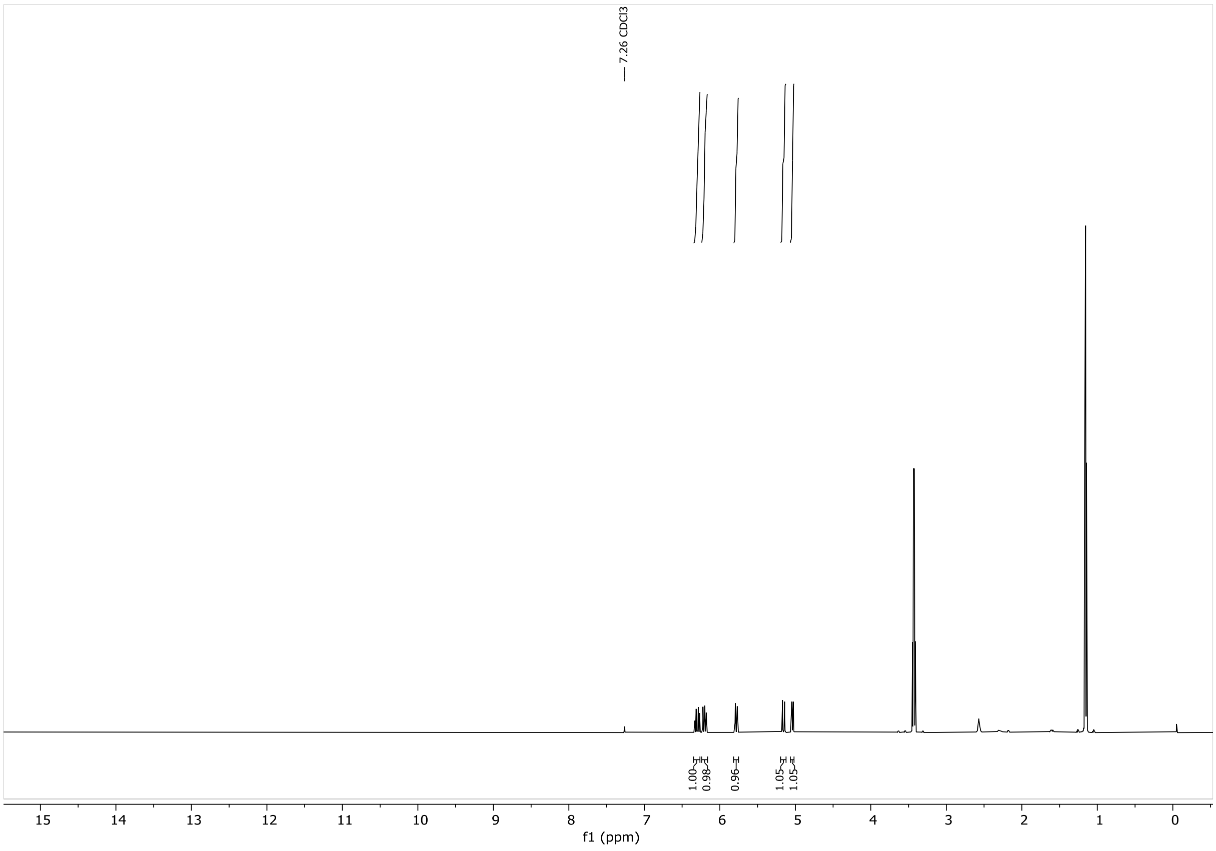

^{13}C-NMR (151 MHz, $CDCl_3$)

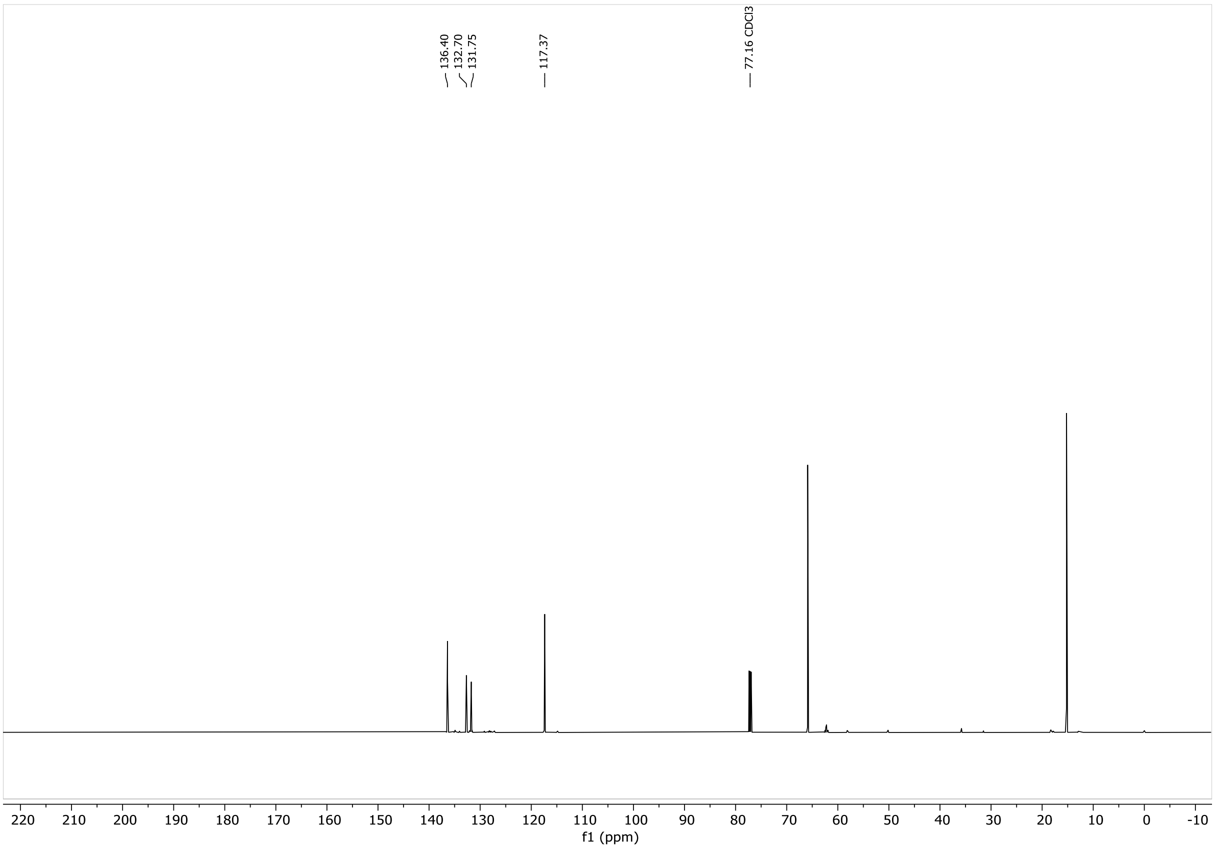

(*E*)-1-(methoxy-d_3)-2-((penta-2,4-dien-1-yl-1,1-d_2)oxy)benzene (10f-d_5)

^{1}H NMR (400 MHz, $CDCl_3$)

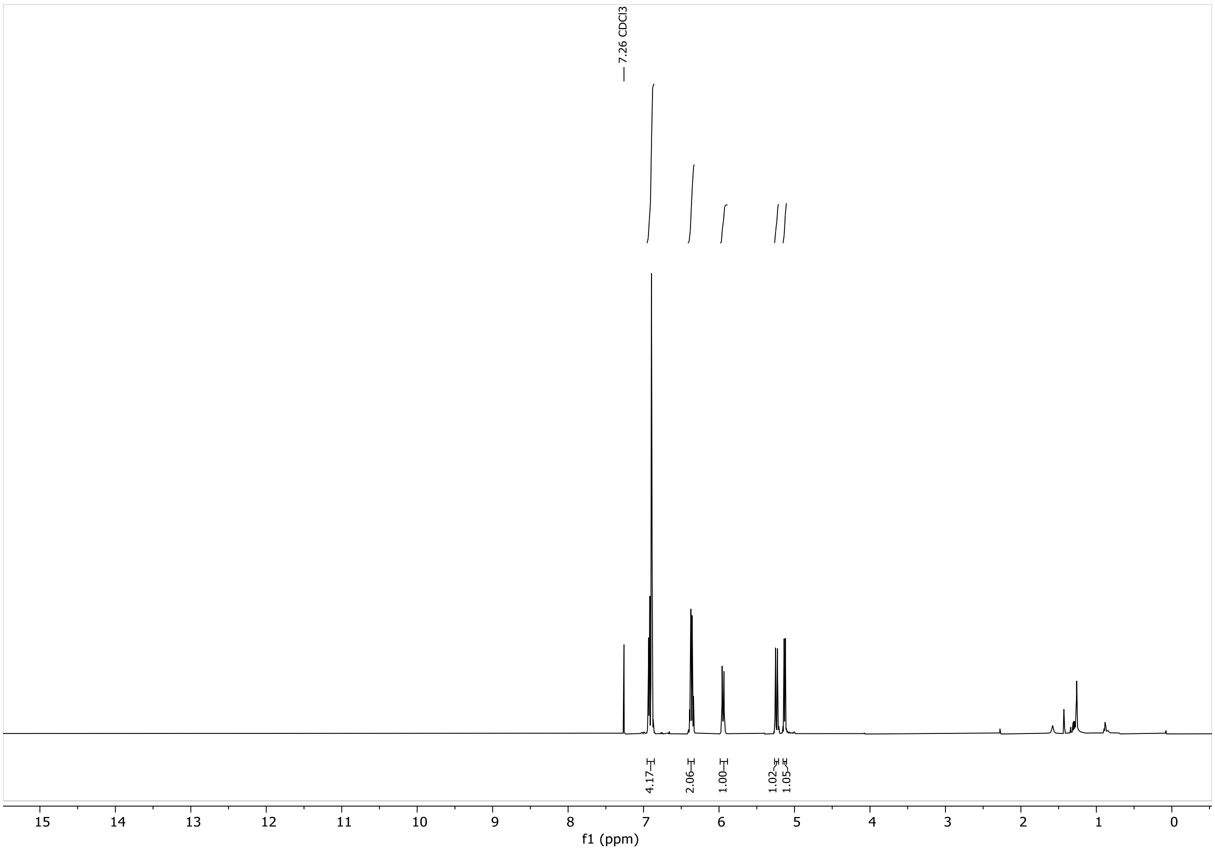

^{13}C-NMR (151 MHz, $CDCl_3$)

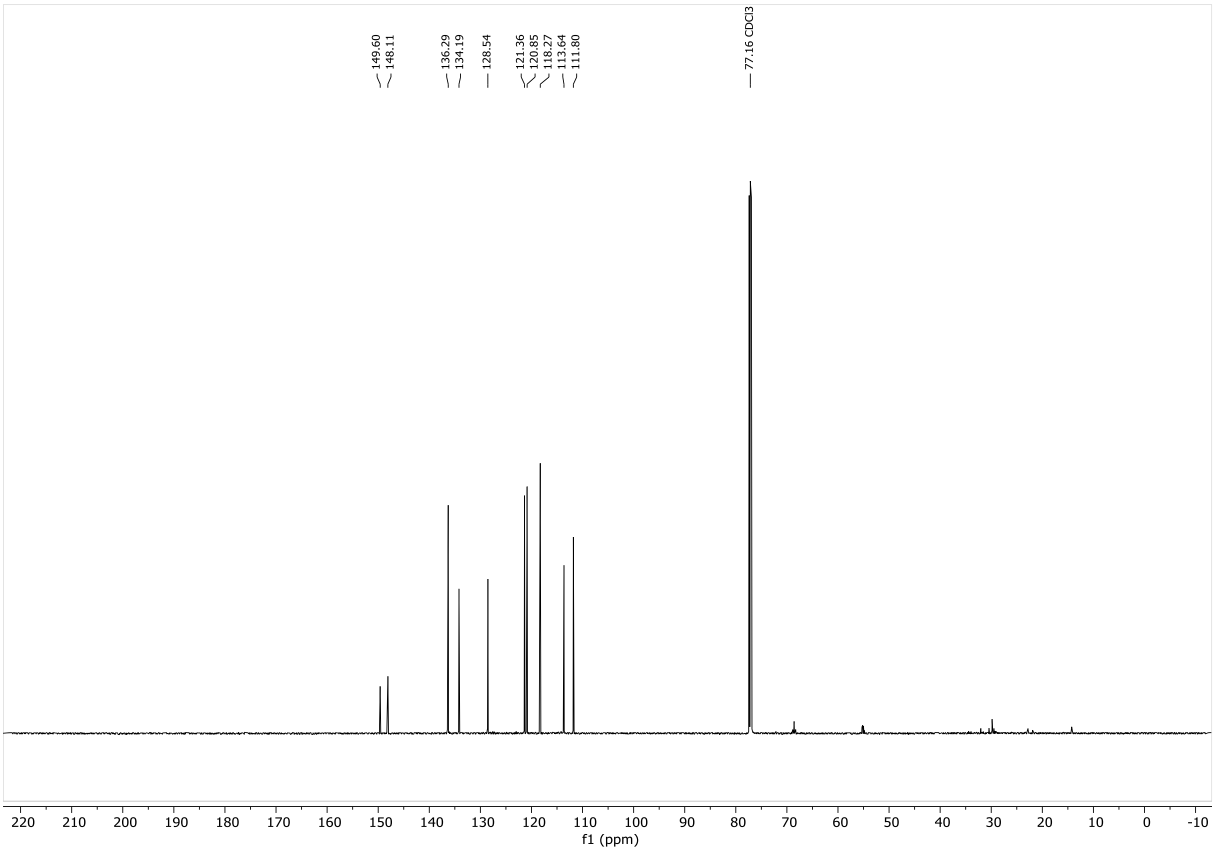

(*E*)-2-(methoxy-d_3)-4-(penta-2,4-dien-1-yl-5,5-d_2)phenol (11f-d_5)

^{1}H NMR (600 MHz, $CDCl_3$)

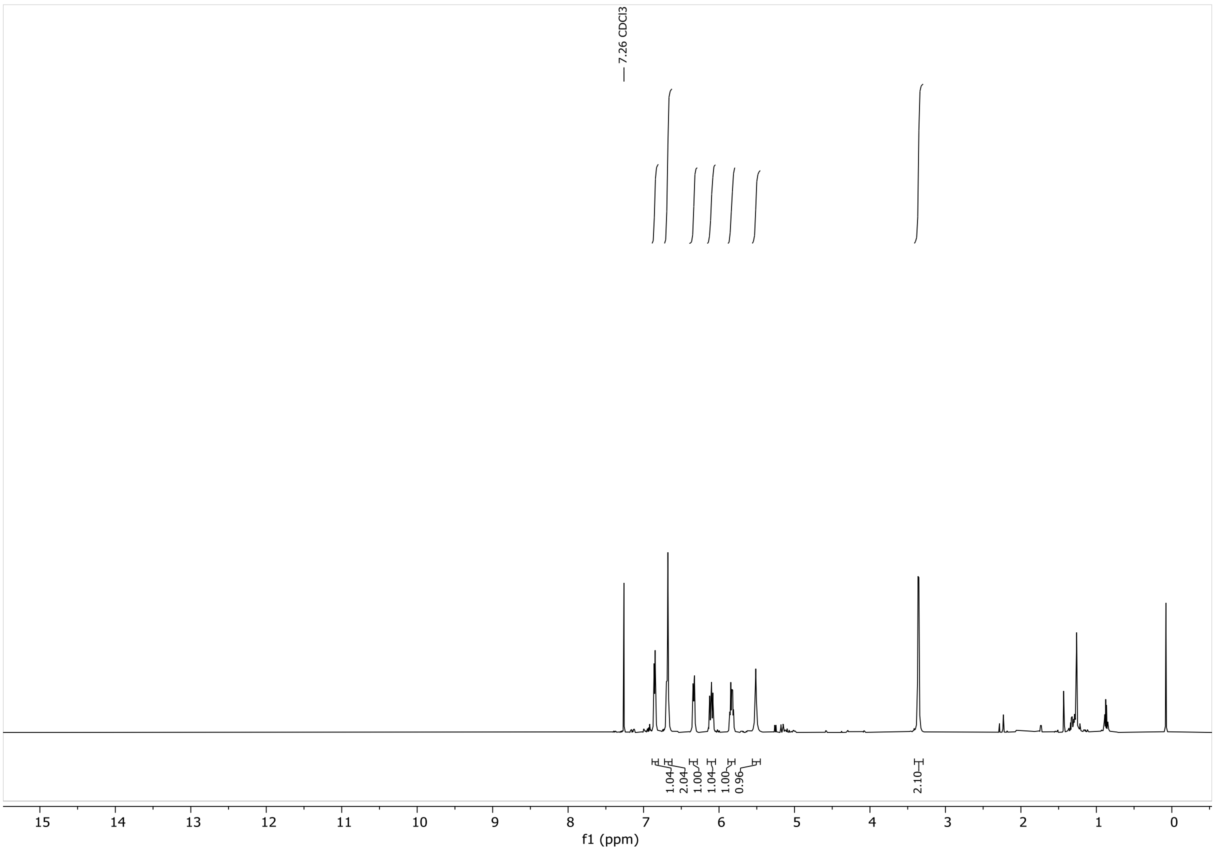

^{13}C-NMR (151 MHz, $CDCl_3$)

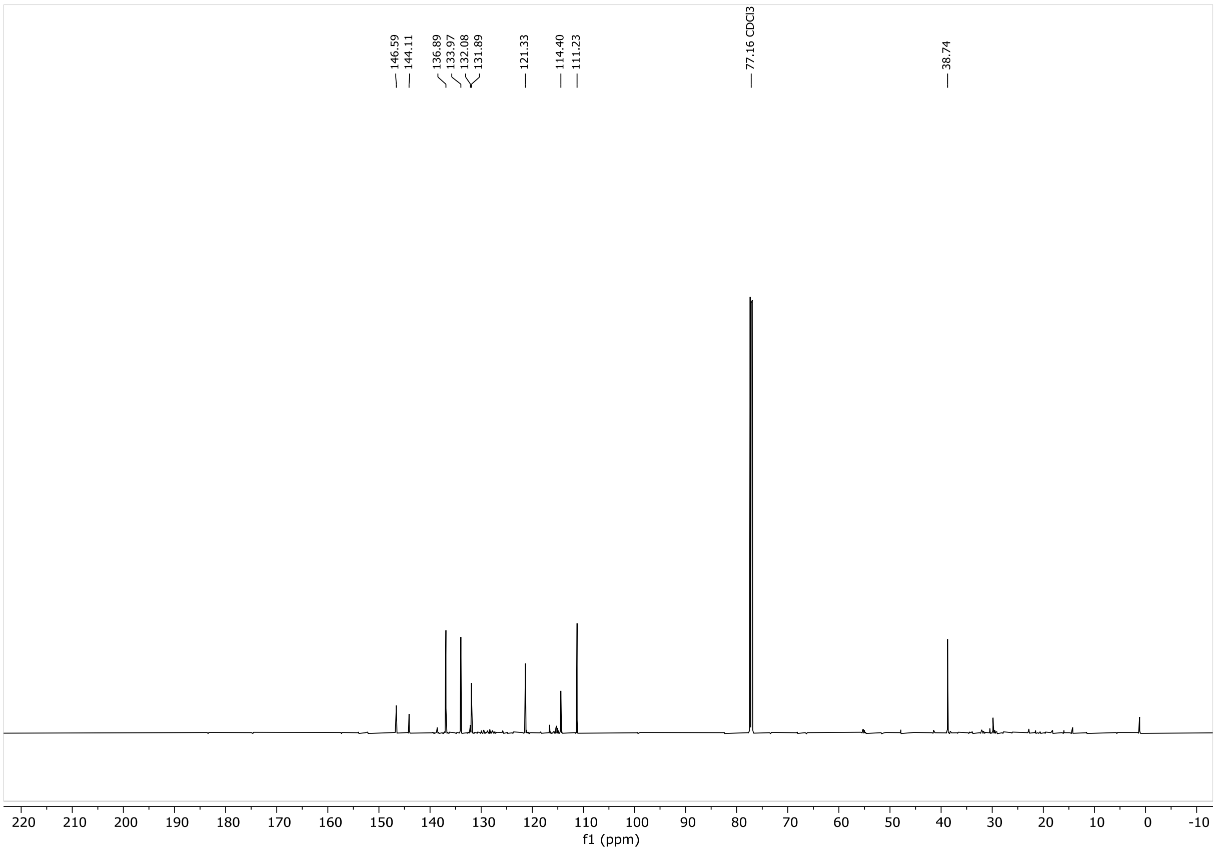

(*E*)-((penta-2,4-dien-1-yl-1,1-d2)oxy)benzene (12)

^{1}H NMR (400 MHz, $CDCl_3$)

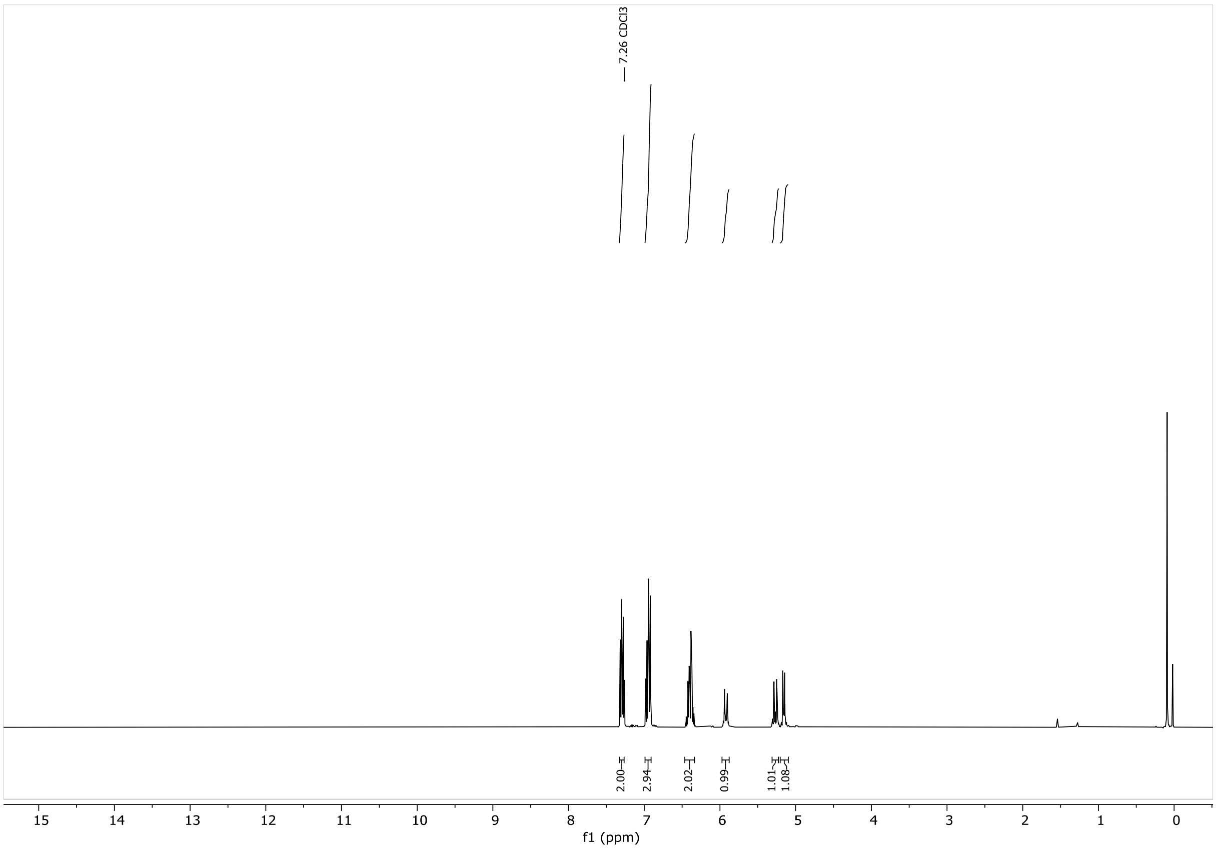

^{13}C-NMR (101 MHz, $CDCl_3$)

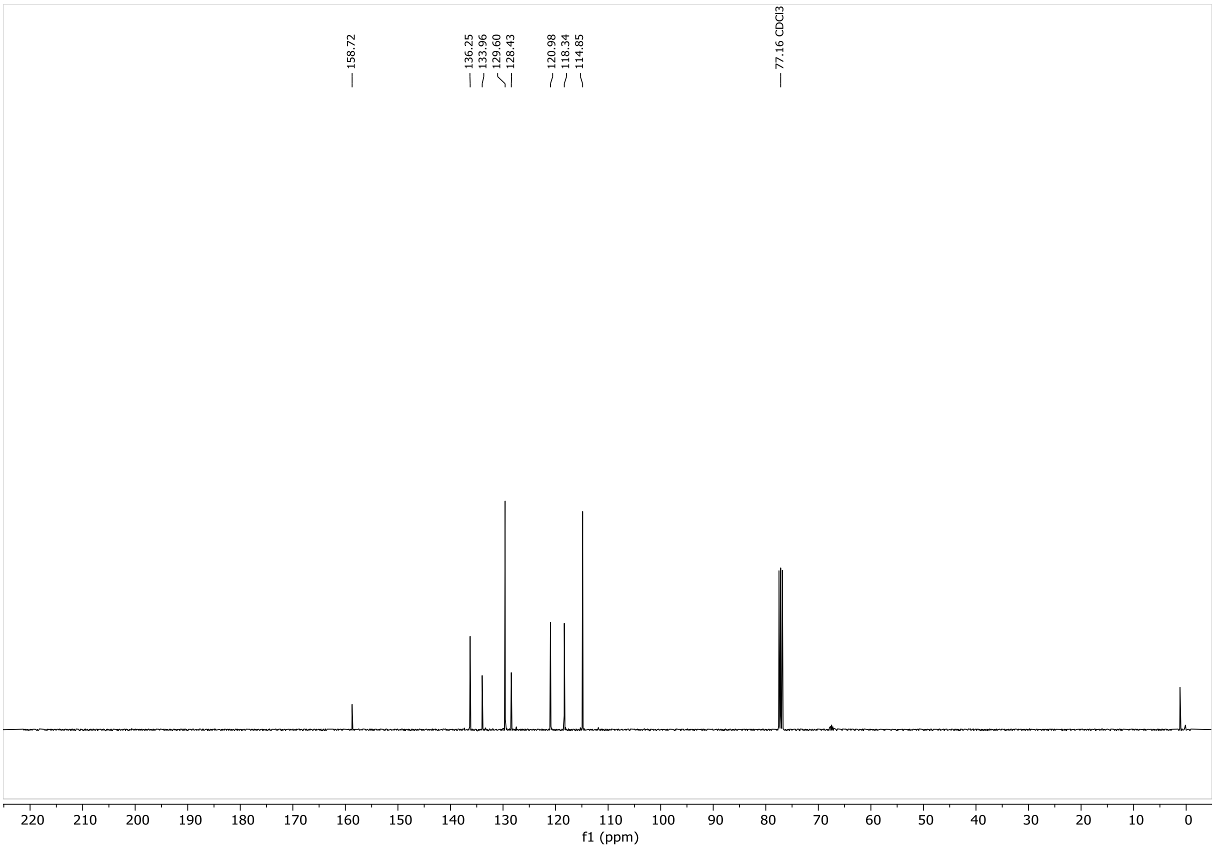

(*E*)-4-(penta-2,4-dien-1-yl-5,5-d2)phenol (13)

^{1}H NMR (600 MHz, $CDCl_3$)

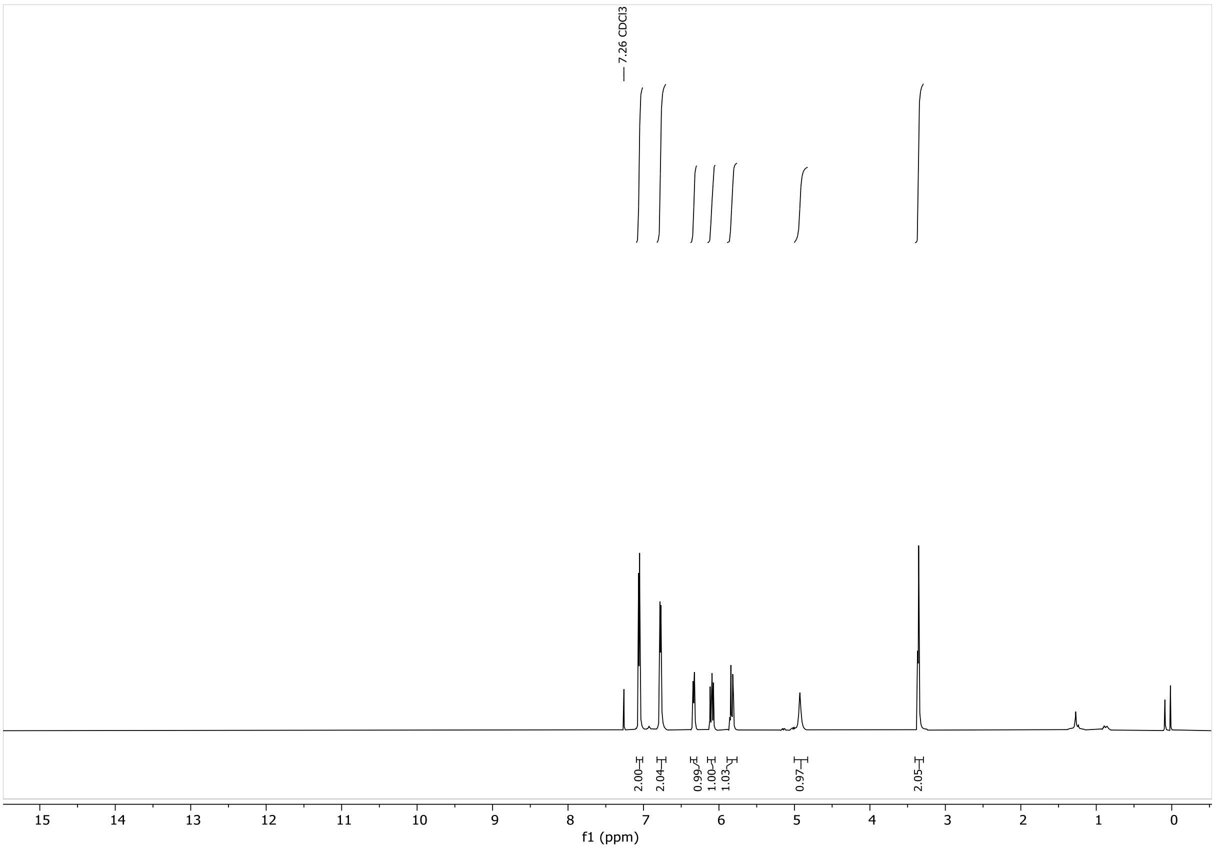

^{13}C-NMR (151 MHz, $CDCl_3$)

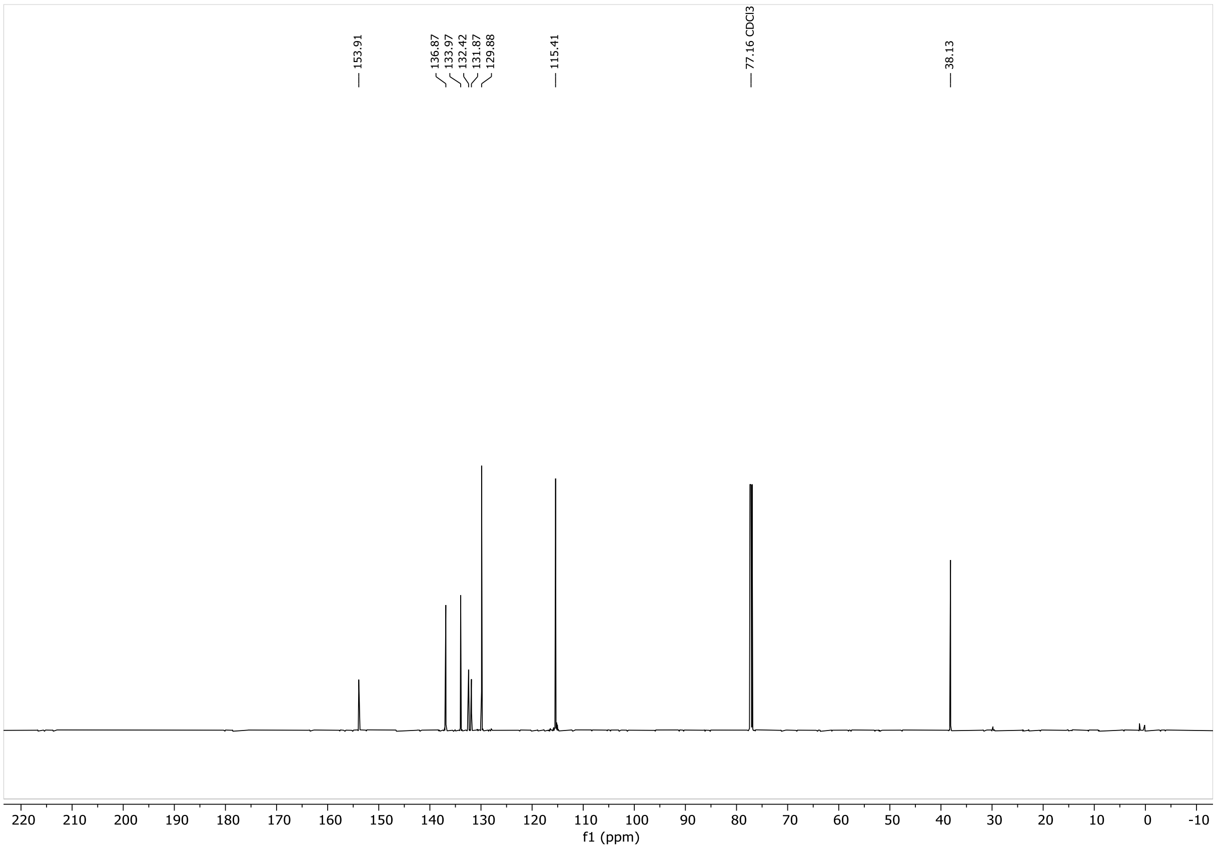

(*E*)-1,3-Dimethyl-2-((penta-2,4-dien-1-yl-1,1-d2)oxy)benzene (14)

^{1}H NMR (400 MHz, $CDCl_3$)

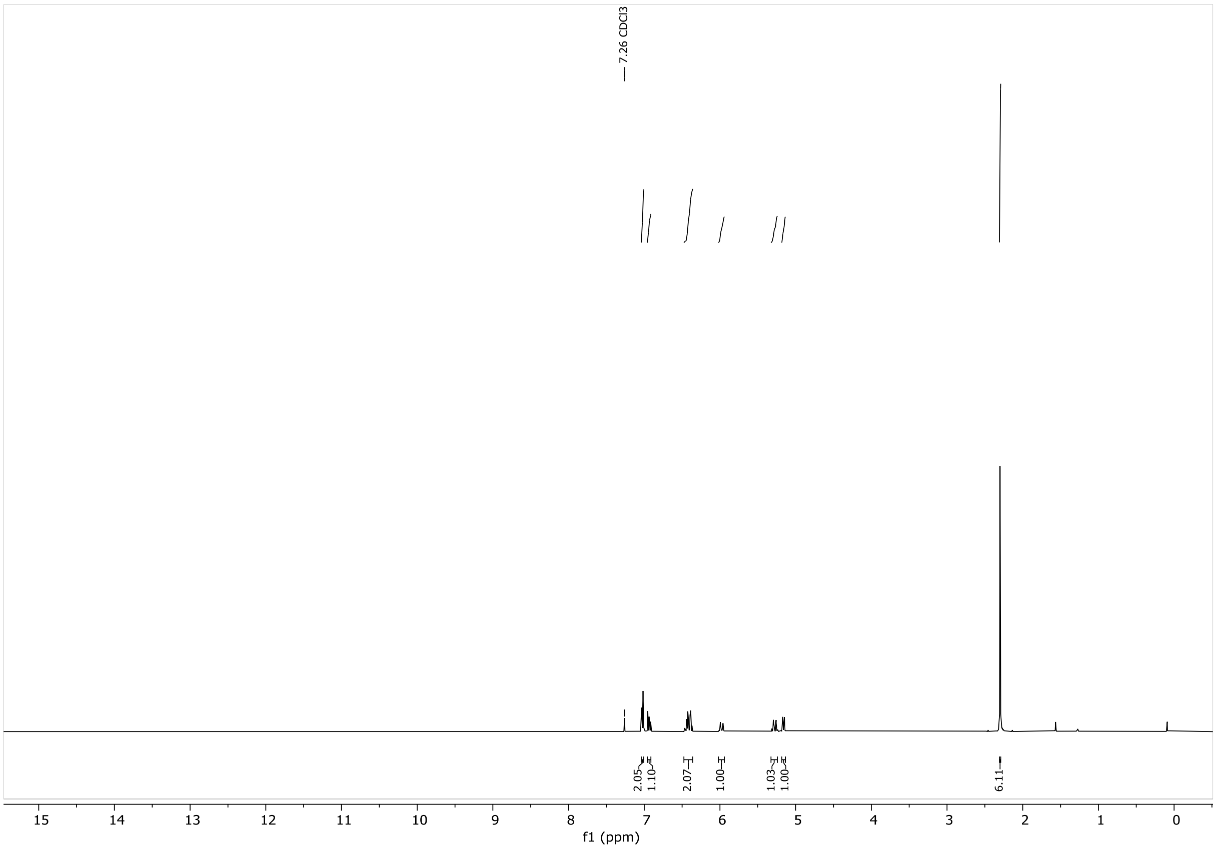

^{13}C-NMR (101 MHz, $CDCl_3$)

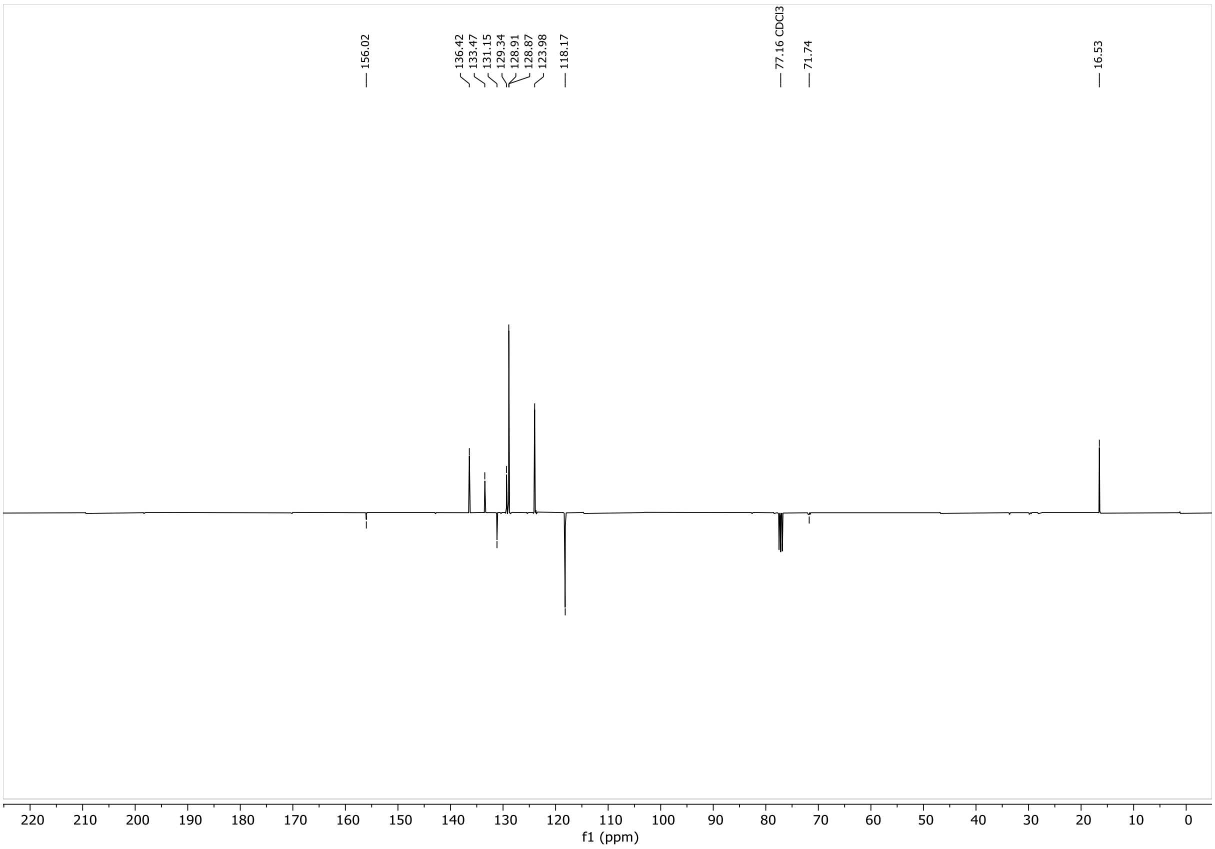

(*E*)-2,6-Dimethyl-4-(penta-2,4-dien-1-yl-5,5-d2)phenol (15)

^{1}H NMR (600 MHz, $CDCl_3$)

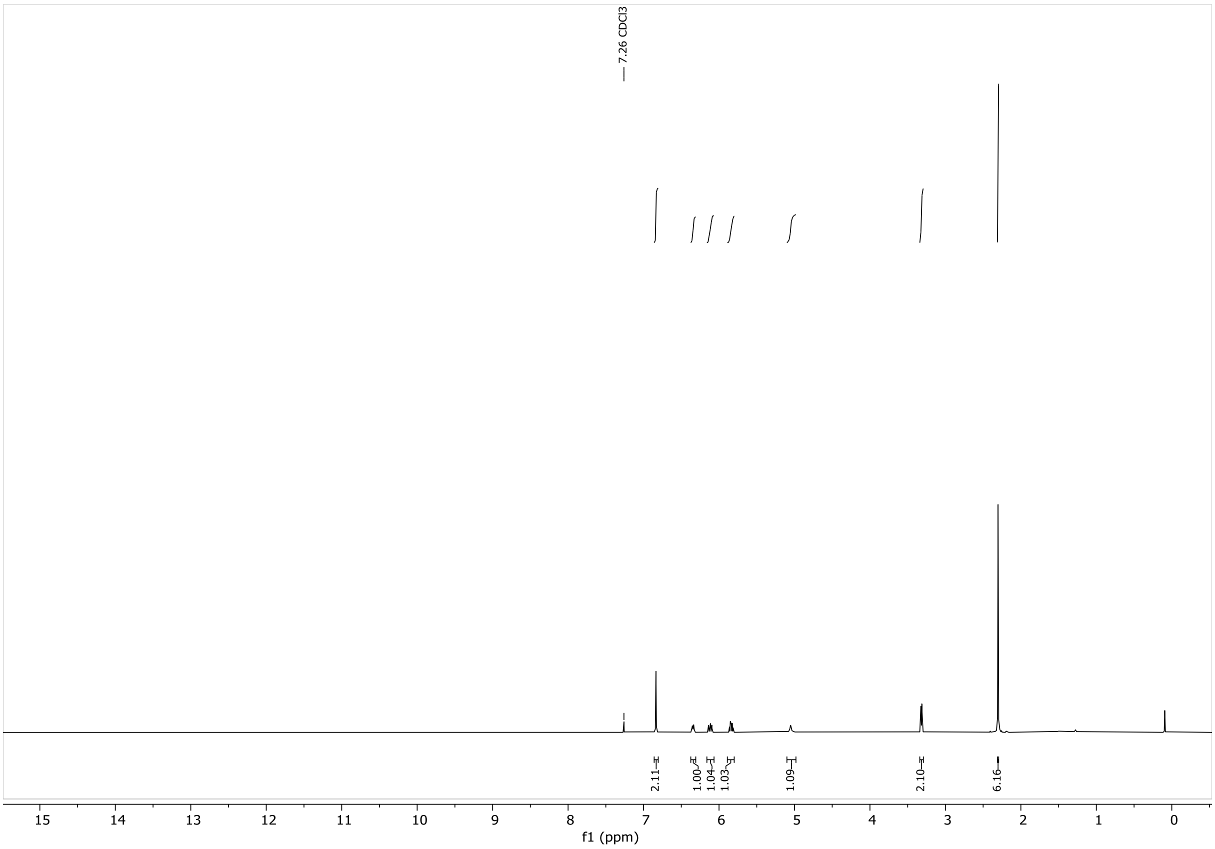

^{13}C-NMR (151 MHz, $CDCl_3$)

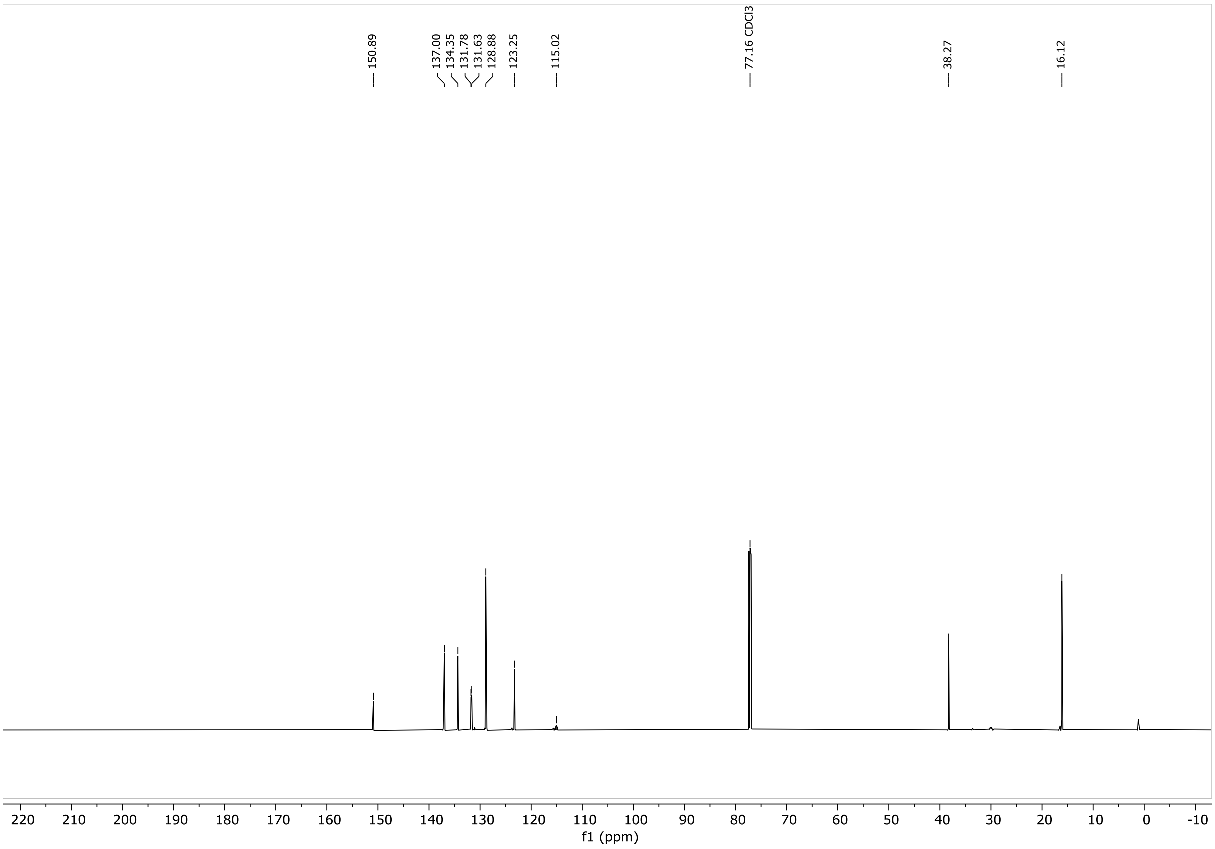

Tert-butyl (*E*)-4-phenoxybut-2-enoate (S2)

^{1}H NMR (400 MHz, $CDCl_3$)

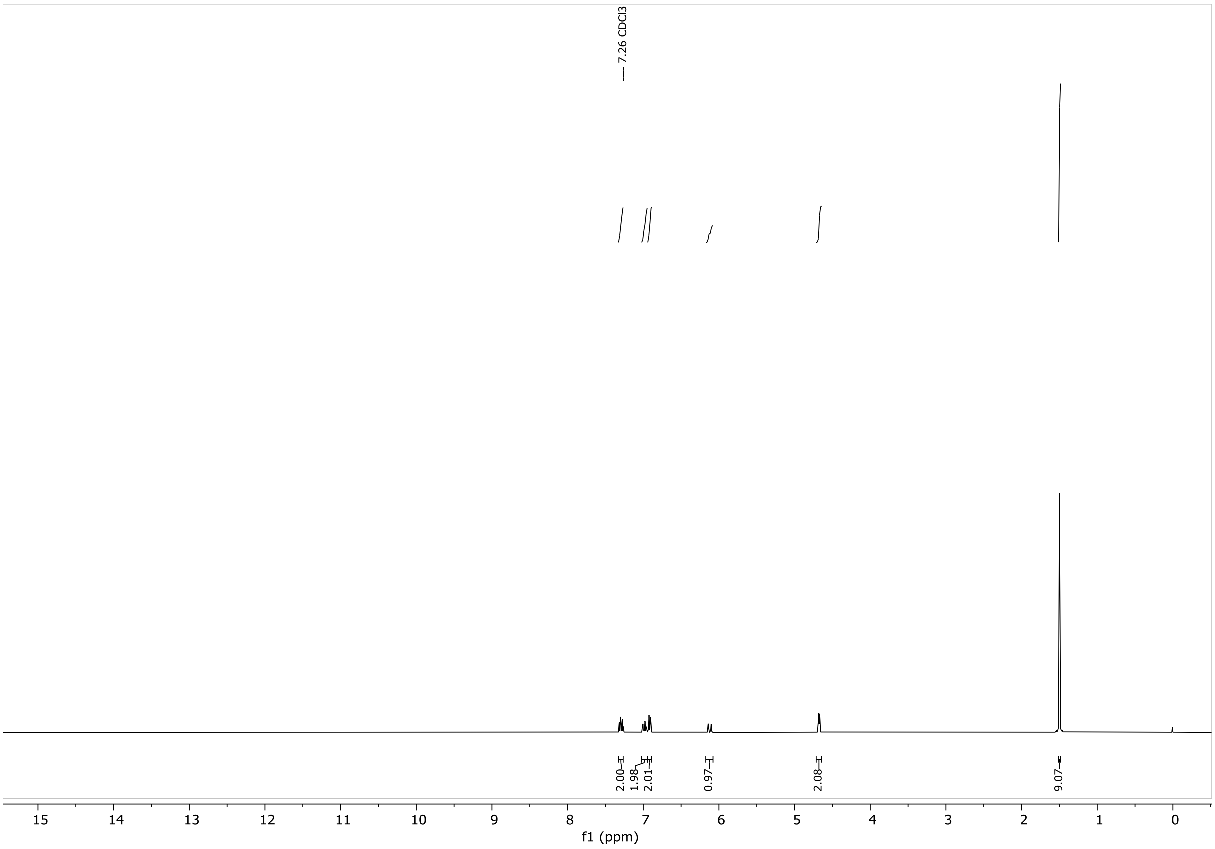

^{13}C-NMR (101 MHz, $CDCl_3$)

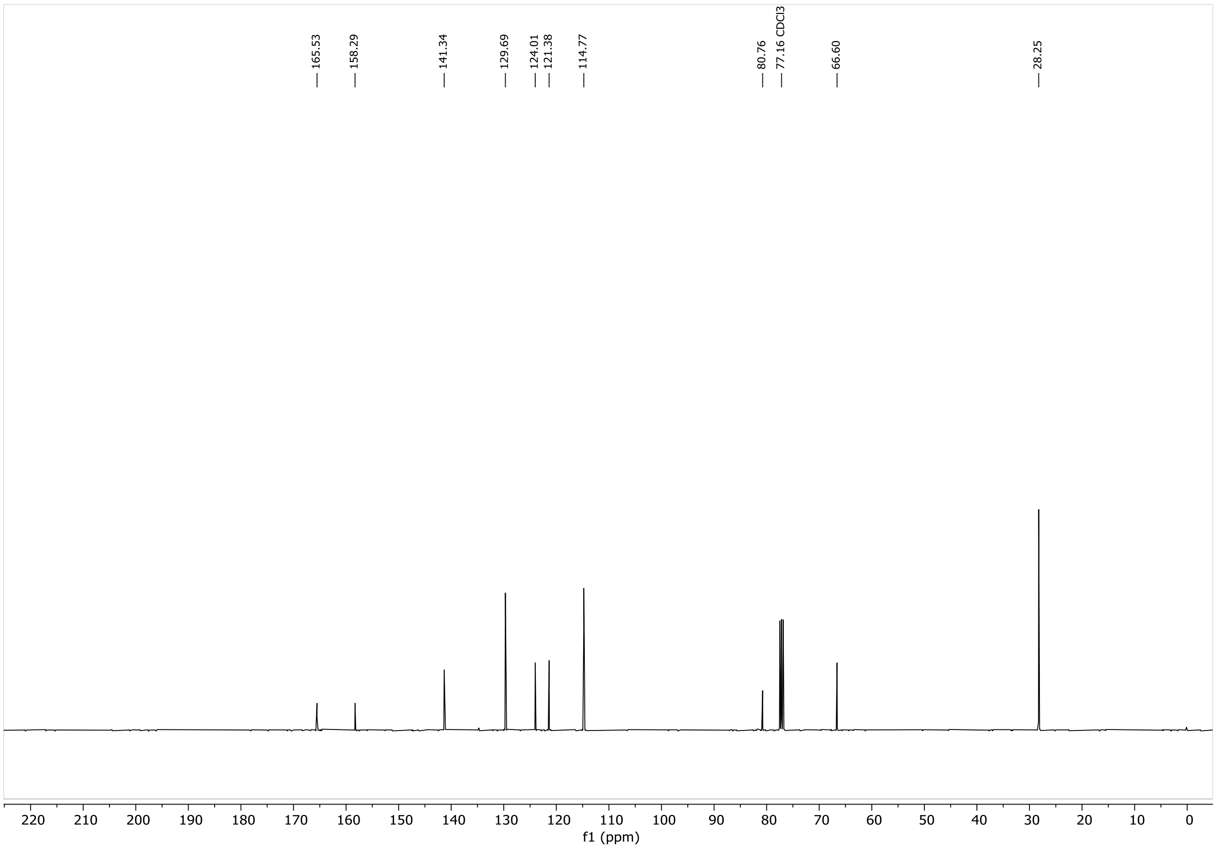

(*E*)-4-phenoxybut-2-enal (S3)

^{1}H NMR (600 MHz, $CDCl_3$)

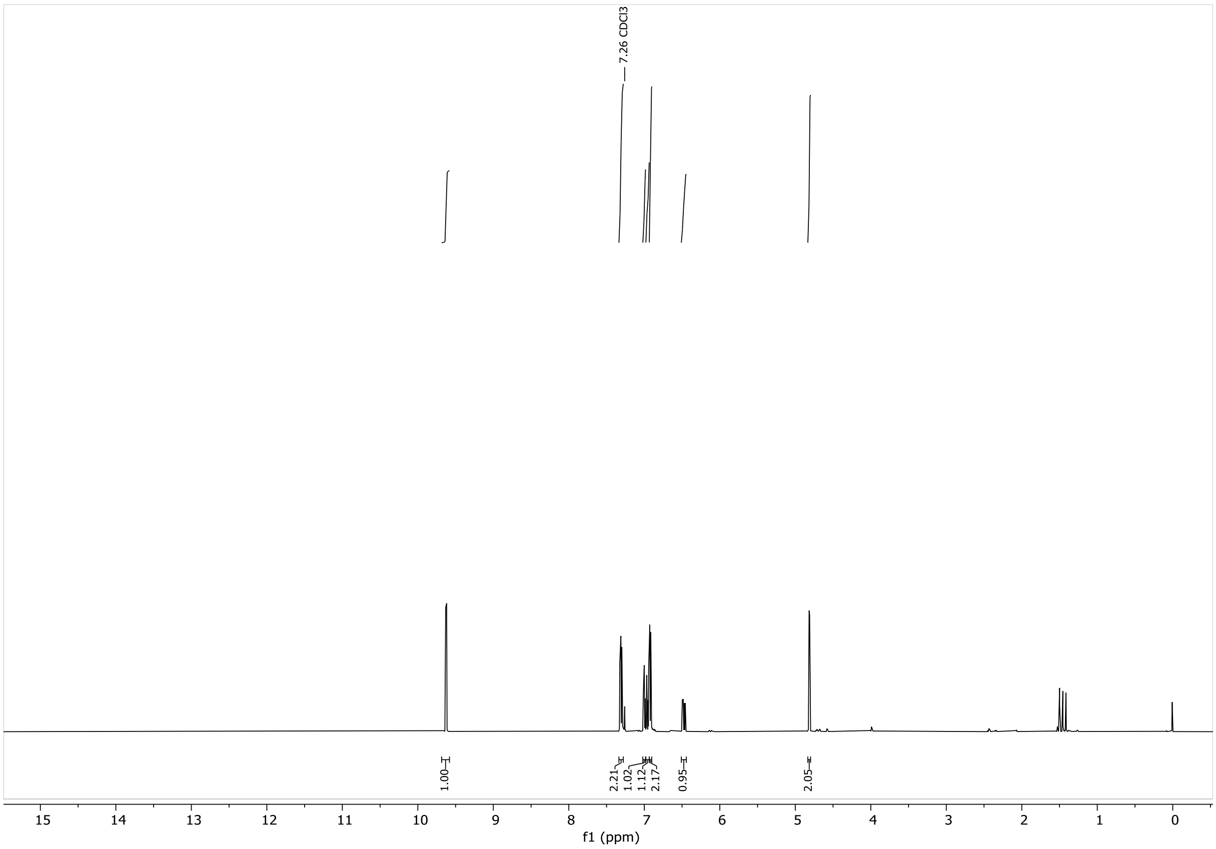

^{13}C-NMR (151 MHz, $CDCl_3$)

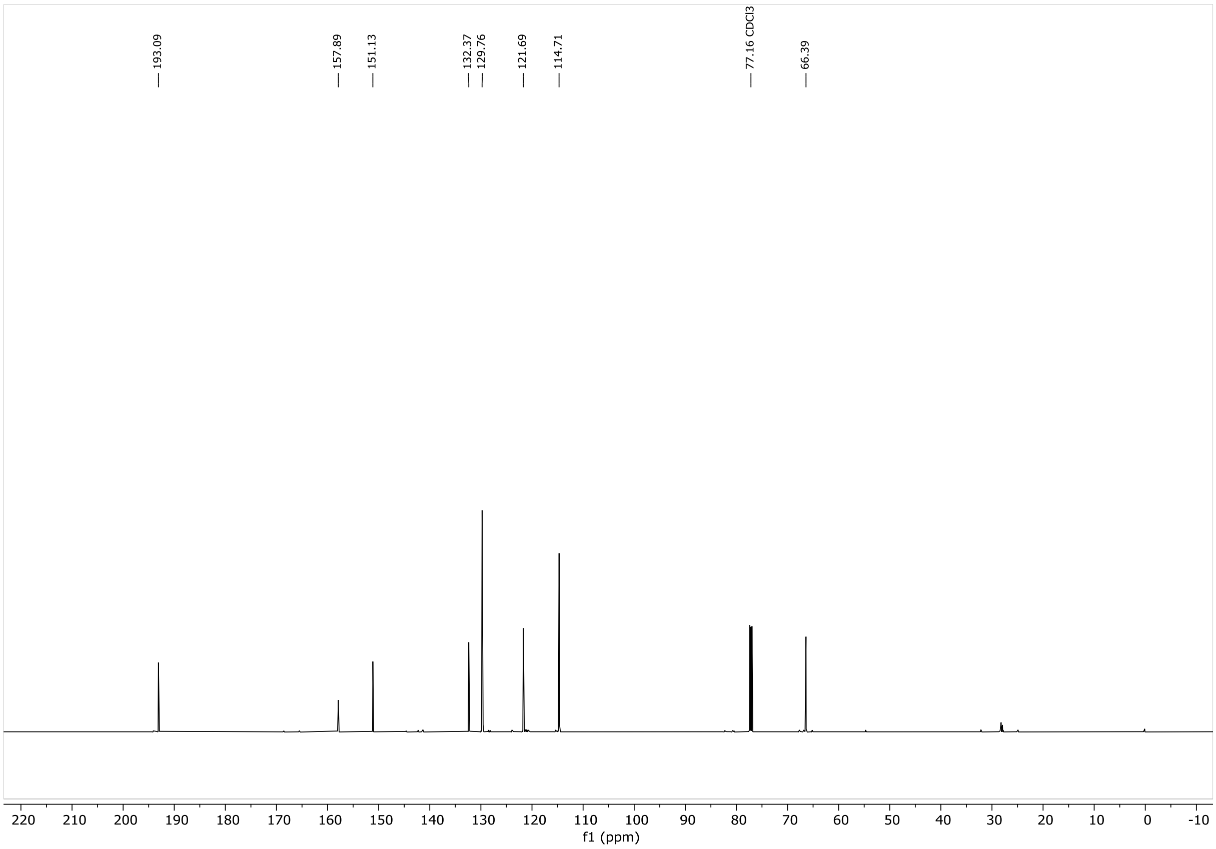

(*E*)-((penta-2,4-dien-1-yl-5,5-d2)oxy)benzene (16)

^{1}H NMR (600 MHz, $CDCl_3$)

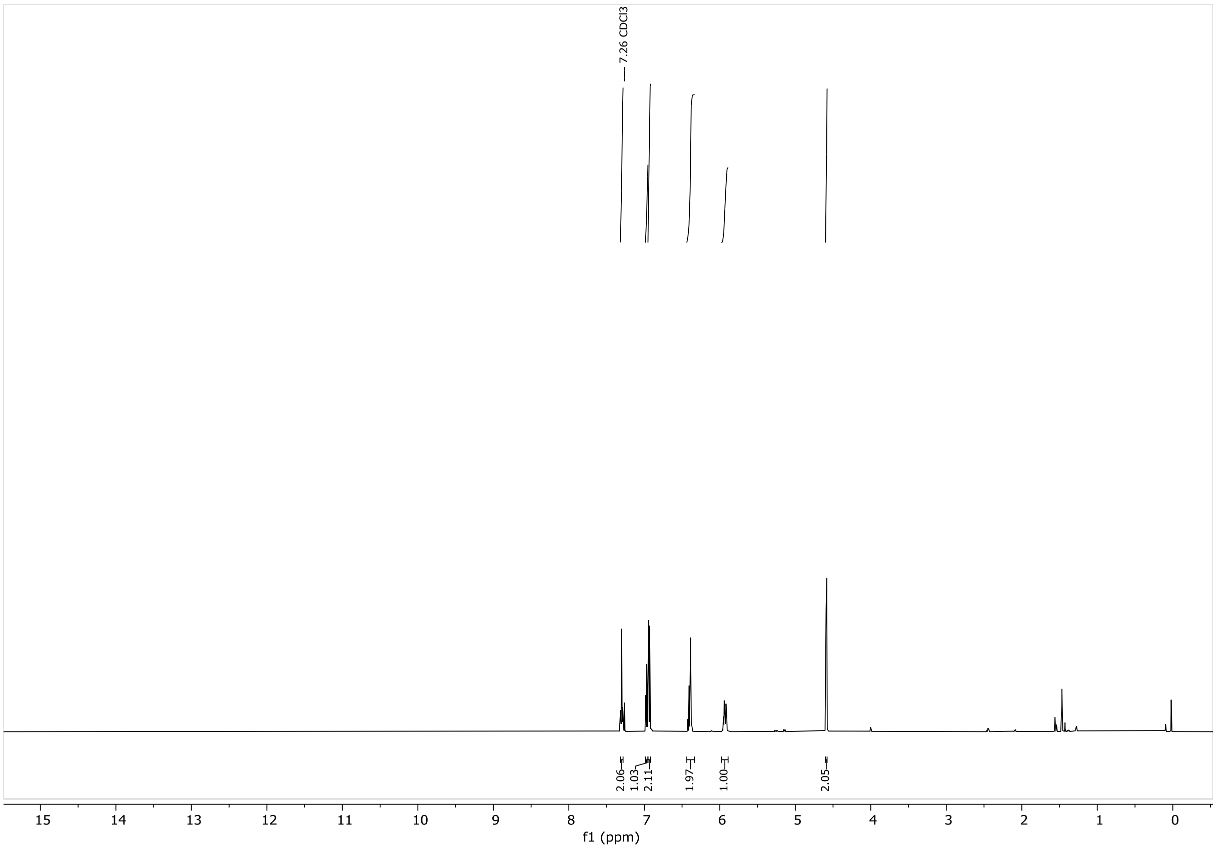

^{13}C-NMR (151 MHz, $CDCl_3$)

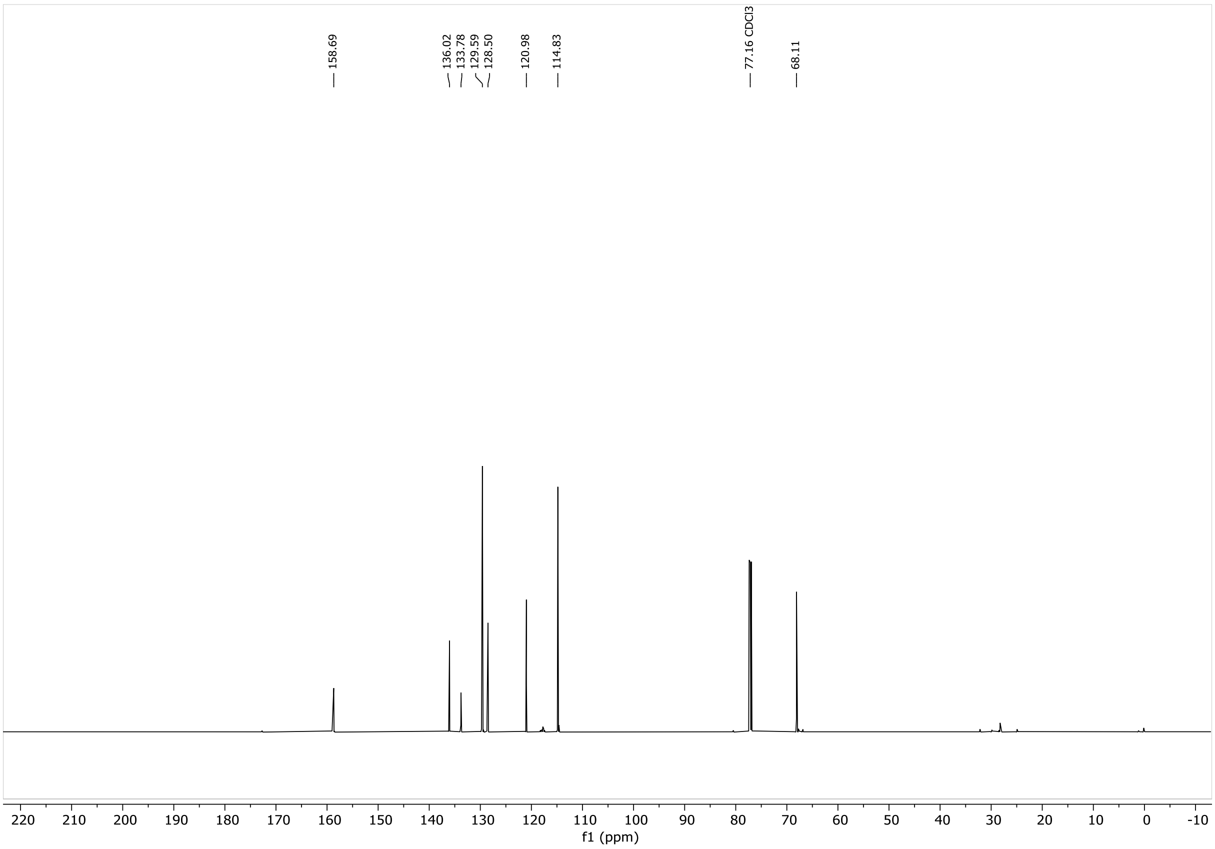

(*E*)-4-(penta-2,4-dien-1-yl-1,1-d2)phenol (17)

^{1}H NMR (600 MHz, $CDCl_3$)

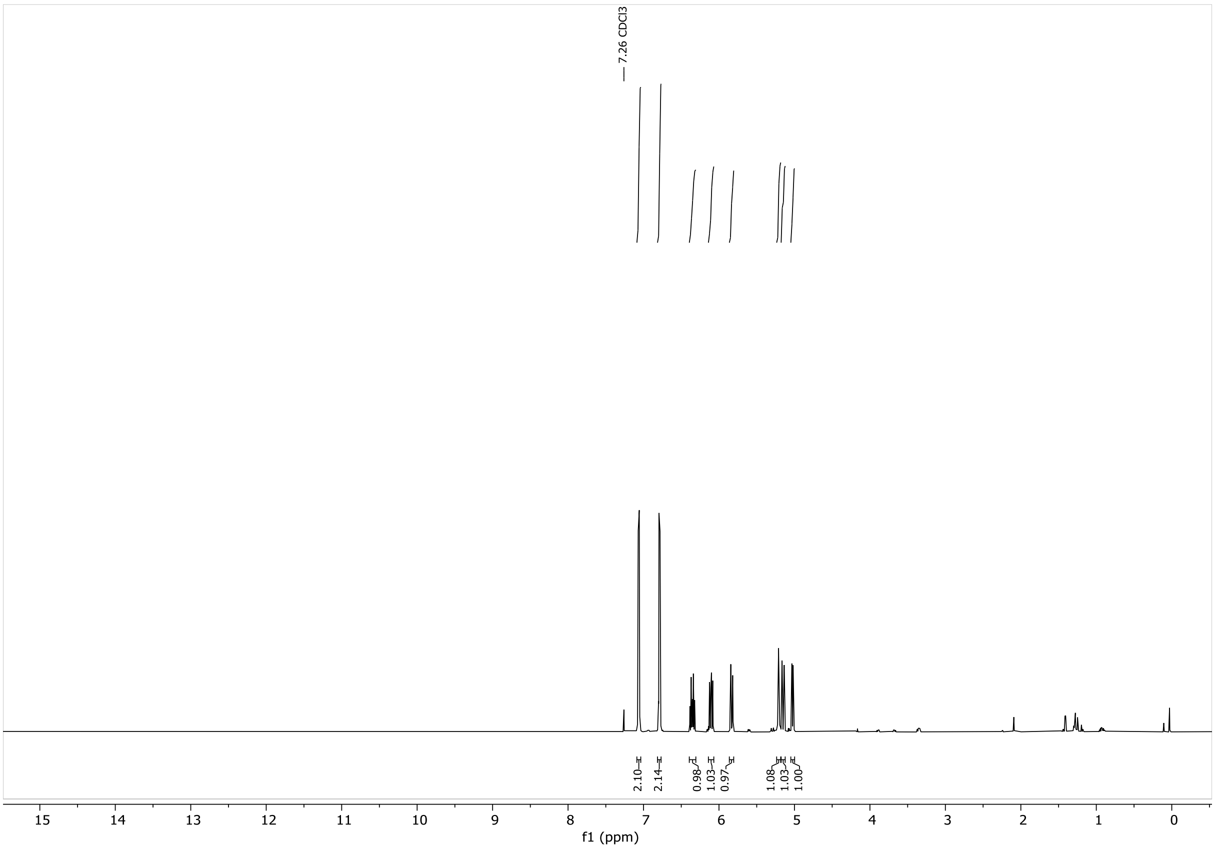

^{13}C-NMR (151 MHz, CDCl$_3$)

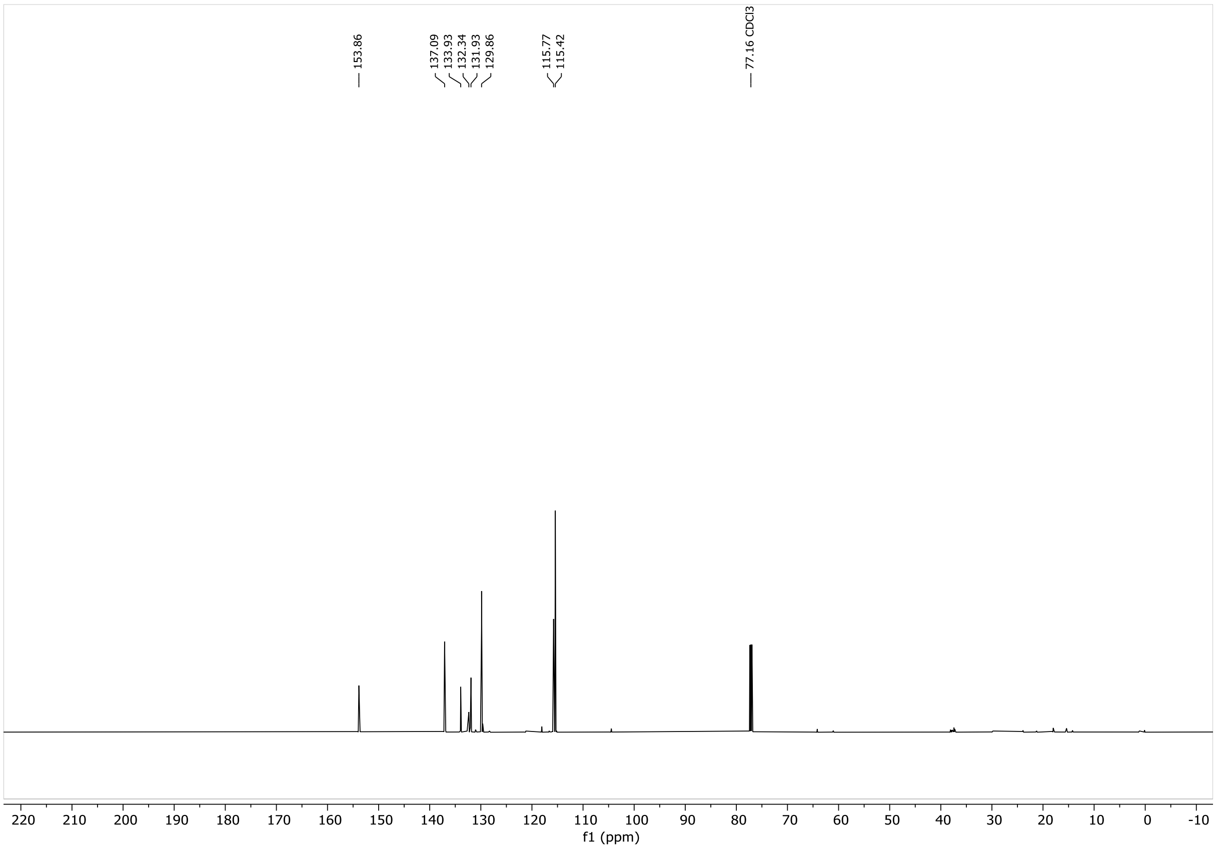

2-(penta-1,4-dien-3-yl)phenol (18)

^{1}H NMR (400 MHz, CDCl$_3$)

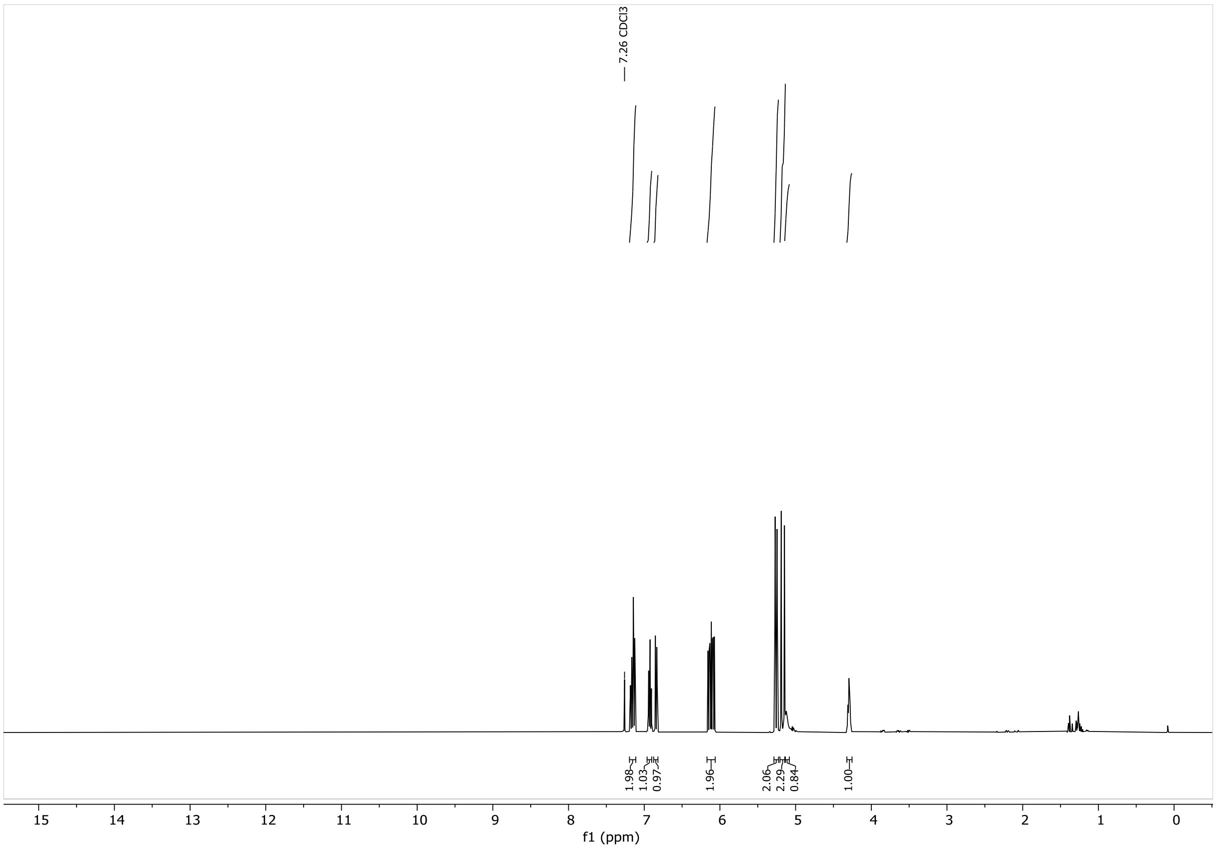

^{13}C-NMR (101 MHz, $CDCl_3$)

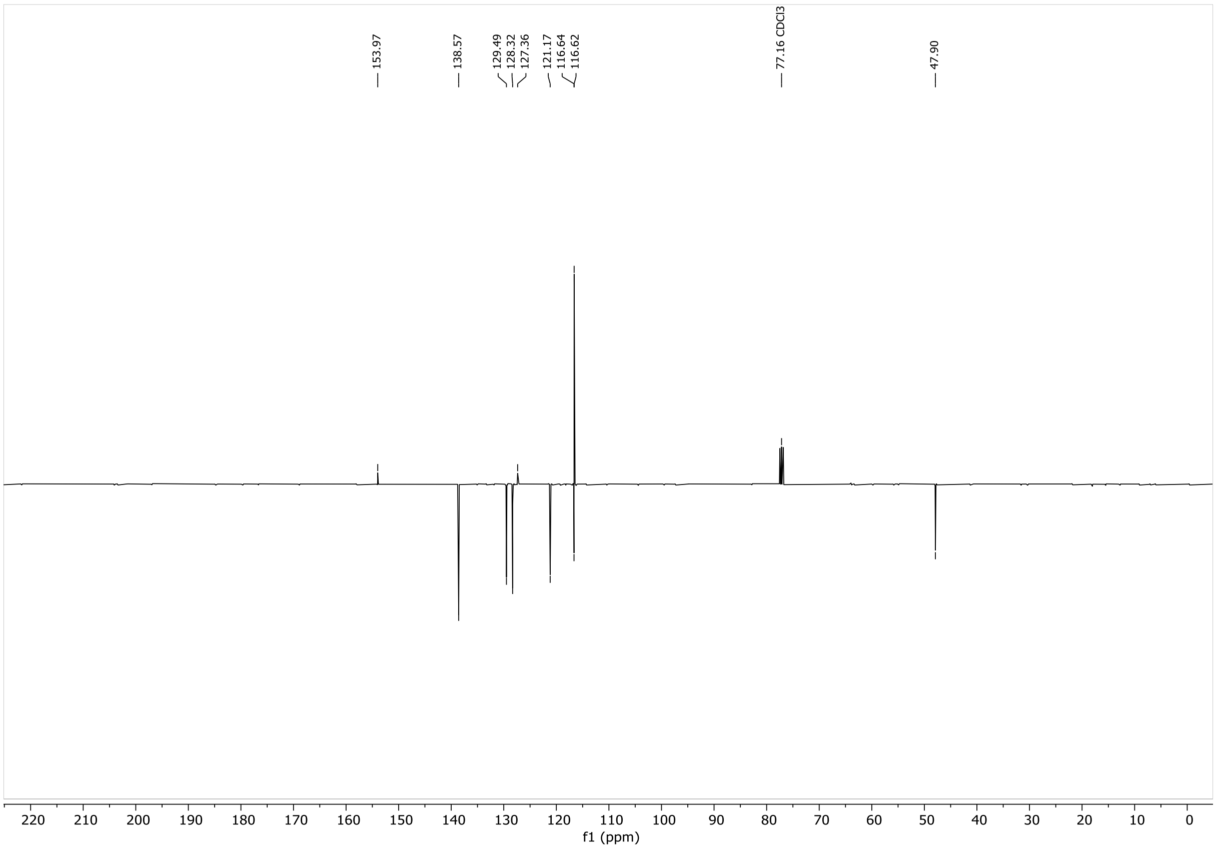

References

1 Z. He, Y. Chen, Y. Wang, J. Wang, J. Mo, B. Fu, Z. Wang, Y. Du and X. Zhou, *Chem. Commun.*, 2016, **52**, 8545–8548.

2 I. Bosque, E. Bagdatli, F. Foubelo and J. C. Gonzalez-Gomez, *J. Org. Chem.*, 2014, **79**, 1796–1804.

3 T.-Z. Li, C.-A. Geng and J.-J. Chen, *Tetrahedron Letters*, 2019, **60**, 151059.

4 J. J. Marugán, C. Manthey, B. Anaclerio, L. Lafrance, T. Lu, T. Markotan, K. A. Leonard, C. Crysler, S. Eisennagel, M. Dasgupta and B. Tomczuk, *J. Med. Chem.*, 2005, **48**, 926–934.

5 Y. Li, S.-H. Chen, T.-M. Ou, J.-H. Tan, D. Li, L.-Q. Gu and Z.-S. Huang, *Bioorganic & Medicinal Chemistry*, 2011, **19**, 2074–2083.

6 D. C. Harrowven, T. Woodcock and P. D. Howes, *Angew. Chem. Int. Ed.*, 2005, **44**, 3899–3901.
